# rCASC: reproducible classification analysis of single-cell sequencing data

**DOI:** 10.1093/gigascience/giz105

**Published:** 2019-09-08

**Authors:** Luca Alessandrì, Francesca Cordero, Marco Beccuti, Maddalena Arigoni, Martina Olivero, Greta Romano, Sergio Rabellino, Nicola Licheri, Gennaro De Libero, Luigia Pace, Raffaele A Calogero

**Affiliations:** 1 Department of Molecular Biotechnology and Health Sciences, University of Torino, Via Nizza 52, 10125 Torino, Italy; 2 Department of Computer Science, University of Torino, Corso Svizzera 185, 10149 Torino, Italy; 3 Department of Oncology, University of Torino, SP142, 95, 10060 Candiolo (TO), Italy; 4 Department Biomedizin, University of Basel, Hebelstrasse 20, 4031 Basel, Switzerland; 5 Italian Istitute for Genomic Medicine, IIGM, c/o IRCCS 10060 Candiolo (TO), Italy

**Keywords:** single-cell data preprocessing, workflow, GUI, clustering, cluster stability metrics, cluster-specific gene signature

## Abstract

**Background:**

Single-cell RNA sequencing is essential for investigating cellular heterogeneity and highlighting cell subpopulation-specific signatures. Single-cell sequencing applications have spread from conventional RNA sequencing to epigenomics, e.g., ATAC-seq. Many related algorithms and tools have been developed, but few computational workflows provide analysis flexibility while also achieving functional (i.e., information about the data and the tools used are saved as metadata) and computational reproducibility (i.e., a real image of the computational environment used to generate the data is stored) through a user-friendly environment.

**Findings:**

rCASC is a modular workflow providing an integrated analysis environment (from count generation to cell subpopulation identification) exploiting Docker containerization to achieve both functional and computational reproducibility in data analysis. Hence, rCASC provides preprocessing tools to remove low-quality cells and/or specific bias, e.g., cell cycle. Subpopulation discovery can instead be achieved using different clustering techniques based on different distance metrics. Cluster quality is then estimated through the new metric "cell stability score" (CSS), which describes the stability of a cell in a cluster as a consequence of a perturbation induced by removing a random set of cells from the cell population. CSS provides better cluster robustness information than the silhouette metric. Moreover, rCASC's tools can identify cluster-specific gene signatures.

**Conclusions:**

rCASC is a modular workflow with new features that could help researchers define cell subpopulations and detect subpopulation-specific markers. It uses Docker for ease of installation and to achieve a computation-reproducible analysis. A Java GUI is provided to welcome users without computational skills in R.

## Findings

### rCASC: a single-cell analysis workflow designed to provide data reproducibility

Since the end of the 90s omics high-throughput technologies have generated an enormous amount of data, reaching today an exponential growth phase. The analysis of omics big data is a revolutionary means of understanding the molecular basis of disease regulation and susceptibility, and this resource is made accessible to the biological/medical community via bioinformatics frameworks. However, owing to the increasing complexity and the fast evolution of omics methods, the reproducibility crisis [1, 2] demands that we find a way to guarantee robust and reliable results to the research community [3].

Single-cell analysis is instrumental to understanding the functional differences among cells within a tissue. Individual cells of the same phenotype are commonly viewed as identical functional units of a tissue or an organ. However, single-cell sequencing results [4] suggest the presence of a complex organization of heterogeneous cell states that together produce system-level functionalities. A mandatory element of single-cell RNA sequencing (RNA-seq) is the availability of dedicated bioinformatics workflows.

To the best of our knowledge, rCASC is the only computational framework that provides both computational and functional reproducibility for an integrated analysis of single-cell data, from count generation to cell subpopulation identification. It is one of the tools developed under the umbrella of the Reproducible Bioinformatics project [5, 8], an open-source community aimed at providing to biologists and medical scientists an easy-to-use and flexible framework, which also guarantees the ability to reproduce results independently by the underlying hardware, using Docker containerization (computational reproducibility). The Reproducible Bioinformatics project was founded and is maintained by the research team of the Elixir node at the University of Turin. An example of stand-alone hardware/software infrastructure for bulk RNA-seq, developed within the Reproducible Bioinformatics project, was described by Beccuti et al. [9]. Indeed, it was developed following the best-practice rules for reproducible computational research, proposed in 2013 by Sandve et al. [6]. It is also listed within the tools developed by the Italian Elixir node [7].

**Figure 1:**
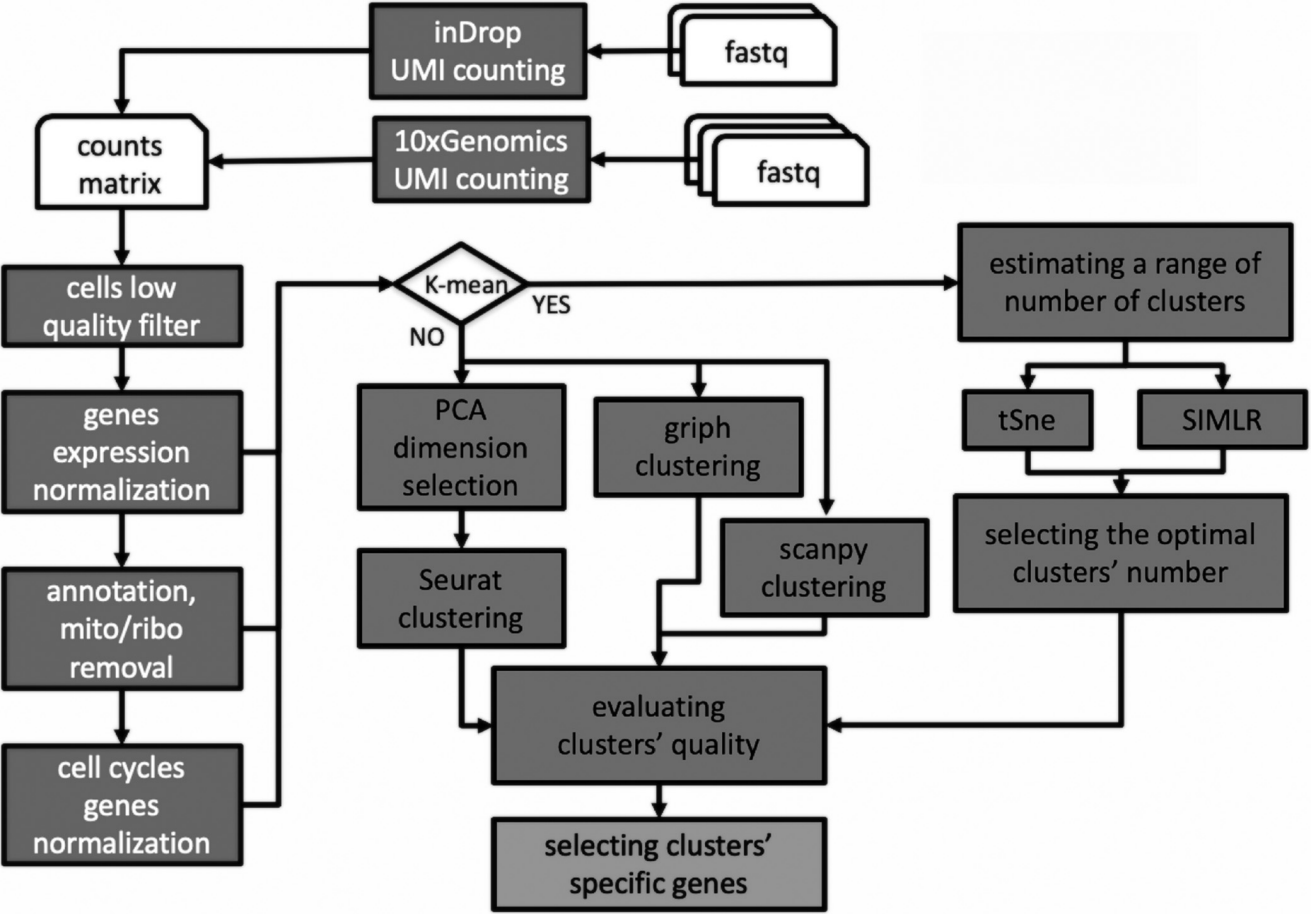
rCASC workflow. Dark gray boxes with white characters indicate preprocessing tools. Dark grey boxes with black characters define clustering tools. Light grey box with black characters indicates gene signature tools.

All the computational tools in rCASC are embedded in Docker images stored in a public repository on the Docker hub. Parameters are delivered to Docker containers via a set of R functions, part of the rCASC R github package [10]. To simplify the use of the rCASC package for users without scripting experience, R functions can be controlled by a dedicated GUI, integrated in the 4SeqGUI tool previously published by us [9], which is also available as a github package [11]. rCASC is specifically designed to provide an integrated analysis environment for cell subpopulation discovery. The workflow allows the direct analysis of fastq files, generated with 10X Genomics and inDrop platforms, or count matrices. Therefore, rCASC provides raw data preprocessing, subpopulation discovery via different clustering approaches, and cluster-specific gene signature detection. The key elements of the rCASC workflow are shown in Fig. 1, and the main functionalities are summarized in the Methods section. A detailed description of the rCASC functions is also available in the vignettes section of the rCASC github [10].

**Figure 2:**
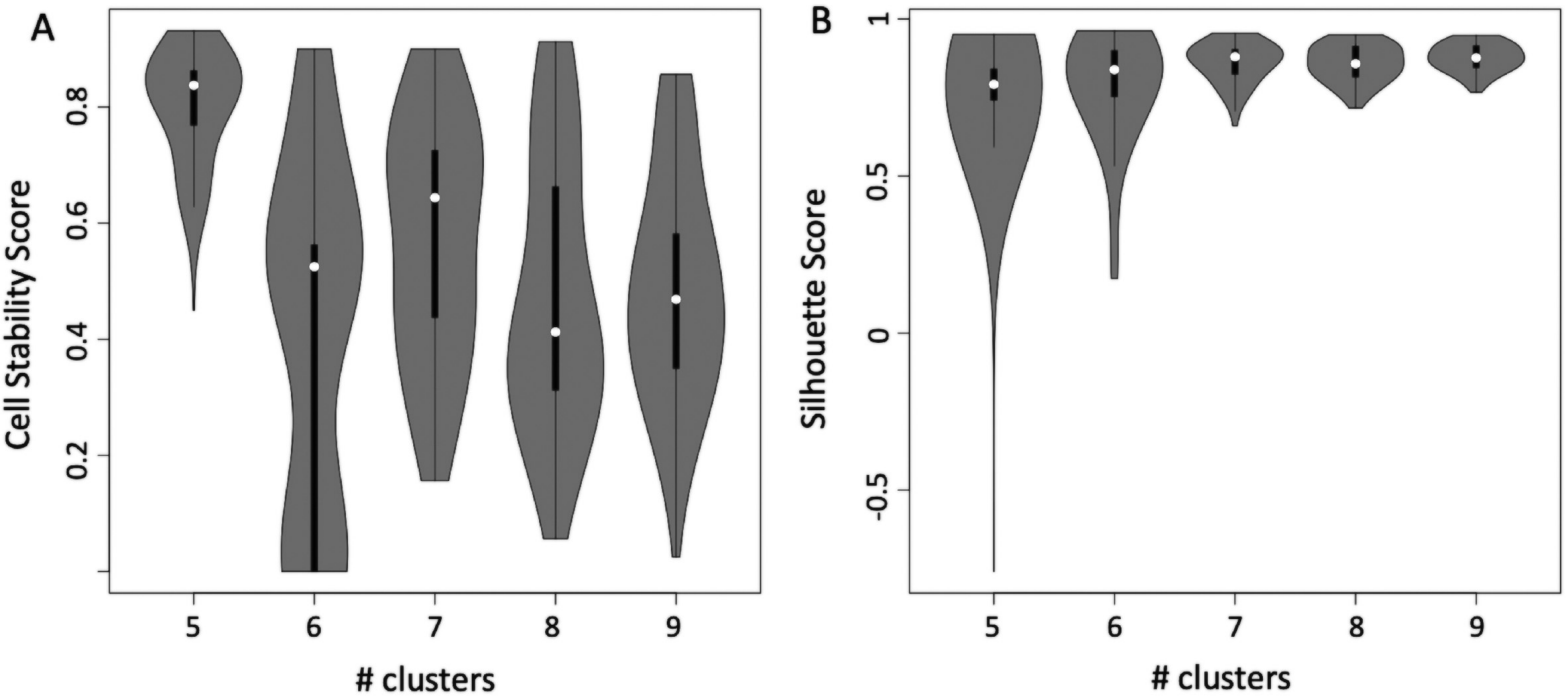
Cell stability score vs silhouette score calculated on the dataset of Pace et al. [24] (see Supplementary file section 8) using SIMLR over a set of number of clusters ranging between 5 and 8. A, Cell stability score violin plot. Mean value and data dispersion suggest that the best number of clusters is 5. Cells remain in the same cluster ∼80% of the time, repeating the clustering upon random removal of 10% of the cells. B, Silhouette score (SS) violin plot. Mean value of the SS distribution does not provide clear evidence that one clustering condition is better than another. Furthermore, the dispersion of the SS value shrinks as the number of clusters increases.

The overall characteristics of rCASC were compared with 4 other workflows for single-cell analysis (Fig. 2): (i) simpleSingleCell, Bioconductor workflow package [12]; (ii) Granatum, web-based single-cell RNA-seq analysis suite [13]; (iii) SCell, graphical workflow for single-cell analysis [14]; and (iv) R toolkit Seurat [15]. The comparison was based on the following elements: (i) supported single-cell platforms, (ii) types of tools provided by the workflow, (iii) type of reproducibility granted by the workflow, and (iv) tool flexibility.

**Figure 3:**
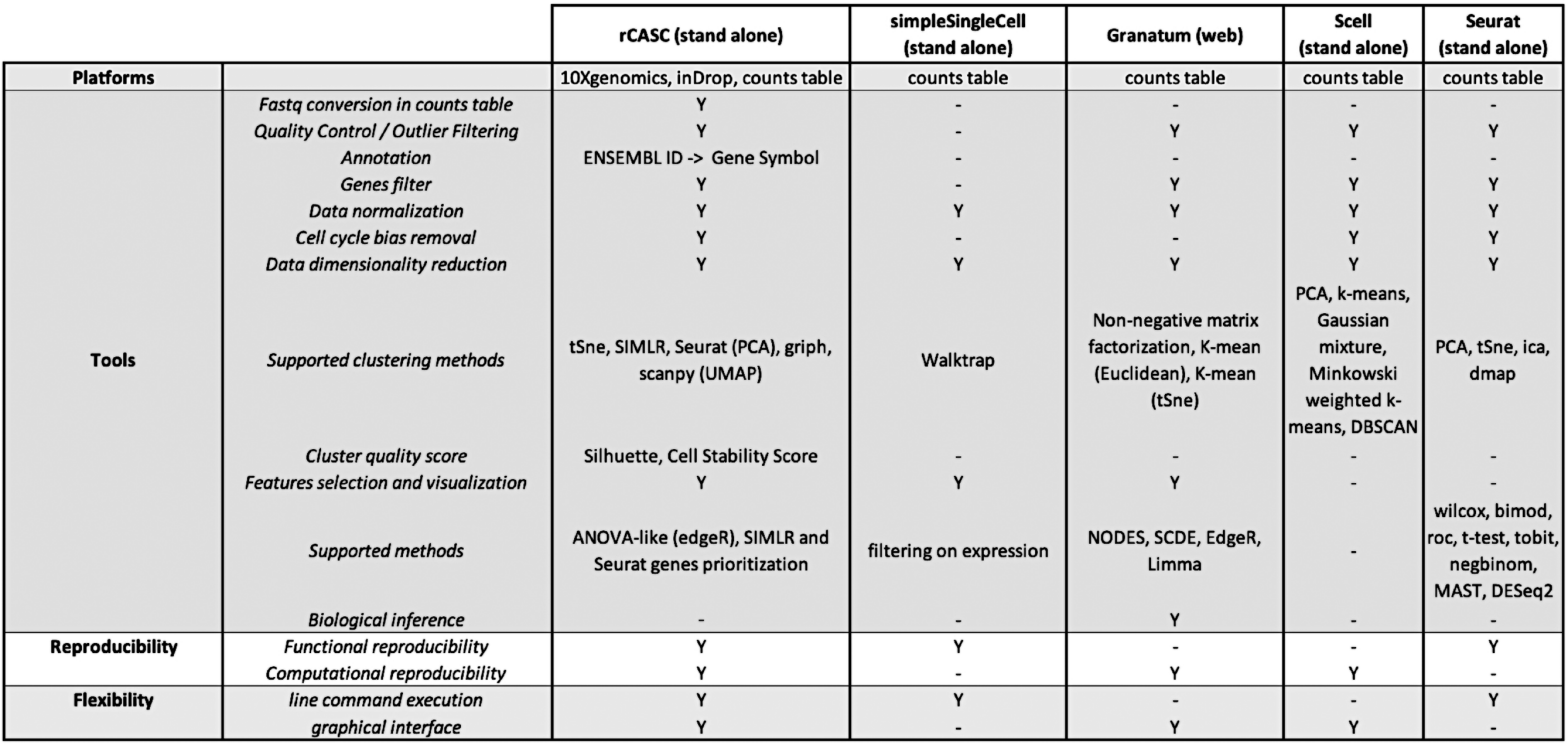
Comparison of analysis features available in rCASC and in the other single-cell analysis workflows.

**Figure 4:**
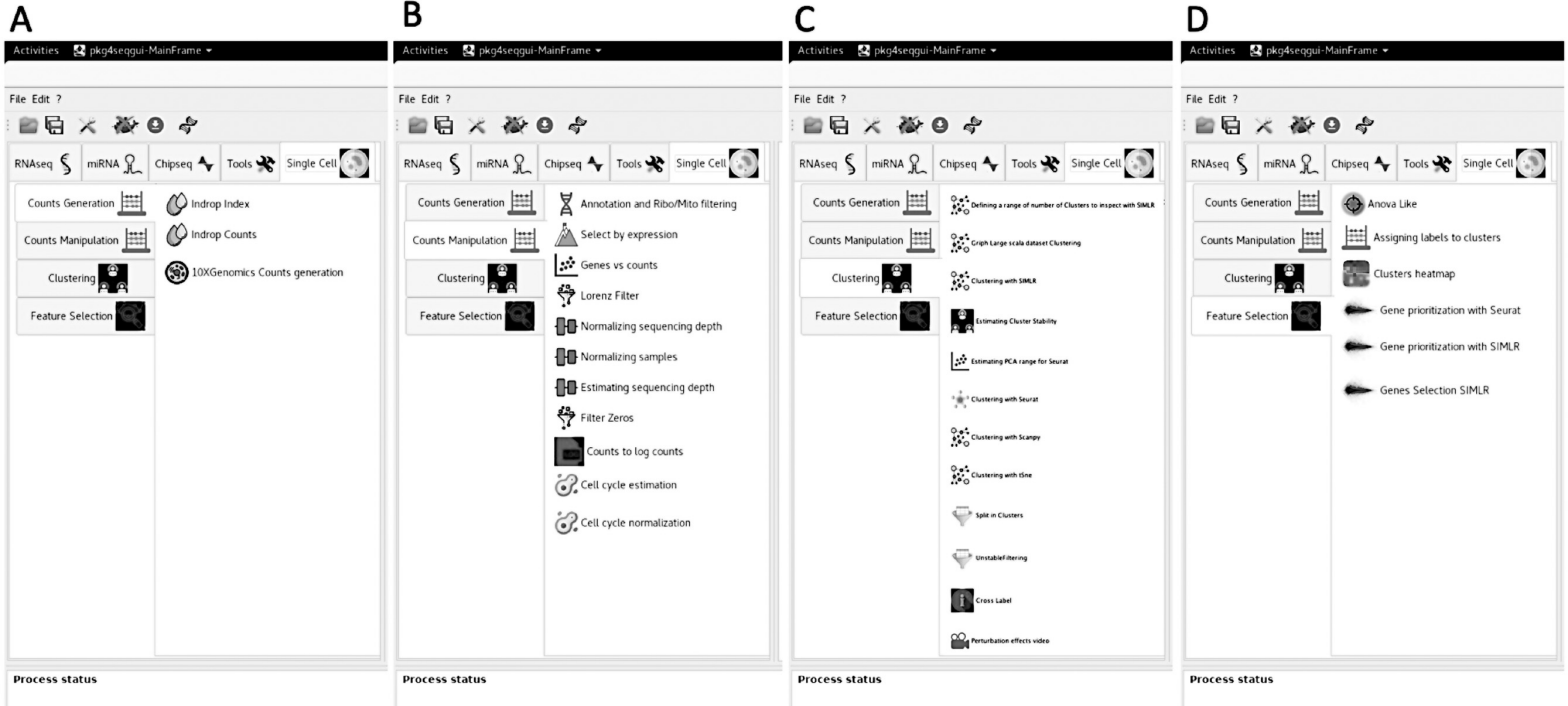
rCASC graphical interface within 4seqGUI. A, Count table generation menu: this set of functions is devoted to the conversion of fastq to a count table. B, Count table manipulation menu: this set of functions provides inspection, filtering, and normalization of the count table. C, Clustering menu: these functions allow the use of SIMLR, t-SNE, Seurat, griph, and scanpy to group cells in subpopulations. D, Feature selection menu: this set of functions allows the identification of cluster-specific subsets of genes and their visualization using heat maps.

rCASC is the only workflow providing support at the fastq level because all the other packages require as input the processed count table. Cell quality control and outlier identification is available in all the workflows but Granatum. Association of ENSEMBL gene IDs to gene symbols is only provided by rCASC. All the workflows provide gene-filtering tools but simpleSingleCell. All packages provide normalization procedures to be applied to raw count data. However, rCASC is the only tool providing both Seurat specific normalization [15] and count-depth specific normalization [16]. The workflows implement different data reduction and clustering methods. rCASC integrates 4 clustering tools, i.e., Seurat [15], SIMLR [17], griph [18], and scanpy [19], which differ in the metrics driving the clustering analysis. Cluster stability is an important topic in clustering (for a review see von Luxburg [20]). Stability measurement, taking advantage of bootstrapping, was also addressed by Hennig [21]. Specifically, Hennig uses the Jaccard index to evaluate the overall stability of each cluster. In rCASC, we have implemented a cell stability score (CSS), which uses the Jaccard index to estimate the stability of each cell in each cluster. The CSS provides an enhanced description of each cluster because it allows the identification of a subset of cells, in any cluster, that are particularly sensitive to perturbation of the overall dataset structure, i.e., cell bootstrapping. Moreover, the cluster stability measurement proposed by Henning was included in rCASC. Specifically, we have implemented the “clusterboot” function from the fpc R package [22], which allows the evaluation of cluster stability using a personalized clustering function (see Supplementary file section 5.3). To the best of our knowledge, rCASC is the only workflow performing clustering in the presence of data perturbation, i.e., removal of a subset of cells, and measuring cluster quality using the CSS (a cluster quality metric developed by us, which measures the persistence of each cell in a cluster upon data perturbation; see Supplementary file section 5.1) and silhouette score (SS), a cluster quality metric measuring the consistency within clusters of data. In our experiments, CSS provides a better estimation of cluster stability compared to that of SS (Fig. 2). Gene feature selection approaches are implemented in a different way in the 5 workflows. Granatum is the only one providing biological inference. Granatum and Seurat implement various statistical methods to detect cluster-specific gene signatures (Fig. 3). rCASC embeds an ANOVA-like statistics derived from the EdgeR Bioconductor package [23] and Seurat/SIMLR gene prioritization procedures (see Supplementary file section 7). Visualization of gene signatures by heat map, with cells colored on the basis of gene expression, is only provided by rCASC (see Supplementary file Fig. 51). Considering reproducibility, only rCASC provides both computational and functional reproducibility. Finally, rCASC is the only one providing both a command line interface and GUI (Fig. 4).

Finally, rCASC was used to re-analyze the single-cell dataset from Pace et al. [24]. In this article, the authors highlighted that Suv39h1-defective CD8^+^ T cells show sustained survival and increased long-term memory reprogramming capacity. Our re-analysis extends the information described by Pace et al. [24], suggesting the presence of an enriched Suv39h1-defective memory subset. A complete description of the above analysis is available in section 8 of the supplementary file.

## Methods

### Count table generation

The inDrop single-cell sequencing approach was originally published by Klein et al. [25]. The authors subsequently published the detailed protocol in 2017 [26]. In rCASC, the generation of the count table starting from fastq files refers to version 2 of the inDrop chemistry described in Zilionis et al. [26], which is commercially distributed by 1CellBio. The procedure described in the inDrop github [27] is embedded in a Docker image. The rCASC function "indropIndex" allows the generation of the transcript index required to convert fastq in counts, and the "indropCounts" function converts reads in unique molecular identifier (UMI) counts. 10X Genomics Cellranger is packed in a Docker image and the function "cellrangerCount" converts fastq to UMI matrix using any of the genome indexes with the "cellrangerIndexing" function. A detailed description of the count table generation is available in Supplementary file section 2.

### Count table exploration and manipulation

rCASC provides various data inspection and preprocessing tools.

**Figure 5:**
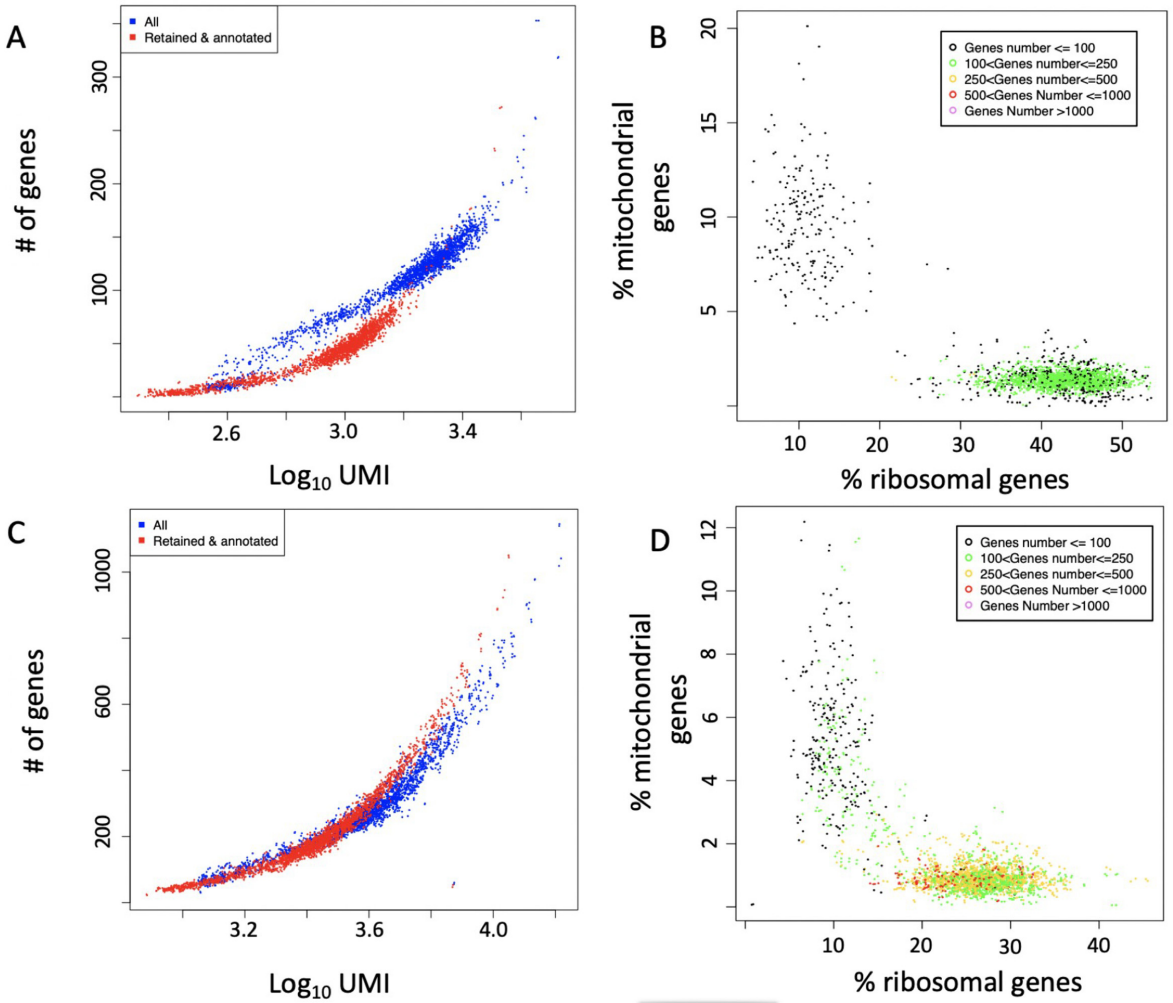
genesUmi plots the number of detectable genes in each cell (a cell is called present if it is supported by at least *N* UMI/reads; suggested values are *N* = 3 for UMI or *N* = 5 for smart-seq sequencing [28]) with respect to the number of UMI per cell. mitoRiboUmi calculates the percentage of mitochondrial and ribosomal genes with respect to the total number of detected genes in each cell. It plots the percentage of mitochondrial genes with respect to the percentage of ribosomal genes. Cell color indicates number of detected genes. A, genesUmi plot for resting CD8+ T cells [24], sequencing average 83,000 reads/cell. B, mitoRiboUmi plot for resting CD8+ T cells [24]. The majority of the cells with <100 detected genes group together, and they are characterized by a high relative percentage of mitochondrial genes and low relative percentage of ribosomal genes. Remaining cells are characterized by few detectable genes, 100–250 genes/cell, with a percentage of ribosomal genes >30%. C, genesUmi plot for *Listeria*-activated CD8+ T cells [24], sequencing average 83,000 reads/cell. It is notable that the activated cells show a wider range of detectable genes with respect to resting cells (B). D, mitoRiboUmi plot for *Listeria*-activated CD8+ T cells [24]. The majority of the cells are characterized by >100 genes and they show a low percentage of mitochondrial genes and percentage of ribosomal genes between 15% and 35%. The remaining cells, with <100 detected genes, group together and are characterized by a high relative percentage of mitochondrial genes and low relative percentage of ribosomal genes.

The "genesUmi" function generates a plot where the number of detected genes is plotted for each cell with respect to the number of UMI (Fig. 5A and C).

mitoRiboUmi calculates the percentage of mitochondrial/ribosomal genes with respect to the total number of detected genes in each cell and plots the percentage of mitochondrial genes with respect to percentage of ribosomal genes. Cell color indicates the number of detected genes (Fig. 5B and D). mitoRiboUmi allows researchers to identify cells with low information content, i.e., those cells with few detectable genes, e.g., <100 genes/cell, little ribosomal content, and high content of mitochondrial genes, which indicate cell stress [29].

The function "scannobyGtf" uses ENSEMBL gtf and the R package refGenome to associate gene symbol with the ENSEMBL gene ID. Furthermore, scannobyGtf allows one to remove mitochondrial/ribosomal genes (Fig. 5A and C) and “stressed” cells detectable with the mitoRiboUmi function (Fig. 5B and D).

The function "lorenzFilter" embeds the Lorenz statistics developed by Diaz et al. [14], a cell quality statistic correlated with cell live-dead staining (see Supplementary file section 3.3). Specifically, the outlier filtering for single-cell RNA-seq experiments designed by Diaz et al. estimates which genes are expressed at background levels in each sample; then samples with significantly high background levels are discarded [14].

As count table preprocessing steps, we implemented the functions "checkCountDepth/scnorm" to detect the presence of sample-specific count–depth relationship [16] (i.e., the relationship existing between transcript-specific expression and sequencing depth) and to adjust the count table for it. Specifically, checkCountDepth initially executes a quantile regression, thus estimating the dependence of transcript expression on sequencing depth for every gene. Then, genes with similar dependence are aggregated (see Supplementary file section Fig. 21). Scnorm, after executing checkCountDepth, performs a new quantile regression to estimate scale factors within each group of genes. Then, sequencing depth adjustment is done within each group using the estimated scale factors. Furthermore, we added 2 other functions "recatPrediction" and "ccRemove," which are based, respectively, on Liu et al. [30] and Barron and Li [31]. The function recatPrediction organizes the single-cell data to reconstruct cell cycle pseudo time-series and is used to understand whether a cell cycle effect is present. The above function embeds reCAT software [30], which models the reconstruction of time-series as a traveling salesman problem, thus identifying the shortest possible cycle by passing through each cell exactly once and returning to the start. Because the traveling salesman problem is an NP-hard problem, reCAT is based on a heuristic algorithm, which is used to find the solution.

The "ccRemove" function is instead based on the work of Barron and Li [31] and embeds their scLVM (single-cell latent variable model) algorithm, which uses a sophisticated Bayesian latent variable model to reconstruct hidden factors in the expression profile of the cell cycle genes. This algorithm is able to remove cell cycle effect from real single-cell RNA-seq datasets. Thus, ccRemove is used to mitigate the cell cycle effect of the inter-sample transcriptome, when it is detected by the "recatPrediction" function (see Supplementary file sections 3.6 and 3.8).

### Clustering

For the identification of cell subpopulations we implemented 4 approaches: Seurat (RRID:SCR_016341) [15], SIMLR [17], griph [18], and scanpy [19]. Seurat is a toolbox for single-cell RNA-seq data analysis. We implemented in rCASC one of the clustering procedures present in the Seurat toolbox. The function "seuratPCAEval" has to be run before executing the clustering program to identify the “metafeatures,” i.e., the subset of principal componet analysis (PCA) components describing the relevant source of cell heterogeneity, to be used for clustering. The "seuratBootstrap" function implements data reduction and clustering. Specifically, cells undergo global scaling normalization, i.e., LogNormalize method, and scaling factor 10,000. Subsequently, a linear dimensional reduction is done using the range of principal components defined with "seuratPCAEval." Then, clustering is performed using the cell PCA scores. The Seurat clustering procedure, embedded in seuratBootstrap, is based on the Louvain modularity optimization algorithm. In contrast, SIMLR implements a *k*-mean clustering, where the number of clusters (i.e., *k*) is taken as input. SIMLR requires as input raw counts that are log_10_ transformed. SIMLR is capable of learning an appropriate cell-to-cell similarity metric from the input single-cell data and can exploit it for the clustering task. In the learning phase SIMLR identifies a distance metric that better fits the structure of the data by combining multiple Gaussian kernels [17]. Thus, the tool can deal with the large noise and dropout effects of single-cell data, which could not easily fit with specific statistical assumptions made by standard dimension reduction algorithms [17]. The function "simlrBootstrap" controls the clustering procedure and the function "nClusterEvaluationSIMLR," a wrapper for the R package griph [18], is exploited to estimate the (sub)optimal number “*k*” of clusters. Griph clustering [18] is based on Louvain modularity. The griph algorithm is closer to agglomerative clustering methods because every node is initially assigned to its own community and communities are subsequently built by iterative merging. Also, scanpy [19] uses for clustering a heuristic method based on modularity optimization.

We developed, for Seurat, SIMLR, griph, and scanpy, a procedure to measure the cluster quality on the basis of data structure. The rationale of our approach is that cells belonging to a specific cluster should be little affected by changes in the size of the dataset, e.g., removal of 10% of the total number of cells used for clustering. Thus, we developed a metric called CSS, which describes the persistence of a cell in a specific cluster upon jackknife resampling and therefore offers a peculiar way of describing cluster stability. A detailed description of the CSS metric is available in Supplementary file section 5.1. CSS is embedded in "seuratBootstrap," "simlrBootstrap," "scanpyBootstrap," and "griphBootstrap."

### Feature selection

**Figure 6:**
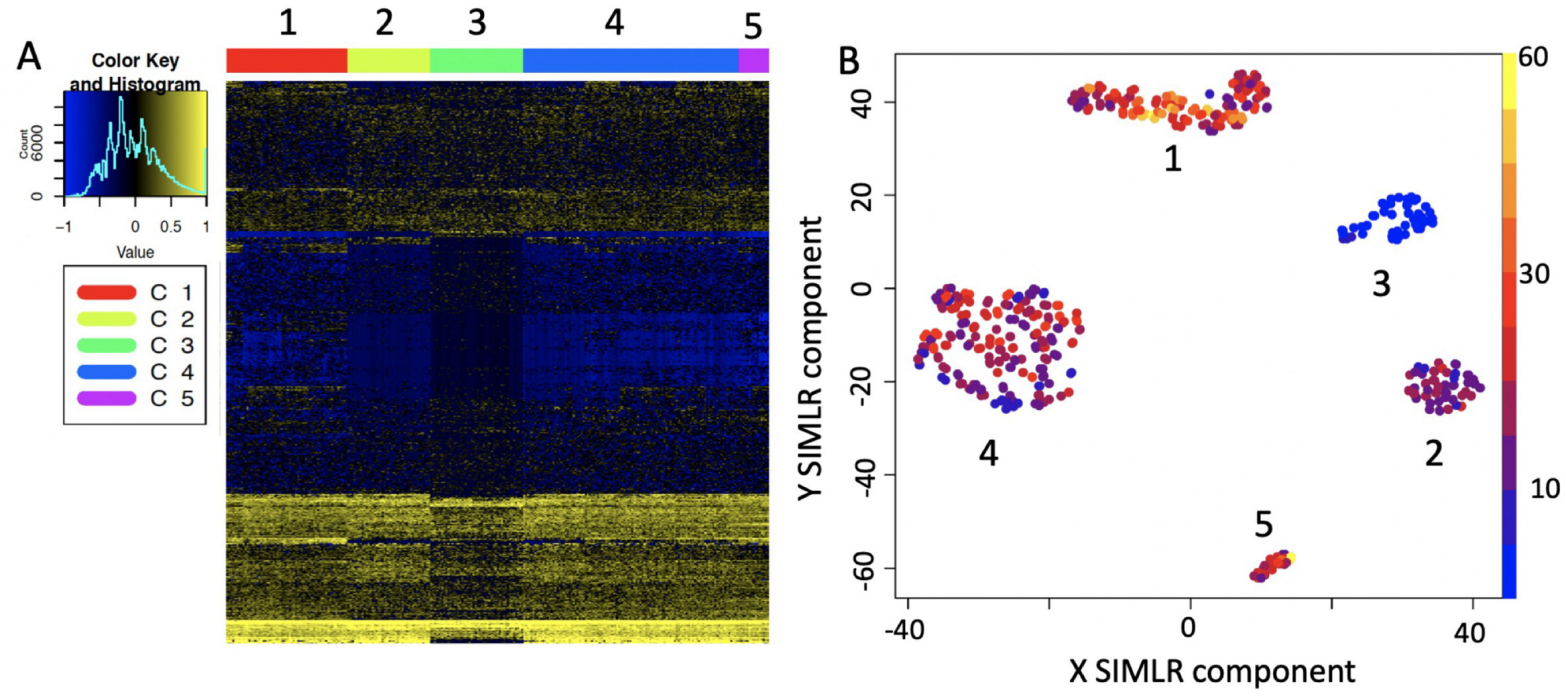
Heat map and cell expression plot for prioritized genes. A, Heat map for the set of 577 genes selected for Pace et al. [24] datasets (see Supplementary file section 8) by SIMLR prioritization. B, Nkg7 CPM expression in the cell clusters. Nkg7 is expressed in activated T cells (clusters 1, 2, 4, 5) [32] but not in resting T cells (cluster 3).

To select the most important features of each cluster we implemented in the "anovaLike" function the edgeR ANOVA-like method for single cells [23] and in the functions "seuratPrior" and "genesPrioritization/genesSelection," respectively, the Seurat and SIMLR gene prioritization methods. The "hfc" function allows visualization of the genes prioritized with the above methods as a heat map and provides plots of prioritized genes in each single cell (Fig. 6).

### Scalability

**Figure 7:**
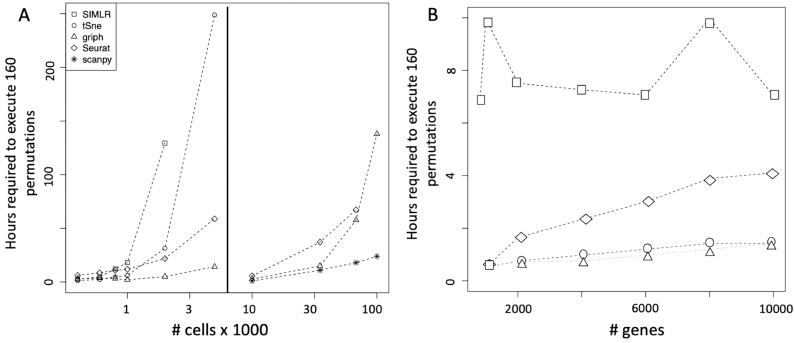
Scalability analysis of the clustering tools implemented in rCASC. A, Time required to perform 160 permutations as function of increasing number of cells on ∼20,000 genes. Left: SIMLR, t-SNE, Seurat, and griph clustering up to 5,000 cells was executed on a SeqBox [9] (1 x CPU i7–6770HQ 3.5 GHz [8 cores], 32 GB RAM, 1 TB SSD). Right: Seurat, griph, and scanpy analyses were extended until 101,000 cells using an SGI server (10 x CPU E5–4650 2.4 GHz [16 cores], 1 TB RAM, 30 TB SATA raid disk). B, Time required to perform 160 permutations as function of increasing number of genes on a set of 800 cells, analysis performed on a SeqBox.

To estimate the scalability of rCASC clustering we used the GSE106264 dataset made of 10,035 cells and published by Pace and coworkers in 2018 [24] and the 10,000/33,000/68,000 cells peripheral blood mononuclear cell (PBMC) human datasets, available at the 10X Genomics repository [33]. We randomly generated from the 10,035 cells (27,998 ENSEMBL gene IDs) the following subsets of cells: 400, 600, 800, 1,000, 2,000, and 5,000. Moreover for the subsets with >600 cells we randomly sampled the genes: 10,000, 8,000, 6,000, 4,000, 2,000, 1,000, 800. We ran SIMLR, t-SNE, griph, and Seurat using 160 permutations within SeqBox hardware [9]: Intel i7 3.5 GHz (4 cores), 32 GB RAM, and 500 GB SSD disk. SIMLR turned out to be the slowest and, given the above hardware implementation, it cannot allocate for the analysis >2,000 cells (Fig. 7A left panel). All the other tools were able to handle up to 5,000 cells within the limit of 32 GB of RAM. Computation time was nearly linear for all tools until 1,000 cells. Only griph clustering turned out to be nearly insensitive to the increasing number of cells (Fig. 7A). We extended, for Seurat, griph, and scanpy, the scalability analysis to 10,000, 33,000, 68,000, and 101,000 cells, using 10,000/33,000/68,000 cells from PBMC human datasets, available at the 10X Genomics repository [33], and a 101,000-cell dataset, made by assembling the aforementioned 33,000 and 68,000 PBMC datasets. The analysis was executed on an SGI server (10 x CPU E5–4650 2.4 GHz [16 cores], 1 TB RAM, 30 TB SATA raid disk) allocating 40 threads for each analysis. Scanpy outperformed the other 2 methods, and griph behaved slightly better than Seurat (Fig. 7A right panel).

All the above samples were preprocessed removing ribosomal/mitochondrial protein genes and cells with a total count of UMIs <100.

The computing time as a function of increasing number of genes had a quite limited effect on the overall computing time (Fig. 7B).

The definition of the computing time for an analysis depends on multiple parameters: (i) the number of permutations performed in parallel, (ii) the number of cells under analysis, (iii) the clustering tool in use, and (iv) the hardware used for the analysis. Concerning the amount of RAM required for each permutation run in parallel, for up to 5,000 cells the maximum amount of RAM required is ∼4 GB; from 10,000 to 100,000 cells, the maximum RAM required is ∼20 GB. Independently by the clustering approach and the size of the dataset, we suggest running ≥100 permutations to correctly estimate CSS.

## Availability of supporting data and materials

Snapshots of the code and test data are available from the *GigaScience* GigaDB repository [34]. All the Docker images are stored in the Docker hub: https://hub.docker.com/u/repbioinfo.

## Availability of supporting source code and requirements

Project name: rCASC: reproducible Classification Analysis of Single Cell sequencing data

Project home page: https://github.com/kendomaniac/rCASC; https://github.com/mbeccuti/4SeqGUI

Operating system: Linux

Programming language: R and JAVA

Other requirements: None

License: GNU Lesser General Public License, version 3.0 (LGPL-3.0)

RRID:SCR_017005

## Additional files

Supplementary Methods: Details about the implemented methods.

giz105_GIGA-D-18-00522_Original_Submission

giz105_GIGA-D-18-00522_Revision_1

giz105_GIGA-D-18-00522_Revision_2

giz105_GIGA-D-18-00522_Revision_3

giz105_Response_to_Reviewer_Comments_Original_Submission

giz105_Response_to_Reviewer_Comments_Revision_1

giz105_Response_to_Reviewer_Comments_Revision_2

giz105_Reviewer_1_Report_Original_Submission Olivier Poirion, Ph.D. -- 1/22/2019 Reviewed

giz105_Reviewer_1_Report_Revision_1 Olivier Poirion, Ph.D. -- 4/30/2019 Reviewed

giz105_Reviewer_1_Report_Revision_2 Olivier Poirion, Ph.D. -- 7/25/2019 Reviewed

giz105_Reviewer_2_Report_Original_Submission Nils Eling -- 1/31/2019 Reviewed

giz105_Reviewer_2_Report_Revision_1 Nils Eling -- 4/29/2019 Reviewed

giz105_Reviewer_2_Report_Revision_2 Nils Eling -- 7/15/2019 Reviewed

giz105_Supplemental_File

## Abbreviations

ANOVA: analysis of variance; ATAC-seq: Assay for Transposase-Accessible Chromatin using sequencing; CPU: central processing unit; CSS: cell stability score; griph: Graph Inference of Population Heterogeneity; GUI: graphical user interface; PBMC: peripheral blood mononuclear cell; PCA: principal componet analysis; RAM: random access memory; rCASC: reproducible Classification Analysis of Single Cell sequencing data; RNA-seq: RNA sequencing; SATA: Serial Advanced Technology Attachment; scanpy: Single-Cell Analysis in Python; SIMLR: Single-cell Interpretation via Multi-kernel LeaRning; SS: silhouette score; SSD: solid-state drive; t-SNE: T-distributed Stochastic Neighbor Embedding; UMI: unique molecular identifier.

## Authors’ contributions

L.A. and F.C. equally participated to write R scripts, to create the majority of Docker images, to package the workflow and release code. M.B. wrote the Java and C++ code and acted as corresponding author. N.L. implemented scanpy and extended the Java GUI. M.A. and M.O. prepared the single-cell data to be used as examples of the workflow functionality. G.R. prepared the Dockers for fastq to count table conversion. S.R. revised all packages and generated the Docker files for Docker image maintenance and further development. G.D.L. gave scientific advice and provided an unpublished dataset for MAIT resting and activated T-cells (generated with Fluidigm C1 platform) to investigate gene detection limits in 3′-end sequencing technologies and whole-transcript sequencing. R.A.C. and L.P. equally oversaw the project and gave scientific advice. All authors read, contributed to, and approved the final manuscript.
