# Supplementary material for: rCASC: reproducible classification analysis of single-cell sequencing data
Source: Gigascience. 2019 Sep 8;8(9):giz105. doi: 10.1093/gigascience/giz105 (PMC6732171; doi:10.1093/gigascience/giz105)
Supplement: giz105_GIGA-D-18-00522_Original_Submission [file giz105_giga-d-18-00522_original_submission.pdf]

GigaScience

# rCASC: reproducible Classification Analysis of Single Cell sequencing data

--Manuscript Draft--

| | |
|---|---|
| **Manuscript Number:** | GIGA-D-18-00522 |
| **Full Title:** | rCASC: reproducible Classification Analysis of Single Cell sequencing data |
| **Article Type:** | Technical Note |
| **Funding Information:** | |
| **Abstract:** | Background<br><br>Single-cell RNA sequencing is an essential tool to investigate cellular heterogeneity, and to highlight cell sub-population specific signatures. Single-cell sequencing applications are now spreading from the most conventional RNAseq to epigenomics, e.g. ATAC-seq. ATAC-seq. Single-cell sequencing led to the development of a large variety of algorithms and tools. However, to the best of our knowledge, there are few computational workflows providing analysis flexibility and achieving at the same time functional (i.e. information about data and the utilized tools are saved in terms of meta-data) and computational reproducibility (i.e. real image of the computation environment used to generate the data is stored) through a user-friendly environment.<br><br>Findings<br><br>rCASC is a modular workflow providing integrated analysis environment (from counts generation to cell subpopulation identification) exploiting docker containerization to achieve both functional and computational reproducibility in data analysis. Hence, rCASC provides preprocessing tools to remove low quality cells and/or specific bias, e.g. cell cycle. Subpopulations discovery can be instead achieved using unsupervised and supervised clustering techniques. Quality of clusters is then estimated through a new metric namely Cell Stability Score (CSS), which describes the stability of a cell in a cluster as consequence of a perturbation induced by removing a random set of cells from the overall cells population. CSS provides better cluster-robustness information than silhouette metric. Moreover, rCASC provides also tools for the identification of clusters-specific gene-signature.<br><br>Conclusions<br><br>rCASC is a modular workflow with valuable new features that could help researchers in defining cells subpopulations and in detecting subpopulation specific markers. It exploits docker framework to make easier its installation and to achieve a computation reproducible analysis. Moreover, a Java Graphical User Interface (GUI), is also provided in rCASC to make friendly the use of the tool even for users without computational skills in R. |
| **Corresponding Author:** | Marco Beccuti, Ph.D<br>Universita degli Studi di Torino<br>Turin, Piemonte ITALY |
| **Corresponding Author Secondary Information:** | |
| **Corresponding Author's Institution:** | Universita degli Studi di Torino |
| **Corresponding Author's Secondary Institution:** | |
| **First Author:** | Luca Alessandrì, Ph.D student |
| **First Author Secondary Information:** | |
| **Order of Authors:** | Luca Alessandrì, Ph.D student |
| | Francesca Cordero, Ph.D. |
| | Marco Beccuti, Ph.D. |

Powered by Editorial Manager® and ProduXion Manager® from Aries Systems Corporation

| | |
|---|---|
| | Maddalena Arigoni, Ph.D. |
| | Martina Olivero, Ph.D. |
| | Greta Romano |
| | Sergio Rabellino |
| | Gennaro De Libero, Ph.D. |
| | Luigia Pace, Ph.D. |
| | Raffaele Calogero |
| **Order of Authors Secondary Information:** | |
| **Additional Information:** | |
| **Question** | **Response** |
| Are you submitting this manuscript to a special series or article collection? | No |
| **Experimental design and statistics**<br><br>Full details of the experimental design and statistical methods used should be given in the Methods section, as detailed in our Minimum Standards Reporting Checklist. Information essential to interpreting the data presented should be made available in the figure legends.<br><br>Have you included all the information requested in your manuscript? | Yes |
| **Resources**<br><br>A description of all resources used, including antibodies, cell lines, animals and software tools, with enough information to allow them to be uniquely identified, should be included in the Methods section. Authors are strongly encouraged to cite Research Resource Identifiers (RRIDs) for antibodies, model organisms and tools, where possible.<br><br>Have you included the information requested as detailed in our Minimum Standards Reporting Checklist? | Yes |
| **Availability of data and materials** | Yes |

| | |
|---|---|
| All datasets and code on which the conclusions of the paper rely must be either included in your submission or deposited in publicly available repositories (where available and ethically appropriate), referencing such data using a unique identifier in the references and in the “Availability of Data and Materials” section of your manuscript.<br><br>Have you have met the above requirement as detailed in our Minimum Standards Reporting Checklist? | |

Powered by Editorial Manager® and ProduXion Manager® from Aries Systems Corporation

Click here to download Manuscript alessandri_et_al_gigascience_submission_v3.docx

# rCASC: reproducible Classification Analysis of Single Cell sequencing data

[1#]Luca Alessandrì, [2#]Francesca Cordero, [2$]Marco Beccuti, [1]Maddalena Arigoni, [3]Martina Olivero, [2]Greta Romano, [2]Sergio Rabellino, [4]Gennaro De Libero, [5*]Luigia Pace and [1*]Raffaele A Calogero

[1]Department of Molecular Biotechnology and Health Sciences, University of Torino, Via Nizza 52, Torino, Italy

[2]Department of Computer Sciences, University of Torino, Corso Svizzera 185, Torino, Italy

[3]Department of Oncology, University of Torino, SP142, 95, 10060 Candiolo TO, Italy

[4]Department Biomedizin, University of Basel, Hebelstrasse 20, 4031 Basel, Switzerland

[5]IIGM, Via Nizza 52, Torino, Italy

#Both authors equally contributed the present work

*Both authors equally supervised the present work

[$]Corresponding author

Luca Alessandrì alessandri.luca1991@gmail.com

Francesca Cordero fcordero@di.unito.it

Marco Beccuti beccuti@di.unito.it

Maddalena Arigoni maddalena.arigoni@unito.it

Martina Olivero martina.olivero@unito.it

Greta Romano greta.romano@unito.it

Sergio Rabellino sergio.rabellino@unito.it

Gennaro De Libero gennaro.delibero@unibas.ch

Luigia Pace luigia.pace@iigm.it

Raffaele A Calogero raffaele.calogero@unito.it

## Abstract

**Background**

Single-cell RNA sequencing is an essential tool to investigate cellular heterogeneity, and to highlight cell sub-population specific signatures. Single-cell sequencing applications are now spreading from the most conventional RNAseq to epigenomics, e.g. ATAC-seq. ATAC-seq. Single-cell sequencing led to the development of a large variety of algorithms and tools. However, to the best of our knowledge, there are few computational workflows providing analysis flexibility and achieving at the same time functional (i.e. information about data and the utilized tools are saved in terms of meta-data) and computational reproducibility (i.e. real image of the computation environment used to generate the data is stored) through a user-friendly environment.

**Findings**

rCASC is a modular workflow providing integrated analysis environment (from counts generation to cell subpopulation identification) exploiting docker containerization to achieve both functional and computational reproducibility in data analysis. Hence, rCASC provides preprocessing tools to remove low quality cells and/or specific bias, e.g. cell cycle. Subpopulations discovery can be instead achieved using unsupervised and supervised clustering techniques. Quality of clusters is then estimated through a new metric namely Cell Stability Score (CSS), which describes the stability of a cell in a cluster as consequence of a perturbation induced by removing a random set of cells from the overall cells population. CSS provides better cluster-robustness information than silhouette metric. Moreover, rCASC provides also tools for the identification of clusters-specific gene-signature.

**Conclusions**

rCASC is a modular workflow with valuable new features that could help researchers in defining cells subpopulations and in detecting subpopulation specific markers. It exploits docker framework to make easier its installation and to achieve a computation reproducible analysis. Moreover, a Java Graphical User Interface (GUI), is also provided in rCASC to make friendly the use of the tool even for users without computational skills in R.



**Keywords**

Single-cell data preprocessing, workflow, GUI, supervised clustering, unsupervised clustering, cluster stability metrics, cluster-specific gene signature.

# Findings

## rCASC: a single cell analysis workflow designed to provide data reproducibility.

Since the end of the 90's omics high-throughput technologies have generated an enormous amount of data, reaching today an exponential growth phase. The analysis of omics big data is a revolutionary means of understanding the molecular basis of disease regulation and susceptibility, and this resource is made accessible to the biological/medical community via bioinformatics frameworks. However, due to the increasing complexity and fast evolution of computation tools and omics methods, the reproducibility crisis [1] is becoming a very important issue [2] and there is a mandatory need to guarantee robust and reliable results to the research community [3].

Single cell analysis is instrumental to understand the functional differences existing among cells within a tissue. Individual cells of the same phenotype are commonly viewed as identical functional units of a tissue or organ. However, single cells sequencing results [4] suggest the presence of a complex organization of heterogeneous cell states producing together system-level functionalities. A mandatory element of single cell RNAseq is the availability of dedicated bioinformatics workflows. In this context rCASC provides a modular workflow to address at the same time the problem of functional and computational reproducibility. rCASC provides single cell analysis functionalities within the reproducible rules described by Sandve [5]. rCASC is part of the Reproducible Bioinformatics Project [6], which is a project designed to provide to the biological community a reproducible and user-friendly bioinformatics ecosystem [7]. All computational tools in rCASC are embedded in dockers images stored in a public repository on docker hub. Parameters are delivered to docker containers via a set of R functions, part of rCASC R github package [8]. To simplify the use of rCASC package to users without scripting experience, R functions can be controlled by a dedicated GUI integrated of the 4SeqGUI tool previously published by us [7], which is also available as github package [9]. rCASC is specifically designed to provide an integrated analysis environment for cell-subpopulation discovery. The workflow allows the direct analysis of fastq files, generated with 10X Genomics and inDrop platforms, or count matrices. Therefore, rCASC provides raw data preprocessing, subpopulation discovery via supervised/unsupervised clustering and cluster-specific genes-signatures detection. The key elements of rCASC workflow are shown in Figure 1, and the main functionalities are summarized in Methods section. A detailed description of the rCASC functions is also available in the vignettes section of rCASC github [8].

The overall characteristics of rCASC were compared with other four workflows for single-cells analysis (Figure 2): i) simpleSingleCell, Bioconductor workflow package [10]; ii) Granatum, web-

based scRNA-Seq analysis suite [11]; iii) SCell, graphical workflow for single-cell analysis [12]; iv) R toolkit Seurat [13]. The comparison was based on the following elements: a) supported single-cell platforms, b) types of tools provided by the workflow, c) type of reproducibility granted by the workflow, d) usage flexibility.

rCASC is the only workflow providing support at fastq level because all the others packages require as input the processed counts table. Cell quality control and outliers' identification is available in all workflow but Granatum. Association of ENSEMBL gene IDs to gene symbols is only provided by rCASC. All workflows provide genes filtering tools but simpleSingleCell. All packages provide normalization procedures to be applied to raw counts data. However, rCASC is the only tool providing both Seurat specific normalization [13] and count-depth specific normalization [14]. The workflows implement different data reduction and clustering methods. rCASC implements Seurat [13] as unsupervised clustering tool and SIMLR [15] as supervised clustering tools. Notably, Freytag [16] recently published a comparison of single-cell clustering methods, in which SIMLR and Seurat were included. Freytag showed that the two methods performed better than other clustering methods and they behaved in similar way on Freytag's golden standard dataset. rCASC is the only workflow performing clustering in presence of data perturbation, i.e. removal of a subset of cells, and measuring cluster quality using Cell Stability Score (CSS is a cluster quality metrics developed by us, which measures the persistence of each cell in a cluster upon data perturbation, see Supplementary file section 5.1) and Silhouette score (SS is a cluster quality metrics measuring the consistency within clusters of data). In our experiments CSS provides a better estimation of the cluster stability with respect to what can be depicted using SS (Figure 2). Gene feature selection approaches are implemented in different way in the five workflows. Granatum is the only one providing biological inference. Granatum and Seurat implements various statistical methods to detect cluster specific genes signatures (Figure 3). rCASC embeds an ANOVA-like statistics derived from EdgeR Bioconductor package [17] and Seurat/SIMLR genes prioritization procedures (see Supplementary file section 7). Visualization of genes-signatures by heatmap of by coloring cell on the basis of gene expression is only provided by rCASC (see Supplementary file Figure 51). Considering reproducibility, only rCASC provides both computational and functional reproducibility. Finally, rCASC is the only one providing both command line and GUI (Figure 4).

rCASC was used to re-analyze the single-cell dataset from Pace paper [18]. In this paper, authors highlighted that Suv39h1-defective CD8$^+$ T-cells show sustained survival and increased long-term memory reprogramming capacity. Our re-analysis extends the information described in Pace paper,

suggesting the presence of an enriched Suv39h1-defective memory subset. A complete description of the above analysis is available at section 8 of supplementary file.

# Methods

## Counts table generation

inDrop single-cell sequencing approach was originally published by Klein [19]. Then, the authors published the detailed protocol in Nature Methods in 2017 [20]. In rCASC, the generation of the count table starting from fastq files refers to the version 2 of the inDrop chemistry described in [20], which is commercially distributed by 1CellBio. The procedure described in the inDrop github [21] in embedded in a docker image. rCASC function *indropIndex* allows the generation of the transcripts index required to convert fastq to counts and *indropCounts* function converts reads in UMI counts.
10XGenomics Cellranger is packed in a docker image and the function *cellrangerCount* converts fastq to UMI matrix using any of the genome indexes available at 10XGenomics data repository. Detailed description about the counts table generation is available in Supplementary file section 2.

## Counts table exploration and manipulation

rCASC provides various data inspection and preprocessing tools.
*genesUmi* function generates a plot where the number of detected genes are plotted for each cell with respect to the number of UMI/reads quantified for each cell (Figure 5A,C).
*mitoRiboUmi* calculates the percentage of mitochondrial/ribosomal genes with respect to the total number of detected genes in each cell and plots percentage of mitochondrial genes with respect to percentage of ribosomal genes. Furthermore, cells are colored on the basis of the number of detected genes (Figure 5B,D). *mitoRiboUmi* allows to identify cells with low information content, i.e. those cells with a little number of detectable genes, e.g. < 100 genes/cell, little ribosomal content and high content of mitochondrial genes, which indicate cell stress [22].
The function *scannobyGtf* uses ENSEMBL gtf and the R package refGenome to associate gene symbol with the ENSEMBL gene ID. Furthermore, *scannobyGtf* allows the removal of mitochondrial/ribosomal genes (Figure 5A,C) and the removal of "stressed" cells detectable with *mitoRiboUmi* function (Figure 5B,D).
The function *lorenzFilter* embeds the Lorenz statistics developed by Diaz [12], a cell quality statistics correlated with cell live-dead staining (see Supplementary file sections 3.3).
As counts table preprocessing steps, we implemented the functions *checkCountDepth/scnorm* to detect the presence of sample specific count–depth relationship [14] (i.e. the relationship existing between transcript-specific expression and sequencing depth) and adjust the counts table for it. Furthermore, we have added two other functions *recatPrediction* and *ccRemove*, which are based

respectively on the paper of Liu [23] and Barron [24]. The function *recatPrediction* organizes the single cell data to reconstruct cell cycle pseudo time-series and it is used to understand if a cell cycle effect is present. Then, *ccRemove* function is used to mitigate the cell cycle effect of the inter-samples transcriptome when it is detected by *recatPrediction* function (see Supplementary file sections 3.6 ad 3.8).

### Clustering

For the identification of cell subpopulations we implemented two clustering approaches: Seurat [13] and SIMLR [15]. Seurat is a hierarchical method and in rCASC is controlled by the functions *seuratPCAEval* and *seuratBootstrap*. The function *seuratPCAEval* is used to identify the subset of PCA components to be used for clustering and *seuratBootstrap* implements the clustering. Differently SIMLR implements a k-mean clustering, where the number of clusters (i.e. k) is taken as input. The function *simlrBootstrap* controls the clustering procedure and the function *nClusterEvaluationSIMLR*, a wrapper for the R package griph (Graph Inference of Population Heterogeneity) [25], is exploited to estimate the (sub)optimal number "k" of clusters. We developed, for both Seurat and SIMLR, a procedure to measure the cluster quality on the basis of data structure. The rationale of our approach is that cells belonging to a specific cluster should be little affected by changes in the numerosity of the dataset, e.g. removal of 10% of the total number of cells used for clustering. Thus, we developed a metrics called CSS (Cell Stability Score), which describes the persistence of a cell in a specific cluster upon jackknifing and therefore offers a peculiar way of describing cluster stability. Detailed description of CSS metrics is available in Supplementary file at section 5.1. CSS is embedded in *seuratBootstrap* and *simlrBootstrap*, and it is also used in *nClusterEvaluationSIMLR* to identify which number of clusters gives the best CSS behavior.

### Feature selection

To select the most important features of each cluster we implemented in the *anovaLike* function the edgeR ANOVA-like method for single cells [17] and in the functions *seuratPrior* and *genesPrioritization/genesSelection* respectively the Seurat and SIMLR genes prioritization methods. *hfc* function allows the visualization of the genes prioritized with the above methods as heatmap and provides plots of prioritized genes in each single cell (Figure 6).

## Availability and requirements

Project name: rCASC: reproducible Classification Analysis of Single Cell sequencing data

Project home page: https://github.com/kendomaniac/rCASC; https://github.com/mbeccuti/4SeqGUI

Operating system: Linux

Programming language: R and JAVA

Other Requirements: None

License: The GNU Lesser General Public License, version 3.0 (LGPL-3.0)

Any restrictions to use by non-academics: None

## Authors' contributions

LA and FC equally participated to write R scripts, to create the majority of docker images, to package the workflow and release code. MB wrote the GUI and acted as corresponding author. MA and MO prepared the single-cell data to be used as examples of the workflow functionality. GR prepared the dockers for fastq to counts table conversion. SR revised all packages and generated the docker files for docker images maintenance and further development. GDL gave scientific advices and provided an unpublished dataset for MAIT resting and activated T-cells (generated with Fluidigm C1 platform) to investigate genes detection limits in 3'end sequencing technologies and whole transcript sequencing. RAC and LP equally oversaw the project and gave scientific advices. All authors read, contributed and approved the final manuscript.

## Supplementary material

rCASC_supplementary_file.pdf

## References


1. Allison DB, Shiffrin RM, Stodden V: **Reproducibility of research: Issues and proposed remedies**. *Proceedings of the National Academy of Sciences of the United States of America* 2018, **115**(11):2561-2562.
2. **Challenges in irreproducible research** [https://www.nature.com/collections/prbfkwmwvz]
3. **Reproducibility in Computational Biology** [http://www.global-engage.com/life-science/reproducibility-computational-biology/]
4. Buettner F, Natarajan KN, Casale FP, Proserpio V, Scialdone A, Theis FJ, Teichmann SA, Marioni JC, Stegle O: **Computational analysis of cell-to-cell heterogeneity in single-cell RNA-sequencing data reveals hidden subpopulations of cells**. *Nature biotechnology* 2015, **33**(2):155-160.
5. Sandve GK, Nekrutenko A, Taylor J, Hovig E: **Ten simple rules for reproducible computational research**. *PLoS computational biology* 2013, **9**(10):e1003285.
6. Kulkarni N, Alessandri L, Panero R, Arigoni M, Olivero M, Ferrero G, Cordero F, Beccuti M, Calogero RA: **Reproducible bioinformatics project: a community for reproducible bioinformatics analysis pipelines**. *BMC bioinformatics* 2018, **19**(Suppl 10):349.
7. Beccuti M, Cordero F, Arigoni M, Panero R, Amparore EG, Donatelli S, Calogero RA: **SeqBox: RNAseq/ChIPseq reproducible analysis on a consumer game computer**. *Bioinformatics* 2018, **34**(5):871-872.
8. **rCASC R Package** [https://github.com/kendomaniac/rCASC]
9. **4SeqGUI** [https://github.com/mbeccuti/4SeqGUI]

10. Lun AT, McCarthy DJ, Marioni JC: **A step-by-step workflow for low-level analysis of single-cell RNA-seq data with Bioconductor**. *F1000Res* 2016, **5**:2122.
11. Zhu X, Wolfgruber TK, Tasato A, Arisdakessian C, Garmire DG, Garmire LX: **Granatum: a graphical single-cell RNA-Seq analysis pipeline for genomics scientists**. *Genome medicine* 2017, **9**(1):108.
12. Diaz A, Liu SJ, Sandoval C, Pollen A, Nowakowski TJ, Lim DA, Kriegstein A: **SCell: integrated analysis of single-cell RNA-seq data**. *Bioinformatics* 2016, **32**(14):2219-2220.
13. Butler A, Hoffman P, Smibert P, Papalexi E, Satija R: **Integrating single-cell transcriptomic data across different conditions, technologies, and species**. *Nature biotechnology* 2018, **36**(5):411-420.
14. Bacher R, Chu LF, Leng N, Gasch AP, Thomson JA, Stewart RM, Newton M, Kendziorski C: **SCnorm: robust normalization of single-cell RNA-seq data**. *Nature methods* 2017, **14**(6):584-586.
15. Wang B, Zhu J, Pierson E, Ramazzotti D, Batzoglou S: **Visualization and analysis of single-cell RNA-seq data by kernel-based similarity learning**. *Nature methods* 2017, **14**(4):414-416.
16. Freytag S, Tian L, Lonnstedt I, Ng M, Bahlo M: **Comparison of clustering tools in R for medium-sized 10x Genomics single-cell RNA-sequencing data**. *F1000Res* 2018, **7**:1297.
17. Robinson MD, McCarthy DJ, Smyth GK: **edgeR: a Bioconductor package for differential expression analysis of digital gene expression data**. *Bioinformatics* 2010, **26**(1):139-140.
18. Pace L, Goudot C, Zueva E, Gueguen P, Burgdorf N, Waterfall JJ, Quivy J-P, Almouzni G, Amigorena S: **The epigenetic control of stemness in CD8+ T cell fate commitment**. *Science (New York, N Y )* 2018, **359**(6372):177-186.
19. Klein AM, Mazutis L, Akartuna I, Tallapragada N, Veres A, Li V, Peshkin L, Weitz DA, Kirschner MW: **Droplet barcoding for single-cell transcriptomics applied to embryonic stem cells**. *Cell* 2015, **161**(5):1187-1201.
20. Zilionis R, Nainys J, Veres A, Savova V, Zemmour D, Klein AM, Mazutis L: **Single-cell barcoding and sequencing using droplet microfluidics**. *Nature protocols* 2017, **12**(1):44-73.
21. **indrops** [https://github.com/indrops/indrops]
22. AlJanahi AA, Danielsen M, Dunbar CE: **An Introduction to the Analysis of Single-Cell RNA-Sequencing Data**. *Mol Ther Methods Clin Dev* 2018, **10**:189-196.
23. Liu ZH, Lou HZ, Xie KK, Wang H, Chen N, Aparicio OM, Zhang MQ, Jiang R, Chen T: **Reconstructing cell cycle pseudo time-series via single-cell transcriptome data**. *Nature Communications* 2017, **8**.
24. Barron M, Li J: **Identifying and removing the cell-cycle effect from single-cell RNA-Sequencing data**. *Sci Rep* 2016, **6**:33892.
25. **griph: Graph Inference of Population Heterogeneity** [https://github.com/ppapasaikas/griph]
26. Chhangawala S, Rudy G, Mason CE, Rosenfeld JA: **The impact of read length on quantification of differentially expressed genes and splice junction detection**. *Genome biology* 2015, **16**:131.
27. Turman MA, Yabe T, McSherry C, Bach FH, Houchins JP: **Characterization of a novel gene (NKG7) on human chromosome 19 that is expressed in natural killer cells and T cells**. *Hum Immunol* 1993, **36**(1):34-40.

## Figure legend

**Figure 1:** rCASC workflow. Blue boxes indicate preprocessing tools. Yellow boxes define clustering tools. Green box indicates genes-signatures tools.

**Figure 2:** Cell Stability Score versus Silhouette Score calculated on Pace's dataset (see Supplementary file section 8) using SIMLAR over a set of number of clusters ranging between 5 and 8. A) Cell Stability Score violin plot. Looking at the mean value and data dispersion the best number of clusters is 5, indicating the with 5 clusters cells remain in the same cluster more about 80% of the times a random removal of 10% of the cell is applied to the full dataset. B) Silhouette Score violin plot Looking at the mean value of the SS distribution there are no clear evidences that one clusterization is better that another. Furthermore, the dispersion of the SS value is getting narrow as the number of the clusters increases.

**Figure 3:** Comparison between the analysis features available in rCASC and in other single-cell analysis workflows.

**Figure 4:** rCASC graphical interface within 4seqGUI. A) Counts table generation menu: this set of function is devoted to the conversion of fastq to a counts table. B) Counts table manipulation menu: this set of functions allows inspection, filtering and normalization of the counts table. C) Clustering menu: these functions allow the use of SIMLR, tSne and Seurat to group cells in subpopulations. D) Feature selection menu: this set of functions allow the identification of cluster-specific subsets of genes and their visualization using heatmaps.

**Figure 5:** *genesUmi* plots the number of detectable genes in each cell (a cell is called present if supported by a user defined N number of UMI/reads, suggested values N=3 for UMI or N=5 for smart-seq sequencing [26]) with respect to the number of sequences UMI/reads. *mitoRiboUmi* calculates the percentage of mitochondrial/ribosomal genes with respect to the total number of detected genes in each cell and plots % of mitochondrial genes with respect to % of ribosomal genes. Furthermore, cells are colored on the basis of the number of detected genes: A) *genesUmi* plot for resting CD8+ T-cells [18], sequencing average 83,000 reads/cell. B) *mitoRiboUmi* plot for resting CD8+ T-cells [18]. It is notable that cells aggregated in two groups: the majority of the cells with less than 100 detected genes groups together and they are characterized by high relative percentage of mitochondrial genes and low relative percentage of ribosomal genes. Remaining cells are

characterized by few detectable genes, 100÷250 genes/cell, with a percentage of ribosomal genes greater than 30%. C) *genesUmi* plot for Listeria activated CD8+ T-cells [18], sequencing average 83,000 reads/cell, it is notable the activated cells show a wider range of detectable genes. D) *mitoRiboUmi* plot for Listeria activated CD8+ T-cells [18]. The majority of the cells are characterized by more the 100 genes called present and they show low percentage of mitochondrial genes and percentage of ribosomal genes between 15 to 35%. The remaining cells, with less than 100 detected genes groups together and are characterized by high relative percentage of mitochondrial genes and low relative percentage of ribosomal genes.

**Figure 6:** Heat map and cell expression plot for prioritized genes. A) Heat map for the set of 577 genes selected for Pace datasets (see Supplementary file section 8) by SIMLR prioritization. B) Nkg7 CPM expression in the cell clusters. Nkg7 is expressed in activated T-cells (clusters 1, 2, 4, 5) [27] but not in resting T-cells (cluster 3).

Figure 1

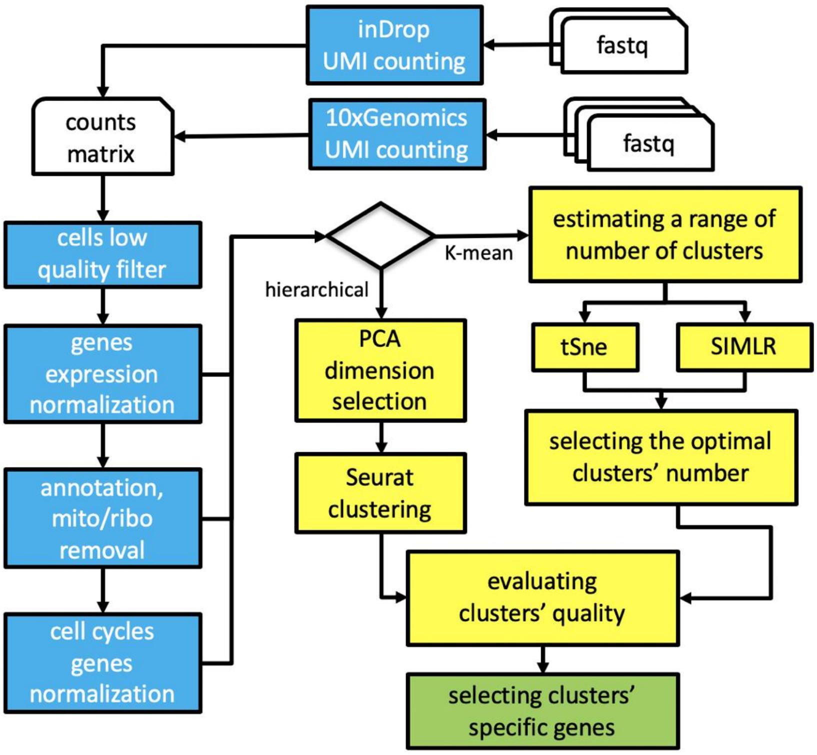

inDrop UMI counting
fastq
counts matrix
10xGenomics UMI counting
fastq
cells low quality filter
genes expression normalization
annotation, mito/ribo removal
cell cycles genes normalization
hierarchical
K-mean
estimating a range of number of clusters
PCA dimension selection
tSne
SIMLR
selecting the optimal clusters' number
Seurat clustering
evaluating clusters' quality
selecting clusters' specific genes

Figure 2

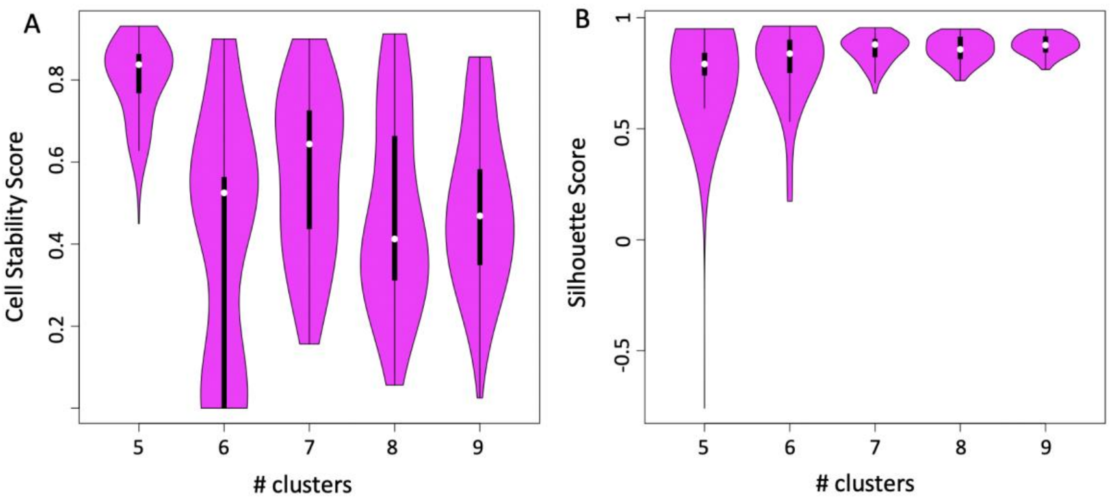
A
Cell Stability Score
0.2
0.4
0.6
0.8
5
6
7
8
9
# clusters
B
Silhouette Score
-0.5
0
0.5
1
5
6
7
8
9
# clusters

Figure 3

| | | rCASC (stand alone) | simpleSingleCell (stand alone) | Granatum (web) | Scell (stand alone) | Seurat (stand alone) |
|---|---|---|---|---|---|---|
| **Platforms** | | 10Xgenomics, inDrop, counts table | counts table | counts table | counts table | counts table |
| **Tools** | *Fastq conversion in counts table* | Y | - | - | - | - |
| | *Quality Control / Outlier Filtering* | Y | - | Y | Y | Y |
| | *Annotation* | ENSEMBL ID -> Gene Symbol | - | - | - | - |
| | *Genes filter* | Y | - | Y | Y | Y |
| | *Data normalization* | Y | Y | Y | Y | Y |
| | *Cell cycle bias removal* | Y | - | - | Y | Y |
| | *Data dimensionality reduction* | Y | Y | Y | Y | Y |
| | *Supported clustering methods* | tSne, SIMLR, Seurat PCA | Walktrap | Non-negative matrix factorization, K-mean (Euclidean), K-mean (tSne) | PCA, k-means, Gaussian mixture, Minkowski weighted k-means, DBSCAN | PCA, tSne, ica, dmap |
| | *Cluster quality score* | Silhuette, Cell Stability Score | - | - | - | - |
| | *Features selection and visualization* | Y | Y | Y | - | - |
| | *Supported methods* | ANOVA-like (edgeR), SIMLR Seurat genes prioritization | filtering on expression | NODES, SCDE, EdgeR, Limma | - | wilcox, bimod, roc, t-test, tobit, negbinom, MAST, DESeq2 |
| | *Biological inference* | - | - | Y | - | - |
| **Reproducibility** | *Functional reproducibility* | Y | Y | - | - | Y |
| | *Computational reproducibility* | Y | - | Y | Y | - |
| **Flexibility** | *line command execution* | Y | Y | - | - | Y |
| | *graphical interface* | Y | - | Y | Y | - |

Figure 4

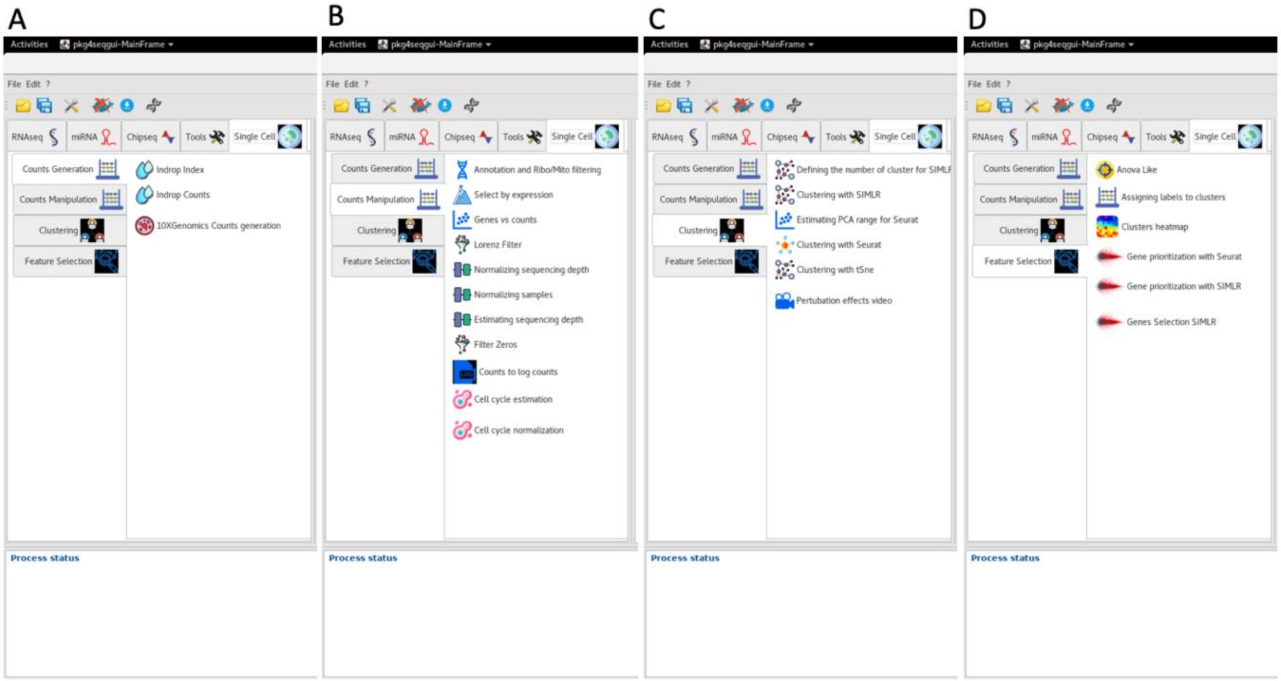
A
B
C
D
Activities
pkg4seqgui-MainFrame
File Edit ?
RNAseq
miRNA
Chipseq
Tools
Single Cell
Counts Generation
Counts Manipulation
Clustering
Feature Selection
Indrop Index
Indrop Counts
10XGenomics Counts generation
Annotation and Ribo/Mito filtering
Select by expression
Genes vs counts
Lorenz Filter
Normalizing sequencing depth
Normalizing samples
Estimating sequencing depth
Filter Zeros
Counts to log counts
Cell cycle estimation
Cell cycle normalization
Defining the number of cluster for SIMLR
Clustering with SIMLR
Estimating PCA range for Seurat
Clustering with Seurat
Clustering with tSne
Pertubation effects video
Anova Like
Assigning labels to clusters
Clusters heatmap
Gene prioritization with Seurat
Gene prioritization with SIMLR
Genes Selection SIMLR
Process status

Figure 5

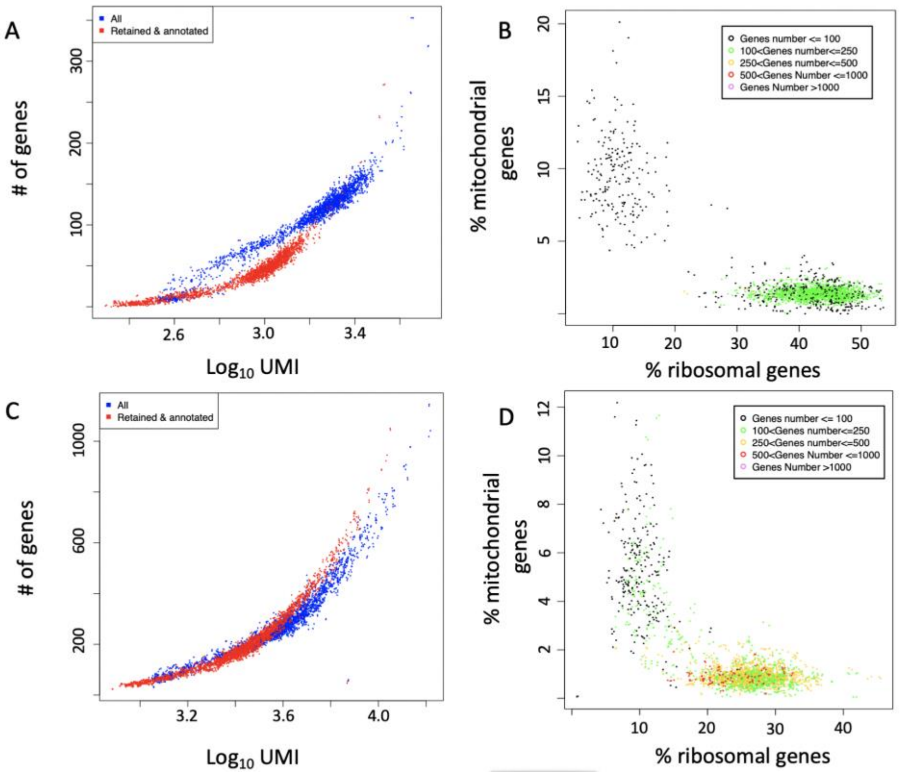

A
All
Retained & annotated
# of genes
100
200
300
2.6
3.0
3.4
Log10 UMI
B
Genes number <= 100
100<Genes number<=250
250<Genes number<=500
500<Genes Number <=1000
Genes Number >1000
% mitochondrial genes
5
10
15
20
10
20
30
40
50
% ribosomal genes
C
All
Retained & annotated
# of genes
200
600
1000
3.2
3.6
4.0
Log10 UMI
D
Genes number <= 100
100<Genes number<=250
250<Genes number<=500
500<Genes Number <=1000
Genes Number >1000
% mitochondrial genes
2
4
6
8
10
12
10
20
30
40
% ribosomal genes

Figure 6

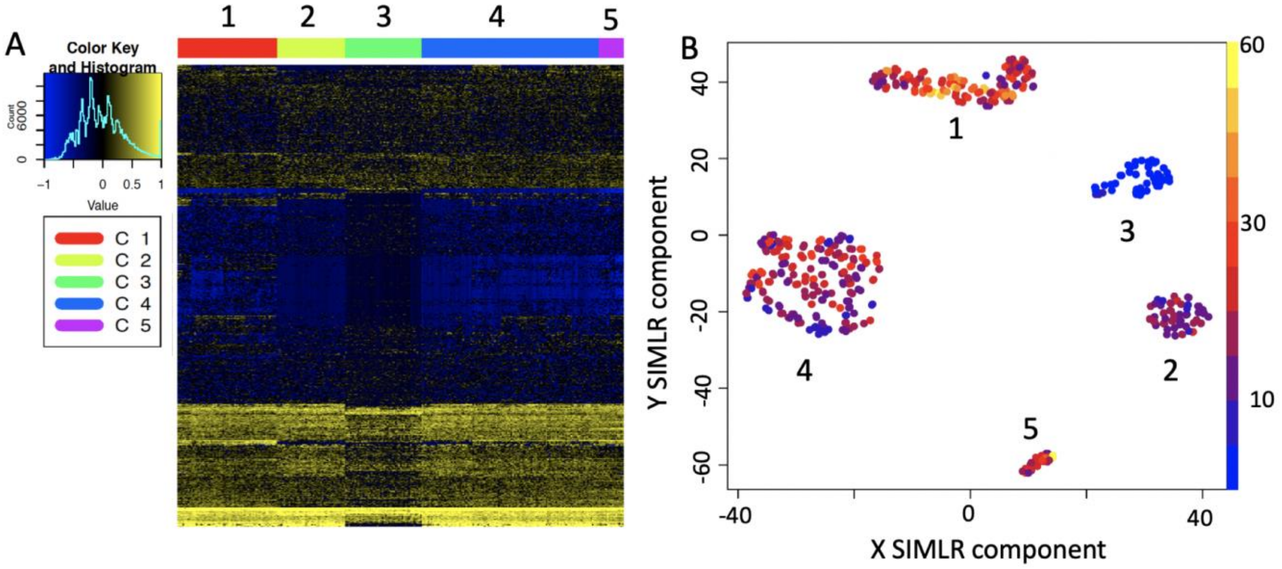
A
Color Key and Histogram
Count
Value
C 1
C 2
C 3
C 4
C 5
1
2
3
4
5
B
Y SIMLR component
X SIMLR component
60
30
10

 Click here to download Figure AllFigure.pdf

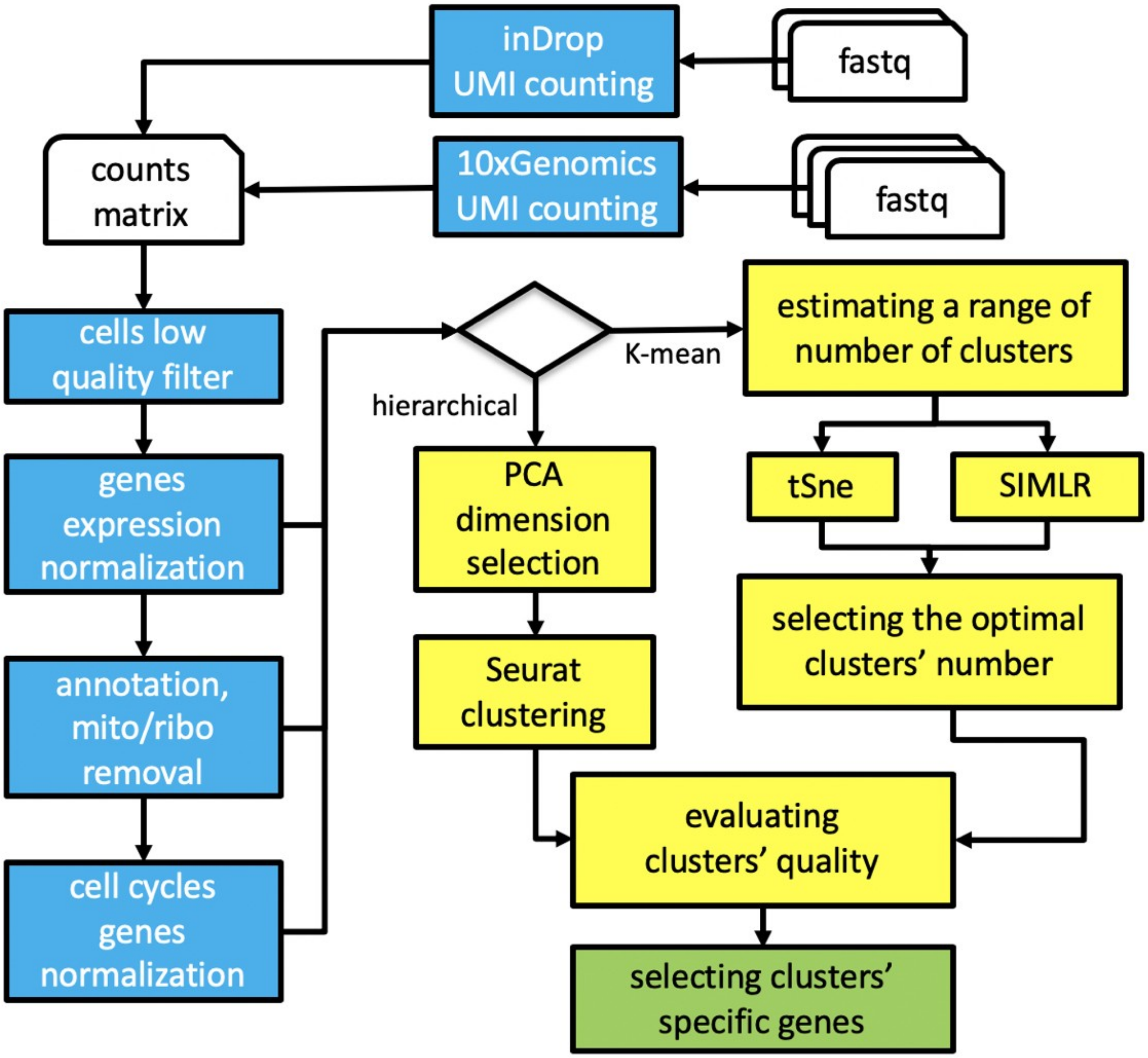


*Figure 1: rCASC workflow. Blue boxes indicate preprocessing tools. Yellow boxes define clustering tools. Green box indicates genes-signatures tools.*

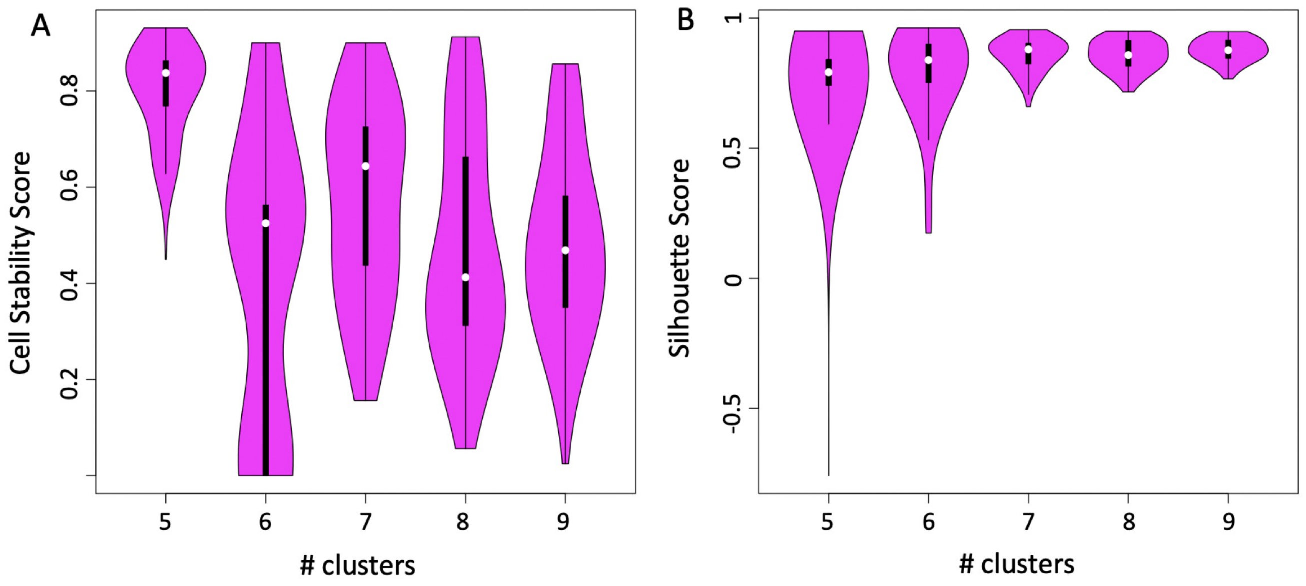


*Figure 2: Cell Stability Score versus Silhouette Score calculated on Pace's dataset (see Supplementary file section 8) using SIMLAR over a set of number of clusters ranging between 5 and 8. A) Cell Stability Score violin plot. Looking at the mean value and data dispersion the best number of clusters is 5, indicating the with 5 clusters cells remain in the same cluster more about 80% of the times a random removal of 10% of the cell is applied to the full dataset. B) Silhouette Score violin plot Looking at the mean value of the SS distribution there are no clear evidences that one clusterization is better that another. Furthermore, the dispersion of the SS value is getting narrow as the number of the clusters increases.*

| | | rCASC (stand alone) | simpleSingleCell (stand alone) | Granatum (web) | Scell (stand alone) | Seurat (stand alone) |
|---|---|---|---|---|---|---|
| **Platforms** | | 10Xgenomics, inDrop, counts table | counts table | counts table | counts table | counts table |
| **Tools** | *Fastq conversion in counts table* | Y | - | - | - | - |
| | *Quality Control / Outlier Filtering* | Y | - | Y | Y | Y |
| | *Annotation* | ENSEMBL ID -> Gene Symbol | - | - | - | - |
| | *Genes filter* | Y | - | Y | Y | Y |
| | *Data normalization* | Y | Y | Y | Y | Y |
| | *Cell cycle bias removal* | Y | - | - | Y | Y |
| | *Data dimensionality reduction* | Y | Y | Y | Y | Y |
| | *Supported clustering methods* | tSne, SIMLR, Seurat PCA | Walktrap | Non-negative matrix factorization, K-mean (Euclidean), K-mean (tSne) | PCA, k-means, Gaussian mixture, Minkowski weighted k-means, DBSCAN | PCA, tSne, ica, dmap |
| | *Cluster quality score* | Silhuette, Cell Stability Score | - | - | - | - |
| | *Features selection and visualization* | Y | Y | Y | - | - |
| | *Supported methods* | ANOVA-like (edgeR), SIMLR Seurat genes prioritization | filtering on expression | NODES, SCDE, EdgeR, Limma | - | wilcox, bimod, roc, t-test, tobit, negbinom, MAST, DESeq2 |
| | *Biological inference* | - | - | Y | - | - |
| **Reproducibility** | *Functional reproducibility* | Y | Y | - | - | Y |
| | *Computational reproducibility* | Y | - | Y | Y | - |
| **Flexibility** | *line command execution* | Y | Y | - | - | Y |
| | *graphical interface* | Y | - | Y | Y | - |

*Figure 3: Comparison between the analysis features available in rCASC and in other single-cell analysis workflows.*

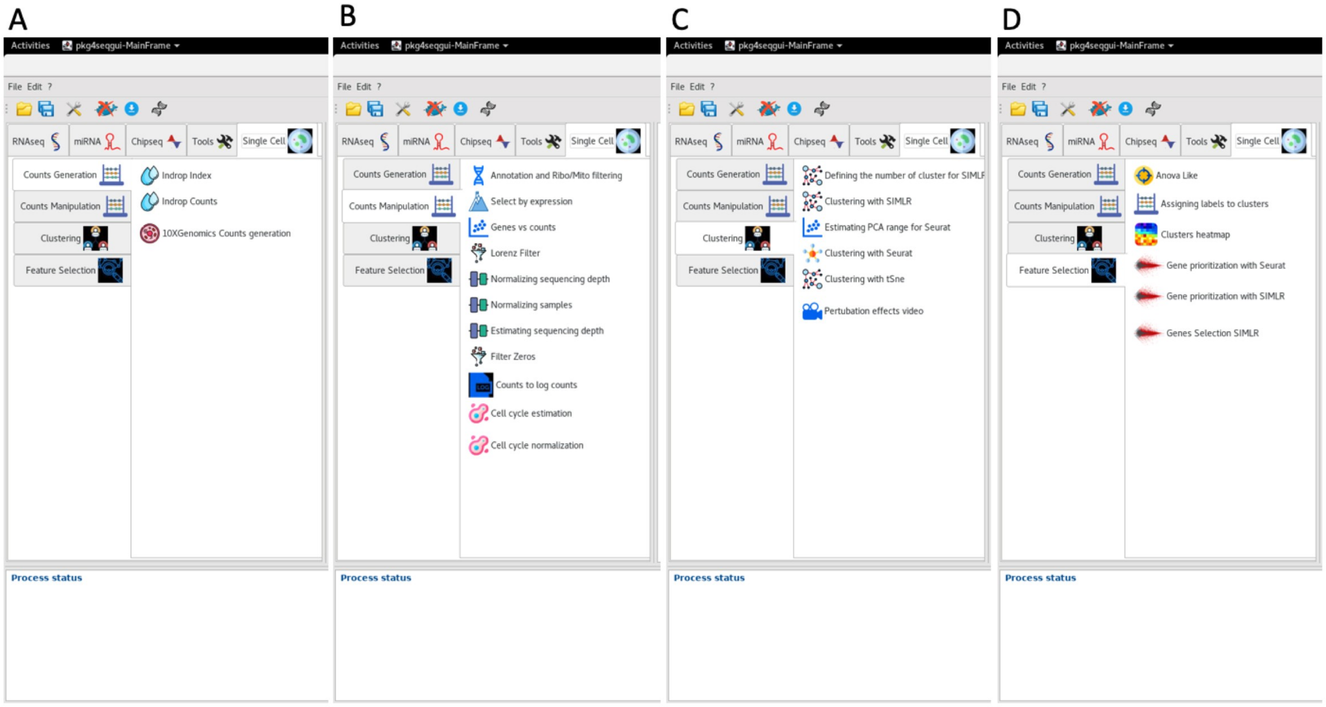


*Figure 4: rCASC graphical interface within 4seqGUI. A) Counts table generation menu: this set of function is devoted to the conversion of fastq to a counts table. B) Counts table manipulation menu: this set of functions allows inspection, filtering and normalization of the counts table. C) Clustering menu: these functions allow the use of SIMLR, tSne and Seurat to group cells in subpopulations. D) Feature selection menu: this set of functions allow the identification of cluster-specific subsets of genes and their visualization using heatmaps.*

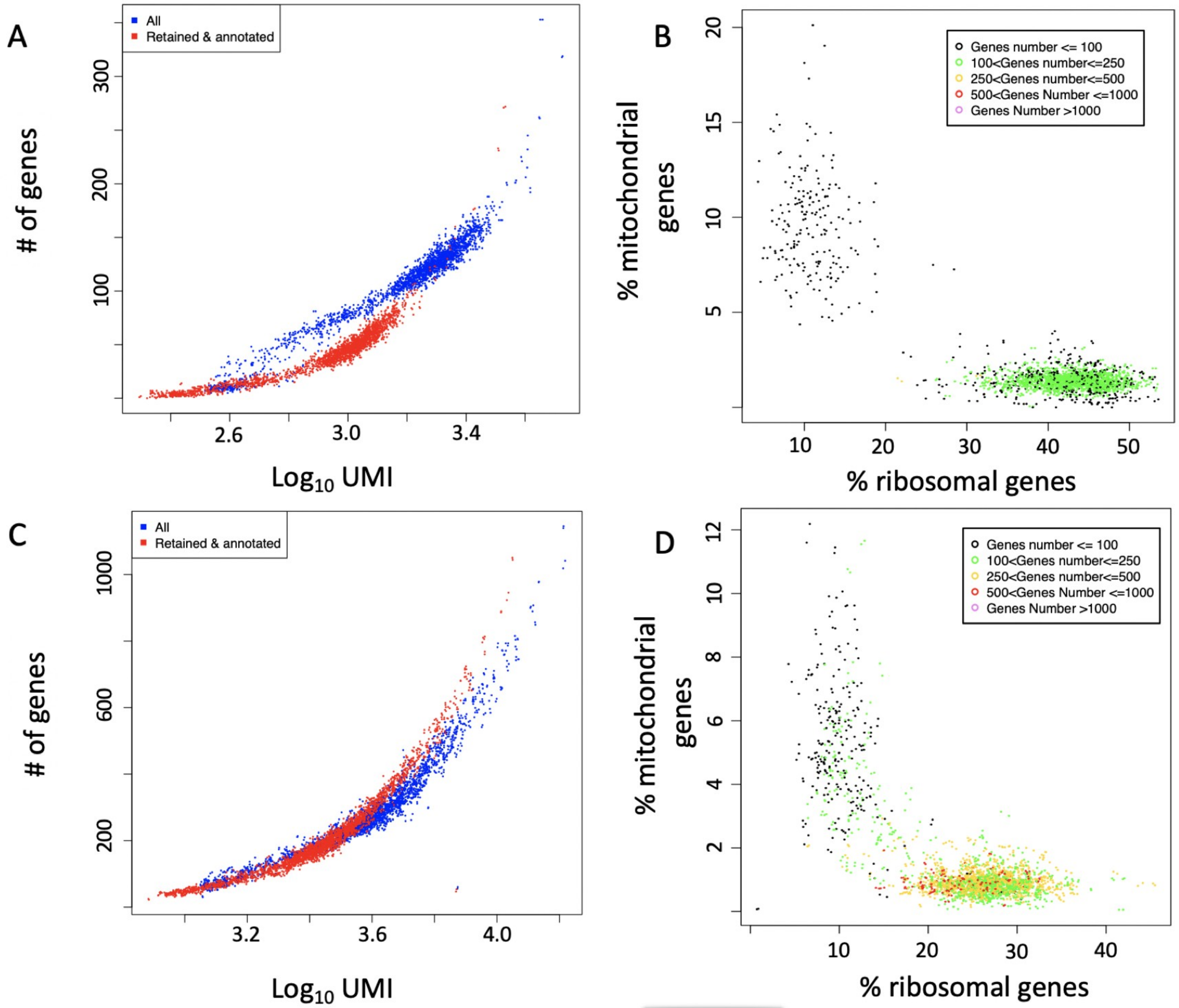


*Figure 5: genesUmi plots the number of detectable genes in each cell (a cell is called present if supported by a user defined N number of UMI/reads, suggested values N=3 for UMI or N=5 for smart-seq sequencing [26]) with respect to the number of sequences UMI/reads. mitoRiboUmi calculates the percentage of mitochondrial/ribosomal genes with respect to the total number of detected genes in each cell and plots % of mitochondrial genes with respect to % of ribosomal genes. Furthermore, cells are colored on the basis of the number of detected genes: A) genesUmi plot for resting CD8+ T-cells [18], sequencing average 83,000 reads/cell. B) mitoRiboUmi plot for resting CD8+ T-cells [18]. It is notable that cells aggregated in two groups: the majority of the cells with less than 100 detected genes groups together and they are characterized by high relative percentage of mitochondrial genes and low relative percentage of ribosomal genes. Remaining cells are characterized by few detectable genes, 100[illegible]250 genes/cell, with a percentage of ribosomal genes greater than 30%. C) genesUmi plot for Listeria activated CD8+ T-cells [18], sequencing average 83,000 reads/cell, it is notable the activated cells show a wider range of detectable genes. D) mitoRiboUmi plot for Listeria activated CD8+ T-cells [18]. The majority of the cells are characterized by more the 100 genes called present and they show low percentage of mitochondrial genes and percentage of ribosomal genes between 15 to 35%. The remaining cells, with less than 100 detected genes groups together and are characterized by high relative percentage of mitochondrial genes and low relative percentage of ribosomal genes.*

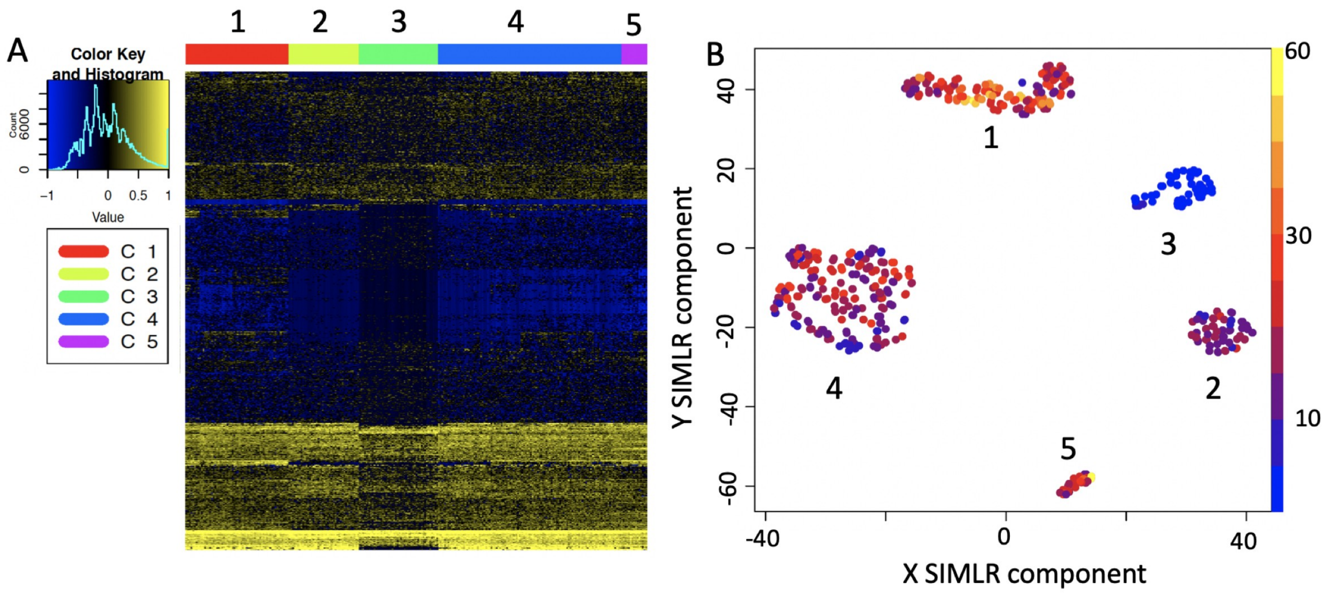


*Figure 6: Heat map and cell expression plot for prioritized genes. A) Heat map for the set of 577 genes selected for Pace datasets (see Supplementary file section 8) by SIMLR prioritization. B) Nkg7 CPM expression in the cell clusters. Nkg7 is expressed in activated T-cells (clusters 1, 2, 4, 5) [27] but not in resting T-cells (cluster 3).*

Manuscript

Click here to access/download
**Supplementary Material**
rCASC_supplementary_file.pdf

Manuscript

Click here to download Personal Cover cover_letter_rCASCmb.docx

Torino 18 December 2018

Dear Editor,

We would like to submit as technical note the attached manuscript entitled “rCASC: reproducible Classification Analysis of Single Cell sequencing data”.

Single-cell RNA sequencing i as an essential tool to investigate cellular heterogeneity, and to highlight cell sub-population specific signatures. Single-cell sequencing applications are now spreading from the most conventional RNAseq to epigenomics, e.g. ATAC-seq. Single-cell sequencing opened new areas of software development, producing an enormous amount of new software. However, only a very small number of them is able to guarantee functional (i.e. the information about data and the utilized tools are saved in terms of meta-data) and/or computational (i.e. the real image of the computation environment used to generate the data is stored) reproducibility.

Being the founders of the bioinformatics community called reproducible-bioinformatics.org (http://www.reproducible-bioinformatics.org/, Kulkarni et al. BMC Bioinformatics, 2018, 19 (Suppl 10):349), whose aim is to provide to the biological community a reproducible bioinformatics ecosystem (Beccuti et al. Bioinformatics, 2018 34 (5), 871–872), then we are very committed to the development of flexible and robust bioinformatics instruments granting reproducibility. Thus, we developed rCASC, which is a modular single-cell RNAseq analysis workflow providing data analysis tools from counts generation to cell sub-population signatures identification, and exploiting docker containerization to achieve computational reproducibility in the data analysis.

Best regards

Raffaele A. Calogero

Bioinformatics and Genomics Unit, Dept. Of Molecular Biotechnology and Health Sciences, University of Torino, Via Nizza 52, Torino (TO), 10126-I, Phone +39 011 670 6454, Fax +39 011 2366454, e-mail raffaele.calogero@unito.it
