# Supplementary material for: rCASC: reproducible classification analysis of single-cell sequencing data
Source: Gigascience. 2019 Sep 8;8(9):giz105. doi: 10.1093/gigascience/giz105 (PMC6732171; doi:10.1093/gigascience/giz105)
Supplement: giz105_GIGA-D-18-00522_Revision_1 [file giz105_giga-d-18-00522_revision_1.pdf]

GigaScience

rCASC: reproducible Classification Analysis of Single Cell sequencing data

--Manuscript Draft--

**Manuscript Number:**	GIGA-D-18-00522R1
**Full Title:**	rCASC: reproducible Classification Analysis of Single Cell sequencing data
**Article Type:**	Technical Note
**Funding Information:**	
**Abstract:**	Background<br>Single-cell RNA sequencing is an essential tool to investigate cellular heterogeneity, and to highlight<br>cell sub-population specific signatures. Single-cell sequencing applications are now spreading from<br>the most conventional RNAseq to epigenomics, e.g. ATAC-seq. Single-cell sequencing led to the<br>development of a large variety of algorithms and tools. However, to the best of our knowledge, there<br>are few computational workflows providing analysis flexibility and achieving at the same time<br>functional (i.e. information about data and the utilized tools are saved in terms of meta-data) and<br>computational reproducibility (i.e. real image of the computation environment used to generate the<br>data is stored) through a user-friendly environment.<br>Findings<br>rCASC is a modular workflow providing integrated analysis environment (from counts generation to<br>cell subpopulation identification) exploiting docker containerization to achieve both functional and<br>computational reproducibility in data analysis. Hence, rCASC provides preprocessing tools to remove<br>low quality cells and/or specific bias, e.g. cell cycle. Subpopulations discovery can be instead<br>achieved using different clustering techniques based on different distance metrics. Quality of clusters<br>is then estimated through a new metric namely Cell Stability Score (CSS), which describes the<br>stability of a cell in a cluster as consequence of a perturbation induced by removing a random set of<br>cells from the overall cells' population. CSS provides better cluster-robustness information than<br>silhouette metric. Moreover, rCASC provides also tools for the identification of clusters-specific<br>gene-signature.<br>Conclusions<br>rCASC is a modular workflow with valuable new features that could help researchers in defining<br>cells subpopulations and in detecting subpopulation specific markers. It exploits docker framework<br>to make easier its installation and to achieve a computation reproducible analysis. Moreover, a Java<br>Graphical User Interface (GUI), is also provided in rCASC to make friendly the use of the tool even<br>for users without computational skills in R.<br>Keywords<br>Single-cell data preprocessing, workflow, GUI, clustering, cluster stability metrics, cluster-specific<br>gene signature.
**Corresponding Author:**	Marco Beccuti, Ph.D<br>Universita degli Studi di Torino

Powered by Editorial Manager® and ProduXion Manager® from Aries Systems Corporation

	Turin, Piemonte ITALY
**Corresponding Author Secondary Information:**	
**Corresponding Author's Institution:**	Universita degli Studi di Torino
**Corresponding Author's Secondary Institution:**	
**First Author:**	Luca Alessandrì, Ph.D student
**First Author Secondary Information:**	
**Order of Authors:**	Luca Alessandrì, Ph.D student
	Francesca Cordero, Ph.D.
	Marco Beccuti, Ph.D.
	Maddalena Arigoni, Ph.D.
	Martina Olivero, Ph.D.
	Greta Romano
	Sergio Rabellino
	Gennaro De Libero, Ph.D.
	Luigia Pace, Ph.D.
	Raffaele Calogero
**Order of Authors Secondary Information:**	
**Response to Reviewers:**	GIGA-D-18-00522<br>Dear Editor,<br>First of all, we wish to thank the reviewers for their valuable comments and useful suggestions which<br>helped us to improve the paper and the tool.<br>Editor request: Please register any new software application in the SciCrunch.org database to<br>receive a RRID (Research Resource Identification Initiative ID) number, and include this in your<br>manuscript. This will facilitate tracking, reproducibility and re-use of your tool.<br>Answer: we registered rCASC to SciCrunch and it is now associated with the Research Resource<br>Identification Initiative ID : SCR_017005<br>Reviewer #1:<br>Comments to the Author:<br>This paper presents a pipeline to infer single-cell clusters using scRNA-Seq data (rCASC), infer<br>significant features linked to each cluster, and can analyse various metrics during the processing.<br>Notably, the results of the pipeline can be divided into 3 major outputs, A) cells-features matrix<br>generation, B) Clustering, and C) inference of significant features per clusters. Also, the pipeline is<br>able to perform various additional substeps such as Matrix preprocessing (normalization), outliers<br>removal, features removal, cell cycle specific features removal. The pipeline is implemented in R<br>using Docker containers and has a GUI interface coded in Java. Finally; the authors claimed have<br>invented a metric: the CSS, to evaluate cluster stability in their single-cell analyses. First, It is a<br>pleasant surprise to be able to install everything needed to perform scRNA-Seq analysis with few<br>simple commands (with exception of Docker which can be tricky for non IT people). Also, developing

scRNA-Seq analytical toolbox easy to use and efficient are an innovative direction due to the
importance and the multidisciplinary aspect of the field. However, I have major concerns which I
think should be addressed before publication.
Major Comments:
Comment 1: First, the abstract and the text contain different confusing aspects that must be
rewritten. The authors describe a "supervised approach": SIMLR which is seen as the alternative of
the "Seurat clustering". From my knowledge, SIMLR is a clustering workflow and thus is also an
unsupervised approach, by contrast with any other supervised approaches using training datasets
as input (classification/regression...). I don't know what is a "supervised clustering" if not a
classification procedure. Clustering are always unsupervised with the exception of "semi-
supervised" clustering (use of seed samples).
Answer 1: As pointed out by the reviewer both Seurat and SIMLR use an unsupervised approach,
and they mainly differ in the metrics driving the clustering analysis. SIMLR is capable of learning an
appropriate cell-to-cell similarity metric from the input single-cell data and to exploit it for the
clustering task. In details, the learning phase identifies a distance metric that best fits the structure
of the data by combining multiple Gaussian kernels. This allows the tool to deal with the large noise
and drop-out effect of single-cell data that could not be easily fitted with specific statistical
assumptions made by standard dimension reduction algorithms. Differently, Seurat clustering
algorithm is based on the Euclidean distance in PCA space, and refines the edge weights between
any two cells based on the shared overlap in their local neighbourhoods (Jaccard similarity). Since
these two clustering approaches have their specific criticality and strengths we decided to integrate
both of them in our framework. Finally, according to the reviewer' comments we modified the
following sentences:
In Abstract-Findings: “Subpopulations discovery can be instead achieved using unsupervised and
supervised clustering technique”
was modified in
“Subpopulations discovery can be instead achieved using different clustering techniques based on
different distance metrics”.
In keywords: “supervised clustering, unsupervised clustering”
was modified in
“clustering”
In Findings: “Therefore, rCASC provides raw data preprocessing, subpopulation discovery via
supervised/unsupervised clustering and cluster-specific genes-signatures detection”
was modified in
“Therefore, rCASC provides raw data preprocessing, subpopulation discovery via different
clustering approaches and cluster-specific genes-signatures detection”
“rCASC implements as unsupervised clustering tool and as supervised clustering tools”.
was modified in
“rCASC integrates two clustering tools, namely Seurat [13] and SIMLR [15], which mainly differ in

the metrics driving the clustering analysis".
Comment 2: Also, I don't understand why this package is superior in term of "Computational and
Functional" reproducibility compared with any other packages for which a similar reasoning can be
also applied.
Answer 2: We would like to thank the reviewer to give us the possibility to explain better this aspect.
rCASC is one of the tools developed under the umbrella of the Reproducible Bioinformatics project
(http://www.reproducible-bioinformatics.org/), an open-source community aimed to provide to
biologists and medical scientists, without scripting skills, a easy to use framework, which also
guarantees the ability to reproduce results independently by the underlying hardware, using docker
containerization (computational reproducibility), and providing tools that fulfil the best practice
rules for reproducible computational research, proposed in 2013 by Sandve [PLoS computational
biology 2013, 9(10)] (functional reproducibility). The reproducible bioinformatics project was
founded and it is maintained by the research team of Elixir node at University of Turin. The
reproducible bioinformatics project was published on BMC Bioinformatics (Kulkarni et al. 2018). An
example of stand-alone hardware/software infrastructure for bulk RNAseq, developed within the
Reproducible Bioinformatics project, was described in Beccuti et al. (Bioinformatics 2017). Thus, to
the best of our knowledge, rCASC is the only computational framework, which provides a complete
computational reproducibility for integrated analysis of single cell data (from counts generation to
cell subpopulation identification). Last but not least rCASC is listed within the tools developed by
the Italian Elixir node (https://bio.tools/rCASC).
Finally, the following phrase in the Findings section: "In this context rCASC provides a modular
workflow to address at the same time the problem of functional and computational reproducibility.
rCASC provides single cell analysis functionalities within the reproducible rules described by Sandve
[5]. rCASC is part of the Reproducible Bioinformatics Project [6], which is a project designed to
provide to the biological community a reproducible and user-friendly bioinformatics ecosystem [8]."
was modified in:
"rCASC is one of the tools developed under the umbrella of the Reproducible Bioinformatics project
(http://www.reproducible-bioinformatics.org/), an open-source community aimed to provide to
biologists and medical scientists, without advance scripting skills, an easy to use framework, which
also guarantees the ability to reproduce results independently by the underlying hardware, using
docker containerization (computational reproducibility). Indeed, it was developed following the
best practice rules for reproducible computational research, proposed in 2013 by Sandve [PLoS
computational biology 2013, 9(10)] (functional reproducibility). The reproducible bioinformatics
project was founded and it is maintained by the research team of Elixir node at University of Turin.

The reproducible bioinformatics project was published on BMC Bioinformatics (Kulkarni et al. 2018).
An example of stand-alone hardware/software infrastructure for bulk RNAseq, developed within
the Reproducible Bioinformatics project, was described in Beccuti et al. (Bioinformatics 2017). Thus,
to the best of our knowledge, rCASC is the only computational framework, which provides both
computational and functional reproducibility for an integrated analysis of single cell data, from
counts generation to cell subpopulation identification. rCASC is also listed within the tools
developed by the Italian Elixir node (https://bio.tools/rCASC).”
Comment 3: Then, the authors claimed to have invented a new metric: the "Cell-stability Score",
which is based on the computation of a stability score by clustering multiple bootstrap sampling and
computing the jaccard index. Clustering stability measurement is not new and previous works
already described more formally the use of bootstrapping together with clustering and Jaccard index
to estimate cluster stability
(http://www.homepages.ucl.ac.uk/~ucakche/papers/clusta.pdf (2006),
https://arxiv.org/abs/1503.0205). These example algorithms are not based on single-cell datasets
(other stability approaches exist for single-cells), but since the approach described in the first paper
is very similar, a more comprehensive bibliography of clustering stability should be present in the
manuscript as well a rewriting of the CSS description/notion, highlighting the similarity with
previous works.
Answer 3: As mentioned by the reviewer the cell stability score method implemented by us uses
Jaccard index, which is also used in Hennig paper
(http://www.homepages.ucl.ac.uk/~ucakche/papers/clusta.pdf 2006). However, between our
approach and the one of Hennig’s paper there is a substantial difference: in Hennig’s paper the
Jaccard index is used to evaluate the similarity between clusters and the calculated score provides
an overall quality score for each of the clusters in toto. In our implementation the stability score is
not related to the clusters, but it is specific for each cell. We believe that cell stability score allows
us to have a more precise view of which are the cells affected by perturbations of the dataset
structure.
To better explain this issue, we modify the paper section “rCASC: a single cell analysis workflow
designed to provide data reproducibility.”, adding the following phrases before the sentence: “To
the best of our knowledge, rCASC is the only workflow performing clustering in presence of data
perturbation ...”:
“Cluster stability is an important topic in Clustering (for a review see von Luxburg

Powered by Editorial Manager® and ProduXion Manager® from Aries Systems Corporation

2010). Stability
measurement, taking advantage of bootstrapping, was also addressed by Hennig (2007). Specifically,
Hennig uses Jaccard index to evaluate the overall stability of each cluster. In rCASC, we have
implemented a cell stability score (CSS), which uses the Jaccard index to estimate the stability of
each cell in each cluster. CSS provides an enhanced description of each cluster, since it allows the
identification of subset of cells, in any cluster, which are particularly sensitive to perturbation of the
overall dataset structure, i.e. cell bootstrapping. Moreover, the cluster stability measurement
proposed by Henning was included in rCASC. Specifically, we have implemented the "clusterboot
"function from the fpc R package (https://cran.r-project.org/web/packages/fpc/index.html), which
allows the evaluation of the cluster stability using a personalized clustering function). So far in our
knowledge, rCASC is the only single-cell analysis workflow performing clustering in presence of data
perturbation ...". We added the Section 5.3 in Supplementary file to describe this functionality.
Comment 4: In term of additional experiments, I think it would be interesting to have an idea of the
ratio: number of CPUs/ RAM/ computational time according to: the number of cells / number of
features (i.e.: matrix dimension), and read depth (linked to fastq size). More specifically, what are
the limiting steps in term of computation? What are the steps the less expensive ? A new figure
might be necessary to represent the contribution of each step in term of computation.
Answer 4: We thanks the reviewer for this comment. We added a new figure describing the effect
of cells and number of genes on the computation efficacy of SIMLR, Seurat, tSne and griph (Figure
7). In the Methods section we also added the paragraph "Scalability", in which we discussed the
computational performance of the used methods.
See also A9 major comments reviewer 2 and A6a to your Comment 6.
Comment 5: Ideally, a comparison with the other cited pipeline would be also interesting, but this
amount of work might be out of scope of this study.
Answer 5: We agree with reviewer that this point is out of the scope of our manuscript.
Comment 6:
Q6a) I have some concerns with the choice of clustering algorithms used. Despite Seurat is well
established in the community and SIMLR is also a well recognized algorithm, I am not sure if these
algorithms can handle very large sparse datasets (i.e. more than 10K cells), that are becoming the
new standard in the field. Notably, are these algorithms able to handle sparse data? SIMLR needs a
specified K, thus inferring the best K requires to screen amongst an array of Ks and thus might be
very time consuming. Would it exist better and simpler alternatives to handle very large and sparse
datasets that might be included in rCASC?
A6a) To answer to the above comment we run a performance experiment increasing the cell size
and varying the number of genes used in clustering experiment. Please see Answer A9 major
comments reviewer 2. A new paragraph, scalability, was added in method section in the main

Powered by Editorial Manager® and ProduXion Manager® from Aries Systems Corporation

manuscript. In Figure 7A of paragraph scalability, it is shown the computing time (Intel i7 3.5 GHx, 4
cores, 32 GB RAM, 500GB SSD) required to execute 160 permutations on a dataset varying from 200
to 5000 cells using SIMLR, tSne, griph and Seurat. Specifically, Seurat analysis with 5000 cells can be
completed in 50 hours. Computing times can be significantly reduced if a high-end multi-cores
server is used. We observe that when the number of considered cells overcomes 5000 units then all
the clustering tool integrated in rCASC requires more than 32GB RAM.
SIMLR, tSne, griph and Seurat were all designed to work with sparse data.
We agree with reviewer that SIMLR is more computational demanding with respect to the other
implemented tools, but it provides very good clustering performance as reported in SIMLR paper
(Wang et al. 2017). SIMLR was also released in a version to handle large scale dataset, but we did
not integrate it because, when we tested it, we observed that the sensitivity and specificity of the
method were not better of those of tSne (not shown in this manuscript). Of course, we are
constantly looking for new clustering methods, proposed in the literature, able to overcame the
limitations of the current tools. The latest comparison between clustering methods (Duò et al. 2018
on F1000: https://www.ncbi.nlm.nih.gov/pmc/articles/PMC6134335/) indicates that the clustering
algorithm implemented in Seurat toolkit is the most computationally time efficient.
Q6b) Using a clustering stability metric is I think a very good idea. Is it possible to get an average
stability score per cluster to have an idea if a cluster is noisy or robust?
A6b) We thank the reviewer with this useful comment. We answer to this point at the above
comment 3.
Q6c) Also, even a stable cluster according to a bootstrap experiment is not a guarantee of a
"biologically" stable cluster, and can reflect a biased in the method used (for example, a dummy
algorithm clustering cells according to their name will produce very stable but useless clusters).
A6c) According to one of the requests of reviewer 2, see A2b major comments reviewer 2, we
collected extra data, which are indicating that cell stability score (CSS) inversely correlate with the
cells' heterogeneity of clusters, see Section 5 Figure 41 of the supplementary information. Since, we
observed that the clusters characterized by high CSS are mainly constituted of cells of the same type,
this suggests a correlation with the biological characteristics of the cells.
Q6d) What is the use of griph (Graph Inference of Population Heterogeneity). Why not using the
stability measure to estimate the best K?
A6d) Indeed, we use the clusters stability measure to estimate with K is the best choice. However,
to provide a suggestion on the range of numbers of clusters to be investigated, we decided to use
griph tool, first because it is based on Louvain modularity optimization algorithm, as the Seurat
clustering method we have integrated in rCASC. Then, because its execution time is the best
between the clustering tools we have implemented in rCASC, see Figure 7 in the main manuscript.
We did not implement griph as complete clustering tool in rCASC, because it is not

published, yet.
Q6e) The package requires a very large amount of memory to be able to install all the docker
dependencies and I was not able to install it on my own computer (out of memory). Is there any way
to propose "lighter" versions in order to be able to use it on a standard computer? Overall, I am not
sure if all these different steps are always mandatory to obtain biologically meaningful single-cell
clusters (Of course, they might be required in some specific cases), compared to more
straightforward approaches (matrix creation -> embedding -> clustering).
A6e) Concerning the docker images storage in local computer, it is not necessary to execute the
command downloadContainers(), which download all rCASC docker images. In this case, only the
required images will be downloaded on the basis of the rCASC function in use. This option will
require extra time to execute each new function, since the corresponding docker image needs to be
downloaded. We have also added the option “mini” for downloadContainers(), which downloads
only 17 dockers out 26 docker images and provides all basic functionalities to handle a single-cell
counts table.
Concerning RAM requirements, we can execute all rCASC functions with 16 GB RAM on datasets up
1000 cells. The main limitation is given by the possibility of performing no more than one
permutation at a time.
Minor Comments:
Q1) The supplementary files document very rigorously the software which is really pleasant.
Some figures are not very informative and might be combined together (For example figures 1, 3
and 4 And figures 2 and 6?).
A1) We thank reviewer for the suggestion, but we prefer to leave these figures separated to keep
clear the analysis workflow structure.
Q2) Can you describe briefly what is the Seurat specific normalization?
A2) Seurat normalization step is the one suggested from the Seurat workflow. This information was
added in supplementary file: “Before clustering data are normalized as suggested from the Seurat
workflow using Seurat NormalizeData function, with LogNormalize as normalization.method and
10000 as scale.factor parameters.”
Reviewer #2: The authors present rCASC, an integrated analysis framework for single-cell RNA
sequencing data that combines a range of existing and novel computational tools. While some
workflows for reproducible scRNA-Seq data analysis exist, the authors provide an analysis strategy
using docker containers and a graphical user interface for reproducibility. rCASC is implemented as
an R package and as graphical user interface, which allows bioinformaticians as well as biologists
with little experience to perform statistical data analysis. However, there are some concerns about
the chosen analysis tools and the presentation of results that need to be addressed:
Major comments
Q1) The description of the algorithms used is often not clear. In the main text (p.4 l.16-21), the
authors mention normalization and clustering strategies taken from other tools. When other tools

	are used, it would be good to briefly describe the underlying algorithms (either in the main text or<br>the methods section). For example, Seurat is a toolbox for data analysis and not a clustering tool<br>and it has to be specified which clustering strategy is used. The authors should also make sure to<br>properly cite the original publication of functions implemented in the rCASC toolbox (for example<br>the reCAT function and the griph package).<br>A1) We added for each function described in section Methods a brief description of the implemented algorithm. We apologize for the error in citing reCAT, we fixed it. Concerning griph,<br>we only provided the github repository, because developers told us that it will be published as part<br>of a larger paper entitled “Self-organization and symmetry breaking in intestinal organoid<br>development” accepted for publication in Nature. However, at the time of this rebuttal the paper is<br>not out, yet. We will update its reference as soon as it will be available.<br>Q2a) The authors developed the Cell Stability Score based on iteratively clustering a sub-sampled<br>dataset. This is a good approach and informative for cluster stability. It would however be good to<br>visualize the stability scores for datasets subsampled to 20%, 30%, and even 50%.<br>A2a) We extended the analysis described in Figure 38 of the vignette removing 20%, 30% and 50%<br>of the cells. The results were added as Figure 40 and commented in vignette text: “The effect of the<br>perturbations induced in the clustering upon the removal of 10%, 20%, 30% and 50% of the data set<br>was also investigated in SetA (annotated_bmsnkn_5x100cells.txt), Figure 40. We can observe that<br>the overall cell stability score of each cell in each cluster is reduced increasing of the fraction of cells<br>removed in each permutation. However, the reduction in CSS is not identical for all clusters. In Figure<br>40, it is clear that cluster 2, completely composed of NK cells, is the most stable cluster to the<br>perturbations induced by increasing the number of removed cells. On the other side, cluster 4,<br>mainly made by stem cells (92 cells), together with few B-cells (2 cells) and Monocytes (7 cells) is<br>the least stable. Sorting by increasing CSS the cells in cluster 4, Figure 40D, all B-cells and Monocytes<br>are found within the first 15 most unstable cells.”<br>Q2b) Furthermore, it is crucial to link the CSS to the clustering ground truth with the underlying<br>assumption that the true discovery rate for "stable" cells is larger than the one for cells with lower<br>CSS.<br>A2b) We have investigated the clusters composition of two sets of cells described in section 4.2:<br>SetA and SetC. SetA is composed by cells with different biological characteristics, i.e. (B) B-cells, (M)<br>Monocytes, (S) Stem cells, (NK) Natural Killer cells, (N) Naive T-cells. SetC contains Monocytes,<br>Natural Killer cells and with T-cells subpopulations, i.e. (C) Cytotoxic T-cells, (H) T-helper cells and<br>Naive T-cell. The results are summarized in Figure 41 and commented in the vignette text: “We<br>investigated clusters composition of two sets of cells described in section 4.2: SetA and SetC.<br>SetA composed by cells with different biological characteristics, i.e. (B) B-cells, (M) Monocytes, (S)

Stem cells, (NK) Natural Killer cells, (N) Naive T-cells.
SetC contains Monocytes, Natural Killer cells and with T-cells subpopulations, i.e. (C) Cytotoxic T-
cells, (H) T-helper cells and Naive T-cell, Figure 41. CSS provides indications on clusters cells
heterogeneity. In Figure 41A and C, clusters characterized by high cell stability score are mainly
constituted by one cell type. On the other hand, as CSS decrease, Figure 41B and D, the clusters
become characterized by an increasing heterogeneity in the cell types composition. E.g. In Figure
41B and D, cluster 5 (violet) has a CSS between 75% to 100% and it is composed only by monocytes,
cluster 2 (light green), which has a CSS between 50% and 75%, has a 11% contamination of T-helper
cells. Cluster 3 (green), which has a CSS between 25 to 50%, is contaminated by 12% Cytotoxic T-
cells and 4% T-helper cells. Finally, clusters 1 and 4, characterized by CSS between 0% to 25%, are
very heterogeneous incorporating 4 out of 5 cell types present in this dataset.".
Q3) The authors should discuss why they chose to store e.g. normalized data and analysis results
in .csv or .txt files rather than using slots of a S4 class (in sparse matrix format) commonly used in R
data analysis.
A3) All tools embedded in rCASC use as input source a tab or a comma delimited file and only Seurat
imports counts table file in an object of class Seurat. 10XGenomics distributes counts table file also
in H5 (Hierarchical Data Format 5 File) format, Bioconductor rhdf5 package offers support for this
type of files, but none of the tools implemented in rCASC is able handle the R data structure returned
by the rhdf5 package. Since, as far as we know there is not a general agreement on the format of
single cell count tables we prefer to wait till an agreement on standardized data format will become
available.
Q4) In the vignette is it often not clear what the scale bars or colour coding means. The authors
should expand figure legends, plots and axes with the necessary information to understand what is
displayed.
A4) We carefully revised the figure legend to clarify colours and legend bars.
Q5) When performing dimensionality reduction it is important to i) correctly normalize the data and
ii) indicate if the counts were log-transformed. These information are missing in some parts of the
vignette. For example the authors use a wrapper function to perform PCA on page 17. It is not clear
if the data was normalized and log-transformed which could have an impact on the interpretability
of the results.
A5) We carefully revised the various part of the vignette and we added information referring to the
data characteristics.
Q6) The scannobyGtf function performs gene annotation and removes mitochondrial genes and
genes encoding ribosomal proteins. For some analyses, these genes can be informative regarding
the metabolic and proliferative state of the cell and should not be removed. The authors should
therefore consider splitting the scannobyGtf function into two functions; one for annotation and

one for filtering. If users detect unwanted variation in the expression of genes encoding for
ribosomal proteins, this effect should be removed using regression approaches such as scLVM rather
than excluding the genes from the dataset.
A6) The scannobyGtf function was updated to make optional the removal of mitochondrial and
ribosomal protein genes.
Q7) Section 3.5 Top expressed genes: By selecting the set of top expressed genes the authors might
be biased by the variation detected in highly expressed housekeeping genes. Instead, and in line
with commonly performed analysis for scRNA-Seq data, the authors should include an approach to
detect highly variable genes as the set of informative genes. This also facilitates the inclusion of cell-
type specific genes in the clustering approach (see paragraph on page 39 in the vignette).
A7) We thank reviewer for this useful comment. We have added the option to filter the dataset on
the basis of gene dispersion in the topx function. Section 3.5: “For clustering purposes user might
decide to use the top expressed genes. The function topx selects the X top expressed genes given a
user defined threshold.”
was modified in:
“For clustering purposes user might decide to use the top expressed/variable genes. The function
topx provides two options:
- the selection of the X top expressed genes given a user defined threshold, parameter type="expression"
-the selection of the X top variable genes given a user defined threshold, parameter type="variance"
The function also produces a pdf file gene_expression_distribution.pdf showing the changes in the
UMIs/gene expression distribution upon topx filtering.”
Q8) When toy datasets are used it is important to state if these are the data from the original
publication or if the data were pre-processed (see bottom of page 29 in the vignette).
A8) We updated the description of the toy experiments
Q9) The authors implemented different clustering strategies in the rCASC toolbox. While the cell
stability score is explained in detail, it is not clear what exactly is used for SIMLR and tSNE clustering.
Both are methods to perform non-linear dimensionality reduction and only SIMLR is designed to
also perform clustering. tSNE is not a clustering tool and it is well known that it introduces artefacts
when visualizing complex data. It would therefore be good to explain if the SIMLR internal clustering
approach is used or if the authors perform k-means clustering on the dimensionality reduced data-
points. For reasons of scalability and the number of input genes, I wonder whether the SIMLR
approach is suitable for large dataset (e.g. 50,000 cells).
A9) In vignette section 5 and 6 we described SIMLR and Seurat as the two clustering tools used in
rCASC. The choice of these two tools is given by the comparisons performed by Wang and coworkers
in their paper on SIMLR (Nat Methods. 2017) and by the independent observations of Freytag and
coworkers
(https://f1000research.com/articles/7-1297/v2)
and

Powered by Editorial Manager® and ProduXion Manager® from Aries Systems Corporation

Duo'
and
Robinson
(https://f1000research.com/articles/7-1141/v2) indicating that, in their tests, Seurat delivered the
overall best performance in cells clustering. The above-mentioned references are indicated in
section 5 and 6.
In section 5 we described that bootstraps are used to estimate the cell stability score using SIMLR.
To clarify that also in Seurat clustering the bootstraps are implemented we added the following
phrase in section 6: "The bootstrap approach described in section 5.1 is also applied to Seurat
clustering to assign cell stability score to the clustered cells."
In rCASC tSne is implemented using the Rtsne package (https://cran.r-project.org/web/packages/Rtsne/index.html). The tSne implementation is only used for
comparison with respect to SIMLR and Seurat in section 6.1. Rtsne package provides an internal k-mean clustering, which is performed on the dimensionality reduced data-points. To clarify this point
we added the following phrases in section 6.1: "The bootstrap approach described in section 5.1 is
also applied on tSne clustering to assign cell stability score to the clustered cells. In rCASC tSne is
implemented using the Rtsne (https://cran.r-project.org/web/packages/Rtsne/index.html) package.
Rtsne performs a data reduction on which k-mean clustering is applied."
Concerning scalability and the number of input genes a similar request was also asked by reviewer
1. Therefore, we run a scalability test using the GSE106264 dataset described in Section 8 of the
vignette. The scalability analysis is described in the main manuscript in materials section subsection
scalability: "To estimate the scalability of rCASC clustering we used the GSE106264 dataset made of
10035 cells and published by Pace and coworkers in 2018 [18]. We randomly sampled the 10035
cells (27998 ENSEMBL GENE IDs) to obtain the following subsets of cells: 400, 600, 800, 1000, 2000,
5000. Starting from the 800 cells set we randomly sampled the genes: 10000, 8000, 6000, 4000,
2000, 1000, 800. We run SIMLR, tSne, griph and Seurat using 160 permutation within SeqBox
hardware [26]: Intel i7 3.5GHz (4 cores), 32 GB RAM and 500 GB SSD disk. SIMLR resulted to be the
slowest and, given the used workstation, it cannot allocate for the analysis more than 2000 cells
(Figure 7A). All the other tools were able to handle up to 5000 cells, within the limit of 32 GB of RAM
available in the hardware setting used in this analysis. Computation time was nearly linear for all
tools till 1000 cells. Only griph clustering resulted to be nearly insensitive to the increasing number
of cells (Figure 7A). The computing time as function of increasing number of genes has

a quite
limited effect on the overall computing time (Figure 7B).”
Q10) There are typos and phrasing issues in the figure legends and the vignette. For example, the
legend of figure 2 needs editing to make it understandable.
A10) We thank reviewer for the careful and precise reading of our manuscript. We edited the
vignette to eliminate typos and phrasing issues.
Minor comments
Q1) The processing of raw sequencing data in the form of fastq files is computationally expensive
and usually performed on high performance computing systems. The authors decided to implement
wrapper functions to process 10X and inDrop data. While these technologies are used by the
majority of the field, other technologies generate individual fastq files per cell and a function could
be implemented to process this data. Furthermore, the authors use the pre-build genomes supplied
by 10X for the cellranger pipeline but on the other hand build the reference for the inDrop pipeline
from scratch. These approaches are not comparable since the 10X genomes are filtered to only
include protein-coding genes. The authors should therefore implement a wrapper function for the
cellranger mkref call to allow a more flexible use of genomic references.
A1) We thank reviewer for the useful comments. Concerning the processing of fastq data we
implemented inDrop and 10XGenomics fastq processing software because they are compliant with
the minimal hardware requirements indicated in Section 1.1.
For smart-seq we have added into the vignette the following paragraph: “Section 2.3 Smart-seq full
transcript sequencing.
Smart-seq protocol generates a full transcript library for each cell, i.e. a fastq file for each cell. To
convert fastq in counts we suggest to use rnaseqCounts or wrapperSalmon counts from docker4seq
package [Kulkarni et al.]. Both above-mentioned functions are compliant with minimal hardware
requirements indicated for rCASC and are part, as rCASC, of the Reproducible Bioinformatics Project.
The function rnaseqCounts is a wrapper executing on each fastq:
• quality evaluation of fastq with FastQC software,
• trimming of adapters with skewer,
• mapping reads on genome using STAR and counting isoforms and genes with RSEM.
The function wrapperSalmon instead implements FastQC and skewer and calculates isoforms and
genes counts using Salmon software.”
We have also implemented a new function called cellrangeIndexing, which allows the generation of
10Xgenomics compliant reference genome. The description of cellrangeIndexing was added to the
vignette in Section 2.2.
Q2) The framework is developed to run on a linux machine and it would be useful to provide an
implementation for Mac and Windows.
A2) The rCASC framework was developed to be compliant with SeqBox (Beccuti et al. Bioinformatics
2017), i7 3.5GHz, 32GB RAM, 500 GB SSD running linux. We have tested rCASC with the latest version
of Docker Desktop (v 2.0.0.3) on a Mac machine with 16 GB RAM i7 GHz 3.5, 4 cores. Configuring

Powered by Editorial Manager® and ProduXion Manager® from Aries Systems Corporation

	the virtual machine to use 12 GB RAM and 2 cores we can execute all examples provided in the<br>rCASC vignette. However, RAM requirements become quite demanding, exceeding 12 GB, when<br>more than 1000 cells are used for clustering. We decided to not extend the rCASC framework to<br>window platform. Instead, within the Elixir framework, we are in the early phase of porting rCASC<br>(https://github.com/pmandreoli/rCASC_wrappers) in LANIAKEA galaxy (https://elixir-italy-science-<br>gateway.cloud.ba.infn.it/) in collaboration with LANIAKEA's developers.<br>Q3) On page 16 in the vignette, the authors discuss the relationship between the number of reads<br>per cell and the number of genes detected. While this dependency is known, the authors should<br>acknowledge that different cell-types show differences in their transcriptional rate and that a<br>technical assessment of the reads per cell vs. genes detected is difficult to perform when comparing<br>different cell-types.<br>A3) We thank reviewer for this important indication. To incorporate reviewer's suggestion, we<br>added in the page 16 the following phrase: "However, it has to be underlined that each cell type is<br>characterized by a peculiar transcriptional rate and therefore the technical assessment of the reads<br>per cell vs. genes detected between different cell types, i.e. Figure 13A-C, might be bias by<br>differences in the transcriptional rate of the different cells used in this specific example. Instead, the<br>above-mentioned bias does not affect Figure 13D-F because they are generated by a down sampling<br>of a set of cells sequenced with the smart-seq protocol at a coverage of 1 million reads/cell."<br>Q4) Figure 16, page 20: It is not possible to identify the cells that were removed after filtering.<br>A4) In Figure 16 the removed cells are those labelled in blue. Figure 16 was modified adding arrows<br>to better highlight cells that were removed and Figure 16 legend: "Effect of Lorenz filtering, cells<br>shown in blue have been discarded because of their low quality"<br>was modified in the following way:<br>"Lorenz filtering: cells retained after filtering are labelled in red as instead cells discarded because<br>of their low quality are labelled in blue."<br>Q5) Figure 21 needs more explanation in the figure legend<br>A5) the phrase: "checkCountDepth output plot" was modified in: "checkCountDepth output plot<br>provides an evaluation of count-depth relationship in un-normalized data. The effects of the<br>normalization procedure is shown in the following figure."<br>Q6) It is not possible to see the CSS for individual cells as displayed by the authors. I would<br>recommend displaying a side-by-side plot where cells in one plot are coloured by cluster ID and cells<br>in the other plot are coloured based on their CSS.<br>A6) A new plot was added to the NameOfCountMatrix_Stability_Plot.pdf file, which provides the<br>results of the clustering. The new plot provides the cluster picture with cell coloured on the basis of<br>their CSS. Figure 38 was also modified to include this new plot. CSS is described with the following<br>colours: 0-25% black, 25-50% green, 50-75% gold and 75-100% red. The phrase: "The plot in Figure

38C provides a 2D view of the clustering results. In this plot each cell is labeled with a symbol
indicating its cell stability score."
was modified in the following way:
"In each clustering folder there is a pdf named NameOfCountMatrix_Stability_Plot.pdf, which
contains two plots (Figure 38C-D) generated by the clustering program. These plots provide a 2D
view of the clustering results from two different perspectives. In Figure 38C plot each cell is coloured
on the basis of the belonging cluster and it is labeled with a symbol indicating its cell stability score
(CSS). Instead, in the plot in Figure 38D each cell is coloured on the basis of its CSS: 0-25% black, 25-
50% green, 50-75% gold and 75-100% red."
Q7) Page 51 in the vignette: there is a broken link to one figure
A7) We fixed it
Q8) There is a colouring discrepancy between Figure 51 (Z score transformed counts) and Figure 54
(log10-tranformed counts) where the rCASC uses the same colour scale.
A8) Now the colours of Fig. 51 B and C correspond to those in Fig. 54 B and C.
Q9) Page 66 in the vignette: the authors should better explain why they chose k=6 and not k=7 and
where the difference between Figure 57 A and B is coming from.
A9) To clarify the description of fig 57, the phrase:
"In Figure 57 are summarized the results of the analysis executed on the Pace's dataset. Data
perturbations, Figure 57A, allows data organization between 6 to 9 clusters, where 7 clusters is the
most represented group. Cell stability score, from the SIMLR analysis executed on the above range
of clusters, is shown in Figure 57B. Six clusters show a slightly higher stability with respect to the
others. The overall stability of 6 clusters is however sub-optimal, since it is spread between 0 and
0.9 cell stability score. In Figure 57C it is shown the clusters structure generated with SIMLR on 6
clusters. Clusters 1, 3 and 4 show a quite good stability, Figure 57C. Cluster 3 is made of 44 N (88%)
and 48 Nd (96%), suggesting that naive CD8+ T lymphocytes are not affected by the silencing of
Suv39h1 gene. Cluster 1 contains 16 NA (6.4%) and 14 NdA (5.6%). Cluster 2 is made of 44.8% of NA
and 39.6% of NdA cells. Clusters 1 and 2 group together, interdependently from Suv39h1 gene
silencing. Cluster 6 is made of 35% NA and 13.6% of NdA. In cluster 6 the amount of NA and NdA is
unbalance, suggesting that the Suv39h1 silencing does not guarantee the efficient differentiation of
the cell subpopulation in cluster 6. Cluster 4 only contains NdA (33%), indicating that at least a
subpopulation of activated Suv39h1-silenced cells has a specific transcription profile that
differentiate them from the wild type activated cells. Cluster 5 is made of 6 N cells, 2 Nd cells, 34 NA
cells, 21 NdA cells. Despite the presence of a limited amount of naive cells, which might be explained
as partially activated, cluster 5 is made mainly of activated cells, i.e. 13.6% NA and 8.4% NdA of total
cells. The cluster structure (Figure 57D) and cell cluster stability scores (Figure 57C) might suggest
that cluster 5 is made of a precursor subset."
was modified in the following way:
"In Figure 57 are summarized the results of the analysis executed on the Pace's

dataset. A limitation
of the clustering based on SIMLR is due to the need of providing as input the number of clusters (k)
in which the data should be organized. Instead of asking to user to define arbitrarily the k number
of clusters, we used griph (https://github.com/ppapasaikas/griph) as tool to identify a range of k
clusters to be inspected by SIMLR. Figure 57A shows the frequency of the k number of clusters, in
which the Pace's dataset can be organized using griph software, upon 160 bootstraps in which 10%
of the cells is randomly removed from the initial data set. Griph analysis identify a range of clusters
going from k=6 to k=9. K=7 is the most represented data organization detected by griph, followed
by 8, 6 and 9 clusters.
The range of k clusters detected using griph is then investigated with SIMLR. SIMLR is run for each k
of the k-range defined with griph tool. CSS violin plot (Figure 57B) shows that the mean stability for
k=6 (CSSm ~ 0.5) is higher than to the others ks (CSSm < 0.3).
Clusters k=7 and k=8 do not represent the most stable organizations in terms of CSS (Figure 57B),
although they are the most frequent organizations observed in griph analysis (Figure 57A).
Since the best CSSm is observed in k=6, we explored these clusters (Figure 57C). In Figure 57C,
clusters 1, 3 and 4 show a quite good stability, since cells stay in these clusters between 75% to
100% of the bootstraps.
The inspection of Pace's experiment groups organization (i.e. N= naïve WT, Nd= naïve Suv39h1 KO,
NA=activated WT, NdA=activated Suv39h1 KO) in k=6 clusters, Figure 57C, show that cluster number
4 is the only one containing only NdA (33% of the total NdA) cells. Thus, suggesting that a
subpopulation of activated Suv39h1-silenced cells has a specific transcription profile, which
differentiates them from all wild type activated cells. Another interesting cluster is number 6, where
the amount of NA and NdA is unbalance, 35% NA and 13.6% of NdA, suggesting that Suv39h1
silencing does not guarantee at the same efficiency the differentiation of this cell subpopulation as
in the case of wild type cells. Cluster 5 is the most heterogeneous cluster. It is composed by 6 N cells,
2 Nd cells, 34 NA cells, 21 NdA cells. Despite the presence of a limited number of naive cells, which
might be explained as partially activated, cluster 5 is composed by an unbalance number of activated
cells, i.e. 13.6% NA and 8.4% NdA of total cells. However, since cluster 5 is characterized by a very
low CSS (0-25%) it is possible that this cluster contains cells localized at the boundaries of clusters 2,
3 and 6. On the other side clusters 1, 2, 3 have nearly the same number of wild type and Suv39h1
silenced cells, suggesting that these subsets of cells are not influenced by Suv39h1 silencing:

•
•
•

cluster 1 contains nearly the same amount of activated wild type, 16 NA (6.4%), and Suv39h1
KO cells, 14 NdA (5.6%); cluster 2 is made of 44.8% of NA and 39.6% of NdA cells;

	cluster 2 is made of 44.8% of NA and 39.6% of NdA cells;<br>cluster 3 is made of 44 N (88%) and 48 Nd (96%)”
**Additional Information:**	
**Question**	**Response**
Are you submitting this manuscript to a special series or article collection?	No
**Experimental design and statistics**<br><br>Full details of the experimental design and statistical methods used should be given in the Methods section, as detailed in our Minimum Standards Reporting Checklist. Information essential to interpreting the data presented should be made available in the figure legends.<br><br>Have you included all the information requested in your manuscript?	Yes
**Resources**<br><br>A description of all resources used, including antibodies, cell lines, animals and software tools, with enough information to allow them to be uniquely identified, should be included in the Methods section. Authors are strongly encouraged to cite Research Resource Identifiers (RRIDs) for antibodies, model organisms and tools, where possible.<br><br>Have you included the information requested as detailed in our Minimum Standards Reporting Checklist?	Yes
**Availability of data and materials**<br><br>All datasets and code on which the conclusions of the paper rely must be either included in your submission or deposited in publicly available repositories (where available and ethically appropriate), referencing such data using a unique identifier in the references and in the “Availability of Data and Materials”	Yes

section of your manuscript.<br><br>Have you have met the above requirement as detailed in our Minimum Standards Reporting Checklist?	

Powered by Editorial Manager® and ProduXion Manager® from Aries Systems Corporation

Manuscript

Click here to view linked References

Click here to access/download;Manuscript;alessandri_et_al_gigascience_re

rCASC: reproducible Classification Analysis of Single Cell sequencing data

[1#]Luca Alessandrì, [2#]Francesca Cordero, [2$]Marco Beccuti, [1]Maddalena Arigoni, [3]Martina Olivero, [2]Greta Romano, [2]Sergio Rabellino, [4]Gennaro De Libero, [5*]Luigia Pace and [1*]Raffaele A Calogero

[1]Department of Molecular Biotechnology and Health Sciences, University of Torino, Via Nizza 52, Torino, Italy

[2]Department of Computer Sciences, University of Torino, Corso Svizzera 185, Torino, Italy

[3]Department of Oncology, University of Torino, SP142, 95, 10060 Candiolo TO, Italy

[4]Department Biomedizin, University of Basel, Hebelstrasse 20, 4031 Basel, Switzerland

[5]IIGM, Via Nizza 52, Torino, Italy

#Both authors equally contributed the present work

*Both authors equally supervised the present work

[$]Corresponding author

Luca Alessandrì alessandri.luca1991@gmail.com

Francesca Cordero fcordero@di.unito.it

Marco Beccuti beccuti@di.unito.it

Maddalena Arigoni maddalena.arigoni@unito.it

Martina Olivero martina.olivero@unito.it

Greta Romano greta.romano@unito.it

Sergio Rabellino sergio.rabellino@unito.it

Gennaro De Libero gennaro.delibero@unibas.ch

Luigia Pace luigia.pace@iigm.it

Raffaele A Calogero raffaele.calogero@unito.it

Abstract

Background

Single-cell RNA sequencing is an essential tool to investigate cellular heterogeneity, and to highlight cell sub-population specific signatures. Single-cell sequencing applications are now spreading from the most conventional RNAseq to epigenomics, e.g. ATAC-seq. Single-cell sequencing led to the development of a large variety of algorithms and tools. However, to the best of our knowledge, there are few computational workflows providing analysis flexibility and achieving at the same time functional (i.e. information about data and the utilized tools are saved in terms of meta-data) and computational reproducibility (i.e. real image of the computation environment used to generate the data is stored) through a user-friendly environment.

Findings

rCASC is a modular workflow providing integrated analysis environment (from counts generation to cell subpopulation identification) exploiting docker containerization to achieve both functional and computational reproducibility in data analysis. Hence, rCASC provides preprocessing tools to remove low quality cells and/or specific bias, e.g. cell cycle. Subpopulations discovery can be instead achieved using different clustering techniques based on different distance metrics. Quality of clusters is then estimated through a new metric namely Cell Stability Score (CSS), which describes the stability of a cell in a cluster as consequence of a perturbation induced by removing a random set of cells from the overall cells' population. CSS provides better cluster-robustness information than silhouette metric. Moreover, rCASC provides also tools for the identification of clusters-specific gene-signature.

Conclusions

rCASC is a modular workflow with valuable new features that could help researchers in defining cells subpopulations and in detecting subpopulation specific markers. It exploits docker framework to make easier its installation and to achieve a computation reproducible analysis. Moreover, a Java Graphical User Interface (GUI), is also provided in rCASC to make friendly the use of the tool even for users without computational skills in R.

Keywords

Single-cell data preprocessing, workflow, GUI, clustering, cluster stability metrics, cluster-specific gene signature.

Findings

rCASC: a single cell analysis workflow designed to provide data reproducibility.

Since the end of the 90's omics high-throughput technologies have generated an enormous amount of data, reaching today an exponential growth phase. The analysis of omics big data is a revolutionary means of understanding the molecular basis of disease regulation and susceptibility, and this resource is made accessible to the biological/medical community via bioinformatics frameworks. However, due to the increasing complexity and fast evolution of computation tools and omics methods, the reproducibility crisis [1] is becoming a very important issue [2] and there is a mandatory need to guarantee robust and reliable results to the research community [3].

Single cell analysis is instrumental to understand the functional differences existing among cells within a tissue. Individual cells of the same phenotype are commonly viewed as identical functional units of a tissue or organ. However, single cells sequencing results [4] suggest the presence of a complex organization of heterogeneous cell states producing together system-level functionalities. A mandatory element of single cell RNAseq is the availability of dedicated bioinformatics workflows. rCASC is one of the tools developed under the umbrella of the Reproducible Bioinformatics project (http://www.reproducible-bioinformatics.org/), an open-source community aimed to provide to biologists and medical scientists, without advance scripting skills, an easy to use framework, which also guarantees the ability to reproduce results independently by the underlying hardware, using docker containerization (computational reproducibility). Indeed, it was developed following the best practice rules for reproducible computational research, proposed in 2013 by Sandve [5] (functional reproducibility). The reproducible bioinformatics project was founded and it is maintained by the research team of Elixir node at University of Turin. The reproducible bioinformatics project was published on BMC Bioinformatics [6]. An example of stand-alone hardware/software infrastructure for bulk RNAseq, developed within the Reproducible Bioinformatics project, was described in Beccuti [7]. Thus, because of the philosophy of the Reproducible Bioinformatics project, to the best of our knowledge, rCASC is the only computational framework, which provides both computational and functional reproducibility for an integrated analysis of single cell data, from counts generation to cell subpopulation identification. rCASC is also listed within the tools developed by the Italian Elixir node (https://bio.tools/rCASC).

All computational tools in rCASC are embedded in dockers images stored in a public repository on docker hub. Parameters are delivered to docker containers via a set of R functions, part of rCASC R github package [8]. To simplify the use of rCASC package to users without scripting experience, R

functions can be controlled by a dedicated GUI, integrated in the 4SeqGUI tool previously published by us [7], which is also available as github package [9]. rCASC is specifically designed to provide an integrated analysis environment for cell-subpopulation discovery. The workflow allows the direct analysis of fastq files, generated with 10X Genomics and inDrop platforms, or count matrices. Therefore, rCASC provides raw data preprocessing, subpopulation discovery via different clustering approaches and cluster-specific genes-signatures detection. The key elements of rCASC workflow are shown in Figure 1, and the main functionalities are summarized in Methods section. A detailed description of the rCASC functions is also available in the vignettes section of rCASC github [8].

The overall characteristics of rCASC were compared with other four workflows for single-cells analysis (Figure 2): i) simpleSingleCell, Bioconductor workflow package [10]; ii) Granatum, web-based scRNA-Seq analysis suite [11]; iii) SCell, graphical workflow for single-cell analysis [12]; iv) R toolkit Seurat [13]. The comparison was based on the following elements: a) supported single-cell platforms, b) types of tools provided by the workflow, c) type of reproducibility granted by the workflow, d) usage flexibility.

rCASC is the only workflow providing support at fastq level because all the others packages require as input the processed counts table. Cell quality control and outliers' identification is available in all workflow but Granatum. Association of ENSEMBL gene IDs to gene symbols is only provided by rCASC. All workflows provide genes filtering tools but simpleSingleCell. All packages provide normalization procedures to be applied to raw counts data. However, rCASC is the only tool providing both Seurat specific normalization [13] and count-depth specific normalization [14]. The workflows implement different data reduction and clustering methods. rCASC integrates two clustering tools, i.e. Seurat [13] and SIMLR [15], which mainly differ in the metrics driving the clustering analysis. Notably, Freytag [16] recently published a comparison of single-cell clustering methods, in which SIMLR and Seurat were included. Freytag showed that the two methods performed better than other clustering methods and they behaved in similar way on Freytag's golden standard dataset. Cluster stability is an important topic in Clustering (for a review see [17]). Stability measurement, taking advantage of bootstrapping, was also addressed by Hennig [18]. Specifically, Hennig uses Jaccard index to evaluate the overall stability of each cluster. In rCASC, we have implemented a cell stability score (CSS), which uses the Jaccard index to estimate the stability of each cell in each cluster. CSS provides an enhanced description of each cluster, since it allows the identification of subset of cells, in any cluster, which are particularly sensitive to perturbation of the overall dataset structure, i.e. cell bootstrapping. Moreover, the cluster stability measurement proposed by Henning was included in rCASC. Specifically, we have implemented the "clusterboot" function

from the fpc R package [19], which allows the evaluation of the cluster stability using a personalized clustering function (see Supplementary file Section 5.3). To the best of our knowledge, rCASC is the only workflow performing clustering in presence of data perturbation, i.e. removal of a subset of cells, and measuring cluster quality using Cell Stability Score (CSS is a cluster quality metrics developed by us, which measures the persistence of each cell in a cluster upon data perturbation, see Supplementary file section 5.1) and Silhouette score (SS is a cluster quality metrics measuring the consistency within clusters of data). In our experiments, CSS provides a better estimation of the cluster stability with respect to what can be depicted using SS (Figure 2). Gene feature selection approaches are implemented in different way in the five workflows. Granatum is the only one providing biological inference. Granatum and Seurat implements various statistical methods to detect cluster specific genes signatures (Figure 3). rCASC embeds an ANOVA-like statistics derived from EdgeR Bioconductor package [20] and Seurat/SIMLR genes prioritization procedures (see Supplementary file section 7). Visualization of genes-signatures by heatmap, coloring cells on the basis of gene expression is only provided by rCASC (see Supplementary file Figure 51). Considering reproducibility, only rCASC provides both computational and functional reproducibility. Finally, rCASC is the only one providing both command line and GUI (Figure 4).

rCASC was used to re-analyze the single-cell dataset from Pace paper [21]. In this paper, authors highlighted that Suv39h1-defective $CD8^{+}$ T-cells show sustained survival and increased long-term memory reprogramming capacity. Our re-analysis extends the information described in Pace paper, suggesting the presence of an enriched Suv39h1-defective memory subset. A complete description of the above analysis is available at section 8 of supplementary file.

Methods

Counts table generation

inDrop single-cell sequencing approach was originally published by Klein [22]. Then, the authors published the detailed protocol in Nature Methods in 2017 [23]. In rCASC, the generation of the count table starting from fastq files refers to the version 2 of the inDrop chemistry described in [23], which is commercially distributed by 1CellBio. The procedure described in the inDrop github [24] in embedded in a docker image. rCASC function *indropIndex* allows the generation of the transcripts index required to convert fastq to counts and *indropCounts* function converts reads in UMI counts.
10XGenomics Cellranger is packed in a docker image and the function *cellrangerCount* converts fastq to UMI matrix using any of the genome indexes available at 10XGenomics data repository. Detailed description about the counts table generation is available in Supplementary file section 2.

Counts table exploration and manipulation

rCASC provides various data inspection and preprocessing tools.

genesUmi function generates a plot where the number of detected genes are plotted for each cell with respect to the number of UMI/reads quantified for each cell (Figure 5A,C).

mitoRiboUmi calculates the percentage of mitochondrial/ribosomal genes with respect to the total number of detected genes in each cell and plots percentage of mitochondrial genes with respect to percentage of ribosomal genes. Furthermore, cells are colored on the basis of the number of detected genes (Figure 5B, D). *mitoRiboUmi* allows to identify cells with low information content, i.e. those cells with a little number of detectable genes, e.g. < 100 genes/cell, little ribosomal content and high content of mitochondrial genes, which indicate cell stress [25].

The function *scannobyGtf* uses ENSEMBL gtf and the R package refGenome to associate gene symbol with the ENSEMBL gene ID. Furthermore, *scannobyGtf* allows the removal of mitochondrial/ribosomal genes (Figure 5A, C) and the removal of "stressed" cells detectable with *mitoRiboUmi* function (Figure 5B, D).

The function *lorenzFilter* embeds the Lorenz statistics developed by Diaz [12], a cell quality statistics correlated with cell live-dead staining (see Supplementary file sections 3.3). Specifically, the outlier filtering for single-cell RNA-seq experiments designed by Diaz estimates genes expressed at background levels in each sample, then samples with significantly high background levels are discarded [12].

As counts table preprocessing steps, we implemented the functions *checkCountDepth/scnorm* to detect the presence of sample specific count–depth relationship [14] (i.e. the relationship existing between transcript-specific expression and sequencing depth) and adjust the counts table for it. Specifically, *checkCountDepth* initially executes a quantile regression, thus estimating the dependence of transcript expression on sequencing depth for every gene. Then, genes with similar dependence are aggregated (see Supplementary file section Figure 21). *Scnorm,* after executing *checkCountDepth*, executes a new quantile regression to estimate scale factors within each group of genes. Then, sequencing depth adjustment is done within each group using the estimated scale factors.

Furthermore, we have added two other functions *recatPrediction* and *ccRemove,* which are based respectively on the paper of Liu [26] and Barron [27]. The function *recatPrediction* organizes the single cell data to reconstruct cell cycle pseudo time-series and it is used to understand if a cell cycle effect is present. The above function embeds reCAT software [26], which models the reconstruction of time-series as a traveling salesman problem, thus identifying the shortest possible cycle by passing through each cell exactly once and returning to the start. Since the traveling salesman problem is a NP-hard problem, reCAT is based on a heuristic algorithm, which is used to find the solution.

ccRemove function is instead based on the work of Barron and Li [27] and embeds their scLVM (single-cell latent variable model) algorithm, which uses a sophisticated Bayesian latent variable model to reconstruct hidden factors in the expression profile of the cell-cycle genes. This algorithm is able to remove cell-cycle effect from real scRNA-Seq datasets. Thus, *ccRemove* is used to mitigate the cell cycle effect of the inter-samples transcriptome, when it is detected by *recatPrediction* function (see Supplementary file sections 3.6 ad 3.8).

Clustering

For the identification of cell subpopulations we implemented two approaches: Seurat [13] and SIMLR [15]. Seurat is a toolbox for single-cell RNAseq data analysis. We have implemented in rCASC one of the clustering procedures present in Seurat toolbox. The function *seuratPCAEval* has to be run before executing the clustering program to identify the 'metafeatures', i.e. subset of PCA components describing the relevant source of cells' heterogeneity, to be used for clustering. *seuratBootstrap* function implements data reduction and clustering. Specifically, cells undergo to global scaling normalization, i.e. LogNormalize method, and scaling factor 10000. Subsequently, a linear dimensional reduction is done using the range of principal components defined with *seuratPCAEval*. Then, clustering is performed using the cell PCA scores. The Seurat clustering procedure, embedded in *seuratBootstrap,* is based on the Louvain modularity optimization algorithm. Differently SIMLR implements a k-mean clustering, where the number of clusters (i.e. k) is taken as input. SIMLR, requires as input raw counts $\log_{10}$ transformed. SIMLR is capable of learning an appropriate cell-to-cell similarity metric from the input single-cell data and to exploit it for the clustering task. In the learning phase SIMLR identifies a distance metric that best fits the structure of the data by combining multiple Gaussian kernels [15]. Thus, the tool can deal with the large noise and drop-out effects of single-cell data, which could not easily fit with specific statistical assumptions made by standard dimension reduction algorithms [15]. The function *simlrBootstrap* controls the clustering procedure and the function *nClusterEvaluationSIMLR*, a wrapper for the R package griph (Graph Inference of Population Heterogeneity) [28], is exploited to estimate the (sub)optimal number "k" of clusters. Griph clustering is based on Louvain modularity optimization algorithm.

We developed, for both Seurat and SIMLR, a procedure to measure the cluster quality on the basis of data structure. The rationale of our approach is that cells belonging to a specific cluster should be little affected by changes in the numerosity of the dataset, e.g. removal of 10% of the total number of cells used for clustering. Thus, we developed a metrics called CSS (Cell Stability Score), which describes the persistence of a cell in a specific cluster upon jackknifing and therefore offers a peculiar way of describing cluster stability. Detailed description of CSS metrics is available in Supplementary

file at section 5.1. CSS is embedded in *seuratBootstrap* and *simlrBootstrap*, and it is also used in *nClusterEvaluationSIMLR* to identify which number of clusters gives the best CSS behavior.

Feature selection

To select the most important features of each cluster we implemented in the *anovaLike* function the edgeR ANOVA-like method for single cells [20] and in the functions *seuratPrior* and *genesPrioritization/genesSelection* respectively the Seurat and SIMLR genes prioritization methods. *hfc* function allows the visualization of the genes prioritized with the above methods as heatmap and provides plots of prioritized genes in each single cell (Figure 6).

Scalability

To estimate the scalability of rCASC clustering we used the GSE106264 dataset made of 10035 cells and published by Pace and coworkers in 2018 [21]. We randomly sampled the 10035 cells (27998 ENSEMBL GENE IDs) to obtain the following subsets of cells: 400, 600, 800, 1000, 2000, 5000. Starting from the 800 cells set we randomly sampled the genes: 10000, 8000, 6000, 4000, 2000, 1000, 800. We run SIMLR, tSne, griph and Seurat using 160 permutation within SeqBox hardware [7]: Intel i7 3.5GHz (4 cores), 32 GB RAM and 500 GB SSD disk. SIMLR resulted to be the slowest and, given the above hardware implementation, it cannot allocate for the analysis more than 2000 cells (Figure 7A). All the other tools were able to handle up to 5000 cells within the limit of 32 GB of RAM available in the hardware setting used in this analysis. Computation time was nearly linear for all tools till 1000 cells. Only griph clustering resulted to be nearly insensitive to the increasing number of cells (Figure 7A). The computing time as function of increasing number of genes has a quite limited effect on the overall computing time (Figure 7B).

Availability and requirements

Project name: rCASC: reproducible Classification Analysis of Single Cell sequencing data

Project home page: https://github.com/kendomaniac/rCASC; https://github.com/mbeccuti/4SeqGUI

Operating system: Linux

Programming language: R and JAVA

Other Requirements: None

License: The GNU Lesser General Public License, version 3.0 (LGPL-3.0)

Any restrictions to use by non-academics: None

Authors' contributions

LA and FC equally participated to write R scripts, to create the majority of docker images, to package the workflow and release code. MB wrote the Java and C++ code, and acted as corresponding author. MA and MO prepared the single-cell data to be used as examples of the workflow functionality. GR prepared the dockers for fastq to counts table conversion. SR revised all packages and generated the docker files for docker images maintenance and further development. GDL gave scientific advices and provided an unpublished dataset for MAIT resting and activated T-cells (generated with Fluidigm C1 platform) to investigate genes detection limits in 3'end sequencing technologies and whole transcript sequencing. RAC and LP equally oversaw the project and gave scientific advices. All authors read, contributed and approved the final manuscript.

Supplementary material

rCASC_supplementary_file.pdf

References

1. Allison DB, Shiffrin RM, Stodden V: **Reproducibility of research: Issues and proposed remedies**. *Proceedings of the National Academy of Sciences of the United States of America* 2018, **115**(11):2561-2562.
2. **Challenges in irreproducible research** [https://www.nature.com/collections/prbfkwmwvz]
3. **Reproducibility in Computational Biology** [http://www.global-engage.com/life-science/reproducibility-computational-biology/]
4. Buettner F, Natarajan KN, Casale FP, Proserpio V, Scialdone A, Theis FJ, Teichmann SA, Marioni JC, Stegle O: **Computational analysis of cell-to-cell heterogeneity in single-cell RNA-sequencing data reveals hidden subpopulations of cells**. *Nature biotechnology* 2015, **33**(2):155-160.
5. Sandve GK, Nekrutenko A, Taylor J, Hovig E: **Ten simple rules for reproducible computational research**. *PLoS computational biology* 2013, **9**(10):e1003285.
6. Kulkarni N, Alessandri L, Panero R, Arigoni M, Olivero M, Ferrero G, Cordero F, Beccuti M, Calogero RA: **Reproducible bioinformatics project: a community for reproducible bioinformatics analysis pipelines**. *BMC bioinformatics* 2018, **19**(Suppl 10):349.
7. Beccuti M, Cordero F, Arigoni M, Panero R, Amparore EG, Donatelli S, Calogero RA: **SeqBox: RNAseq/ChIPseq reproducible analysis on a consumer game computer**. *Bioinformatics* 2017.
8. **rCASC R Package** [https://github.com/kendomaniac/rCASC]
9. **4SeqGUI** [https://github.com/mbeccuti/4SeqGUI]
10. Lun AT, McCarthy DJ, Marioni JC: **A step-by-step workflow for low-level analysis of single-cell RNA-seq data with Bioconductor**. *F1000Res* 2016, **5**:2122.
11. Zhu X, Wolfgruber TK, Tasato A, Arisdakessian C, Garmire DG, Garmire LX: **Granatum: a graphical single-cell RNA-Seq analysis pipeline for genomics scientists**. *Genome medicine* 2017, **9**(1):108.
12. Diaz A, Liu SJ, Sandoval C, Pollen A, Nowakowski TJ, Lim DA, Kriegstein A: **SCell: integrated analysis of single-cell RNA-seq data**. *Bioinformatics* 2016, **32**(14):2219-2220.

13. Butler A, Hoffman P, Smibert P, Papalexi E, Satija R: **Integrating single-cell transcriptomic data across different conditions, technologies, and species**. *Nature biotechnology* 2018, **36**(5):411-420.
14. Bacher R, Chu LF, Leng N, Gasch AP, Thomson JA, Stewart RM, Newton M, Kendziorski C: **SCnorm: robust normalization of single-cell RNA-seq data**. *Nature methods* 2017, **14**(6):584-586.
15. Wang B, Zhu J, Pierson E, Ramazzotti D, Batzoglou S: **Visualization and analysis of single-cell RNA-seq data by kernel-based similarity learning**. *Nature methods* 2017, **14**(4):414-416.
16. Freytag S, Tian L, Lonnstedt I, Ng M, Bahlo M: **Comparison of clustering tools in R for medium-sized 10x Genomics single-cell RNA-sequencing data**. *F1000Res* 2018, **7**:1297.
17. von Luxburg U: **Clustering Stability: An Overview**: Now publishers inc; 2010.
18. Hennig C: **Cluster-wise assessment of cluster stability**, vol. 52: Elsevier; 2007.
19. **fpc R package** [https://cran.r-project.org/web/packages/fpc/index.html]
20. Robinson MD, McCarthy DJ, Smyth GK: **edgeR: a Bioconductor package for differential expression analysis of digital gene expression data**. *Bioinformatics* 2010, **26**(1):139-140.
21. Pace L, Goudot C, Zueva E, Gueguen P, Burgdorf N, Waterfall JJ, Quivy J-P, Almouzni G, Amigorena S: **The epigenetic control of stemness in CD8+ T cell fate commitment**. *Science (New York, N Y)* 2018, **359**(6372):177-186.
22. Klein AM, Mazutis L, Akartuna I, Tallapragada N, Veres A, Li V, Peshkin L, Weitz DA, Kirschner MW: **Droplet barcoding for single-cell transcriptomics applied to embryonic stem cells**. *Cell* 2015, **161**(5):1187-1201.
23. Zilionis R, Nainys J, Veres A, Savova V, Zemmour D, Klein AM, Mazutis L: **Single-cell barcoding and sequencing using droplet microfluidics**. *Nature protocols* 2017, **12**(1):44-73.
24. **indrops** [https://github.com/indrops/indrops]
25. AlJanahi AA, Danielsen M, Dunbar CE: **An Introduction to the Analysis of Single-Cell RNA-Sequencing Data**. *Mol Ther Methods Clin Dev* 2018, **10**:189-196.
26. Liu ZH, Lou HZ, Xie KK, Wang H, Chen N, Aparicio OM, Zhang MQ, Jiang R, Chen T: **Reconstructing cell cycle pseudo time-series via single-cell transcriptome data**. *Nature Communications* 2017, **8**.
27. Barron M, Li J: **Identifying and removing the cell-cycle effect from single-cell RNA-Sequencing data**. *Sci Rep* 2016, **6**:33892.
28. **griph: Graph Inference of Population Heterogeneity** [https://github.com/ppapasaikas/griph]
29. Chhangawala S, Rudy G, Mason CE, Rosenfeld JA: **The impact of read length on quantification of differentially expressed genes and splice junction detection**. *Genome biology* 2015, **16**:131.
30. Turman MA, Yabe T, McSherry C, Bach FH, Houchins JP: **Characterization of a novel gene (NKG7) on human chromosome 19 that is expressed in natural killer cells and T cells**. *Hum Immunol* 1993, **36**(1):34-40.

Figure legend

Figure 1: rCASC workflow. Blue boxes indicate preprocessing tools. Yellow boxes define clustering tools. Green box indicates genes-signatures tools.

Figure 2: Cell Stability Score versus Silhouette Score calculated on Pace's dataset (see Supplementary file section 8) using SIMLAR over a set of number of clusters ranging between 5 and 8. A) Cell Stability Score violin plot. Looking at the mean value and data dispersion the best number of clusters is 5, indicating the with 5 clusters cells remain in the same cluster more about 80% of the times a random removal of 10% of the cell is applied to the full dataset. B) Silhouette Score violin plot. Looking at the mean value of the SS distribution there are no clear evidences that one clusterization is better that another. Furthermore, the dispersion of the SS value is getting narrow as the number of the clusters increases.

Figure 3: Comparison between the analysis features available in rCASC and in other single-cell analysis workflows.

Figure 4: rCASC graphical interface within 4seqGUI. A) Counts table generation menu: this set of function is devoted to the conversion of fastq to a counts table. B) Counts table manipulation menu: this set of functions allows inspection, filtering and normalization of the counts table. C) Clustering menu: these functions allow the use of SIMLR, tSne and Seurat to group cells in subpopulations. D) Feature selection menu: this set of functions allow the identification of cluster-specific subsets of genes and their visualization using heatmaps.

Figure 5: *genesUmi* plots the number of detectable genes in each cell (a cell is called present if supported by a user defined N number of UMI/reads, suggested values N=3 for UMI or N=5 for smart-seq sequencing [29]) with respect to the number of sequences UMI/reads. *mitoRiboUmi* calculates the percentage of mitochondrial/ribosomal genes with respect to the total number of detected genes in each cell and plots % of mitochondrial genes with respect to % of ribosomal genes. Furthermore, cells are colored on the basis of the number of detected genes: A) *genesUmi* plot for resting CD8+ T-cells [21], sequencing average 83,000 reads/cell. B) *mitoRiboUmi* plot for resting CD8+ T-cells [21]. It is notable that cells aggregated in two groups: the majority of the cells with less than 100 detected genes groups together and they are characterized by high relative percentage of mitochondrial genes and low relative percentage of ribosomal genes. Remaining cells are

characterized by few detectable genes, 100÷250 genes/cell, with a percentage of ribosomal genes greater than 30%. C) *genesUmi* plot for Listeria activated CD8+ T-cells [21], sequencing average 83,000 reads/cell, it is notable the activated cells show a wider range of detectable genes. D) *mitoRiboUmi* plot for Listeria activated CD8+ T-cells [21]. The majority of the cells are characterized by more the 100 genes called present and they show low percentage of mitochondrial genes and percentage of ribosomal genes between 15 to 35%. The remaining cells, with less than 100 detected genes groups together and are characterized by high relative percentage of mitochondrial genes and low relative percentage of ribosomal genes.

Figure 6: Heat map and cell expression plot for prioritized genes. A) Heat map for the set of 577 genes selected for Pace datasets (see Supplementary file section 8) by SIMLR prioritization. B) Nkg7 CPM expression in the cell clusters. Nkg7 is expressed in activated T-cells (clusters 1, 2, 4, 5) [30] but not in resting T-cells (cluster 3).

Figure 7: Scalability analysis of clustering tools implemented in rCASC. A) Time required to perform 160 permutations as function of increasing number of cells on a set of 27998 genes. B) Time required to perform 160 permutations as function of increasing number of genes on a set of 800 cells.

Figure 1

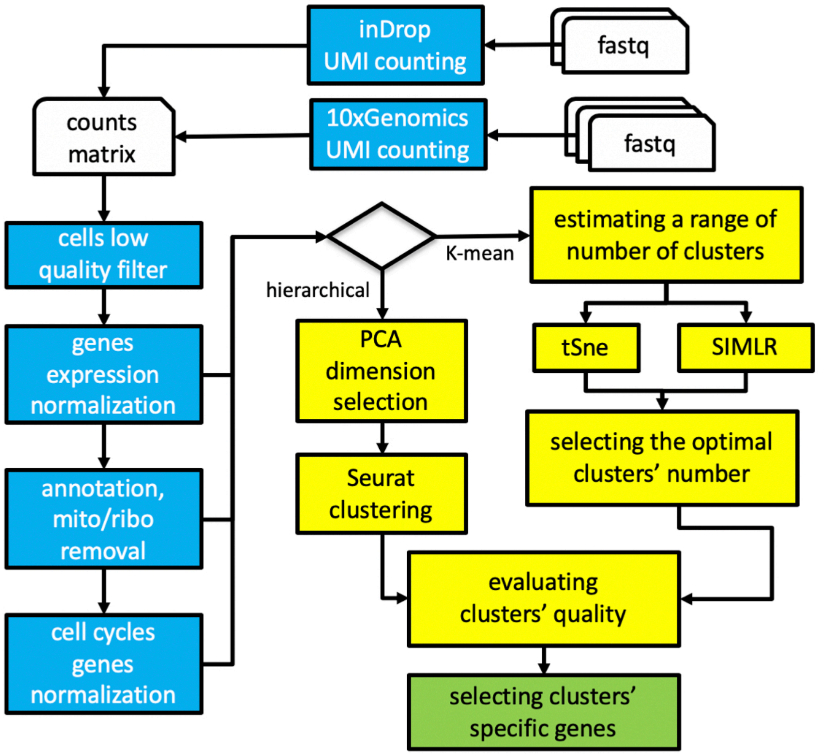
inDrop UMI counting
fastq
counts matrix
10xGenomics UMI counting
fastq
cells low quality filter
genes expression normalization
annotation, mito/ribo removal
cell cycles genes normalization
K-mean
hierarchical
estimating a range of number of clusters
PCA dimension selection
tSne
SIMLR
selecting the optimal clusters' number
Seurat clustering
evaluating clusters' quality
selecting clusters' specific genes

Figure 2

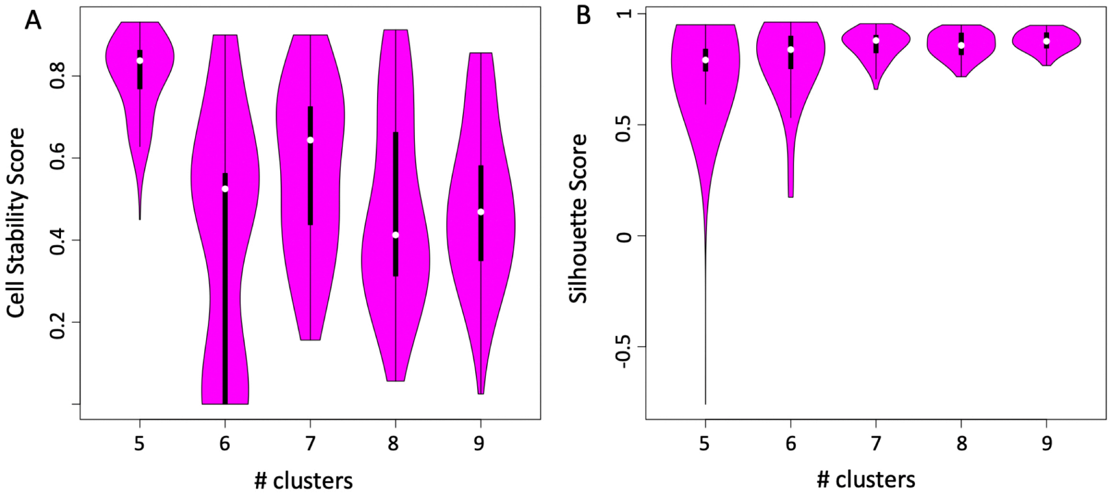

A
Cell Stability Score
0.2
0.4
0.6
0.8
5
6
7
8
9
clusters
B
Silhouette Score
-0.5
0
0.5
1
5
6
7
8
9
clusters

Figure 3

		rCASC (stand alone)	simpleSingleCell (stand alone)	Granatum (web)	Scell (stand alone)	Seurat (stand alone)
**Platforms**		10Xgenomics, inDrop, counts table	counts table	counts table	counts table	counts table
**Tools**	*Fastq conversion in counts table*	Y	-	-	-	-
	Quality Control / Outlier Filtering	Y	-	Y	Y	Y
	Annotation	ENSEMBL ID -> Gene Symbol	-	-	-	-
	Genes filter	Y	-	Y	Y	Y
	Data normalization	Y	Y	Y	Y	Y
	Cell cycle bias removal	Y	-	-	Y	Y
	Data dimensionality reduction	Y	Y	Y	Y	Y
	Supported clustering methods	tSne, SIMLR, Seurat PCA	Walktrap	Non-negative matrix factorization, K-mean (Euclidean), K-mean (tSne)	PCA, k-means, Gaussian mixture, Minkowski weighted k-means, DBSCAN	PCA, tSne, ica, dmap
	Cluster quality score	Silhuette, Cell Stability Score	-	-	-	-
	Features selection and visualization	Y	Y	Y	-	-
	Supported methods	ANOVA-like (edgeR), SIMLR Seurat genes prioritization	filtering on expression	NODES, SCDE, EdgeR, Limma	-	wilcox, bimod, roc, t-test, tobit, negbinom, MAST, DESeq2
	Biological inference	-	-	Y	-	-
**Reproducibility**	*Functional reproducibility*	Y	Y	-	-	Y
	Computational reproducibility	Y	-	Y	Y	-
**Flexibility**	*line command execution*	Y	Y	-	-	Y
	graphical interface	Y	-	Y	Y	-

Figure 4

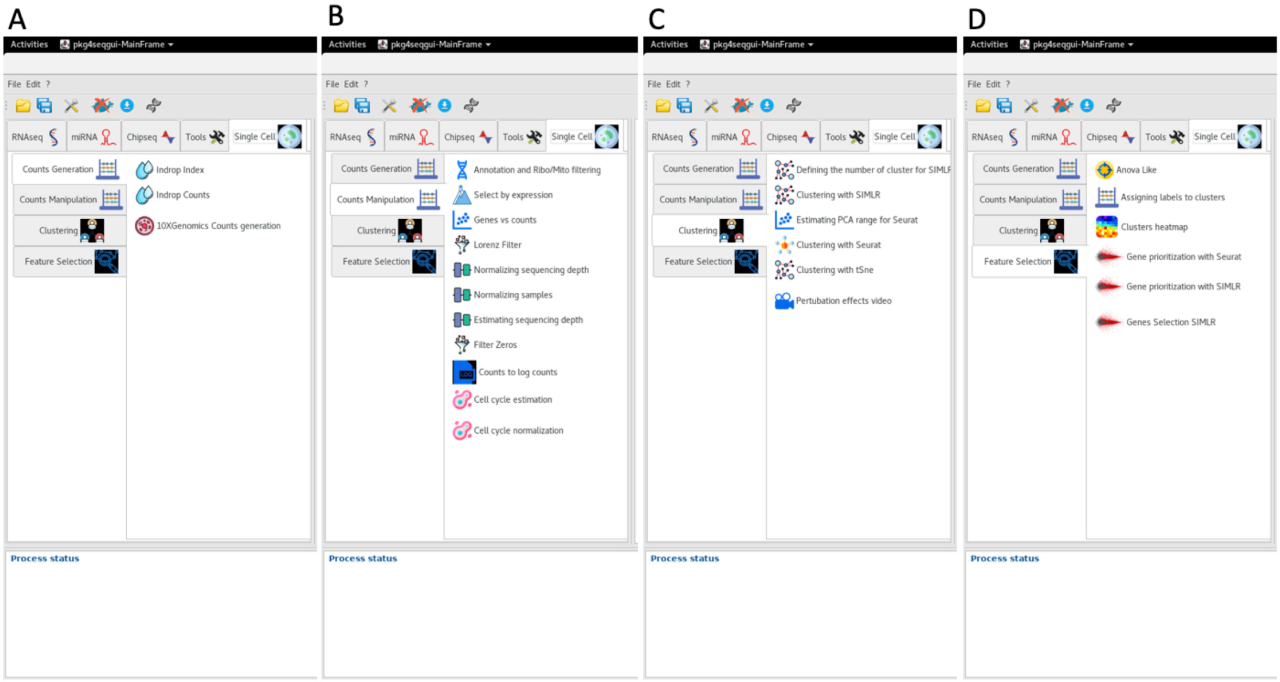
A
B
C
D
Activities
pkg4seqgui-MainFrame
File Edit ?
RNAseq
miRNA
Chipseq
Tools
Single Cell
Counts Generation
Counts Manipulation
Clustering
Feature Selection
Indrop Index
Indrop Counts
10XGenomics Counts generation
Annotation and Ribo/Mito filtering
Select by expression
Genes vs counts
Lorenz Filter
Normalizing sequencing depth
Normalizing samples
Estimating sequencing depth
Filter Zeros
Counts to log counts
Cell cycle estimation
Cell cycle normalization
Defining the number of cluster for SIMLR
Clustering with SIMLR
Estimating PCA range for Seurat
Clustering with Seurat
Clustering with tSne
Pertubation effects video
Anova Like
Assigning labels to clusters
Clusters heatmap
Gene prioritization with Seurat
Gene prioritization with SIMLR
Genes Selection SIMLR
Process status

Figure 5

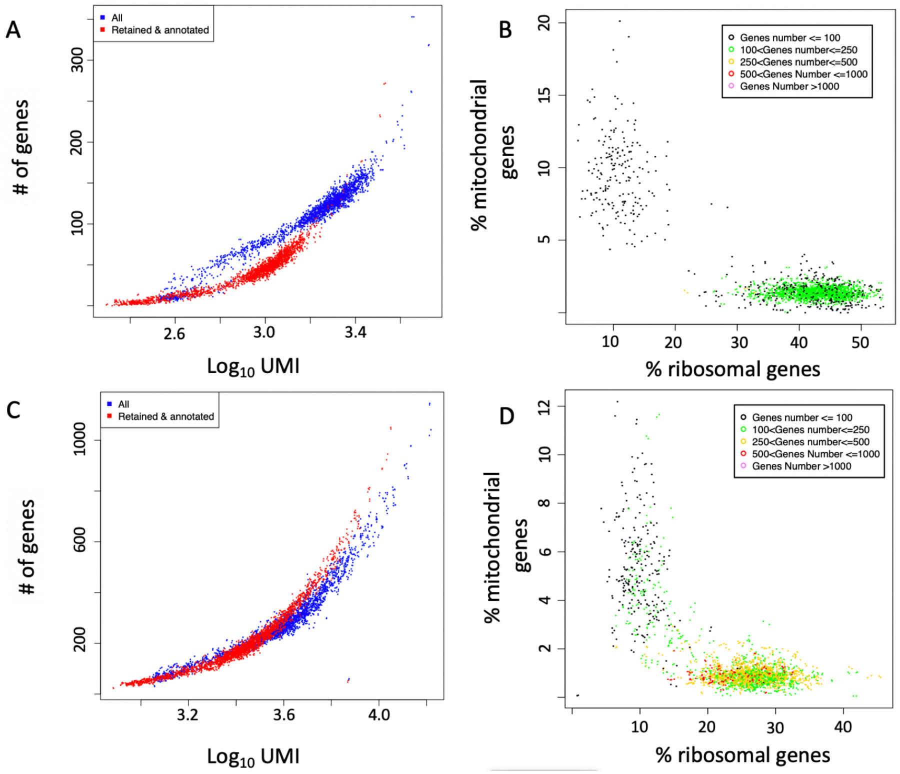
A
All
Retained & annotated
# of genes
100
200
300
2.6
3.0
3.4
Log10 UMI
B
Genes number <= 100
100<Genes number<=250
250<Genes number<=500
500<Genes Number <=1000
Genes Number >1000
% mitochondrial genes
5
10
15
20
10
20
30
40
50
% ribosomal genes
C
All
Retained & annotated
# of genes
200
600
1000
3.2
3.6
4.0
Log10 UMI
D
Genes number <= 100
100<Genes number<=250
250<Genes number<=500
500<Genes Number <=1000
Genes Number >1000
% mitochondrial genes
2
4
6
8
10
12
10
20
30
40
% ribosomal genes

Figure 6

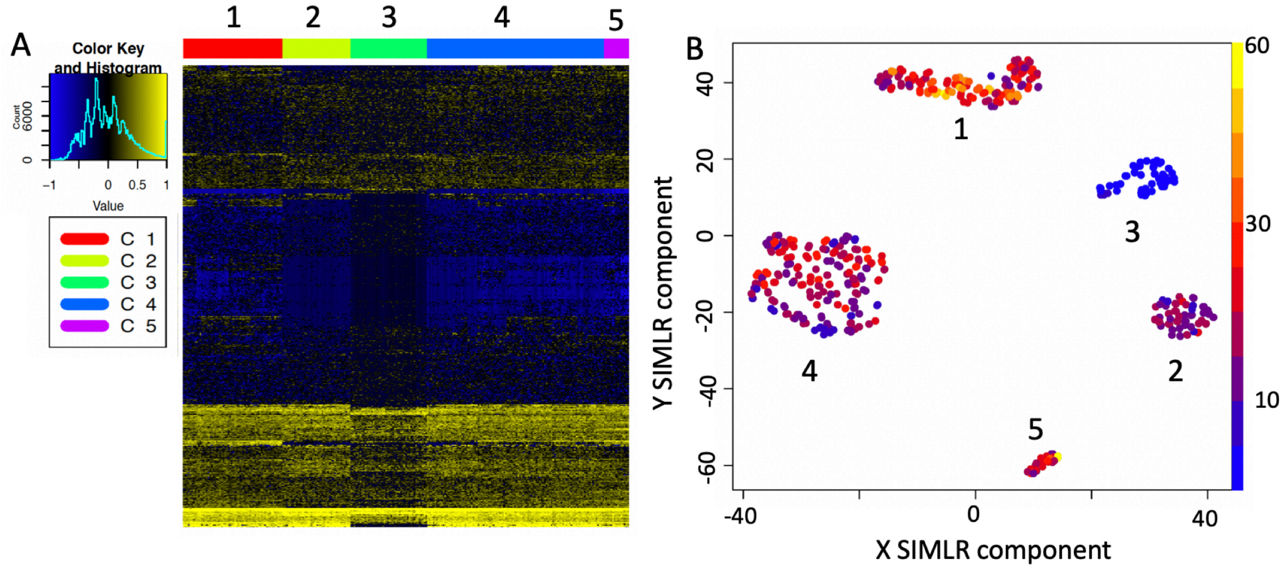

A
Color Key and Histogram
Count
6000
0
−1
0
0.5
1
Value
C 1
C 2
C 3
C 4
C 5
1
2
3
4
5
B
Y SIMLR component
X SIMLR component
40
20
0
-20
-40
-60
-40
0
40
60
30
10

Figure 7

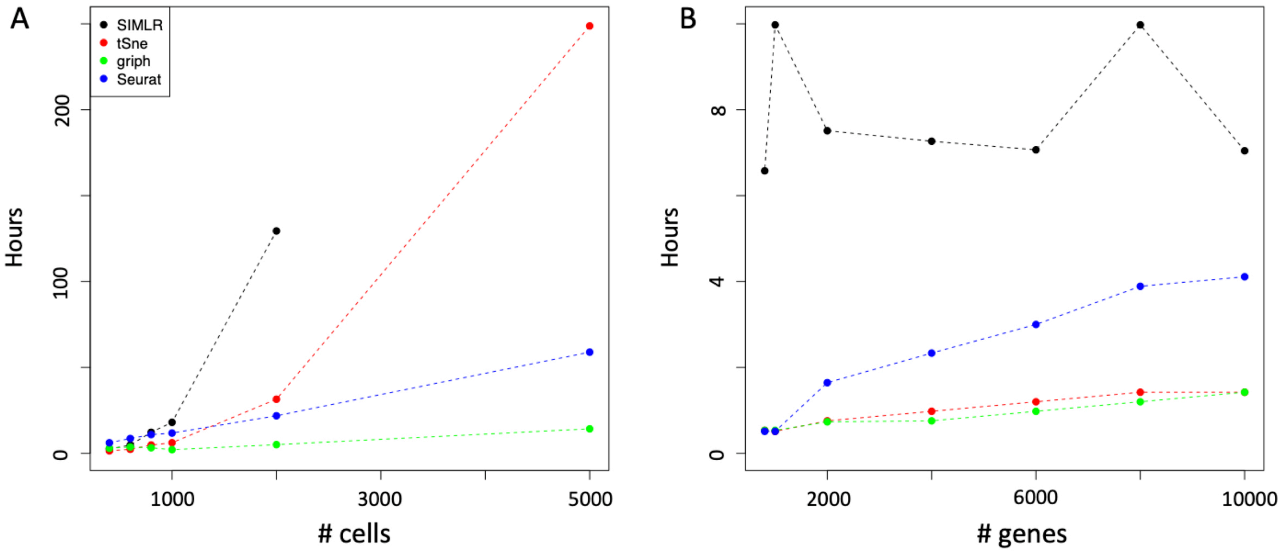
A
SIMLR
tSne
griph
Seurat
Hours
0
100
200
1000
3000
5000
cells
B
Hours
0
4
8
2000
6000
10000
genes

Click here to access/download;Figure;AllFigures.pdf

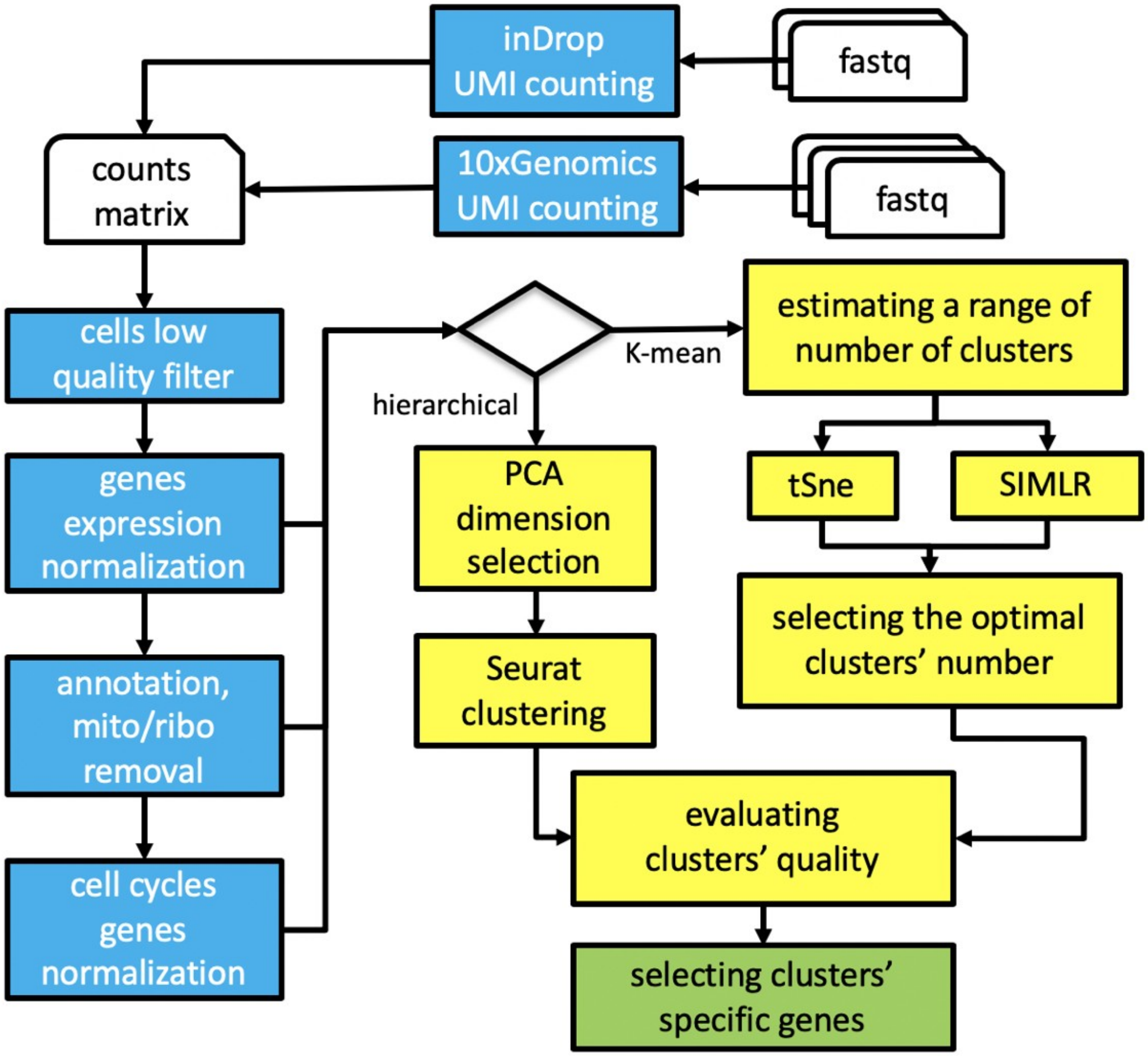

Fig.1

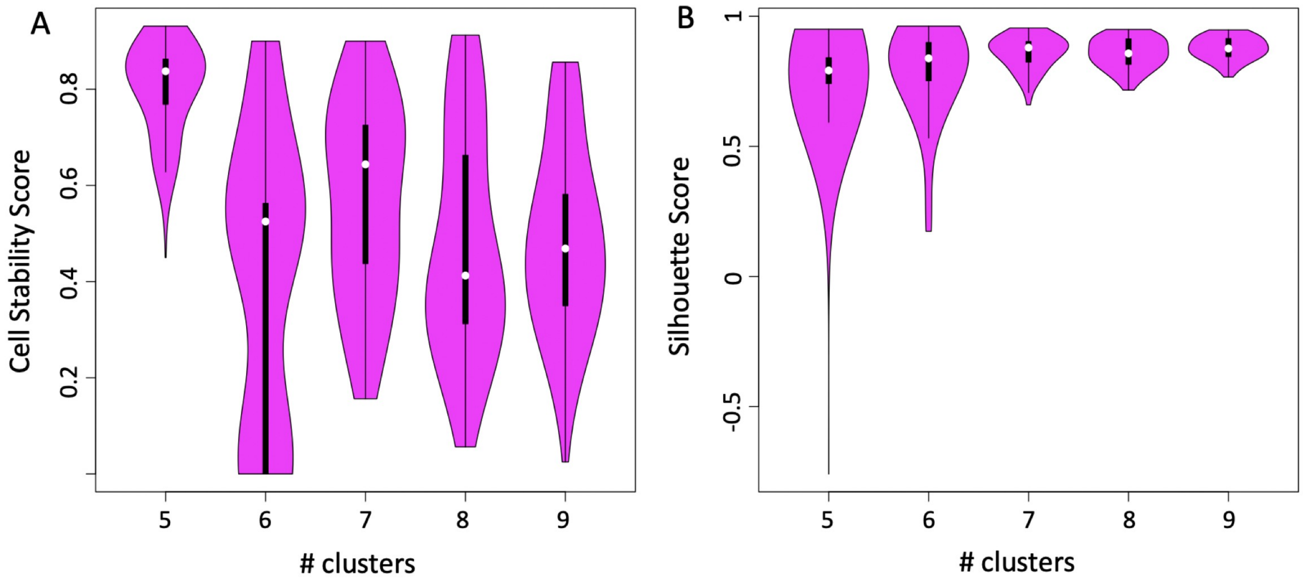

Fig.2

		rCASC (stand alone)	simpleSingleCell (stand alone)	Granatum (web)	Scell (stand alone)	Seurat (stand alone)
**Platforms**		10Xgenomics, inDrop, counts table	counts table	counts table	counts table	counts table
**Tools**	*Fastq conversion in counts table*	Y	-	-	-	-
	Quality Control / Outlier Filtering	Y	-	Y	Y	Y
	Annotation	ENSEMBL ID -> Gene Symbol	-	-	-	-
	Genes filter	Y	-	Y	Y	Y
	Data normalization	Y	Y	Y	Y	Y
	Cell cycle bias removal	Y	-	-	Y	Y
	Data dimensionality reduction	Y	Y	Y	Y	Y
	Supported clustering methods	tSne, SIMLR, Seurat PCA	Walktrap	Non-negative matrix factorization, K-mean (Euclidean), K-mean (tSne)	PCA, k-means, Gaussian mixture, Minkowski weighted k-means, DBSCAN	PCA, tSne, ica, dmap
	Cluster quality score	Silhuette, Cell Stability Score	-	-	-	-
	Features selection and visualization	Y	Y	Y	-	-
	Supported methods	ANOVA-like (edgeR), SIMLR Seurat genes prioritization	filtering on expression	NODES, SCDE, EdgeR, Limma	-	wilcox, bimod, roc, t-test, tobit, negbinom, MAST, DESeq2
	Biological inference	-	-	Y	-	-
**Reproducibility**	*Functional reproducibility*	Y	Y	-	-	Y
	Computational reproducibility	Y	-	Y	Y	-
**Flexibility**	*line command execution*	Y	Y	-	-	Y
	graphical interface	Y	-	Y	Y	-

Fig.3

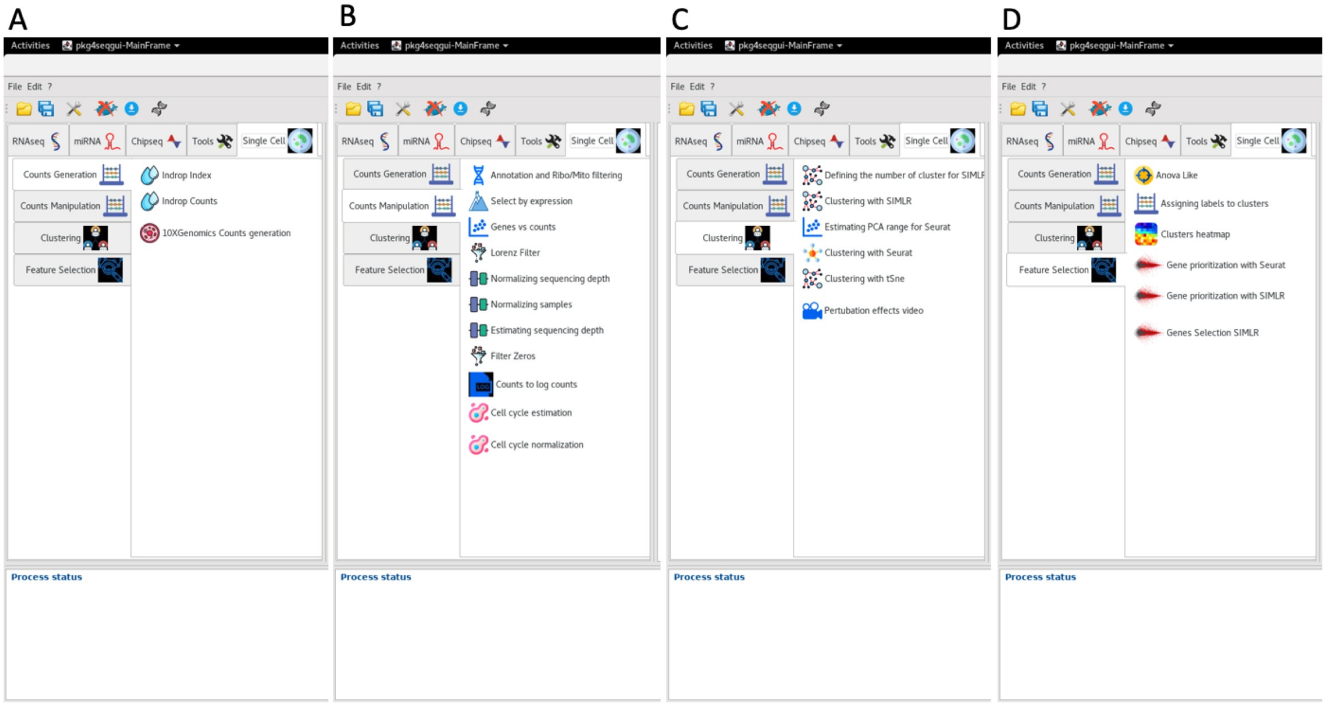
A
B
C
D
Activities
pkg4seqgui-MainFrame
File Edit ?
RNAseq
miRNA
Chipseq
Tools
Single Cell
Counts Generation
Counts Manipulation
Clustering
Feature Selection
Indrop Index
Indrop Counts
10XGenomics Counts generation
Annotation and Ribo/Mito filtering
Select by expression
Genes vs counts
Lorenz Filter
Normalizing sequencing depth
Normalizing samples
Estimating sequencing depth
Filter Zeros
Counts to log counts
Cell cycle estimation
Cell cycle normalization
Defining the number of cluster for SIMLR
Clustering with SIMLR
Estimating PCA range for Seurat
Clustering with Seurat
Clustering with tSne
Pertubation effects video
Anova Like
Assigning labels to clusters
Clusters heatmap
Gene prioritization with Seurat
Gene prioritization with SIMLR
Genes Selection SIMLR
Process status

Fig.4

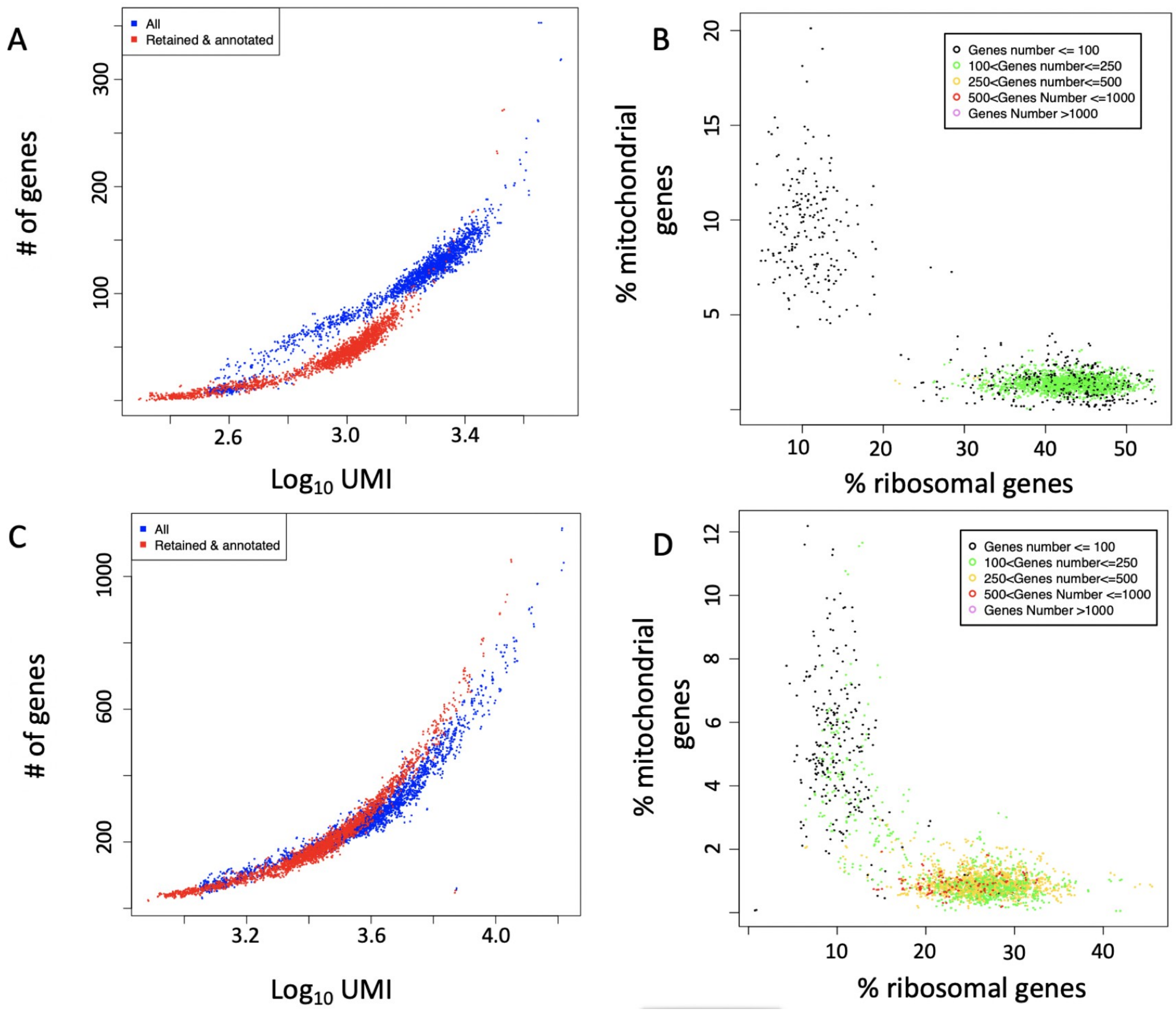
A
B
C
D
All
Retained & annotated
# of genes
Log10 UMI
% mitochondrial genes
% ribosomal genes
Genes number <= 100
100<Genes number<=250
250<Genes number<=500
500<Genes Number <=1000
Genes Number >1000

Fig.5

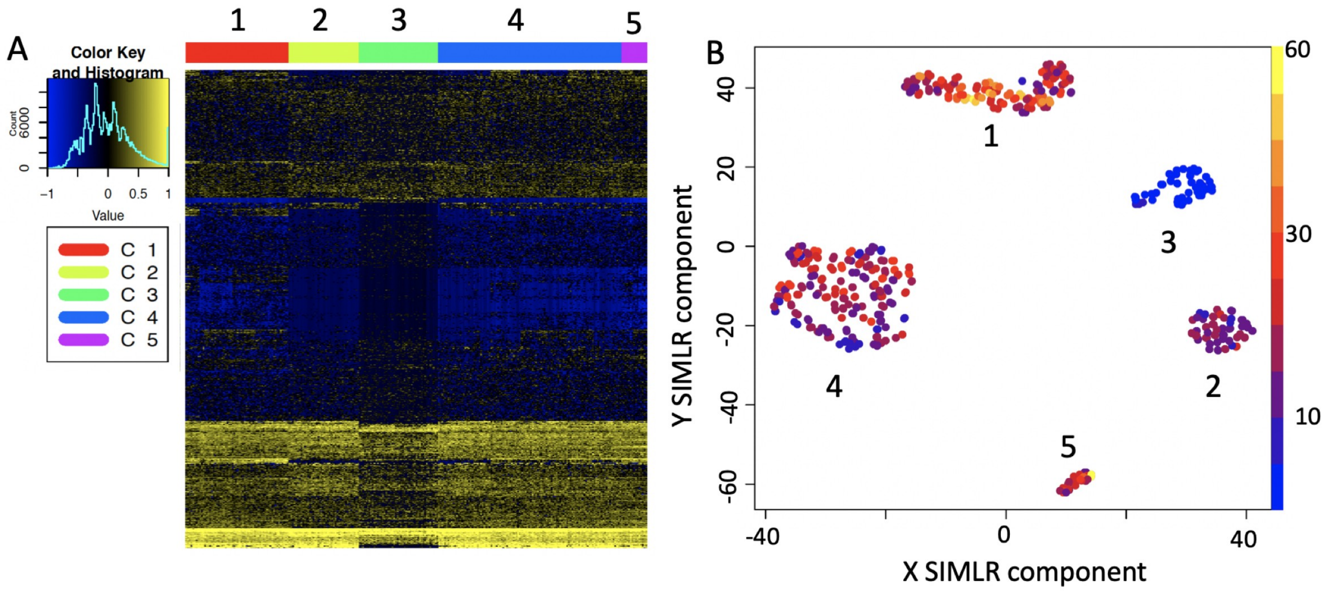
A
Color Key and Histogram
Count
0
6000
−1
0
0.5
1
Value
C 1
C 2
C 3
C 4
C 5
1
2
3
4
5
B
Y SIMLR component
X SIMLR component
40
20
0
-20
-40
-60
-40
0
40
60
30
10
1
2
3
4
5

Fig.6

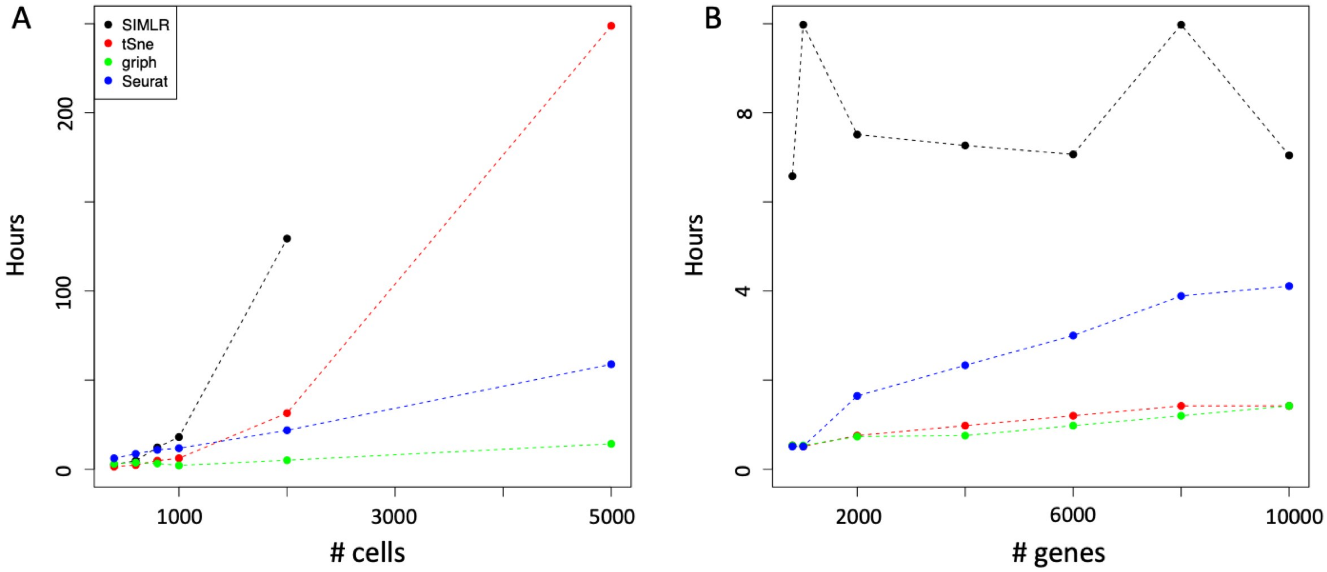
A
SIMLR
tSne
griph
Seurat
Hours
0
100
200
1000
3000
5000
cells
B
Hours
0
4
8
2000
6000
10000
genes

Fig.7

Supplementary material

Click here to access/download
Supplementary Material
rCASC_supplementary_file.pdf

Rebuttal

Click here to access/download;Personal Cover;GIGA_rebuttal_craMBFCcra1a.pdf

GIGA-D-18-00522

Dear Editor,

First of all, we wish to thank the reviewers for their valuable comments and useful suggestions which helped us to improve the paper and the tool.

Editor request: Please register any new software application in the SciCrunch.org database to receive a RRID (Research Resource Identification Initiative ID) number, and include this in your manuscript. This will facilitate tracking, reproducibility and re-use of your tool.
Answer: we registered rCASC to SciCrunch and it is now associated with the Research Resource Identification Initiative ID : SCR_017005

Reviewer #1:
Comments to the Author:
This paper presents a pipeline to infer single-cell clusters using scRNA-Seq data (rCASC), infer significant features linked to each cluster, and can analyse various metrics during the processing. Notably, the results of the pipeline can be divided into 3 major outputs, A) cells-features matrix generation, B) Clustering, and C) inference of significant features per clusters. Also, the pipeline is able to perform various additional substeps such as Matrix preprocessing (normalization), outliers removal, features removal, cell cycle specific features removal. The pipeline is implemented in R using Docker containers and has a GUI interface coded in Java. Finally; the authors claimed have invented a metric: the CSS, to evaluate cluster stability in their single-cell analyses. First, It is a pleasant surprise to be able to install everything needed to perform scRNA-Seq analysis with few simple commands (with exception of Docker which can be tricky for non IT people). Also, developing scRNA-Seq analytical toolbox easy to use and efficient are an innovative direction due to the importance and the multidisciplinary aspect of the field. However, I have major concerns which I think should be addressed before publication.

Major Comments:
Comment 1: First, the abstract and the text contain different confusing aspects that must be rewritten. The authors describe a "supervised approach": SIMLR which is seen as the alternative of the "Seurat clustering". From my knowledge, SIMLR is a clustering workflow and thus is also an unsupervised approach, by contrast with any other supervised approaches using training datasets as input (classification/regression…). I don't know what is a "supervised clustering" if not a classification procedure. Clustering are always unsupervised with the exception of "semi-supervised" clustering (use of seed samples).
Answer 1: As pointed out by the reviewer both Seurat and SIMLR use an unsupervised approach, and they mainly differ in the metrics driving the clustering analysis. ***SIMLR*** is capable of learning an appropriate cell-to-cell similarity metric from the input single-cell data and to exploit it for the clustering task. In details, the learning phase identifies a distance metric that best fits the structure of the data by combining multiple Gaussian kernels. This allows the tool to deal with the large noise and drop-out effect of single-cell data that could not be easily fitted with specific statistical assumptions made by standard dimension reduction algorithms. Differently, **Seurat** clustering algorithm is based on the Euclidean distance in PCA space, and refines the edge weights between any two cells based on the shared overlap in their local neighbourhoods (Jaccard similarity). Since these two clustering approaches have their specific criticality and strengths we decided to integrate

both of them in our framework. Finally, according to the reviewer' comments we modified the following sentences:
In Abstract-Findings: "Subpopulations discovery can be instead achieved using unsupervised and supervised clustering technique"
was modified in
"Subpopulations discovery can be instead achieved using different clustering techniques based on different distance metrics".
In keywords: "supervised clustering, unsupervised clustering"
was modified in
"clustering"
In Findings: "Therefore, rCASC provides raw data preprocessing, subpopulation discovery via supervised/unsupervised clustering and cluster-specific genes-signatures detection"
was modified in
"Therefore, rCASC provides raw data preprocessing, subpopulation discovery via different clustering approaches and cluster-specific genes-signatures detection"
"rCASC implements as unsupervised clustering tool and as supervised clustering tools".
was modified in
"rCASC integrates two clustering tools, namely Seurat [13] and SIMLR [15], which mainly differ in the metrics driving the clustering analysis".
Comment 2: Also, I don't understand why this package is superior in term of "Computational and Functional" reproducibility compared with any other packages for which a similar reasoning can be also applied.
Answer 2: We would like to thank the reviewer to give us the possibility to explain better this aspect. rCASC is one of the tools developed under the umbrella of the Reproducible Bioinformatics project (http://www.reproducible-bioinformatics.org/), an open-source community aimed to provide to biologists and medical scientists, without scripting skills, a easy to use framework, which also guarantees the ability to reproduce results independently by the underlying hardware, using docker containerization (computational reproducibility), and providing tools that fulfil the best practice rules for reproducible computational research, proposed in 2013 by Sandve [PLoS computational biology 2013, 9(10)] (functional reproducibility). The reproducible bioinformatics project was founded and it is maintained by the research team of Elixir node at University of Turin. The reproducible bioinformatics project was published on BMC Bioinformatics (Kulkarni et al. 2018). An example of stand-alone hardware/software infrastructure for bulk RNAseq, developed within the Reproducible Bioinformatics project, was described in Beccuti et al. (Bioinformatics 2017). Thus, to the best of our knowledge, rCASC is the only computational framework, which provides a complete computational reproducibility for integrated analysis of single cell data (from counts generation to cell subpopulation identification). Last but not least rCASC is listed within the tools developed by the Italian Elixir node (https://bio.tools/rCASC).
Finally, the following phrase in the Findings section: "In this context rCASC provides a modular workflow to address at the same time the problem of functional and computational reproducibility. rCASC provides single cell analysis functionalities within the reproducible rules described by Sandve [5]. rCASC is part of the Reproducible Bioinformatics Project [6], which is a project designed to provide to the biological community a reproducible and user-friendly bioinformatics ecosystem [8]."
was modified in:
"rCASC is one of the tools developed under the umbrella of the Reproducible Bioinformatics project (http://www.reproducible-bioinformatics.org/), an open-source community aimed to provide to biologists and medical scientists, without advance scripting skills, an easy to use framework, which also guarantees the ability to reproduce results independently by the underlying hardware, using

docker containerization (computational reproducibility). Indeed, it was developed following the best practice rules for reproducible computational research, proposed in 2013 by Sandve [PLoS computational biology 2013, 9(10)] (functional reproducibility). The reproducible bioinformatics project was founded and it is maintained by the research team of Elixir node at University of Turin. The reproducible bioinformatics project was published on BMC Bioinformatics (Kulkarni et al. 2018). An example of stand-alone hardware/software infrastructure for bulk RNAseq, developed within the Reproducible Bioinformatics project, was described in Beccuti et al. (Bioinformatics 2017). Thus, to the best of our knowledge, rCASC is the only computational framework, which provides both computational and functional reproducibility for an integrated analysis of single cell data, from counts generation to cell subpopulation identification. rCASC is also listed within the tools developed by the Italian Elixir node (https://bio.tools/rCASC)."

Comment 3: Then, the authors claimed to have invented a new metric: the "Cell-stability Score", which is based on the computation of a stability score by clustering multiple bootstrap sampling and computing the jaccard index. Clustering stability measurement is not new and previous works already described more formally the use of bootstrapping together with clustering and Jaccard index to estimate cluster stability (http://www.homepages.ucl.ac.uk/~ucakche/papers/clusta.pdf (2006), https://arxiv.org/abs/1503.0205). These example algorithms are not based on single-cell datasets (other stability approaches exist for single-cells), but since the approach described in the first paper is very similar, a more comprehensive bibliography of clustering stability should be present in the manuscript as well a rewriting of the CSS description/notion, highlighting the similarity with previous works.

Answer 3: As mentioned by the reviewer the cell stability score method implemented by us uses Jaccard index, which is also used in Hennig paper (http://www.homepages.ucl.ac.uk/~ucakche/papers/clusta.pdf 2006). However, between our approach and the one of Hennig's paper there is a substantial difference: in Hennig's paper the Jaccard index is used to evaluate the similarity between clusters and the calculated score provides an overall quality score for each of the clusters in toto. In our implementation the stability score is not related to the clusters, but it is specific for each cell. We believe that cell stability score allows us to have a more precise view of which are the cells affected by perturbations of the dataset structure.

To better explain this issue, we modify the paper section "rCASC: a single cell analysis workflow designed to provide data reproducibility.", adding the following phrases before the sentence: "To the best of our knowledge, rCASC is the only workflow performing clustering in presence of data perturbation …":

"Cluster stability is an important topic in Clustering (for a review see von Luxburg 2010). Stability measurement, taking advantage of bootstrapping, was also addressed by Hennig (2007). Specifically, Hennig uses Jaccard index to evaluate the overall stability of each cluster. In rCASC, we have implemented a cell stability score (CSS), which uses the Jaccard index to estimate the stability of each cell in each cluster. CSS provides an enhanced description of each cluster, since it allows the identification of subset of cells, in any cluster, which are particularly sensitive to perturbation of the overall dataset structure, i.e. cell bootstrapping. Moreover, the cluster stability measurement proposed by Henning was included in rCASC. Specifically, we have implemented the "clusterboot "function from the fpc R package (https://cran.r-project.org/web/packages/fpc/index.html), which allows the evaluation of the cluster stability using a personalized clustering function). So far in our knowledge, rCASC is the only single-cell analysis workflow performing clustering in presence of data perturbation …". We added the Section 5.3 in Supplementary file to describe this functionality.

Comment 4: In term of additional experiments, I think it would be interesting to have an idea of the ratio: number of CPUs/ RAM/ computational time according to: the number of cells / number of features (i.e.: matrix dimension), and read depth (linked to fastq size). More specifically, what are the limiting steps in term of computation? What are the steps the less expensive ? A new figure might be necessary to represent the contribution of each step in term of computation.
Answer 4: We thanks the reviewer for this comment. We added a new figure describing the effect of cells and number of genes on the computation efficacy of SIMLR, Seurat, tSne and griph (Figure 7). In the Methods section we also added the paragraph "Scalability", in which we discussed the computational performance of the used methods.
See also A9 major comments reviewer 2 and A6a to your Comment 6.
Comment 5: Ideally, a comparison with the other cited pipeline would be also interesting, but this amount of work might be out of scope of this study.
Answer 5: We agree with reviewer that this point is out of the scope of our manuscript.
Comment 6:
Q6a) I have some concerns with the choice of clustering algorithms used. Despite Seurat is well established in the community and SIMLR is also a well recognized algorithm, I am not sure if these algorithms can handle very large sparse datasets (i.e. more than 10K cells), that are becoming the new standard in the field. Notably, are these algorithms able to handle sparse data? SIMLR needs a specified K, thus inferring the best K requires to screen amongst an array of Ks and thus might be very time consuming. Would it exist better and simpler alternatives to handle very large and sparse datasets that might be included in rCASC?
A6a) To answer to the above comment we run a performance experiment increasing the cell size and varying the number of genes used in clustering experiment. Please see Answer A9 major comments reviewer 2. A new paragraph, scalability, was added in method section in the main manuscript. In Figure 7A of paragraph scalability, it is shown the computing time (Intel i7 3.5 GHx, 4 cores, 32 GB RAM, 500GB SSD) required to execute 160 permutations on a dataset varying from 200 to 5000 cells using SIMLR, tSne, griph and Seurat. Specifically, Seurat analysis with 5000 cells can be completed in 50 hours. Computing times can be significantly reduced if a high-end multi-cores server is used. We observe that when the number of considered cells overcomes 5000 units then all the clustering tool integrated in rCASC requires more than 32GB RAM.
SIMLR, tSne, griph and Seurat were all designed to work with sparse data.
We agree with reviewer that SIMLR is more computational demanding with respect to the other implemented tools, but it provides very good clustering performance as reported in SIMLR paper (Wang et al. 2017). SIMLR was also released in a version to handle large scale dataset, but we did not integrate it because, when we tested it, we observed that the sensitivity and specificity of the method were not better of those of tSne (not shown in this manuscript). Of course, we are constantly looking for new clustering methods, proposed in the literature, able to overcame the limitations of the current tools. The latest comparison between clustering methods (Duò et al. 2018 on F1000: https://www.ncbi.nlm.nih.gov/pmc/articles/PMC6134335/) indicates that the clustering algorithm implemented in Seurat toolkit is the most computationally time efficient.
Q6b) Using a clustering stability metric is I think a very good idea. Is it possible to get an average stability score per cluster to have an idea if a cluster is noisy or robust?
A6b) We thank the reviewer with this useful comment. We answer to this point at the above comment 3.
Q6c) Also, even a stable cluster according to a bootstrap experiment is not a guarantee of a "biologically" stable cluster, and can reflect a biased in the method used (for example, a dummy algorithm clustering cells according to their name will produce very stable but useless clusters).

A6c) According to one of the requests of reviewer 2, see A2b major comments reviewer 2, we collected extra data, which are indicating that cell stability score (CSS) inversely correlate with the cells' heterogeneity of clusters, see Section 5 Figure 41 of the supplementary information. Since, we observed that the clusters characterized by high CSS are mainly constituted of cells of the same type, this suggests a correlation with the biological characteristics of the cells.
Q6d) What is the use of griph (Graph Inference of Population Heterogeneity). Why not using the stability measure to estimate the best K?
A6d) Indeed, we use the clusters stability measure to estimate with K is the best choice. However, to provide a suggestion on the range of numbers of clusters to be investigated, we decided to use griph tool, first because it is based on Louvain modularity optimization algorithm, as the Seurat clustering method we have integrated in rCASC. Then, because its execution time is the best between the clustering tools we have implemented in rCASC, see Figure 7 in the main manuscript. We did not implement griph as complete clustering tool in rCASC, because it is not published, yet.
Q6e) The package requires a very large amount of memory to be able to install all the docker dependencies and I was not able to install it on my own computer (out of memory). Is there any way to propose "lighter" versions in order to be able to use it on a standard computer? Overall, I am not sure if all these different steps are always mandatory to obtain biologically meaningful single-cell clusters (Of course, they might be required in some specific cases), compared to more straightforward approaches (matrix creation -> embedding -> clustering).
A6e) Concerning the docker images storage in local computer, it is not necessary to execute the command downloadContainers(), which download all rCASC docker images. In this case, only the required images will be downloaded on the basis of the rCASC function in use. This option will require extra time to execute each new function, since the corresponding docker image needs to be downloaded. We have also added the option "mini" for downloadContainers(), which downloads only 17 dockers out 26 docker images and provides all basic functionalities to handle a single-cell counts table.
Concerning RAM requirements, we can execute all rCASC functions with 16 GB RAM on datasets up 1000 cells. The main limitation is given by the possibility of performing no more than one permutation at a time.
Minor Comments:
Q1) The supplementary files document very rigorously the software which is really pleasant.
Some figures are not very informative and might be combined together (For example figures 1, 3 and 4 And figures 2 and 6?).
A1) We thank reviewer for the suggestion, but we prefer to leave these figures separated to keep clear the analysis workflow structure.
Q2) Can you describe briefly what is the Seurat specific normalization?
A2) Seurat normalization step is the one suggested from the Seurat workflow. This information was added in supplementary file: "Before clustering data are normalized as suggested from the Seurat workflow using Seurat NormalizeData function, with LogNormalize as normalization.method and 10000 as scale.factor parameters."

Reviewer #2: The authors present rCASC, an integrated analysis framework for single-cell RNA sequencing data that combines a range of existing and novel computational tools. While some workflows for reproducible scRNA-Seq data analysis exist, the authors provide an analysis strategy using docker containers and a graphical user interface for reproducibility. rCASC is implemented as an R package and as graphical user interface, which allows bioinformaticians as well as biologists with little experience to perform statistical data analysis. However, there are some concerns about

the chosen analysis tools and the presentation of results that need to be addressed: Major comments

Q1) The description of the algorithms used is often not clear. In the main text (p.4 l.16-21), the authors mention normalization and clustering strategies taken from other tools. When other tools are used, it would be good to briefly describe the underlying algorithms (either in the main text or the methods section). For example, Seurat is a toolbox for data analysis and not a clustering tool and it has to be specified which clustering strategy is used. The authors should also make sure to properly cite the original publication of functions implemented in the rCASC toolbox (for example the reCAT function and the griph package).

A1) We added for each function described in section Methods a brief description of the implemented algorithm. We apologize for the error in citing reCAT, we fixed it. Concerning griph, we only provided the github repository, because developers told us that it will be published as part of a larger paper entitled "Self-organization and symmetry breaking in intestinal organoid development" accepted for publication in Nature. However, at the time of this rebuttal the paper is not out, yet. We will update its reference as soon as it will be available.

Q2a) The authors developed the Cell Stability Score based on iteratively clustering a sub-sampled dataset. This is a good approach and informative for cluster stability. It would however be good to visualize the stability scores for datasets subsampled to 20%, 30%, and even 50%.

A2a) We extended the analysis described in Figure 38 of the vignette removing 20%, 30% and 50% of the cells. The results were added as Figure 40 and commented in vignette text: "The effect of the perturbations induced in the clustering upon the removal of 10%, 20%, 30% and 50% of the data set was also investigated in SetA (annotated_bmsnkn_5x100cells.txt), Figure 40. We can observe that the overall cell stability score of each cell in each cluster is reduced increasing of the fraction of cells removed in each permutation. However, the reduction in CSS is not identical for all clusters. In Figure 40, it is clear that cluster 2, completely composed of NK cells, is the most stable cluster to the perturbations induced by increasing the number of removed cells. On the other side, cluster 4, mainly made by stem cells (92 cells), together with few B-cells (2 cells) and Monocytes (7 cells) is the least stable. Sorting by increasing CSS the cells in cluster 4, Figure 40D, all B-cells and Monocytes are found within the first 15 most unstable cells."

Q2b) Furthermore, it is crucial to link the CSS to the clustering ground truth with the underlying assumption that the true discovery rate for "stable" cells is larger than the one for cells with lower CSS.

A2b) We have investigated the clusters composition of two sets of cells described in section 4.2: SetA and SetC. SetA is composed by cells with different biological characteristics, i.e. (B) B-cells, (M) Monocytes, (S) Stem cells, (NK) Natural Killer cells, (N) Naive T-cells. SetC contains Monocytes, Natural Killer cells and with T-cells subpopulations, i.e. (C) Cytotoxic T-cells, (H) T-helper cells and Naive T-cell. The results are summarized in Figure 41 and commented in the vignette text: "We investigated clusters composition of two sets of cells described in section 4.2: SetA and SetC.

SetA composed by cells with different biological characteristics, i.e. (B) B-cells, (M) Monocytes, (S) Stem cells, (NK) Natural Killer cells, (N) Naive T-cells.

SetC contains Monocytes, Natural Killer cells and with T-cells subpopulations, i.e. (C) Cytotoxic T-cells, (H) T-helper cells and Naive T-cell, Figure 41. CSS provides indications on clusters cells heterogeneity. In Figure 41A and C, clusters characterized by high cell stability score are mainly constituted by one cell type. On the other hand, as CSS decrease, Figure 41B and D, the clusters become characterized by an increasing heterogeneity in the cell types composition. E.g. In Figure 41B and D, cluster 5 (violet) has a CSS between 75% to 100% and it is composed only by monocytes, cluster 2 (light green), which has a CSS between 50% and 75%, has a 11% contamination of T-helper cells. Cluster 3 (green), which has a CSS between 25 to 50%, is contaminated by 12% Cytotoxic T-

cells and 4% T-helper cells. Finally, clusters 1 and 4, characterized by CSS between 0% to 25%, are very heterogeneous incorporating 4 out of 5 cell types present in this dataset.”.

Q3) The authors should discuss why they chose to store e.g. normalized data and analysis results in .csv or .txt files rather than using slots of a S4 class (in sparse matrix format) commonly used in R data analysis.

A3) All tools embedded in rCASC use as input source a tab or a comma delimited file and only Seurat imports counts table file in an object of class Seurat. 10XGenomics distributes counts table file also in H5 (Hierarchical Data Format 5 File) format, Bioconductor rhdf5 package offers support for this type of files, but none of the tools implemented in rCASC is able handle the R data structure returned by the rhdf5 package. Since, as far as we know there is not a general agreement on the format of single cell count tables we prefer to wait till an agreement on standardized data format will become available.

Q4) In the vignette is it often not clear what the scale bars or colour coding means. The authors should expand figure legends, plots and axes with the necessary information to understand what is displayed.

A4) We carefully revised the figure legend to clarify colours and legend bars.

Q5) When performing dimensionality reduction it is important to i) correctly normalize the data and ii) indicate if the counts were log-transformed. These information are missing in some parts of the vignette. For example the authors use a wrapper function to perform PCA on page 17. It is not clear if the data was normalized and log-transformed which could have an impact on the interpretability of the results.

A5) We carefully revised the various part of the vignette and we added information referring to the data characteristics.

Q6) The scannobyGtf function performs gene annotation and removes mitochondrial genes and genes encoding ribosomal proteins. For some analyses, these genes can be informative regarding the metabolic and proliferative state of the cell and should not be removed. The authors should therefore consider splitting the scannobyGtf function into two functions; one for annotation and one for filtering. If users detect unwanted variation in the expression of genes encoding for ribosomal proteins, this effect should be removed using regression approaches such as scLVM rather than excluding the genes from the dataset.

A6) The scannobyGtf function was updated to make optional the removal of mitochondrial and ribosomal protein genes.

Q7) Section 3.5 Top expressed genes: By selecting the set of top expressed genes the authors might be biased by the variation detected in highly expressed housekeeping genes. Instead, and in line with commonly performed analysis for scRNA-Seq data, the authors should include an approach to detect highly variable genes as the set of informative genes. This also facilitates the inclusion of cell-type specific genes in the clustering approach (see paragraph on page 39 in the vignette).

A7) We thank reviewer for this useful comment. We have added the option to filter the dataset on the basis of gene dispersion in the topx function. Section 3.5: “For clustering purposes user might decide to use the top expressed genes. The function topx selects the X top expressed genes given a user defined threshold.”

was modified in:

“For clustering purposes user might decide to use the top expressed/variable genes. The function topx provides two options:

- the selection of the X top expressed genes given a user defined threshold, parameter type="expression"

-the selection of the X top variable genes given a user defined threshold, parameter type="variance"

The function also produces a pdf file gene_expression_distribution.pdf showing the changes in the UMIs/gene expression distribution upon topx filtering."

Q8) When toy datasets are used it is important to state if these are the data from the original publication or if the data were pre-processed (see bottom of page 29 in the vignette).

A8) We updated the description of the toy experiments

Q9) The authors implemented different clustering strategies in the rCASC toolbox. While the cell stability score is explained in detail, it is not clear what exactly is used for SIMLR and tSNE clustering. Both are methods to perform non-linear dimensionality reduction and only SIMLR is designed to also perform clustering. tSNE is not a clustering tool and it is well known that it introduces artefacts when visualizing complex data. It would therefore be good to explain if the SIMLR internal clustering approach is used or if the authors perform k-means clustering on the dimensionality reduced data-points. For reasons of scalability and the number of input genes, I wonder whether the SIMLR approach is suitable for large dataset (e.g. 50,000 cells).

A9) In vignette section 5 and 6 we described SIMLR and Seurat as the two clustering tools used in rCASC. The choice of these two tools is given by the comparisons performed by Wang and coworkers in their paper on SIMLR (Nat Methods. 2017) and by the independent observations of Freytag and coworkers (https://f1000research.com/articles/7-1297/v2) and Duo' and Robinson (https://f1000research.com/articles/7-1141/v2) indicating that, in their tests, Seurat delivered the overall best performance in cells clustering. The above-mentioned references are indicated in section 5 and 6.

In section 5 we described that bootstraps are used to estimate the cell stability score using SIMLR. To clarify that also in Seurat clustering the bootstraps are implemented we added the following phrase in section 6: "The bootstrap approach described in section 5.1 is also applied to Seurat clustering to assign cell stability score to the clustered cells."

In rCASC tSne is implemented using the Rtsne package (https://cran.r-project.org/web/packages/Rtsne/index.html). The tSne implementation is only used for comparison with respect to SIMLR and Seurat in section 6.1. Rtsne package provides an internal k-mean clustering, which is performed on the dimensionality reduced data-points. To clarify this point we added the following phrases in section 6.1: "The bootstrap approach described in section 5.1 is also applied on tSne clustering to assign cell stability score to the clustered cells. In rCASC tSne is implemented using the Rtsne (https://cran.r-project.org/web/packages/Rtsne/index.html) package. Rtsne performs a data reduction on which k-mean clustering is applied."

Concerning scalability and the number of input genes a similar request was also asked by reviewer 1. Therefore, we run a scalability test using the GSE106264 dataset described in Section 8 of the vignette. The scalability analysis is described in the main manuscript in materials section subsection scalability: "To estimate the scalability of rCASC clustering we used the GSE106264 dataset made of 10035 cells and published by Pace and coworkers in 2018 [18]. We randomly sampled the 10035 cells (27998 ENSEMBL GENE IDs) to obtain the following subsets of cells: 400, 600, 800, 1000, 2000, 5000. Starting from the 800 cells set we randomly sampled the genes: 10000, 8000, 6000, 4000, 2000, 1000, 800. We run SIMLR, tSne, griph and Seurat using 160 permutation within SeqBox hardware [26]: Intel i7 3.5GHz (4 cores), 32 GB RAM and 500 GB SSD disk. SIMLR resulted to be the slowest and, given the used workstation, it cannot allocate for the analysis more than 2000 cells (Figure 7A). All the other tools were able to handle up to 5000 cells, within the limit of 32 GB of RAM available in the hardware setting used in this analysis. Computation time was nearly linear for all tools till 1000 cells. Only griph clustering resulted to be nearly insensitive to the increasing number of cells (Figure 7A). The computing time as function of increasing number of genes has a quite limited effect on the overall computing time (Figure 7B)."

Q10) There are typos and phrasing issues in the figure legends and the vignette. For example, the legend of figure 2 needs editing to make it understandable.
A10) We thank reviewer for the careful and precise reading of our manuscript. We edited the vignette to eliminate typos and phrasing issues.

Minor comments
Q1) The processing of raw sequencing data in the form of fastq files is computationally expensive and usually performed on high performance computing systems. The authors decided to implement wrapper functions to process 10X and inDrop data. While these technologies are used by the majority of the field, other technologies generate individual fastq files per cell and a function could be implemented to process this data. Furthermore, the authors use the pre-build genomes supplied by 10X for the cellranger pipeline but on the other hand build the reference for the inDrop pipeline from scratch. These approaches are not comparable since the 10X genomes are filtered to only include protein-coding genes. The authors should therefore implement a wrapper function for the cellranger mkref call to allow a more flexible use of genomic references.
A1) We thank reviewer for the useful comments. Concerning the processing of fastq data we implemented inDrop and 10XGenomics fastq processing software because they are compliant with the minimal hardware requirements indicated in Section 1.1.
For smart-seq we have added into the vignette the following paragraph: "Section 2.3 Smart-seq full transcript sequencing.
Smart-seq protocol generates a full transcript library for each cell, i.e. a fastq file for each cell. To convert fastq in counts we suggest to use rnaseqCounts or wrapperSalmon counts from docker4seq package [Kulkarni et al.]. Both above-mentioned functions are compliant with minimal hardware requirements indicated for rCASC and are part, as rCASC, of the Reproducible Bioinformatics Project. The function rnaseqCounts is a wrapper executing on each fastq:
• quality evaluation of fastq with FastQC software,
• trimming of adapters with skewer,
• mapping reads on genome using STAR and counting isoforms and genes with RSEM.
The function wrapperSalmon instead implements FastQC and skewer and calculates isoforms and genes counts using Salmon software."
We have also implemented a new function called cellrangeIndexing, which allows the generation of 10Xgenomics compliant reference genome. The description of cellrangeIndexing was added to the vignette in Section 2.2.
Q2) The framework is developed to run on a linux machine and it would be useful to provide an implementation for Mac and Windows.
A2) The rCASC framework was developed to be compliant with SeqBox (Beccuti et al. Bioinformatics 2017), i7 3.5GHz, 32GB RAM, 500 GB SSD running linux. We have tested rCASC with the latest version of Docker Desktop (v 2.0.0.3) on a Mac machine with 16 GB RAM i7 GHz 3.5, 4 cores. Configuring the virtual machine to use 12 GB RAM and 2 cores we can execute all examples provided in the rCASC vignette. However, RAM requirements become quite demanding, exceeding 12 GB, when more than 1000 cells are used for clustering. We decided to not extend the rCASC framework to window platform. Instead, within the Elixir framework, we are in the early phase of porting rCASC (https://github.com/pmandreoli/rCASC_wrappers) in LANIAKEA galaxy (https://elixir-italy-science-gateway.cloud.ba.infn.it/) in collaboration with LANIAKEA's developers.
Q3) On page 16 in the vignette, the authors discuss the relationship between the number of reads per cell and the number of genes detected. While this dependency is known, the authors should acknowledge that different cell-types show differences in their transcriptional rate and that a

technical assessment of the reads per cell vs. genes detected is difficult to perform when comparing different cell-types.

A3) We thank reviewer for this important indication. To incorporate reviewer's suggestion, we added in the page 16 the following phrase: "However, it has to be underlined that each cell type is characterized by a peculiar transcriptional rate and therefore the technical assessment of the reads per cell vs. genes detected between different cell types, i.e. Figure 13A-C, might be bias by differences in the transcriptional rate of the different cells used in this specific example. Instead, the above-mentioned bias does not affect Figure 13D-F because they are generated by a down sampling of a set of cells sequenced with the smart-seq protocol at a coverage of 1 million reads/cell."

Q4) Figure 16, page 20: It is not possible to identify the cells that were removed after filtering.

A4) In Figure 16 the removed cells are those labelled in blue. Figure 16 was modified adding arrows to better highlight cells that were removed and Figure 16 legend: "Effect of Lorenz filtering, cells shown in blue have been discarded because of their low quality"
was modified in the following way:
"Lorenz filtering: cells retained after filtering are labelled in red as instead cells discarded because of their low quality are labelled in blue."

Q5) Figure 21 needs more explanation in the figure legend

A5) the phrase: "checkCountDepth output plot" was modified in: "checkCountDepth output plot provides an evaluation of count-depth relationship in un-normalized data. The effects of the normalization procedure is shown in the following figure."

Q6) It is not possible to see the CSS for individual cells as displayed by the authors. I would recommend displaying a side-by-side plot where cells in one plot are coloured by cluster ID and cells in the other plot are coloured based on their CSS.

A6) A new plot was added to the NameOfCountMatrix_Stability_Plot.pdf file, which provides the results of the clustering. The new plot provides the cluster picture with cell coloured on the basis of their CSS. Figure 38 was also modified to include this new plot. CSS is described with the following colours: 0-25% black, 25-50% green, 50-75% gold and 75-100% red. The phrase: "The plot in Figure 38C provides a 2D view of the clustering results. In this plot each cell is labeled with a symbol indicating its cell stability score."
was modified in the following way:
"In each clustering folder there is a pdf named NameOfCountMatrix_Stability_Plot.pdf, which contains two plots (Figure 38C-D) generated by the clustering program. These plots provide a 2D view of the clustering results from two different perspectives. In Figure 38C plot each cell is coloured on the basis of the belonging cluster and it is labeled with a symbol indicating its cell stability score (CSS). Instead, in the plot in Figure 38D each cell is coloured on the basis of its CSS: 0-25% black, 25-50% green, 50-75% gold and 75-100% red."

Q7) Page 51 in the vignette: there is a broken link to one figure

A7) We fixed it

Q8) There is a colouring discrepancy between Figure 51 (Z score transformed counts) and Figure 54 (log10-tranformed counts) where the rCASC uses the same colour scale.

A8) Now the colours of Fig. 51 B and C correspond to those in Fig. 54 B and C.

Q9) Page 66 in the vignette: the authors should better explain why they chose k=6 and not k=7 and where the difference between Figure 57 A and B is coming from.

A9) To clarify the description of fig 57, the phrase:
"In Figure 57 are summarized the results of the analysis executed on the Pace's dataset. Data perturbations, Figure 57A, allows data organization between 6 to 9 clusters, where 7 clusters is the most represented group. Cell stability score, from the SIMLR analysis executed on the above range of clusters, is shown in Figure 57B. Six clusters show a slightly higher stability with respect to the

others. The overall stability of 6 clusters is however sub-optimal, since it is spread between 0 and 0.9 cell stability score. In Figure 57C it is shown the clusters structure generated with SIMLR on 6 clusters. Clusters 1, 3 and 4 show a quite good stability, Figure 57C. Cluster 3 is made of 44 N (88%) and 48 Nd (96%), suggesting that naive CD8+ T lymphocytes are not affected by the silencing of Suv39h1 gene. Cluster 1 contains 16 NA (6.4%) and 14 NdA (5.6%). Cluster 2 is made of 44.8% of NA and 39.6% of NdA cells. Clusters 1 and 2 group together, interdependently from Suv39h1 gene silencing. Cluster 6 is made of 35% NA and 13.6% of NdA. In cluster 6 the amount of NA and NdA is unbalance, suggesting that the Suv39h1 silencing does not guarantee the efficient differentiation of the cell subpopulation in cluster 6. Cluster 4 only contains NdA (33%), indicating that at least a subpopulation of activated Suv39h1-silenced cells has a specific transcription profile that differentiate them from the wild type activated cells. Cluster 5 is made of 6 N cells, 2 Nd cells, 34 NA cells, 21 NdA cells. Despite the presence of a limited amount of naive cells, which might be explained as partially activated, cluster 5 is made mainly of activated cells, i.e. 13.6% NA and 8.4% NdA of total cells. The cluster structure (Figure 57D) and cell cluster stability scores (Figure 57C) might suggest that cluster 5 is made of a precursor subset."

was modified in the following way:

"In Figure 57 are summarized the results of the analysis executed on the Pace's dataset. A limitation of the clustering based on SIMLR is due to the need of providing as input the number of clusters (k) in which the data should be organized. Instead of asking to user to define arbitrarily the k number of clusters, we used griph (https://github.com/ppapasaikas/griph) as tool to identify a range of k clusters to be inspected by SIMLR. Figure 57A shows the frequency of the k number of clusters, in which the Pace's dataset can be organized using griph software, upon 160 bootstraps in which 10% of the cells is randomly removed from the initial data set. Griph analysis identify a range of clusters going from k=6 to k=9. K=7 is the most represented data organization detected by griph, followed by 8, 6 and 9 clusters.

The range of k clusters detected using griph is then investigated with SIMLR. SIMLR is run for each k of the k-range defined with griph tool. CSS violin plot (Figure 57B) shows that the mean stability for k=6 ($CSS_m \sim 0.5$) is higher than to the others ks ($CSS_m < 0.3$).

Clusters k=7 and k=8 do not represent the most stable organizations in terms of CSS (Figure 57B), although they are the most frequent organizations observed in griph analysis (Figure 57A).

Since the best CSS_m is observed in k=6, we explored these clusters (Figure 57C). In Figure 57C, clusters 1, 3 and 4 show a quite good stability, since cells stay in these clusters between 75% to 100% of the bootstraps.

The inspection of Pace's experiment groups organization (i.e. N= naïve WT, Nd= naïve Suv39h1 KO, NA=activated WT, NdA=activated Suv39h1 KO) in k=6 clusters, Figure 57C, show that cluster number 4 is the only one containing only NdA (33% of the total NdA) cells. Thus, suggesting that a subpopulation of activated Suv39h1-silenced cells has a specific transcription profile, which differentiates them from all wild type activated cells. Another interesting cluster is number 6, where the amount of NA and NdA is unbalance, 35% NA and 13.6% of NdA, suggesting that Suv39h1 silencing does not guarantee at the same efficiency the differentiation of this cell subpopulation as in the case of wild type cells. Cluster 5 is the most heterogeneous cluster. It is composed by 6 N cells, 2 Nd cells, 34 NA cells, 21 NdA cells. Despite the presence of a limited number of naive cells, which might be explained as partially activated, cluster 5 is composed by an unbalance number of activated cells, i.e. 13.6% NA and 8.4% NdA of total cells. However, since cluster 5 is characterized by a very low CSS (0-25%) it is possible that this cluster contains cells localized at the boundaries of clusters 2, 3 and 6. On the other side clusters 1, 2, 3 have nearly the same number of wild type and Suv39h1 silenced cells, suggesting that these subsets of cells are not influenced by Suv39h1 silencing:

- cluster 1 contains nearly the same amount of activated wild type, 16 NA (6.4%), and Suv39h1 KO cells, 14 NdA (5.6%); cluster 2 is made of 44.8% of NA and 39.6% of NdA cells;
- cluster 2 is made of 44.8% of NA and 39.6% of NdA cells;
- cluster 3 is made of 44 N (88%) and 48 Nd (96%)”
