# Supplementary material for: rCASC: reproducible classification analysis of single-cell sequencing data
Source: Gigascience. 2019 Sep 8;8(9):giz105. doi: 10.1093/gigascience/giz105 (PMC6732171; doi:10.1093/gigascience/giz105)
Supplement: giz105_GIGA-D-18-00522_Revision_3 [file giz105_giga-d-18-00522_revision_3.pdf]

GigaScience
rCASC: reproducible Classification Analysis of Single Cell sequencing data
--Manuscript Draft--

**Manuscript Number:**	GIGA-D-18-00522R3
**Full Title:**	rCASC: reproducible Classification Analysis of Single Cell sequencing data
**Article Type:**	Technical Note
**Funding Information:**	
**Abstract:**	Background<br>Single-cell RNA sequencing is an essential tool to investigate cellular heterogeneity, and to highlight cell sub-population specific signatures. Single-cell sequencing applications are now spreading from the most conventional RNAseq to epigenomics, e.g. ATAC-seq. Single-cell sequencing led to the development of a large variety of algorithms and associated tools. However, to the best of our knowledge, there are few computational workflows providing analysis flexibility and achieving at the same time functional (i.e. information about the data and the utilized tools are saved in terms of meta-data) and computational reproducibility (i.e. real image of the computational environment used to generate the data is stored) through a user-friendly environment.<br><br>Findings<br><br>rCASC is a modular workflow providing integrated analysis environment (from counts generation to cell subpopulation identification) exploiting docker containerization to achieve both functional and computational reproducibility in data analysis. Hence, rCASC provides preprocessing tools to remove low quality cells and/or specific bias, e.g. cell cycle. Subpopulations discovery can be instead achieved using different clustering techniques based on different distance metrics. Quality of clusters is then estimated through a new metric namely Cell Stability Score (CSS), which describes the stability of a cell in a cluster as consequence of a perturbation induced by removing a random set of cells from the overall cells' population. Our experiments highlight that CSS provides better cluster-robustness information than silhouette metric. Moreover, rCASC provides tools for the identification of clusters-specific gene-signature.<br><br>Conclusions<br><br>rCASC is a modular workflow with valuable new features that could help researchers in defining cells subpopulations and in detecting subpopulation specific markers. It exploits docker framework to make easier its installation and to achieve a computation reproducible analysis. Moreover, a Java Graphical User Interface (GUI), is provided in rCASC to make friendly the use of the tool even for users without computational skills in R.
**Corresponding Author:**	Marco Beccuti, Ph.D<br>Universita degli Studi di Torino<br>Turin, Piemonte ITALY
**Corresponding Author Secondary Information:**	
**Corresponding Author's Institution:**	Universita degli Studi di Torino
**Corresponding Author's Secondary Institution:**	
**First Author:**	Luca Alessandrì, Ph.D student
**First Author Secondary Information:**	
**Order of Authors:**	Luca Alessandrì, Ph.D student
	Francesca Cordero, Ph.D.
	Marco Beccuti, Ph.D.

Powered by Editorial Manager® and ProduXion Manager® from Aries Systems Corporation

	Maddalena Arigoni, Ph.D.
	Martina Olivero, Ph.D.
	Greta Romano
	Sergio Rabellino
	Nicola Licheri, Ph.D student
	Gennaro De Libero, Ph.D.
	Luigia Pace, Ph.D.
	Raffaele Calogero
**Order of Authors Secondary Information:**	
**Response to Reviewers:**	rCASC: reproducible Classification Analysis of Single Cell sequencing data.<br><br>Luca Alessandrì, Francesca Cordero, Marco Beccuti, Maddalena Arigoni, Martina Olivero, Greta Romano, Sergio Rabellino,Nicola Licheri, Gennaro De Libero, Luigia Pace and Raffaele A Calogero<br><br>Dear Editor,<br>First of all, we wish to thank the reviewers for their valuable comments and useful suggestions which helped us to substantially improve the paper and its associated tool.<br><br>Hereafter we report our answers to the reviews' comments.<br><br>Reviewer reports:<br><br>Reviewer #1: The authors incorporated additional clustering methods (Scanpy and Griph) that prove to be scalable for datasets having larger sizes which corresponds to the field needs.<br>In particular, Scanpy seems to reveal no issue to scale up to 100K cells in the benchmark executed opposite to the other methods.<br>I recommend accepting this manuscript since I think it is well suited for current and future analytical needs for single cells.<br><br>Minor comments:<br><br>Question 1: Is there any limitation or trick to use for the preprocessing procedures (low cell quality filter, normalization, annotation, cell cycle removal, matrix creation) executed before the clustering when increasing the sample / feature size?<br>I presume no because the authors have used them with large dataset. Then, It will be worth metioning that in the manuscript with a brief estimate of the computational time / memory needed.<br><br>Answer 1: All samples were preprocessed removing ribosomal/mitochondrial protein genes and cells with a total count of UMIs lower than 100. This information was added in the scalability paragraph: "All the above samples were preprocessed removing ribosomal/mitochondrial protein genes and cells with a total count of UMIs lower than 100."<br>Concerning the computational time/memory required for the analysis we added the following phrase at the end of Scalability paragraph:<br>"The definition of the computing time for an analysis depends on multiple parameters: i) the number of permutations performed in parallel, ii) the number of cells under analysis, iii) the clustering tool in use and iv) the hardware used for the analysis. Concerning the amount of RAM required for each permutation run in parallel, up to 5000 cells the maximum amount of RAM required is approximately 4 GB, from 10000 to 100000 cells, the maximum RAM required is approximately 20 GB. Independently by the clustering approach and the size of the dataset, we suggest to run at least 100 permutations to correctly estimate CSS."<br><br>Question 2: The figure 3 is not updated with Scanpy and griph.

	Answer2: We updated Fig. 3 as suggested by the reviewer. Moreover, we updated Fig. 4C which now includes griph and scanpy functions<br>Question 3: I don't understand the use of the term hierarchical clustering in the manuscript and in the suppl. material.<br><br>Answer 3: We removed the term "hierarchical" from Fig. 1 and in supplementary data.<br><br>--<br>Concerning software repository:<br>--<br><br>Dear Editor,<br>hereafter we reported general information about the current software repositories:<br><br>1. The rCASC package is available at this github repository: https://github.com/kendomaniac/rCASC<br>2. All the docker images are stored in the docker hub: docker.io/repbioinfo/<br>3.GUI for rCASC is available at this github repository: https://github.com/mbeccuti/4SeqGUI<br>4. All the sample data are retrievable at 130.192.119.59 and the paths are indicated in the supplementary material.<br><br>Moreover we registered rCASC in bio.tools and in SciCrunch.org (id: SCR_017005)
**Additional Information:**	
**Question**	**Response**
Are you submitting this manuscript to a special series or article collection?	No
**Experimental design and statistics**<br><br>Full details of the experimental design and statistical methods used should be given in the Methods section, as detailed in our Minimum Standards Reporting Checklist. Information essential to interpreting the data presented should be made available in the figure legends.<br><br>Have you included all the information requested in your manuscript?	Yes
**Resources**<br><br>A description of all resources used, including antibodies, cell lines, animals and software tools, with enough information to allow them to be uniquely identified, should be included in the Methods section. Authors are strongly	Yes

Powered by Editorial Manager® and ProduXion Manager® from Aries Systems Corporation

encouraged to cite Research Resource Identifiers (RRIDs) for antibodies, model organisms and tools, where possible.<br><br>Have you included the information requested as detailed in our Minimum Standards Reporting Checklist?	
**Availability of data and materials**<br><br>All datasets and code on which the conclusions of the paper rely must be either included in your submission or deposited in publicly available repositories (where available and ethically appropriate), referencing such data using a unique identifier in the references and in the "Availability of Data and Materials" section of your manuscript.<br><br>Have you have met the above requirement as detailed in our Minimum Standards Reporting Checklist?	Yes

Manuscript

Click here to view linked References

Click here to access/download;Manuscript;alessandri_et_al_gigascience_re

rCASC: reproducible Classification Analysis of Single Cell sequencing data

[1#]Luca Alessandrì, [2#]Francesca Cordero, [2$]Marco Beccuti, [1]Maddalena Arigoni, [3]Martina Olivero, [2]Greta Romano, [2]Sergio Rabellino, [2]Nicola Licheri, [4]Gennaro De Libero, [5]*Luigia Pace and [1]*Raffaele A Calogero

[1]Department of Molecular Biotechnology and Health Sciences, University of Torino, Via Nizza 52, Torino, Italy

[2]Department of Computer Sciences, University of Torino, Corso Svizzera 185, Torino, Italy

[3]Department of Oncology, University of Torino, SP142, 95, 10060 Candiolo TO, Italy

[4]Department Biomedizin, University of Basel, Hebelstrasse 20, 4031 Basel, Switzerland

[5]IIGM, Via Nizza 52, Torino, Italy

#Both authors equally contributed the present work

*Both authors equally supervised the present work

[$]Corresponding author

Luca Alessandrì alessandri.luca1991@gmail.com

Francesca Cordero fcordero@di.unito.it

Marco Beccuti beccuti@di.unito.it, ORCID: 0000-0001-6125-9460

Maddalena Arigoni maddalena.arigoni@unito.it

Martina Olivero martina.olivero@unito.it

Greta Romano greta.romano@unito.it

Sergio Rabellino sergio.rabellino@unito.it, ORCID: 0000-0002-1757-2000

Nicola Licheri nicola.licheri@unito.it

Gennaro De Libero gennaro.delibero@unibas.ch, ORCID: 0000-0003-0853-7868

Luigia Pace luigia.pace@iigm.it

Raffaele A Calogero raffaele.calogero@unito.it, ORCID: 0000-0002-2848-628X

Abstract

Background

Single-cell RNA sequencing is an essential tool to investigate cellular heterogeneity, and to highlight cell sub-population specific signatures. Single-cell sequencing applications are now spreading from the most conventional RNAseq to epigenomics, e.g. ATAC-seq. Single-cell sequencing led to the development of a large variety of algorithms and associated tools. However, to the best of our knowledge, there are few computational workflows providing analysis flexibility and achieving at the same time functional (i.e. information about the data and the utilized tools are saved in terms of meta-data) and computational reproducibility (i.e. real image of the computational environment used to generate the data is stored) through a user-friendly environment.

Findings

rCASC is a modular workflow providing integrated analysis environment (from counts generation to cell subpopulation identification) exploiting docker containerization to achieve both functional and computational reproducibility in data analysis. Hence, rCASC provides preprocessing tools to remove low quality cells and/or specific bias, e.g. cell cycle. Subpopulations discovery can be instead achieved using different clustering techniques based on different distance metrics. Quality of clusters is then estimated through a new metric namely Cell Stability Score (CSS), which describes the stability of a cell in a cluster as consequence of a perturbation induced by removing a random set of cells from the overall cells' population. Our experiments highlight that CSS provides better cluster-robustness information than silhouette metric. Moreover, rCASC provides tools for the identification of clusters-specific gene-signature.

Conclusions

rCASC is a modular workflow with valuable new features that could help researchers in defining cells subpopulations and in detecting subpopulation specific markers. It exploits docker framework to make easier its installation and to achieve a computation reproducible analysis. Moreover, a Java Graphical User Interface (GUI), is provided in rCASC to make friendly the use of the tool even for users without computational skills in R.

Keywords

Single-cell data preprocessing, workflow, GUI, clustering, cluster stability metrics, cluster-specific gene signature.

Findings

rCASC: a single cell analysis workflow designed to provide data reproducibility.

Since the end of the 90's omics high-throughput technologies have generated an enormous amount of data, reaching today an exponential growth phase. The analysis of omics big data is a revolutionary means of understanding the molecular basis of disease regulation and susceptibility, and this resource is made accessible to the biological/medical community via bioinformatics frameworks. However, due to the increasing complexity and the fast evolution of omics methods, the reproducibility crisis [1] is becoming a very important issue [2] and there is a mandatory need to guarantee robust and reliable results to the research community [3].

Single cell analysis is instrumental to understand the functional differences existing among cells within a tissue. Individual cells of the same phenotype are commonly viewed as identical functional units of a tissue or an organ. However, single cells sequencing results [4] suggest the presence of a complex organization of heterogeneous cell states producing together system-level functionalities. A mandatory element of single cell RNAseq is the availability of dedicated bioinformatics workflows.

To the best of our knowledge, rCASC is the only computational framework, which provides both computational and functional reproducibility for an integrated analysis of single cell data, from counts generation to cell subpopulation identification. It is one of the tools developed under the umbrella of the Reproducible Bioinformatics project[1] (http://reproducible-bioinformatics.org/), an open-source community aimed to provide to biologists and medical scientists an easy-to-use and flexible framework, which also guarantees the ability to reproduce results independently by the underlying hardware, using docker containerization (computational reproducibility). Indeed, it was developed following the best practice rules for reproducible computational research, proposed in 2013 by Sandve [5]. It is also listed within the tools developed by the Italian Elixir node (https://bio.tools/rCASC).

In details all the computational tools in rCASC are embedded in docker images stored in a public repository on docker hub. Parameters are delivered to docker containers via a set of R functions, part of rCASC R github package [8]. To simplify the use of rCASC package to users without scripting experience, R functions can be controlled by a dedicated GUI, integrated in the 4SeqGUI tool

[1] The reproducible bioinformatics project was founded and it is maintained by the research team of Elixir node at University of Turin. The reproducible bioinformatics project was published on BMC Bioinformatics [6]. An example of stand-alone hardware/software infrastructure for bulk RNAseq, developed within the Reproducible Bioinformatics project, was described in Beccuti [7].

previously published by us [7], which is also available as github package [9]. rCASC is specifically designed to provide an integrated analysis environment for cell subpopulation discovery. The workflow allows the direct analysis of fastq files, generated with 10X Genomics and inDrop platforms, or count matrices. Therefore, rCASC provides raw data preprocessing, subpopulation discovery via different clustering approaches and cluster-specific genes-signatures detection. The key elements of rCASC workflow are shown in Figure 1, and the main functionalities are summarized in Methods section. A detailed description of the rCASC functions is also available in the vignettes section of rCASC github [8].

The overall characteristics of rCASC were compared with other four workflows for single-cells analysis (Figure 2): i) simpleSingleCell, Bioconductor workflow package [10]; ii) Granatum, web-based scRNA-Seq analysis suite [11]; iii) SCell, graphical workflow for single-cell analysis [12]; iv) R toolkit Seurat [13]. The comparison was based on the following elements: a) supported single-cell platforms, b) types of tools provided by the workflow, c) type of reproducibility granted by the workflow, d) tools flexibility.

rCASC is the only workflow providing support at fastq level because all the other packages require as input the processed counts table. Cell quality control and outliers' identification is available in all the workflows but Granatum. Association of ENSEMBL gene IDs to gene symbols is only provided by rCASC. All the workflows provide genes filtering tools but simpleSingleCell. All packages provide normalization procedures to be applied to raw counts data. However, rCASC is the only tool providing both Seurat specific normalization [13] and count-depth specific normalization [14]. The workflows implement different data reduction and clustering methods. rCASC integrates four clustering tools, i.e. Seurat [13] SIMLR [15], griph [16], and scanpy [17] which differ in the metrics driving the clustering analysis. Cluster stability is an important topic in Clustering (for a review see [19]). Stability measurement, taking advantage of bootstrapping, was also addressed by Hennig [20]. Specifically, Hennig uses Jaccard index to evaluate the overall stability of each cluster. In rCASC, we have implemented a cell stability score (CSS), which uses the Jaccard index to estimate the stability of each cell in each cluster. CSS provides an enhanced description of each cluster, since it allows the identification of subset of cells, in any cluster, which are particularly sensitive to perturbation of the overall dataset structure, i.e. cell bootstrapping. Moreover, the cluster stability measurement proposed by Henning was included in rCASC. Specifically, we have implemented the "clusterboot" function from the fpc R package [21], which allows the evaluation of the cluster stability using a personalized clustering function (see Supplementary file Section 5.3). To the best of our knowledge, rCASC is the only workflow performing clustering in presence of data perturbation,

i.e. removal of a subset of cells, and measuring cluster quality using Cell Stability Score (CSS is a cluster quality metrics developed by us, which measures the persistence of each cell in a cluster upon data perturbation, see Supplementary file section 5.1) and Silhouette score (SS is a cluster quality metrics measuring the consistency within clusters of data). In our experiments, CSS provides a better estimation of the cluster stability compared to that of SS (Figure 2). Gene feature selection approaches are implemented in different way in the five workflows. Granatum is the only one providing biological inference. Granatum and Seurat implements various statistical methods to detect cluster specific genes signatures (Figure 3). rCASC embeds an ANOVA-like statistics derived from EdgeR Bioconductor package [22] and Seurat/SIMLR genes prioritization procedures (see Supplementary file section 7). Visualization of genes-signatures by heatmap, coloring cells on the basis of gene expression is only provided by rCASC (see Supplementary file Figure 51). Considering reproducibility, only rCASC provides both computational and functional reproducibility. Finally, rCASC is the only one providing both a command line interface and graphical user interface (Figure 4).

Finally, rCASC was used to re-analyze the single-cell dataset from Pace paper [23]. In this paper, authors highlighted that Suv39h1-defective $CD8^+$ T-cells show sustained survival and increased long-term memory reprogramming capacity. Our re-analysis extends the information described in Pace paper, suggesting the presence of an enriched Suv39h1-defective memory subset. A complete description of the above analysis is available at section 8 of supplementary file.

Methods

Counts table generation

inDrop single-cell sequencing approach was originally published by Klein [24]. Then, the authors published the detailed protocol in *Nature Methods* in 2017 [25]. In rCASC, the generation of the count table starting from fastq files refers to the version 2 of the inDrop chemistry described in [25], which is commercially distributed by 1CellBio. The procedure described in the inDrop github [26] is embedded in a docker image. rCASC function *indropIndex* allows the generation of the transcripts index required to convert fastq in counts, and *indropCounts* function converts reads in UMI counts. 10XGenomics Cellranger is packed in a docker image and the function *cellrangerCount* converts fastq to UMI matrix using any of the genome indexes with *cellrangerIndexing* function. Detailed description about the counts table generation is available in Supplementary file section 2.

Counts table exploration and manipulation

rCASC provides various data inspection and preprocessing tools.

genesUmi function generates a plot where the number of detected genes are plotted for each cell with respect to the number of UMI (Figure 5A,C).

mitoRiboUmi calculates the percentage of mitochondrial/ribosomal genes with respect to the total number of detected genes in each cell and plots percentage of mitochondrial genes with respect to percentage of ribosomal genes. Furthermore, cells are colored on the basis of the number of detected genes (Figure 5B, D). *mitoRiboUmi* allows to identify cells with low information content, i.e. those cells with a little number of detectable genes, e.g. < 100 genes/cell, little ribosomal content and high content of mitochondrial genes, which indicate cell stress [27].

The function *scannobyGtf* uses ENSEMBL gtf and the R package refGenome to associate gene symbol with the ENSEMBL gene ID. Furthermore, *scannobyGtf* allows one to remove mitochondrial/ribosomal genes (Figure 5A, C) and "stressed" cells detectable with *mitoRiboUmi* function (Figure 5B, D).

The function *lorenzFilter* embeds the Lorenz statistics developed by Diaz [12], a cell quality statistics correlated with cell live-dead staining (see Supplementary file sections 3.3). Specifically, the outlier filtering for single-cell RNA-seq experiments designed by Diaz estimates which genes are expressed at background levels in each sample, then samples with significantly high background levels are discarded [12].

As counts table preprocessing steps, we implemented the functions *checkCountDepth/scnorm* to detect the presence of sample specific count–depth relationship [14] (i.e. the relationship existing between transcript-specific expression and sequencing depth) and to adjust the counts table for it. Specifically, *checkCountDepth* initially executes a quantile regression, thus estimating the dependence of transcript expression on sequencing depth for every gene. Then, genes with similar dependence are aggregated (see Supplementary file section Figure 21). *Scnorm,* after executing *checkCountDepth*, performs a new quantile regression to estimate scale factors within each group of genes. Then, sequencing depth adjustment is done within each group using the estimated scale factors. Furthermore, we added two other functions *recatPrediction* and *ccRemove,* which are based respectively on the paper of Liu [28] and Barron [29]. The function *recatPrediction* organizes the single cell data to reconstruct cell cycle pseudo time-series and it is used to understand if a cell cycle effect is present. The above function embeds reCAT software [28], which models the reconstruction of time-series as a traveling salesman problem, thus identifying the shortest possible cycle by passing through each cell exactly once and returning to the start. Since the traveling salesman problem is a NP-hard problem, reCAT is based on a heuristic algorithm, which is used to find the solution.

ccRemove function is instead based on the work of Barron and Li [29] and embeds their scLVM (single-cell latent variable model) algorithm, which uses a sophisticated Bayesian latent variable

model to reconstruct hidden factors in the expression profile of the cell-cycle genes. This algorithm is able to remove cell-cycle effect from real scRNA-Seq datasets. Thus, *ccRemove* is used to mitigate the cell cycle effect of the inter-samples transcriptome, when it is detected by *recatPrediction* function (see Supplementary file sections 3.6 ad 3.8).

Clustering

For the identification of cell subpopulations we implemented four approaches: Seurat (RRID:SCR_016341) [13], SIMLR [15], griph [16] and scanpy [17]. Seurat is a toolbox for single-cell RNAseq data analysis. We implemented in rCASC one of the clustering procedures present in Seurat toolbox. The function *seuratPCAEval* has to be run before executing the clustering program to identify the 'metafeatures', i.e. the subset of PCA components describing the relevant source of cells' heterogeneity, to be used for clustering. *seuratBootstrap* function implements data reduction and clustering. Specifically, cells undergo to global scaling normalization, i.e. LogNormalize method, and scaling factor 10000. Subsequently, a linear dimensional reduction is done using the range of principal components defined with *seuratPCAEval*. Then, clustering is performed using the cell PCA scores. The Seurat clustering procedure, embedded in *seuratBootstrap,* is based on the Louvain modularity optimization algorithm. Differently SIMLR implements a k-mean clustering, where the number of clusters (i.e. k) is taken as input. SIMLR, requires as input raw counts $\log_{10}$ transformed. SIMLR is capable of learning an appropriate cell-to-cell similarity metric from the input single-cell data and to exploit it for the clustering task. In the learning phase SIMLR identifies a distance metric that better fits the structure of the data by combining multiple Gaussian kernels [15]. Thus, the tool can deal with the large noise and drop-out effects of single-cell data, which could not easily fit with specific statistical assumptions made by standard dimension reduction algorithms [15]. The function *simlrBootstrap* controls the clustering procedure and the function *nClusterEvaluationSIMLR*, a wrapper for the R package griph (Graph Inference of Population Heterogeneity) [16], is exploited to estimate the (sub)optimal number "k" of clusters. Griph clustering [16] is based on Louvain modularity. Griph algorithm is closer to agglomerative clustering methods, since every node is initially assigned to its own community and communities are subsequently built by iterative merging. Also scanpy [17] uses for clustering a heuristic method based on modularity optimization.

We developed, for Seurat, SIMLR, griph and scanpy, a procedure to measure the cluster quality on the basis of data structure. The rationale of our approach is that cells belonging to a specific cluster should be little affected by changes in the numerosity of the dataset, e.g. removal of 10% of the total number of cells used for clustering. Thus, we developed a metrics called CSS (Cell Stability Score), which describes the persistence of a cell in a specific cluster upon Jackknife resampling and therefore offers a peculiar way of describing cluster stability. Detailed description of CSS metrics is available

in Supplementary file at section 5.1. CSS is embedded in *seuratBootstrap*, *simlrBootstrap*, *scanpyBootstrap* and *griphBootstrap*.

Feature selection

To select the most important features of each cluster we implemented in the *anovaLike* function the edgeR ANOVA-like method for single cells [22] and in the functions *seuratPrior* and *genesPrioritization/genesSelection* respectively the Seurat and SIMLR genes prioritization methods. *hfc* function allows the visualization of the genes prioritized with the above methods as heatmap and provides plots of prioritized genes in each single cell (Figure 6).

Scalability

To estimate the scalability of rCASC clustering we used the GSE106264 dataset made of 10,035 cells and published by Pace and coworkers in 2018 [23] and the 10,000/33,000/68,000 cells PBMC human datasets, available at 10xGenomics repository (www.10xgenomics.com). We randomly generated from the 10035 cells (27998 ENSEMBL GENE IDs) the following subsets of cells: 400, 600, 800, 1000, 2000, 5000. Moreover for the subsets with more than 600 cells we randomly sampled the genes: 10000, 8000, 6000, 4000, 2000, 1000, 800. We run SIMLR, tSne, griph and Seurat using 160 permutations within SeqBox hardware [7]: Intel i7 3.5GHz (4 cores), 32 GB RAM and 500 GB SSD disk. SIMLR resulted to be the slowest and, given the above hardware implementation, it cannot allocate for the analysis more than 2000 cells (Figure 7A left panel). All the other tools were able to handle up to 5000 cells within the limit of 32 GB of RAM available in the hardware setting used in this analysis. Computation time was nearly linear for all tools till 1000 cells. Only griph clustering resulted to be nearly insensitive to the increasing number of cells (Figure 7A). We extended, for Seurat, griph and scanpy, the scalability analysis to 10K, 33K, 68K and 101K cells, using 10,000/33,000/68,000 cells from PBMC human datasets, available at 10xGenomics repository (www.10xgenomics.com), and 101,000 cells dataset, made assembling the above mentioned 33,000 and 68,000 PBMC datasets. The analysis was executed on a SGI server (10 x CPU E5-4650 2.4GHz (16 cores), 1TB RAM, 30 TB SATA raid disk) allocating 40 threads for each analysis. Scanpy outperforms the other two methods and griph behaves slightly better than Seurat (Figure 7A right panel).

All the above samples were preprocessed removing ribosomal/mitochondrial protein genes and cells with a total count of UMIs lower than 100.

The computing time as function of increasing number of genes has a quite limited effect on the overall computing time (Figure 7B)."

The definition of the computing time for an analysis depends on multiple parameters: i) the number of permutations performed in parallel, ii) the number of cells under analysis, iii) the clustering tool in

use and iv) the hardware used for the analysis. Concerning the amount of RAM required for each permutation run in parallel, up to 5000 cells the maximum amount of RAM required is approximately 4 GB, from 10000 to 100000 cells, the maximum RAM required is approximately 20 GB. Independently by the clustering approach and the size of the dataset, we suggest to run at least 100 permutations to correctly estimate CSS.

Availability of supporting data

Snapshots of the code and test data are available from the *GigaScience* GigaDB repository [32]. All the docker images are stored in docker hub: https://hub.docker.com/u/repbioinfo

Availability and requirements

Project name: rCASC: reproducible Classification Analysis of Single Cell sequencing data

Project home page: https://github.com/kendomaniac/rCASC; https://github.com/mbeccuti/4SeqGUI

Operating system: Linux

Programming language: R and JAVA

Other Requirements: None

License: The GNU Lesser General Public License, version 3.0 (LGPL-3.0)

RRID:SCR_017005

Authors' contributions

LA and FC equally participated to write R scripts, to create the majority of docker images, to package the workflow and release code. MB wrote the Java and C++ code, and acted as corresponding author. NL implemented scanpy and extended the Java GUI. MA and MO prepared the single-cell data to be used as examples of the workflow functionality. GR prepared the dockers for fastq to counts table conversion. SR revised all packages and generated the docker files for docker images maintenance and further development. GDL gave scientific advices and provided an unpublished dataset for MAIT resting and activated T-cells (generated with Fluidigm C1 platform) to investigate genes detection limits in 3'end sequencing technologies and whole transcript sequencing. RAC and LP equally oversaw the project and gave scientific advices. All authors read, contributed and approved the final manuscript.

Supplementary material

rCASC_supplementary_file.pdf

References

1. Allison DB, Shiffrin RM and Stodden V. Reproducibility of research: Issues and proposed remedies. Proceedings of the National Academy of Sciences of the United States of America. 2018;115 11:2561-2.
2. Nature: Challenges in irreproducible research. https://www.nature.com/collections/prbfkwmwvz (2018). Accessed Special.
3. Calogero RA: Reproducibility in Computational Biology. http://www.global-engage.com/life-science/reproducibility-computational-biology/ (2017).
4. Buettner F, Natarajan KN, Casale FP, Proserpio V, Scialdone A, Theis FJ, et al. Computational analysis of cell-to-cell heterogeneity in single-cell RNA-sequencing data reveals hidden subpopulations of cells. Nature biotechnology. 2015;33 2:155-60. doi:10.1038/nbt.3102.
5. Sandve GK, Nekrutenko A, Taylor J and Hovig E. Ten simple rules for reproducible computational research. PLoS computational biology. 2013;9 10:e1003285. doi:10.1371/journal.pcbi.1003285.
6. Kulkarni N, Alessandri L, Panero R, Arigoni M, Olivero M, Ferrero G, et al. Reproducible bioinformatics project: a community for reproducible bioinformatics analysis pipelines. BMC Bioinformatics. 2018;19 Suppl 10:349. doi:10.1186/s12859-018-2296-x.
7. Beccuti M, Cordero F, Arigoni M, Panero R, Amparore EG, Donatelli S, et al. SeqBox: RNAseq/ChIPseq reproducible analysis on a consumer game computer. Bioinformatics. 2017; doi:10.1093/bioinformatics/btx674.
8. rCASC R Package. https://github.com/kendomaniac/rCASC (2018).
9. 4SeqGUI. https://github.com/mbeccuti/4SeqGUI (2018).
10. Lun AT, McCarthy DJ and Marioni JC. A step-by-step workflow for low-level analysis of single-cell RNA-seq data with Bioconductor. F1000Res. 2016;5:2122. doi:10.12688/f1000research.9501.2.
11. Zhu X, Wolfgruber TK, Tasato A, Arisdakessian C, Garmire DG and Garmire LX. Granatum: a graphical single-cell RNA-Seq analysis pipeline for genomics scientists. Genome medicine. 2017;9 1:108. doi:10.1186/s13073-017-0492-3.
12. Diaz A, Liu SJ, Sandoval C, Pollen A, Nowakowski TJ, Lim DA, et al. SCell: integrated analysis of single-cell RNA-seq data. Bioinformatics. 2016;32 14:2219-20. doi:10.1093/bioinformatics/btw201.
13. Butler A, Hoffman P, Smibert P, Papalexi E and Satija R. Integrating single-cell transcriptomic data across different conditions, technologies, and species. Nature biotechnology. 2018;36 5:411-20. doi:10.1038/nbt.4096.
14. Bacher R, Chu LF, Leng N, Gasch AP, Thomson JA, Stewart RM, et al. SCnorm: robust normalization of single-cell RNA-seq data. Nature methods. 2017;14 6:584-6. doi:10.1038/nmeth.4263.
15. Wang B, Zhu J, Pierson E, Ramazzotti D and Batzoglou S. Visualization and analysis of single-cell RNA-seq data by kernel-based similarity learning. Nature methods. 2017;14 4:414-6. doi:10.1038/nmeth.4207.
16. Serra D, Mayr U, Boni A, Lukonin I, Rempfler M, Challet Meylan L, et al. Self-organization and symmetry breaking in intestinal organoid development. Nature. 2019;569 7754:66-72. doi:10.1038/s41586-019-1146-y.
17. Wolf FA, Angerer P and Theis FJ. SCANPY: large-scale single-cell gene expression data analysis. Genome biology. 2018;19 1:15. doi:10.1186/s13059-017-1382-0.

18. Freytag S, Tian L, Lonnstedt I, Ng M and Bahlo M. Comparison of clustering tools in R for medium-sized 10x Genomics single-cell RNA-sequencing data. F1000Res. 2018;7:1297. doi:10.12688/f1000research.15809.1.
19. Ulrike von Luxburg (2010), "Clustering Stability: An Overview", Foundations and Trends in Machine Learning: Vol. 2: No. 3, pp 235-274. http://dx.doi.org/10.1561/2200000008.
20. Hennig, C; (2007) Cluster-wise assessment of cluster stability. COMPUT STAT DATA AN , 52 (1) 258 - 271. 10.1016/j.csda.2006.11.025.
21. Hennig C: fpc R package. https://cran.r-project.org/web/packages/fpc/index.html.
22. Robinson MD, McCarthy DJ and Smyth GK. edgeR: a Bioconductor package for differential expression analysis of digital gene expression data. Bioinformatics. 2010;26 1:139-40. doi:10.1093/bioinformatics/btp616.
23. Pace L, Goudot C, Zueva E, Gueguen P, Burgdorf N, Waterfall JJ, et al. The epigenetic control of stemness in CD8+ T cell fate commitment. Science (New York, N Y). 2018;359 6372:177-86.
24. Klein AM, Mazutis L, Akartuna I, Tallapragada N, Veres A, Li V, et al. Droplet barcoding for single-cell transcriptomics applied to embryonic stem cells. Cell. 2015;161 5:1187-201. doi:10.1016/j.cell.2015.04.044.
25. Zilionis R, Nainys J, Veres A, Savova V, Zemmour D, Klein AM, et al. Single-cell barcoding and sequencing using droplet microfluidics. Nature protocols. 2017;12 1:44-73. doi:10.1038/nprot.2016.154.
26. indrops github repository. https://github.com/indrops/indrops.
27. AlJanahi AA, Danielsen M and Dunbar CE. An Introduction to the Analysis of Single-Cell RNA-Sequencing Data. Mol Ther Methods Clin Dev. 2018;10:189-96. doi:10.1016/j.omtm.2018.07.003.
28. Liu ZH, Lou HZ, Xie KK, Wang H, Chen N, Aparicio OM, et al. Reconstructing cell cycle pseudo time-series via single-cell transcriptome data. Nature Communications. 2017;8 doi: 10.1038/s41467-017-00039-z
29. Barron M and Li J. Identifying and removing the cell-cycle effect from single-cell RNA-Sequencing data. Sci Rep. 2016;6:33892. doi:10.1038/srep33892.
30. Chhangawala S, Rudy G, Mason CE and Rosenfeld JA. The impact of read length on quantification of differentially expressed genes and splice junction detection. Genome biology. 2015;16:131. doi:10.1186/s13059-015-0697-y.
31. Turman MA, Yabe T, McSherry C, Bach FH and Houchins JP. Characterization of a novel gene (NKG7) on human chromosome 19 that is expressed in natural killer cells and T cells. Hum Immunol. 1993;36 1:34-40.
32. Alessandri L; Cordero F; Beccuti M; Arigoni M; Olivero M; Romano G; Rabellino S; Licheri N; Libero GD; Pace L; Calogero R (2019): Supporting data for "rCASC: reproducible Classification Analysis of Single Cell sequencing data" GigaScience Database. http://dx.doi.org/10.5524/100636

Figure legend

Figure 1: rCASC workflow. Blue boxes indicate preprocessing tools. Yellow boxes define clustering tools. Green box indicates genes-signatures tools.

Figure 2: Cell Stability Score versus Silhouette Score calculated on Pace's dataset (see Supplementary file section 8) using SIMLR over a set of number of clusters ranging between 5 and 8. A) Cell Stability Score violin plot. Mean value and data dispersion suggest that the best number of clusters is 5. Cells remain in the same cluster about 80% of the times, repeating the clustering upon random removal of 10% of the cells. B) Silhouette Score violin plot. Mean value of the SS distribution does not provide clear evidences that one clustering condition is better that another. Furthermore, the dispersion of the SS value shrinks as the number of the clusters increases.

Figure 3: Comparison between the analysis features available in rCASC and in the other single-cell analysis workflows.

Figure 4: rCASC graphical interface within 4seqGUI. A) Counts table generation menu: this set of functions is devoted to the conversion of fastq to a counts table. B) Counts table manipulation menu: this set of functions provides inspection, filtering and normalization of the counts table. C) Clustering menu: these functions allow the use of SIMLR, tSne, Seurat, griph and scanpy to group cells in subpopulations. D) Feature selection menu: this set of functions allows the identification of cluster-specific subsets of genes and their visualization using heatmaps.

Figure 5: *genesUmi* plots the number of detectable genes in each cell (a cell is called present if it is supported by at least N UMI/reads, suggested values are N=3 for UMI or N=5 for smart-seq sequencing [30]) with respect to the number of UMI/cell. *mitoRiboUmi* calculates the percentage of mitochondrial and ribosomal genes with respect to the total number of detected genes in each cell. It plots % of mitochondrial genes with respect to % of ribosomal genes. Furthermore, cells are colored on the basis of the number of detected genes: A) *genesUmi* plot for resting CD8+ T-cells [23], sequencing average 83,000 reads/cell. B) *mitoRiboUmi* plot for resting CD8+ T-cells [23]. The majority of the cells with less than 100 detected genes groups together and they are characterized by high relative percentage of mitochondrial genes and low relative percentage of ribosomal genes. Remaining cells are characterized by few detectable genes, 100÷250 genes/cell, with a percentage of ribosomal genes greater than 30%. C) *genesUmi* plot for Listeria activated CD8+ T-cells [23],

sequencing average 83,000 reads/cell, it is notable the activated cells show a wider range of detectable genes with respect to resting cells (B). D) *mitoRiboUmi* plot for Listeria activated CD8+ T-cells [23]. The majority of the cells are characterized by more the 100 genes and they show low percentage of mitochondrial genes and percentage of ribosomal genes between 15% and 35%. The remaining cells, with less than 100 detected genes groups together and are characterized by high relative percentage of mitochondrial genes and low relative percentage of ribosomal genes.

Figure 6: Heatmap and cell expression plot for prioritized genes. A) Heatmap for the set of 577 genes selected for Pace datasets (see Supplementary file section 8) by SIMLR prioritization. B) Nkg7 CPM expression in the cell clusters. Nkg7 is expressed in activated T-cells (clusters 1, 2, 4, 5) [31] but not in resting T-cells (cluster 3).

Figure 7: Scalability analysis of the clustering tools implemented in rCASC. A) Time required to perform 160 permutations as function of increasing number of cells on approximately 20,000 genes. Left panel: SIMLR, tSne, Seurat and griph clustering up to 5,000 cells was executed on a SeqBox [7] (1 x CPU i7-6770HQ 3.5 GHz (8 cores), 32 GB RAM, 1TB SSD). Right panel: Seurat, griph and scanpy analyses were extended until 101,000 cells using an SGI server (10 x CPU E5-4650 2.4GHz (16 cores), 1TB RAM, 30 TB SATA raid disk). B) Time required to perform 160 permutations as function of increasing number of genes on a set of 800 cells, analysis performed on a SeqBox.

Figure 1

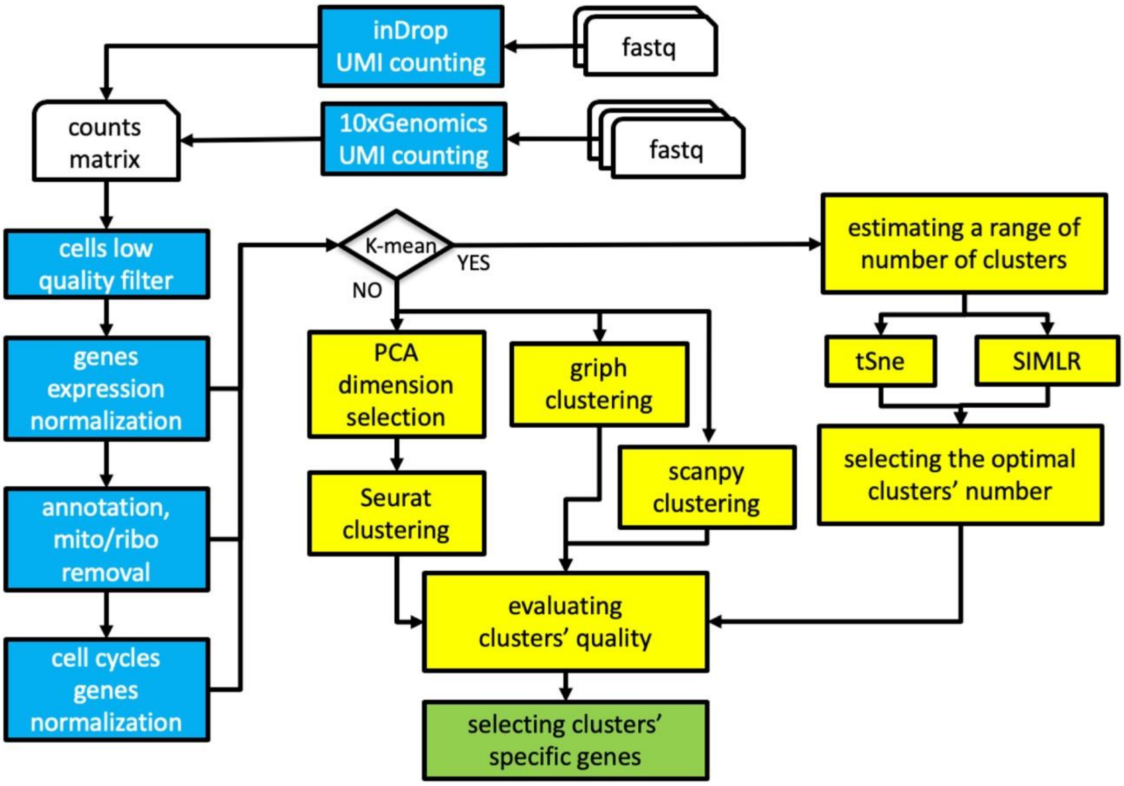
inDrop
UMI counting
fastq
counts
matrix
10xGenomics
UMI counting
fastq
cells low
quality filter
genes
expression
normalization
annotation,
mito/ribo
removal
cell cycles
genes
normalization
K-mean
YES
NO
estimating a range of
number of clusters
tSne
SIMLR
selecting the optimal
clusters' number
PCA
dimension
selection
Seurat
clustering
griph
clustering
scanpy
clustering
evaluating
clusters' quality
selecting clusters'
specific genes

Figure 2

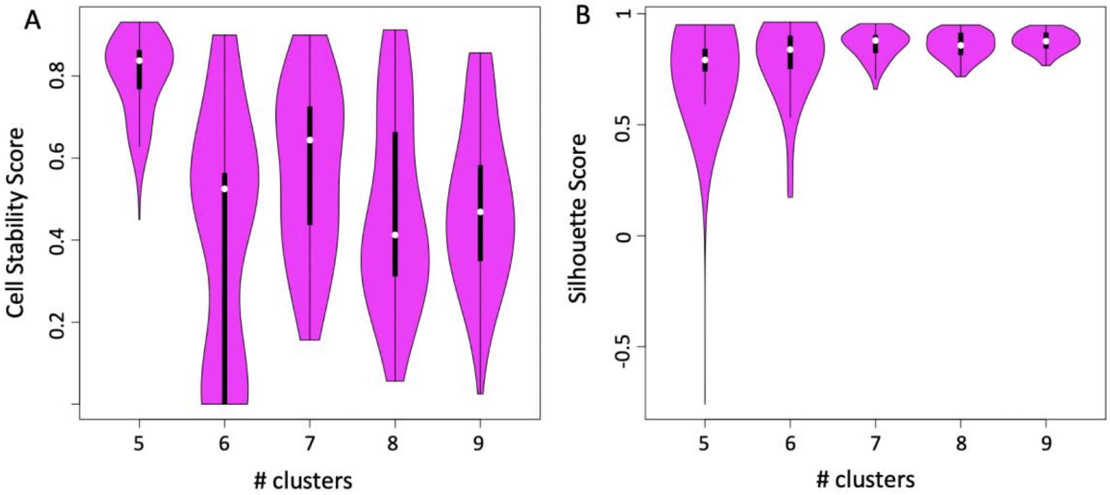

A
Cell Stability Score
0.2
0.4
0.6
0.8
5
6
7
8
9
clusters
B
Silhouette Score
-0.5
0
0.5
1
5
6
7
8
9
clusters

Figure 3

		rCASC (stand alone)	simpleSingleCell (stand alone)	Granatum (web)	Scell (stand alone)	Seurat (stand alone)
**Platforms**		10Xgenomics, inDrop, counts table	counts table	counts table	counts table	counts table
**Tools**	*Fastq conversion in counts table*	Y	-	-	-	-
	Quality Control / Outlier Filtering	Y	-	Y	Y	Y
	Annotation	ENSEMBL ID -> Gene Symbol	-	-	-	-
	Genes filter	Y	-	Y	Y	Y
	Data normalization	Y	Y	Y	Y	Y
	Cell cycle bias removal	Y	-	-	Y	Y
	Data dimensionality reduction	Y	Y	Y	Y	Y
	Supported clustering methods	tSne, SIMLR, Seurat (PCA), griph, scanpy (UMAP)	Walktrap	Non-negative matrix factorization, K-mean (Euclidean), K-mean (tSne)	PCA, k-means, Gaussian mixture, Minkowski weighted k-means, DBSCAN	PCA, tSne, ica, dmap
	Cluster quality score	Silhuette, Cell Stability Score	-	-	-	-
	Features selection and visualization	Y	Y	Y	-	-
	Supported methods	ANOVA-like (edgeR), SIMLR and Seurat genes prioritization	filtering on expression	NODES, SCDE, EdgeR, Limma	-	wilcox, bimod, roc, t-test, tobit, negbinom, MAST, DESeq2
	Biological inference	-	-	Y	-	-
**Reproducibility**	*Functional reproducibility*	Y	Y	-	-	Y
	Computational reproducibility	Y	-	Y	Y	-
**Flexibility**	*line command execution*	Y	Y	-	-	Y
	graphical interface	Y	-	Y	Y	-

Figure 4

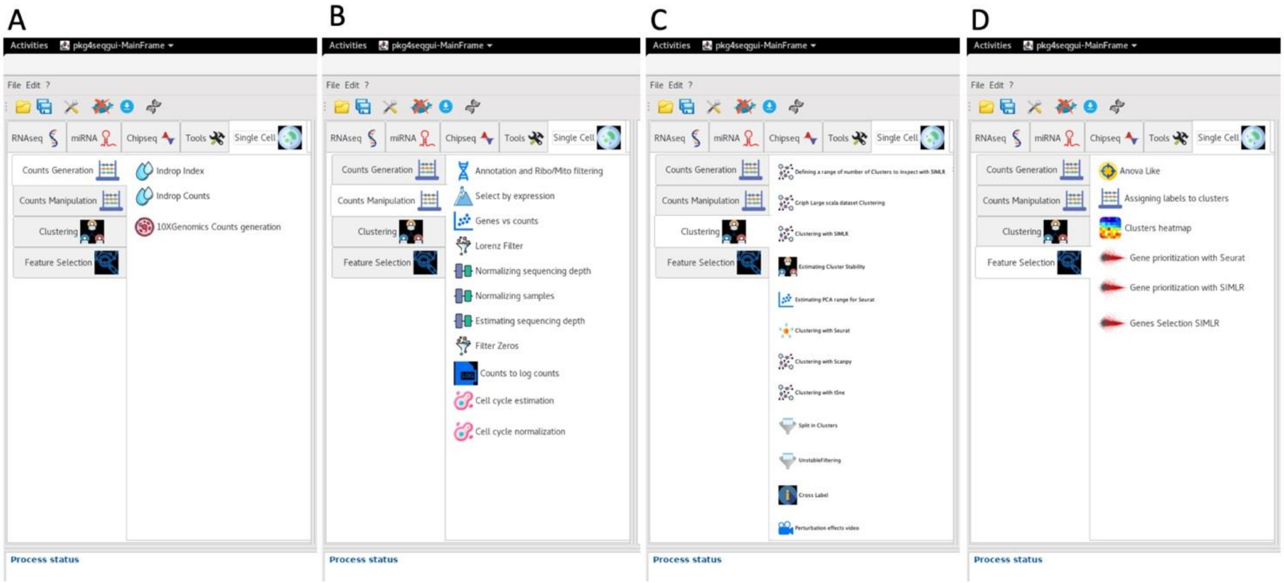
A
B
C
D
Activities
pkg4seqgui-MainFrame
File Edit ?
RNAseq
miRNA
Chipseq
Tools
Single Cell
Counts Generation
Counts Manipulation
Clustering
Feature Selection
Indrop Index
Indrop Counts
10XGenomics Counts generation
Annotation and Ribo/Mito filtering
Select by expression
Genes vs counts
Lorenz Filter
Normalizing sequencing depth
Normalizing samples
Estimating sequencing depth
Filter Zeros
Counts to log counts
Cell cycle estimation
Cell cycle normalization
Defining a range of number of Clusters to inspect with SIMLR
Griph Large scala dataset Clustering
Clustering with SIMLR
Estimating Cluster Stability
Estimating PCA range for Seurat
Clustering with Seurat
Clustering with Scanpy
Clustering with tSne
Split in Clusters
UnstableFiltering
Cross Label
Perturbation effects video
Anova Like
Assigning labels to clusters
Clusters heatmap
Gene prioritization with Seurat
Gene prioritization with SIMLR
Genes Selection SIMLR
Process status

Figure 5

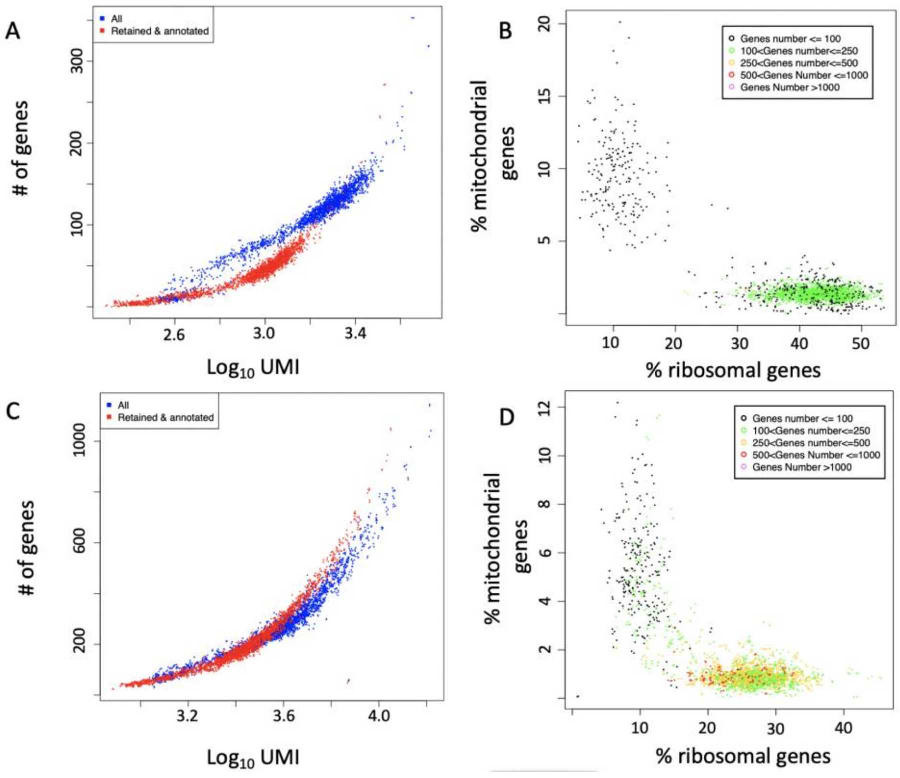

A
All
Retained & annotated
# of genes
Log10 UMI
B
% mitochondrial genes
% ribosomal genes
Genes number <= 100
100<Genes number<=250
250<Genes number<=500
500<Genes Number <=1000
Genes Number >1000
C
All
Retained & annotated
# of genes
Log10 UMI
D
% mitochondrial genes
% ribosomal genes
Genes number <= 100
100<Genes number<=250
250<Genes number<=500
500<Genes Number <=1000
Genes Number >1000

Figure 6

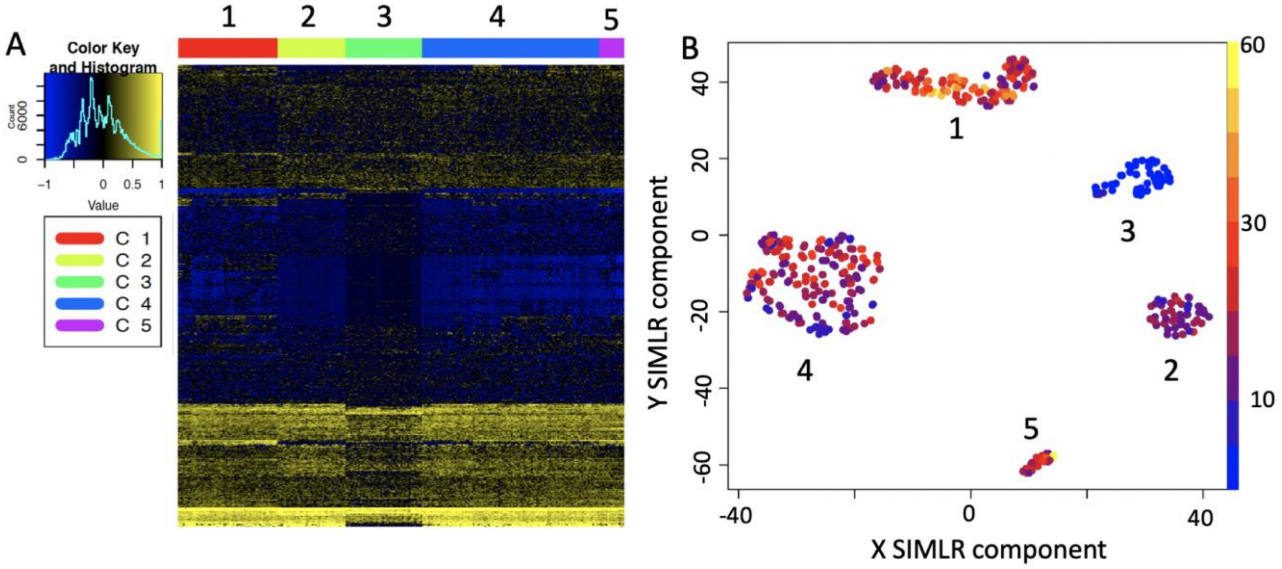
A
Color Key and Histogram
Count
6000
0
-1
0
0.5
1
Value
C 1
C 2
C 3
C 4
C 5
1
2
3
4
5
B
Y SIMLR component
X SIMLR component
40
20
0
-20
-40
-60
-40
0
40
60
30
10
1
2
3
4
5

Figure 7

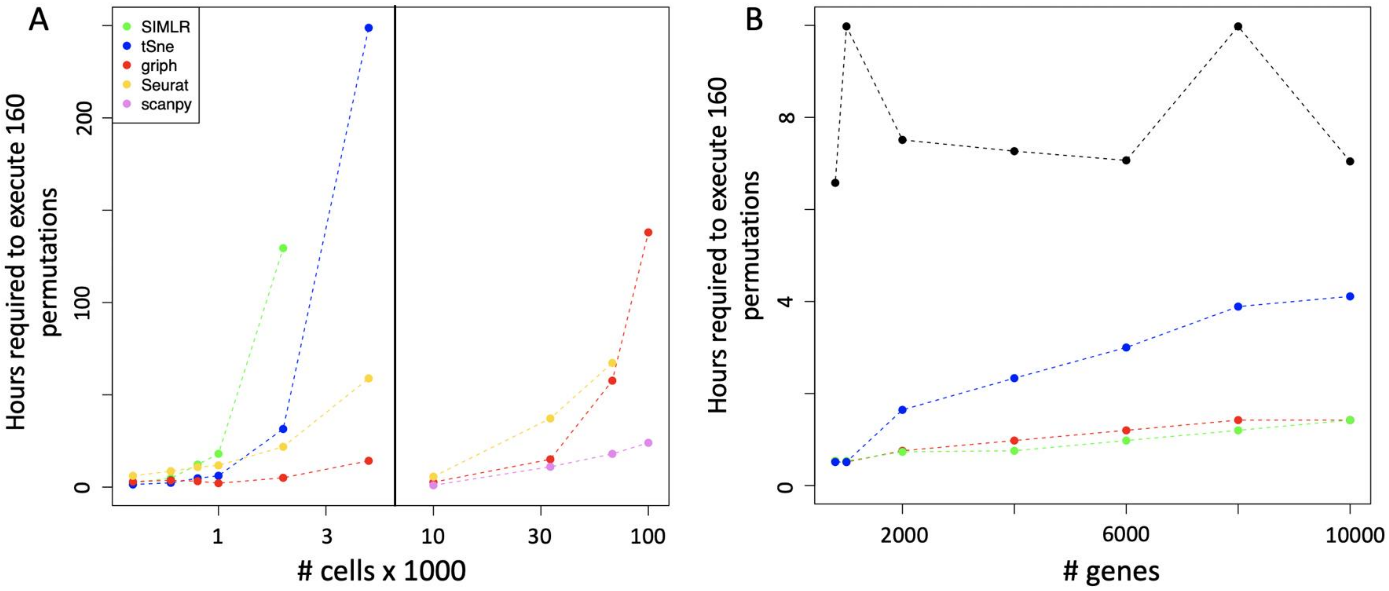
A
SIMLR
tSne
griph
Seurat
scanpy
Hours required to execute 160 permutations
0
100
200
1
3
10
30
100
cells x 1000
B
Hours required to execute 160 permutations
0
4
8
2000
6000
10000
genes

 Click here to access/download;Figure;AllFigures.pdf

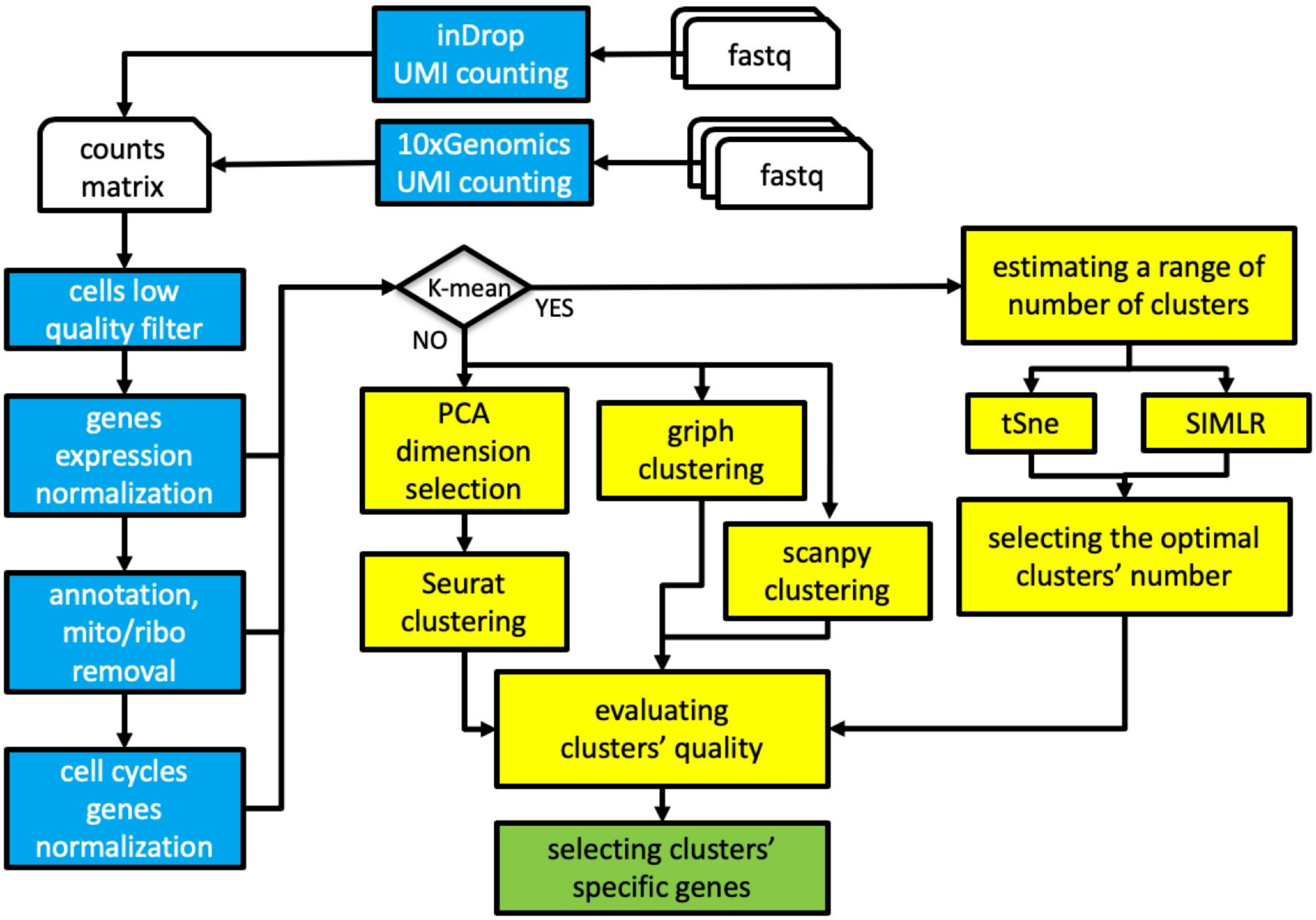

Fig.1

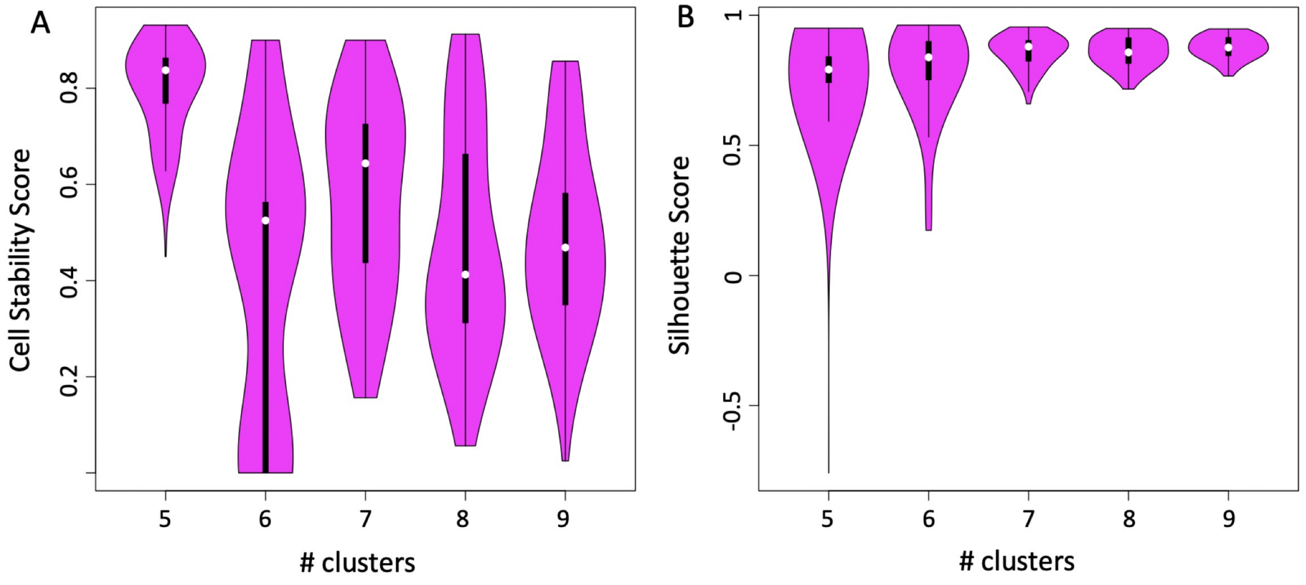

Fig.2

		rCASC (stand alone)	simpleSingleCell (stand alone)	Granatum (web)	Scell (stand alone)	Seurat (stand alone)
**Platforms**		10Xgenomics, inDrop, counts table	counts table	counts table	counts table	counts table
**Tools**	*Fastq conversion in counts table*	Y	-	-	-	-
	Quality Control / Outlier Filtering	Y	-	Y	Y	Y
	Annotation	ENSEMBL ID -> Gene Symbol	-	-	-	-
	Genes filter	Y	-	Y	Y	Y
	Data normalization	Y	Y	Y	Y	Y
	Cell cycle bias removal	Y	-	-	Y	Y
	Data dimensionality reduction	Y	Y	Y	Y	Y
	Supported clustering methods	tSne, SIMLR, Seurat (PCA), griph, scanpy (UMAP)	Walktrap	Non-negative matrix factorization, K-mean (Euclidean), K-mean (tSne)	PCA, k-means, Gaussian mixture, Minkowski weighted k-means, DBSCAN	PCA, tSne, ica, dmap
	Cluster quality score	Silhuette, Cell Stability Score	-	-	-	-
	Features selection and visualization	Y	Y	Y	-	-
	Supported methods	ANOVA-like (edgeR), SIMLR and Seurat genes prioritization	filtering on expression	NODES, SCDE, EdgeR, Limma	-	wilcox, bimod, roc, t-test, tobit, negbinom, MAST, DESeq2
	Biological inference	-	-	Y	-	-
**Reproducibility**	*Functional reproducibility*	Y	Y	-	-	Y
	Computational reproducibility	Y	-	Y	Y	-
**Flexibility**	*line command execution*	Y	Y	-	-	Y
	graphical interface	Y	-	Y	Y	-

Fig.3

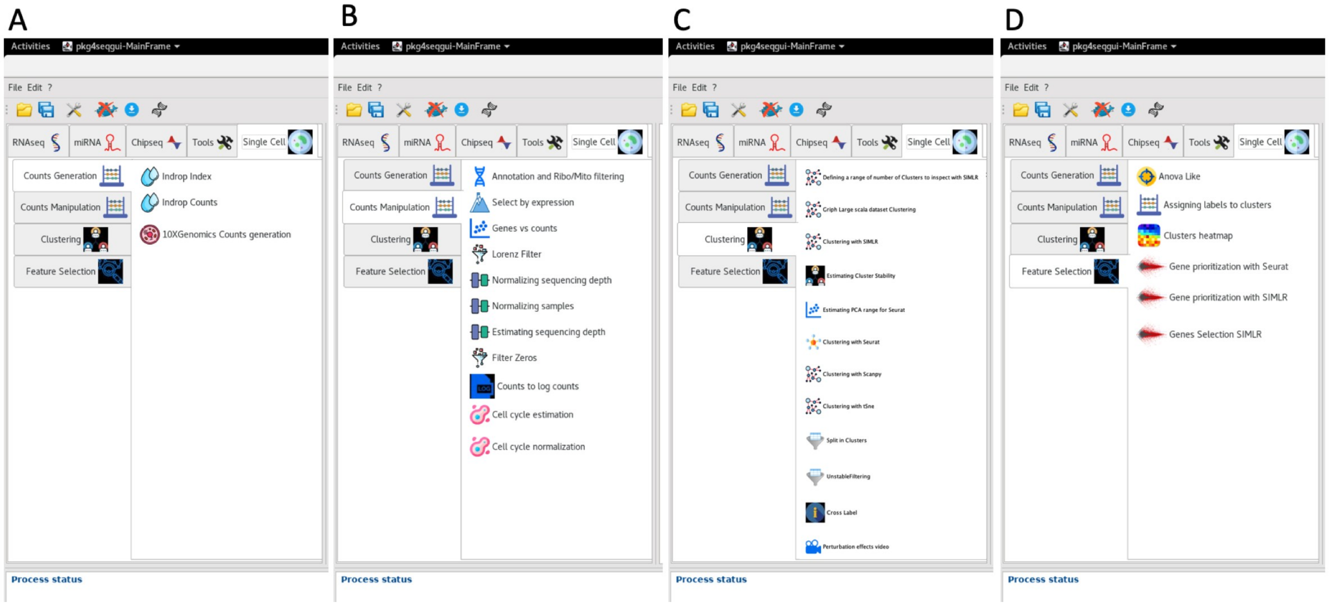

Fig.4

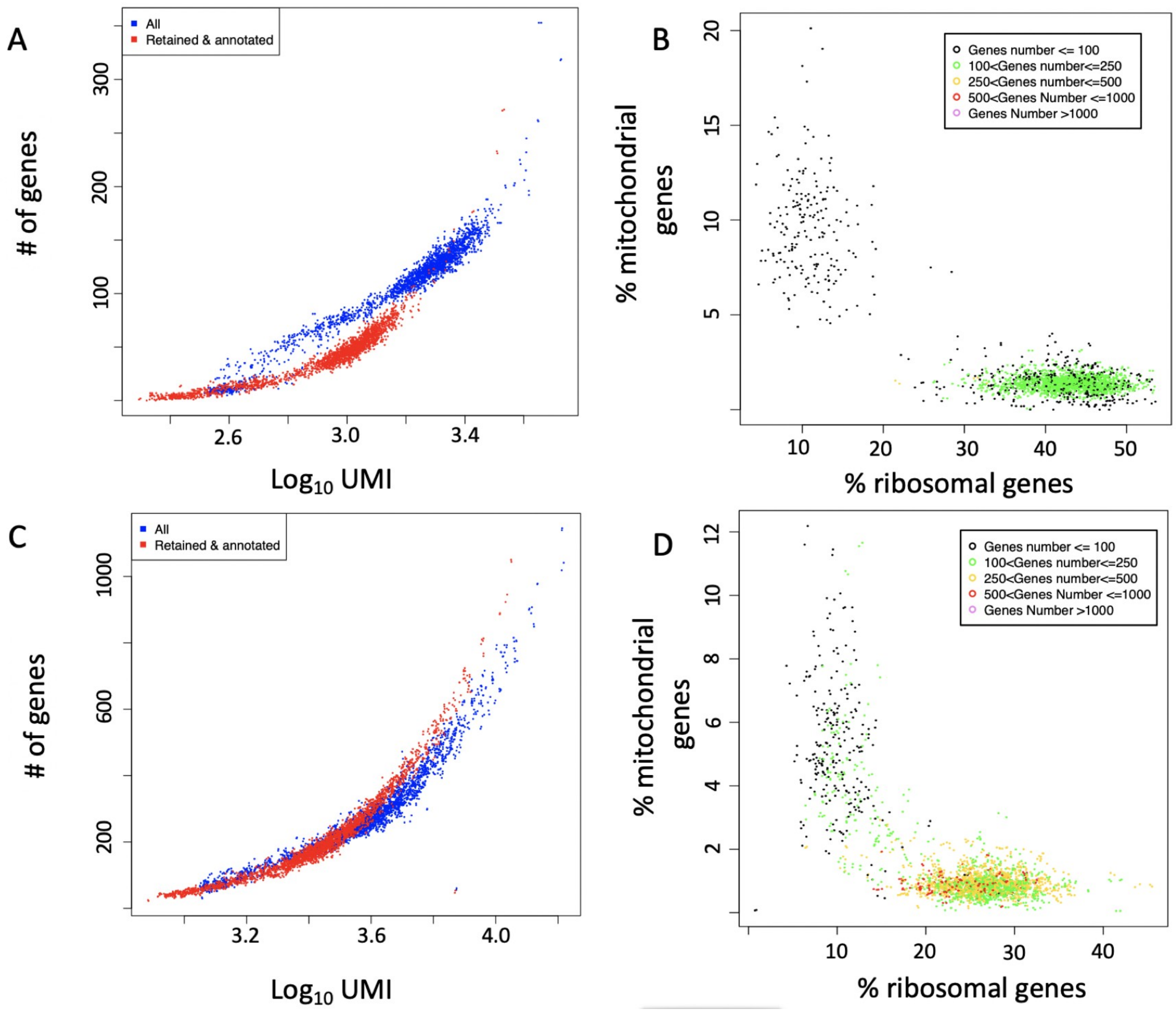
A
All
Retained & annotated
# of genes
Log10 UMI
B
Genes number <= 100
100<Genes number<=250
250<Genes number<=500
500<Genes Number <=1000
Genes Number >1000
% mitochondrial genes
% ribosomal genes
C
All
Retained & annotated
# of genes
Log10 UMI
D
Genes number <= 100
100<Genes number<=250
250<Genes number<=500
500<Genes Number <=1000
Genes Number >1000
% mitochondrial genes
% ribosomal genes

Fig.5

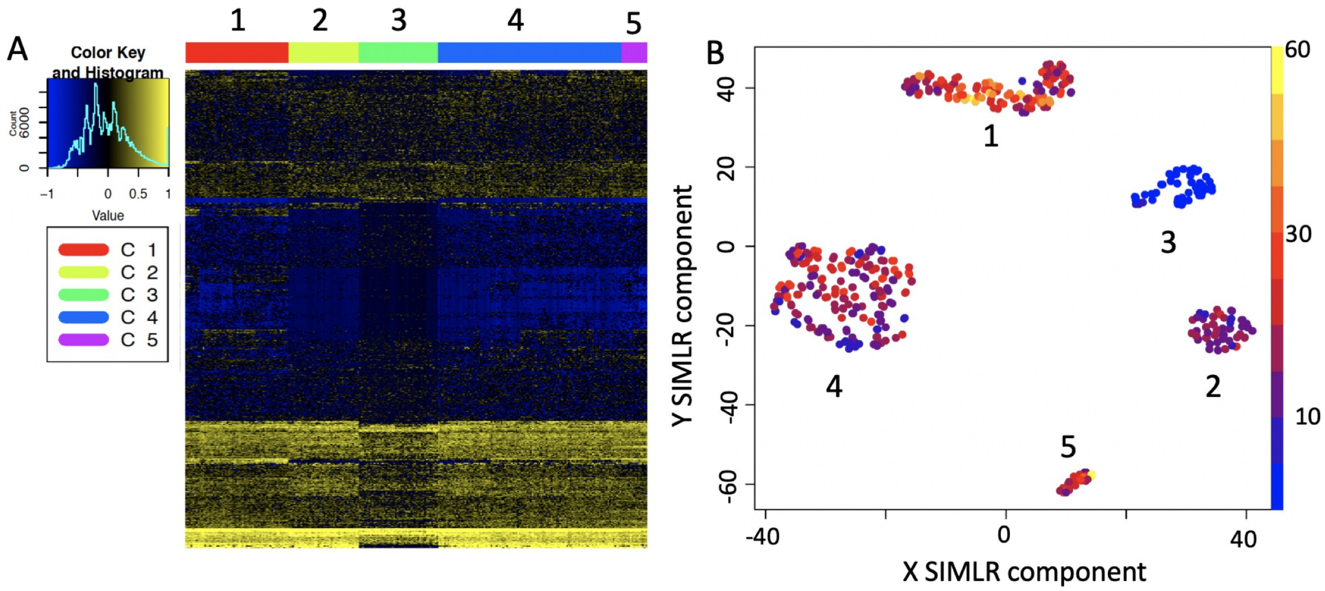
A
Color Key and Histogram
Count
Value
C 1
C 2
C 3
C 4
C 5
1
2
3
4
5
B
Y SIMLR component
X SIMLR component
60
30
10
Fig.6

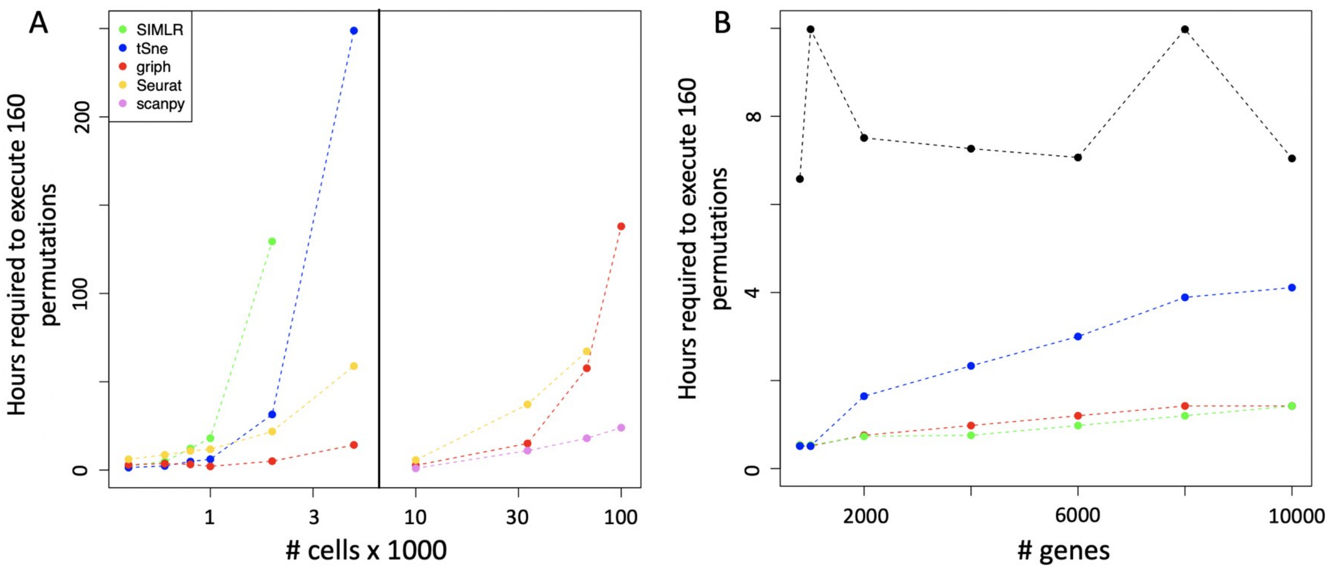
A
SIMLR
tSne
griph
Seurat
scanpy
Hours required to execute 160 permutations
cells x 1000
B
genes
Fig.7

Click here to access/download
Supplementary Material
rCASC_supplementary_file.pdf

Rebuttal Letter Click here to access/download;Personal Cover;rebuttal3.pdf

Rebuttal letter for the paper:

rCASC: reproducible Classification Analysis of Single Cell sequencing data.

Luca Alessandrì, Francesca Cordero, Marco Beccuti, Maddalena Arigoni, Martina Olivero, Greta Romano, Sergio Rabellino,Nicola Licheri, Gennaro De Libero, Luigia Pace and Raffaele A Calogero

Dear Editor,

First of all, we wish to thank the reviewers for their valuable comments and useful suggestions which helped us to substantially improve the paper and its associated tool.

Hereafter we report our answers to the reviews' comments.

Reviewer reports:

Reviewer #1: The authors incorporated additional clustering methods (Scanpy and Griph) that prove to be scalable for datasets having larger sizes which corresponds to the field needs.
In particular, Scanpy seems to reveal no issue to scale up to 100K cells in the benchmark executed opposite to the other methods.
I recommend accepting this manuscript since I think it is well suited for current and future analytical needs for single cells.

Minor comments:

Question 1: Is there any limitation or trick to use for the preprocessing procedures (low cell quality filter, normalization, annotation, cell cycle removal, matrix creation) executed before the clustering when increasing the sample / feature size?
I presume no because the authors have used them with large dataset. Then, It will be worth metioning that in the manuscript with a brief estimate of the computational time / memory needed.

Answer 1: All samples were preprocessed removing ribosomal/mitochondrial protein genes and cells with a total count of UMIs lower than 100. This information was added in the scalability paragraph: "All the above samples were preprocessed removing ribosomal/mitochondrial protein genes and cells with a total count of UMIs lower than 100."
Concerning the computational time/memory required for the analysis we added the following phrase at the end of Scalability paragraph:
"The definition of the computing time for an analysis depends on multiple parameters: i) the number of permutations performed in parallel, ii) the number of cells under analysis, iii) the clustering tool in use and iv) the hardware used for the analysis. Concerning the amount of RAM required for each permutation run in parallel, up to 5000 cells the maximum amount of RAM required is approximately 4 GB, from 10000 to 100000 cells, the maximum RAM required is approximately 20 GB. Independently by the clustering approach and the size of the dataset, we suggest to run at least 100 permutations to correctly estimate CSS."

Question 2: The figure 3 is not updated with Scanpy and griph.

Answer2: We updated Fig. 3 as suggested by the reviewer. Moreover, we updated Fig. 4C which now includes griph and scanpy functions

Question 3: I don't understand the use of the term hierarchical clustering in the manuscript and in the suppl. material.

Answer 3: We removed the term “hierarchical” from Fig. 1 and in supplementary data.
