# Supplementary material for: rCASC: reproducible classification analysis of single-cell sequencing data
Source: Gigascience. 2019 Sep 8;8(9):giz105. doi: 10.1093/gigascience/giz105 (PMC6732171; doi:10.1093/gigascience/giz105)
Supplement: giz105_Supplemental_File [file giz105_supplemental_file.pdf]

rCASC Supplementary data

Luca Alessandrì, Francesca Cordero, Marco Beccuti, Maddalena Arigoni, Martina Olivero, Greta Romano, Sergio Rabellino, Nicola Licheri, Gennaro De Libero, Luigia Pace, and Raffaele A Calogero

30/07/2019

Contents

Section 1 rCASC: a single cell analysis workflow designed to provide data reproducibility 3

Section 1.1 Minimal hardware requirements to run rCASC 4

Section 1.2 Installation 4

Section 2 Counts generation 5

Section 2.1 inDrop seq 6

Section 2.2 10XGenomics 8

Section 2.3 Smart-seq full transcript sequencing. 10

Section 3 Counts matrix manipulation 10

Section 3.1 Removing non informative genes 11

Section 3.2 Plotting genes numbers versus total UMIs/reads in each cell 13

Section 3.3 Identifying and removing cell low-quality outliers 19

Section 3.4 Annotation and mitochondrial/ribosomal protein genes removal 21

Section 3.5 Top expressed genes 23

Section 3.6 Data normalization 24

Section 3.7 Log conversion of a count table 29

Section 3.8 Detecting and removing cell cycle bias 29

Section 4 Estimating the number of clusters to be used for cell sub-population discovery. 33

Section 4.1 Estimating the number of cluster to be used for cell sub-population discovery by community detection method. 34

Section 5 K-mean clustering: detecting cell sub-populations by mean of kernel based similarity learning (*SIMLR*). 40

Section 5.1 Cell Stability Score: mathematical description. 41

Section 5.2 Visualizing the cell clusters relocation during bootstraps. 52
Section 5.3 Estimating cluster stability. 54
Section 6 Clustering (autonomously detecting the number of clusters required for data partitioning): *Seurat*, *griph* and *scanpy* graph-based clustering. 56
Section 6.1: *Seurat* 56
Section 6.2: *griph* 59
Section 6.3: *scanpy* 60
Section 6.4 Comparing SIMLR, tSne, Seurat, griph and scanpy clustering. 61
Section 7 Detecting the genes playing the major role in clusters formation 65
Section 7.1 A statistical approach to select genes affecting clusters formation: EdgeR ANOVA-like 65
Section 7.2 A machine learning approach: SIMLR genes prioritization 70
Section 7.3 Seurat genes prioritization 75
Section 8 Supporting tools 77
Section 9 An example of rCASC analysis: *GEO:GSE106264*. 80
Section 9.1 Selecting subsets of cells with the highest number of called genes. 80
Section 9.2 Improving clustering results 84
Section 9.3 Detecting the genes playing the major role in clusters generation: EdgeR ANOVA-like analysis 85
Section 10 Tips and tricks 87
Section 10.1 Data preprocessing 87
Section 10.2 Clustering 88

Contents

Section 1 rCASC: a single cell analysis workflow designed to provide data reproducibility

Since the end of the 90's omics high-throughput technologies have generated an enormous amount of data, reaching today an exponential growth phase. Analysis of omics big data is a revolutionary means of understanding the molecular basis of disease regulation and susceptibility, and this resource is accessible to the biological/medical community via bioinformatics frameworks. However, because of the fast evolution of computation tools and omics methods, the *reproducibility crisis* is becoming a very important issue [*Nature, 2018*] and there is a mandatory need to guarantee robust and reliable results to the research community [*Global Engage Blog*].

Our group is deeply involved in developing workflows that guarantee both **functional** (i.e. the information about data and the utilized tools are saved in terms of meta-data) and **computation** reproducibility (i.e. the real image of the computation environment used to generate the data is stored). For this reason, we are managing a bioinformatics community called *reproducible-bioinformatics.org* [*Kulkarni et al.*] designed to provide to the biological community a reproducible bioinformatics ecosystem [*Beccuti et al.*].

rCASC, reproducible Cluster Analysis of Single Cells, is part of the *reproducible-bioinformatics.org* project and provides single cell analysis functionalities within the reproducible rules described by Sandve et al. [*PLoS Comp Biol. 2013*]. rCASC is designed to provide a workflow (Figure 1) for cell-subpopulation discovery.

The workflow allows the direct analysis of fastq files generated with *10X Genomics platform*, *InDrop technology* or a count matrix having as column-names cells identifier and as row names ENSEMBL gene annotation. In the following paragraphs the functionalities of rCASC workflow are described.

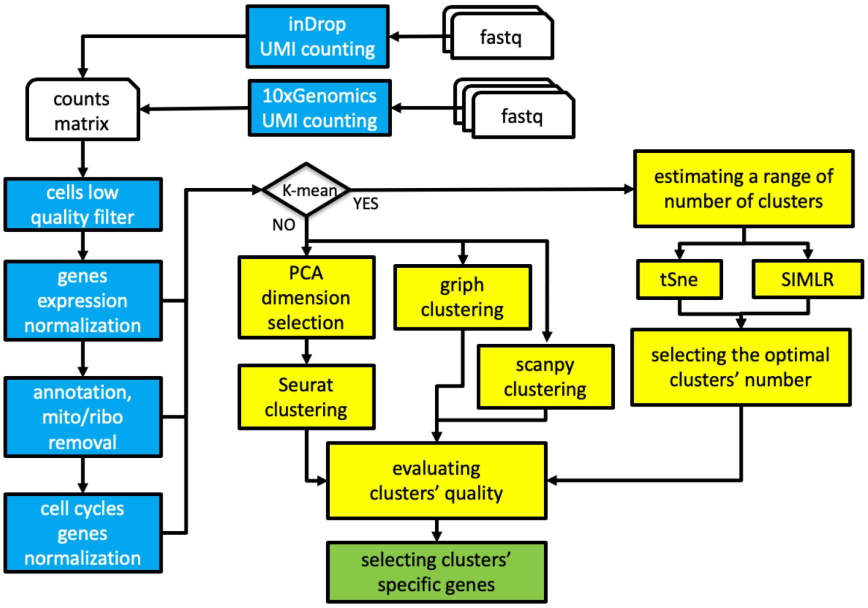

Figure 1: rCASC workflow

Section 1.1 Minimal hardware requirements to run rCASC

The RAM and CPU requirements are dependent on the dataset under analysis, e.g. up to 2000 cells can be effectively analyzed using the hardware described by Beccuti [*Bioinformatics2018*]:

- 32 Gb RAM
- 2.6 GHz Core i7 6700HQ with 8 threads.
- 500 GB SSD

The analysis time can be significantly improved increasing the number of cores. Implementation of the workflow for computers farm, using *swarm*, is under implementation.

Section 1.2 Installation

The minimal requirements for installation are:

- A workstation/server running 64 bits Linux.
- Docker daemon installed on the machine, for more info see this document:
  - *https://docs.docker.com/engine/installation/*.
- A scratch folder, e.g. /data/scratch, possibly on a fast I/O SSD disk, writable by everybody:

```
chmod 777 /data/scratch
```

The functions in rCASC package require that user belongs to a *group* with the rights to execute docker. See the following document for more info: *https://docs.docker.com/install/linux/linux-postinstall/*

The following commands allow the rCASC installation in *R* environment:

```r
install.packages("devtools")
library(devtools)
install_github("kendomaniac/rCASC")

# downloading the required docker containers
library(rCASC)
downloadContainers()
```

Section 2 Counts generation

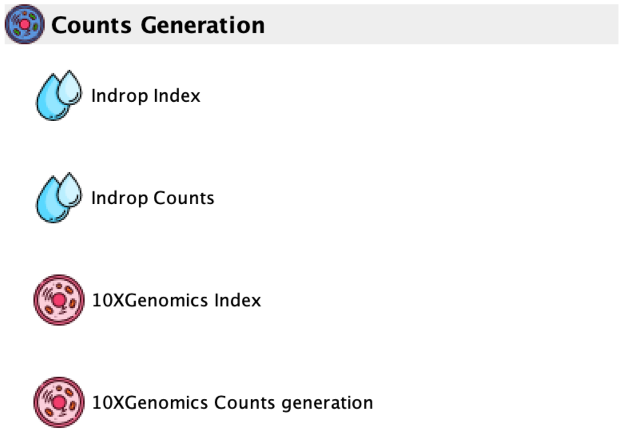

Figure 2: GUI: Counts generation Menu

This session refers to the generation of a counts table starting from fastq files generated by inDrop and 10XGenomics platforms.

Section 2.1 inDrop seq

inDrop single-cell sequencing approach was originally published by Klein [*Cell 2015*]. Then, the authors published the detailed protocol in [*Zilionis et al. Nature Protocols 2017*], which has different primer design comparing to the original paper (Figure 3).

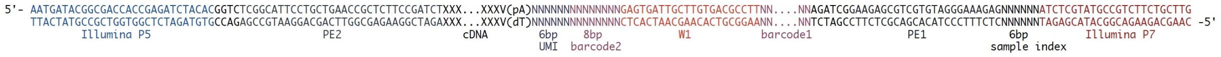

Figure 3: inDrop library structure

The analysis shown below is based on the protocol in Zilionis [*Nature Protocols 2017*], which is the version 2 (Figure 4) of the inDrop technology, actually distributed by *1CellBio*.

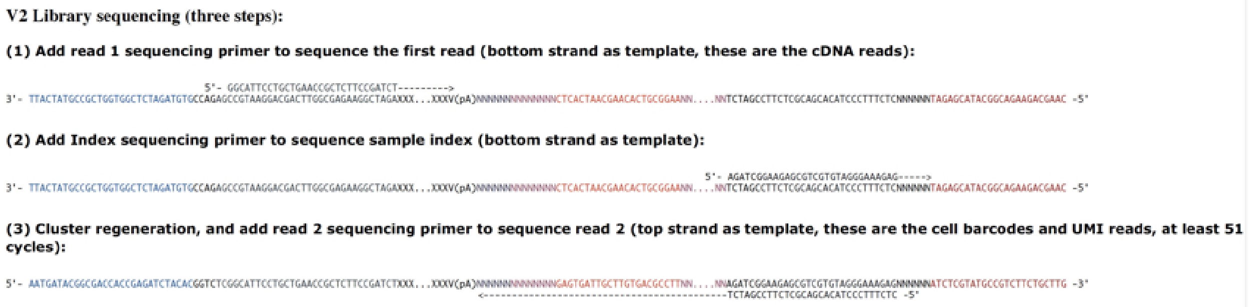

Figure 4: inDrop v2

Section 2.1.1 inDrop data analysis

There are two main functions, **indropIndex** and **indropCounts**, allowing the generation of a counts table starting from fastq files.

Section 2.1.1.1 indropIndex: Creates a reference genome for inDrop V2

- *Parameters* (only those without default; for the full list of parameters please refer to the function help) (Figure 5A):
  - *index.folder*: the folder where the reference genome will be created
  - *ensembl.urlgenome*: the link to the ENSEMBL unmasked genome sequence of interest.
  - *ensembl.urlgtf*: the link to the ENSEMBL GTF of the genome of interest.

```
library(rCASC)
#running indropCounts index build
indropIndex(group="docker", index.folder=getwd(),
 ensembl.urlgenome="ftp://ftp.ensembl.org/pub/release-87/fasta/mus_musculus/dna/Mus_musculus.GRCm38.dna.toplevel.fa.gz",
 ensembl.urlgtf="ftp://ftp.ensembl.org/pub/release-87/gtf/mus_musculus/Mus_musculus.GRCm38.87.gtf.gz")
```

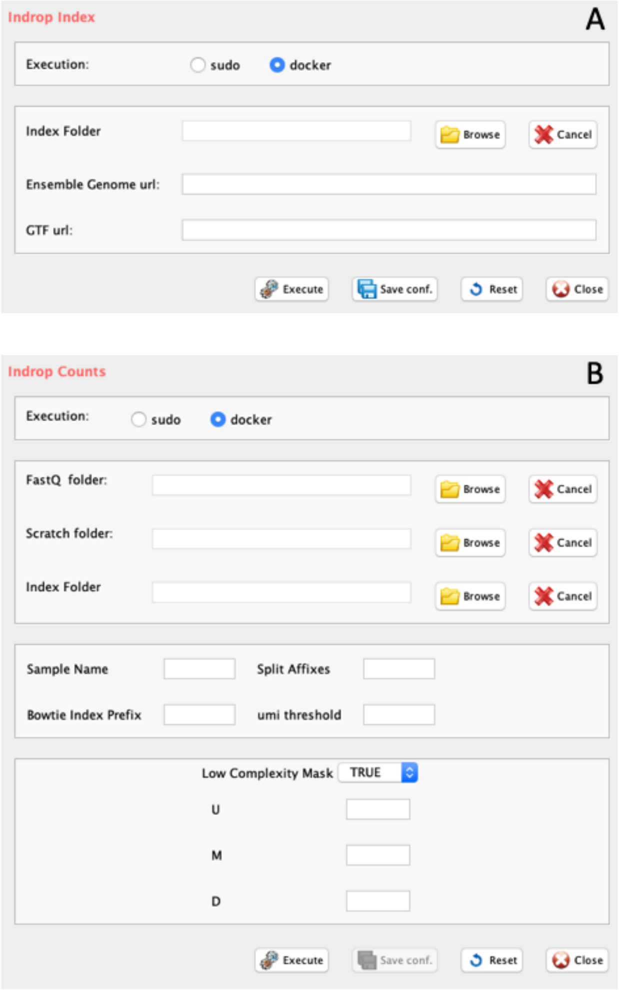

Figure 5: GUI: inDrop related panels: A) inDrop Index generation, B) inDrop counts table generation

Section 2.1.1.2 indropCounts: Converts fastq in UMI counts using *inDrop workflow*

- *Parameters* (only those without default; for the full list of parameters please refer to the function help) (Figure 5B):
  - *scratch.folder*, a character string indicating the path of the scratch folder
  - *fastq.folder (GUI FastQ folder)*, a character string indicating the folder where input data are located and where output will be written
  - *index.folder (GUI Index Folder)*, a character string indicating the folder where transcriptome index was created with indropIndex.
  - *split.affixes*, the string separating SAMPLENAME from the Rz_001.fastq.gz, e.g. S0_L001.
  - *bowtie.index.prefix*, the prefix name of the bowtie index. If genome was generated with indropIndex function the bowtie index is genome (default).
  - *M*, integer. Ignore reads with more than M alignments, after filtering on distance from transcript end, suggested value 10

  - *U*, integer. Ignore counts from UMI that should be split among more than U genes, suggested value 2
  - *D*, integer. Maximal distance from transcript end, suggested value 400.
  - *low.complexity.mask*, boolean, masking low complexity regions

```
# Example is part of an unpublished mouse blood cells
system("wget 130.192.119.59/public/testMm_S0_L001_R1_001.fastq.gz")
system("wget 130.192.119.59/public/testMm_S0_L001_R2_001.fastq.gz")
library(rCASC)
indropCounts(group="docker", scratch.folder="/data/scratch", fastq.folder=getwd(),
        index.folder="/data/genomes/indropMm10", sample.name="testMm", split.affixes="S0_L001",
        bowtie.index.prefix="genome", M=10, U=2, D=400, low.complexity.mask="False")
```

Section 2.2 10XGenomics

The rCASC function, **cellrangerCount** allows the generation of a counts table starting from fastq files. This function implements *Cellranger*, the 10xGenomics tool allowing the conversion of the fastqs, generated with 10XGenomics platform, into a count matrix.

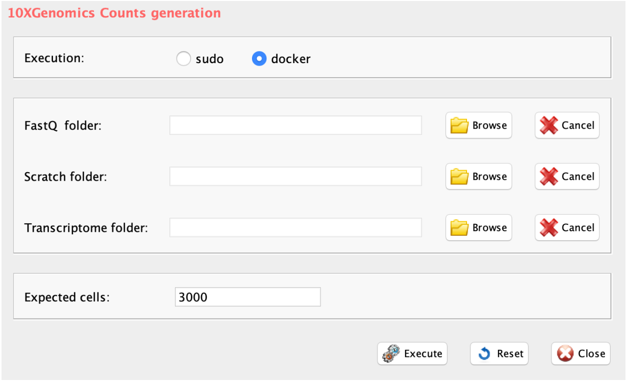

Figure 6: GUI: 10XGenomics counts table generation

- *Parameters* (only those without default; for the full list of parameters please refer to the function help) (Figure 6):
  - *fastq (GUI FastQ folder)*, the path to the folder, where 10XGenomics fastq.gz files are located.
  - *transcriptome (GUI Transcriptome folder)*, the path to the Cellranger compatible transcriptome reference
  - *scratch.folder (GUI Scratch folder)*, a character string indicating the path of the scratch folder
  - *expect.cells (GUI Expected cells)*, optional setting the number of recovered cells. Default: 3000 cells

```
home <- getwd()
library(rCASC)
downloadContainers()
setwd("/data/genomes/cellranger_hg19mm10")
```

```
#getting the human and mouse cellranger index
system("wget http://cf.10xgenomics.com/supp/cell-exp/refdata-cellranger-hg19-and-mm10-2.1.0.tar.gz")
setwd(home)
# downloading 100 cells 1:1 Mixture of Fresh Frozen Human (HEK293T) and Mouse (NIH3T3) Cells
system("wget http://cf.10xgenomics.com/samples/cell-exp/2.1.0/hgmm_100/hgmm_100_fastqs.tar")
system("tar xvf hgmm_100_fastqs.tar")
# The cellranger analysis is run without the generation of the secondary analysis
cellrangerCount(group="docker",  transcriptome.folder="/data/genomes/cellranger_hg19mm10",
                fastq.folder="/data/test_cell_ranger/fastqs",  expect.cells=100,
                nosecondary=TRUE, scratch.folder="/data/scratch")
```

The analysis done above took 56.4 mins on a *SeqBox*, equipped with an Intel i7-6770HQ (8 threads), 32 Gb RAM and 500Gb SSD.

The output of the above analysis are two counts matrices **results_cellranger.cvs** and **results_cellranger.txt** and a folder called **results_cellranger**, which contains the full cellranger output, more information on cellranger output can be found at *10XGenomics web site.*

Genome indexes can be retrieved from 10Xgenomics repository

```
setwd("/data/genomes/cellranger_hg38")
#getting the hg38 human genome cellranger index
system("wget  http://cf.10xgenomics.com/supp/cell-exp/refdata-cellranger-GRCh38-3.0.0.tar.gz")
setwd("/data/genomes/cellranger_hg19")
#getting the hg19 human genome cellranger index
system("wget  http://cf.10xgenomics.com/supp/cell-exp/refdata-cellranger-hg19-3.0.0.tar.gz")
setwd("/data/genomes/cellranger_mm10")
#getting the mm10 mouse genome cellranger index
system("wget http://cf.10xgenomics.com/supp/cell-exp/refdata-cellranger-mm10-3.0.0.tar.gz")
setwd("/data/genomes/cellranger_hg19mm10")
#getting the human and mouse cellranger index
system("wget http://cf.10xgenomics.com/supp/cell-exp/refdata-cellranger-hg19-and-mm10-2.1.0.tar.gz")
```

or can be generated using the **cellrangeIndexing**

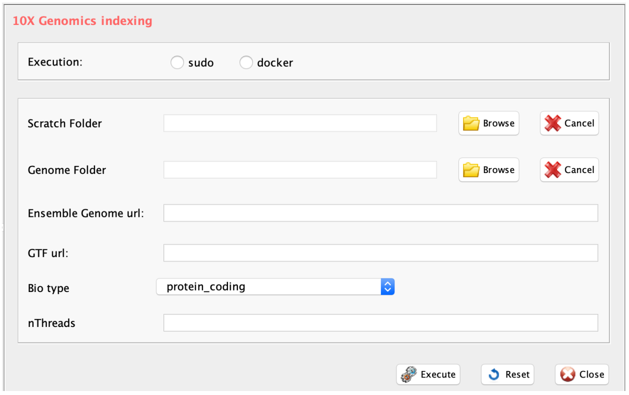

Figure 7: GUI: 10XGenomics genome indexing

- *Parameters* (only those without default; for the full list of parameters please refer to the function help):
  - *group*, a character string. two options: sudo or docker, depending to which group the user belongs
  - *scratch.folder*, a character string indicating the path of the scratch folder
  - *genomeFolder*, path for the genome folder
  - *gtf.url*, url for ENSEMBL gtf download
  - *fasta.url*, url for genome assembly fasta download
  - *bio.type*, ENSEMBL biotype to select the subset of genes of interest from the ENSEMBL gtf

```
library(rCASC)
setwd("/data/genomes/hg38refcellranger")
cellrangerIndexing(group="docker", scratch.folder="/data/scratch",
  gtf.url="ftp://ftp.ensembl.org/pub/release-87/gtf/homo_sapiens/Homo_sapiens.GRCh38.87.gtf.gz",
  fasta.url=
    "ftp://ftp.ensembl.org/pub/release-87/fasta/homo_sapiens/dna/Homo_sapiens.GRCh38.dna.toplevel.fa.gz
  genomeFolder = getwd(), bio.type="protein_coding", nThreads = 8)
```

Section 2.3 Smart-seq full transcript sequencing.

Smart-seq protocol generates a full transcript library for each cell, i.e. a fastq file for each cell. To convert fastq in counts we suggest to use **rnaseqCounts** or **wrapperSalmon** counts from *docker4seq* package [*Kulkarni et al.*]. Both above-mentioned functions are compliant with minimal hardware requirements indicated for rCASC and are part, as rCASC, of the *Reproducible Bioinformatics Project*. **rnaseqCounts** is a wrapper executing on each fastq:

- quality evaluation of fastq with *FastQC* software,
- trimming of adapters with *skewer*,
- mapping reads on genome using *STAR* and counting isoforms and genes with *RSEM*.

wrapperSalmon instead implements FastQC and skewer and calculates isoforms and genes counts using *Salmon* software.

Section 3 Counts matrix manipulation

This paragraph describes a set of functions that can be used to remove low quality cells and non-informative genes from counts tables generated by any single-cell sequencing platform, Fig. 8.

- **Counts manipulation**:
  - Plotting detected genes versus total number of UMIs (Unique Molecular Identifier)/reads: **mitoRiboUmi**, **genesUmi**
  - ENSEMBL annotation and genes filtering: **scannobyGtf**

– Removing low quality cells: **lorenzFilter**
– Selecting the top X variable/expressed genes: **topx**
– Removing non informative genes: **filterZeros**
– Converting a count table in log10: **counts2log**
– Data normalization: **scnorm**, minimal requirements 10K counts/cell, works best with whole transcript sequencing
– Data normalization: **umiNorm**, global normalization methods TMM and RLE are suitable for UMI data
– Removing cell cycle bias: **recatPrediction/ccremove**

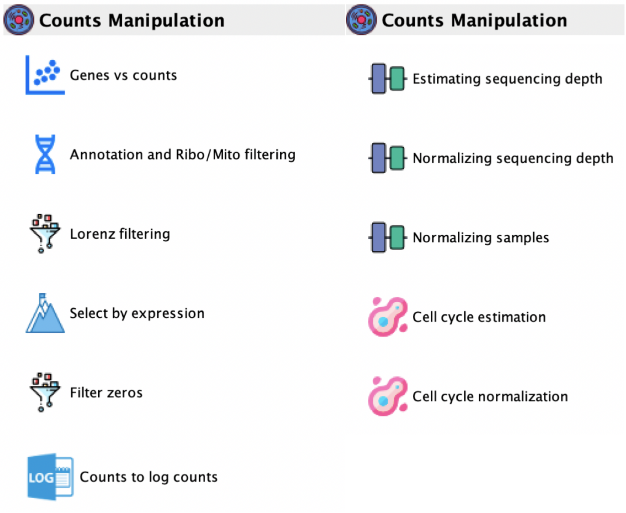

Figure 8: GUI: Counts manipulation menu

Section 3.1 Removing non informative genes

The function **filterZeros** retains all genes having a user defined fraction of zeros (between 0 and 1, where 1 indicate that only genes without any 0s are retained, and 0 indicates that genes with at least a single value different from zero are retained), and plots the frequency distribution of gene counts in the dataset.

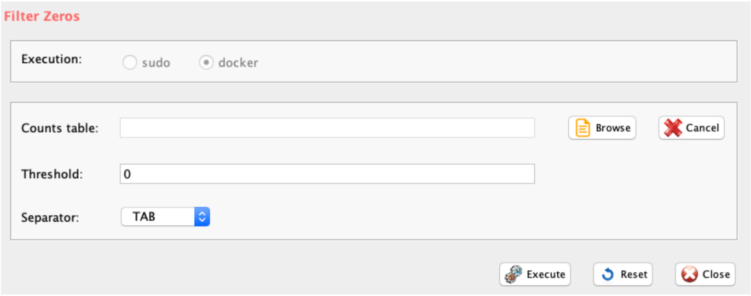

Figure 9: GUI: Filter Zeros panel

- *Parameters* (only those without default; for the full list of parameters please refer to the function help) (Figure 9):
  - *file (GUI Counts table)*, a character string indicating the path of the tab delimited file of cells un-normalized expression counts
  - *threshold (GUI Threshold)*, a number from 0 to 1 indicating the fraction of max accepted zeros in each gene. 0 is set as default and it eliminates only genes having no expression in any cell.
  - *sep (GUI Separator)*, separator used in count file, e.g. '\t', ','

The output is a PDF providing zeros distributions before and after removal of genes with 0s counts. A tab delimited file with the prefix **filtered__** in which the filtered data are saved.

IMPORTANT: In case user would like to apply cell quality filter, e.g. **lorenzFilter**, it is convenient to remove only genes with 0 counts in all cells, i.e. threshold=0 (Figure 10).

```
# Subset of a mouse single cell experiment made to quickly test Lorenz filter.
system("wget http://130.192.119.59/public/testSCumi_mm10.csv.zip")
unzip("testSCumi_mm10.csv.zip")
tmp <- read.table("testSCumi_mm10.csv", sep=",", header=T, row.names=1)
dim(tmp)
#27998   806
write.table(tmp, "testSCumi_mm10.txt", sep="\t", col.names=NA)
filterZeros(file=paste(getwd(),"testSCumi_mm10.txt",sep="/"), threshold=0, sep="\t")
#Out of 27998 genes 11255 are left after removing genes with no counts
#output is filtered_testSCumi_mm10.txt
```

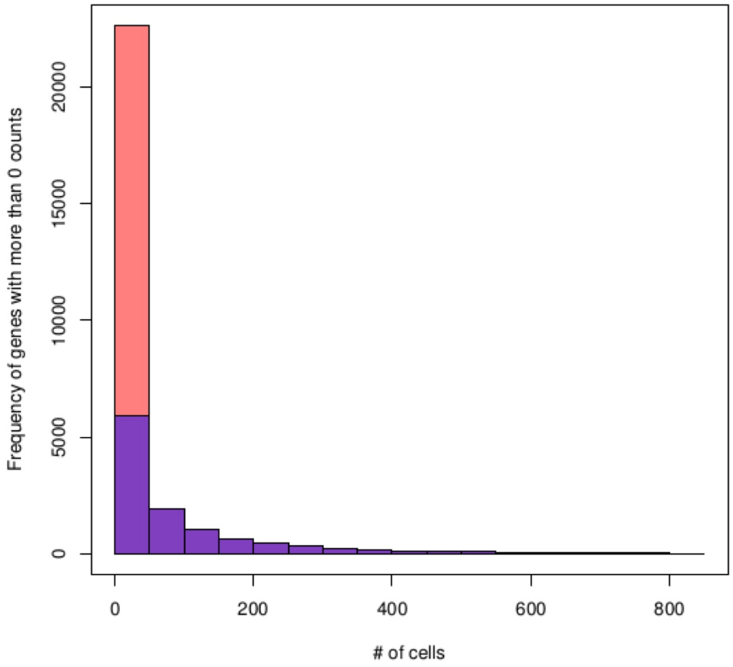

Figure 10: Zeros distribution in full table, orange, and filtered table, blue

Section 3.2 Plotting genes numbers versus total UMIs/reads in each cell

To estimate the overall number of genes detectable in each cell, the function **genesUmi** generates a plot of the number of genes present in a cell with respect to the total number of UMI in the same cell. The number of UMIs required to call a gene present in a cell is a parameter defined by the user, the suggested value is 3 UMIs. The function **mitoRiboUmi** plots **genesUmi** output and also generate the percentage of mitochondrial protein genes with respect to percentage of ribosomal protein genes for each cell.

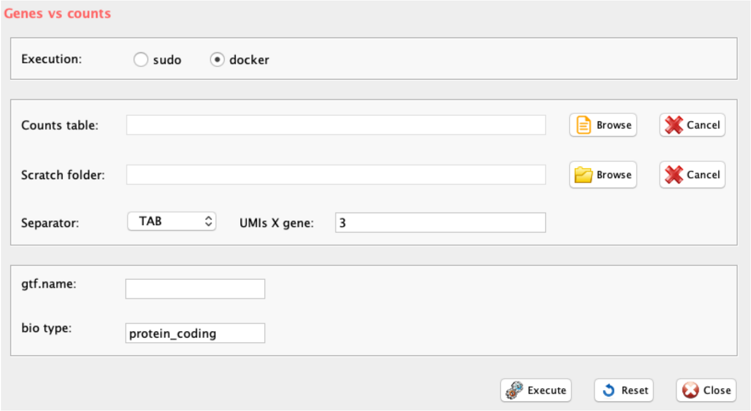

Figure 11: GUI: Genes versus counts panel

- *Parameters genesUmi function* (only those without default; for the full list of parameters please refer to the function help):
  - *file (GUI Counts table)*, a character string indicating the path of the file tab delimited of cells un-normalized expression counts.
  - *umiXgene (UMIs X genes)*, an integer defining how many UMIs are required to call a gene present. default: 3
  - *sep*, separator used in count file, e.g. '\t', ','
- *Parameters mitoRiboUmi* (only those without default; for the full list of parameters please refer to the function help) (Figure 11):
  - *file (GUI Counts table)*, a character string indicating the path of the file tab delimited of cells un-normalized expression counts.
  - *scratch.folder*, a character string indicating the path of the scratch folder
  - *umiXgene (UMIs X genes)*, an integer defining how many UMIs are required to call a gene present. default: 3
  - *separator*, separator used in count file, e.g. '\t', ','
  - *gtf.name*, name for the gtf file to be used
  - *bio.type*, ENSEMBL biotype of interest, default "protein_coding"

The output are two pdfs named respectively **genes.umi.pdf** (Figure 12A), where each dot represents a cell. X axis is the total number of UMIs/reads mapped on each cell in log10 format and Y axis is the number of detected genes and **Ribo_mito.pdf** (Figure 12B), Percentage of mitochondrial protein genes plotted with

respect to percentage of ribosomal protein genes. Cells are colored on the basis of total number detected genes. The latter plot is useful to identify stressed cells, i.e. those with high percentage of mitochondrial genes (Figure 12B, black cells), and low informative cells, i.e. those in which genes are few and mainly ribosomal/mitochondrial (Figure 12B, black and green cells).

```
#N.B. geneUmi and mitoRiboUmi expect as input a raw counts table having as rownames ENSEMBL IDs

library(rCASC)
system("wget ftp://ftp.ensembl.org/pub/release-94/gtf/mus_musculus/Mus_musculus.GRCm38.94.gtf.gz")
system("gzip -d Mus_musculus.GRCm38.94.gtf.gz")
system("wget http://130.192.119.59/public/testSCumi_mm10.csv.zip")
unzip("testSCumi_mm10.csv.zip")
mitoRiboUmi(group="docker", file=paste(getwd(), "testSCumi_mm10.csv", sep="/"),
            scratch.folder="/data/scratch", separator=",", umiXgene=3,
            gtf.name="Mus_musculus.GRCm38.94.gtf", bio.type="protein_coding")

genesUmi(file=paste(getwd(),"testSCumi_mm10.csv",sep="/"), umiXgene=3, sep=",")
```

In Figure 12 it is shown the distribution of genes in cells for 'filtered_testSCumi_mm10.txt' counts table.

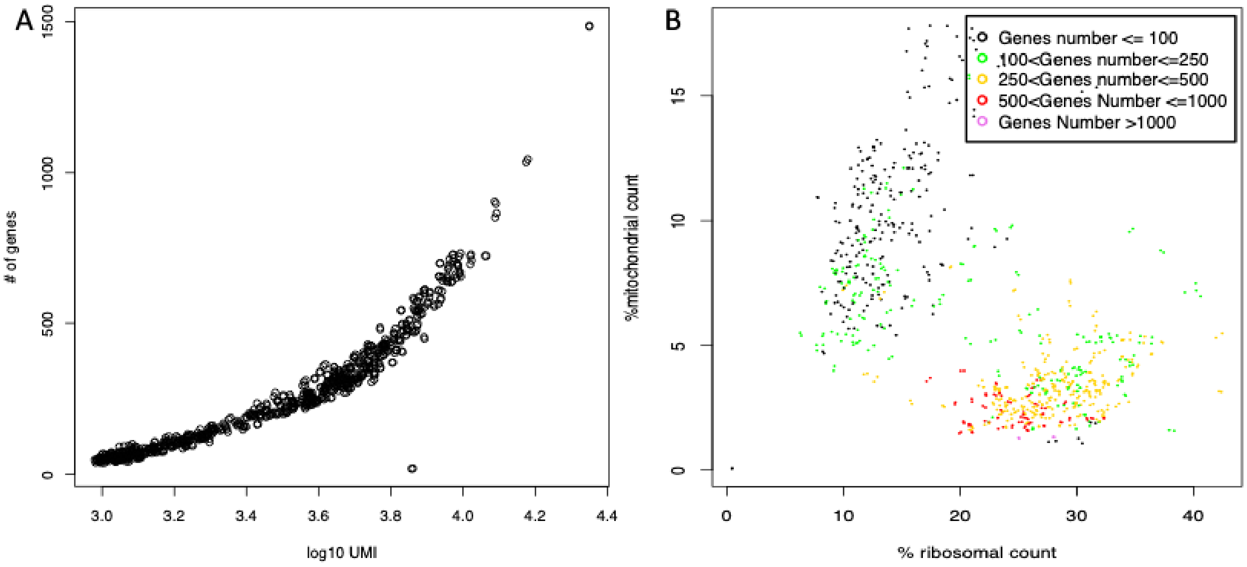

Figure 12: mitoRiboUmi output: A) Number of detected genes plotted for each cell with respect to the total number of UMI/reads in that cell. B) Percentage of mitochondrial protein genes plotted with respect to percentage of ribosomal protein genes. Cells are colored on the basis of total number detected genes

Section 3.2.1 Further considerations about the number of reads/UMIs to be used in a single-cell sequencing experiment.

Ziegenhain et al. published a comparison between single cell sequencing protocols and they show that, in a simulated experiment, at least 250K reads/cell are required for the detection of at least 80% of differentially expressed genes between two groups (Figure 13). Ziegenhain observation clearly also apply to sub-populations clustering.

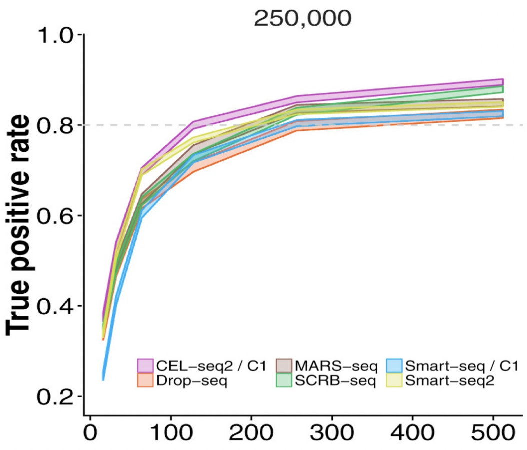

Figure 13: Modified from Figure 6 in Ziegenhain et al. (Mol. Cell 2017). Power of scRNA-Seq methods. For more information on the experiment please see Ziegenhain paper.

Sequencing depth, which affects differential expression analysis and sub-population partitioning, influences the structure of a single-cell dataset at two levels:

- number of genes called present in the experiment,
- robustness of gene expression, i.e. number of reads associated to a gene.

In particular, the number of genes called present in the experiment is the key element for the discrimination between sub-populations. In Figure 14 it is shown the effect of sequencing depth on the number of detectable genes in a set of 10XGenomics sequencing experiments (25K sequenced reads/cell extracted from CD19 B-cells [*Zheng et al 2016*], 83K sequenced reads/cell extracted from naive CD8+ T-cells [*GSM2833284*], and 250K sequenced reads/cell from an unpublished brain mouse experiment) and in an unpublished whole transcript human MAIT-cells single-cell sequencing done on Fluidigm C1 (25K, 100K, and 250K sequenced reads/cell were subsampled from the original fastqs). 3 UMIs are used as minimal threshold to call present a gene in 10XGenomics experiments and 5 reads [*Tarazona et al. 2011*] as minimal threshold to call a gene present in single cell whole transcriptome experiments.

```
#Raw counts for 288 cells randomly extracted from the Zheng data set downloaded from 10XGenomics.
system("wget http://130.192.119.59/public/Zheng_cd19_288cells.txt.zip")
unzip("Zheng_cd19_288cells.txt.zip")
library(rCASC)
genesUmi(file=paste(getwd(),"testSCumi_mm10.csv",sep="/"), umiXgene=3, sep=",")
```

```r
system("mv genes.umi.pdf genes.umi_25k.pdf")
#raw counts for 288 cells randomly extracted from the full GSM2833284 dataset.
#Raw counts table was generated with cellranger 2.0 starting from the h5 file deposited on GEO.
system("wget http://130.192.119.59/public/GSM2833284_Naive_WT_Rep1_288cell.txt.zip")
unzip("GSM2833284_Naive_WT_Rep1_288cell.txt.zip")
library(rCASC)
genesUmi(file=paste(getwd(),"GSM2833284_Naive_WT_Rep1_288cell.txt",sep="/"), umiXgene=3, sep="\t")
system("mv genes.umi.pdf genes.umi_86k.pdf")
#raw counts from 288 cells randomly extracted from an unpublished brain dataset.
#Raw counts table was generated with cellranger 2.0
system("wget http://130.192.119.59/public/brain_unpublished_288cells.txt.zip")
unzip("brain_unpublished_288cells.txt.zip")
library(rCASC)
genesUmi(file=paste(getwd(),"brain_unpublished_288cells.txt",sep="/"), umiXgene=3, sep="\t")
system("mv genes.umi.pdf genes.umi_250k.pdf")

#Smart-seq experiment: Unpublished human MAIT cells.
#Counts table was generated using the rnaseqCounts function implemented in docker4seq package
#(https://github.com/kendomaniac/docker4seq)
system("wget http://130.192.119.59/public/c1_experiment.zip")
unzip("c1_experiment.zip")
setwd("c1_experiment")
library(rCASC)
genesUmi(file=paste(getwd(),"250K_counts.txt",sep="/"), umiXgene=5, sep="\t")
system("mv genes.umi.pdf genes.umi_250K.pdf")
genesUmi(file=paste(getwd(),"100K_counts.txt",sep="/"), umiXgene=5, sep="\t")
system("mv genes.umi.pdf genes.umi_100K.pdf")
genesUmi(file=paste(getwd(),"25K_counts.txt",sep="/"), umiXgene=5, sep="\t")
system("mv genes.umi.pdf genes.umi_25K.pdf")
```

Figure 14 clearly shows that the number of genes detectable by 10XGenomics sequencing (Figure 14A-C) is far less of those detectable using a whole transcript experiment (Figure 14D-F). In the case of 25K reads/cells in 3' end sequencing (Figure 14A) the number of called genes goes from a dozen of genes to 350. In a whole transcript sequencing experiment where 25K reads/cells were considered (Figure 14D), 350 genes is the lowest number of genes detectable in a cell. In 10XGenomics experiment with 250K reads/cell the range of detectable genes goes from few hundred to 2000, which is relatively similar to the number of detectable genes in a whole transcripts experiment where the same number of reads/cells were considered. The larger scattering and the overall lower number of detectable genes in 3' end sequencing experiment, with respect to whole transcript experiments, is a peculiarity of the technology [*Ziegenhain et al. 2017*].
It has also to be highlighted that cells with a very low number of genes called present will have a genes repertoire made mainly of housekeeping genes, ribosomal and mitochondrial genes, see Figure 19 in **Section 3.4**. Thus, the lack of cell-type specific genes makes these cells useless for the identification of functional cell sub-populations.

However, it has to be underlined that each cell type is characterized by a peculiar transcriptional rate and

therefore, the technical assessment of the reads per cell vs. genes detected between different cell types, i.e. 14A-C, might be bias by differences in the transcriptional rate of the different cells used in this specific example. Instead, the above-mentioned bias does not affect 14D-F because they are generated by a down sampling of a set of cells sequenced with the smart-seq protocol at a coverage of 1 million reads/cell.

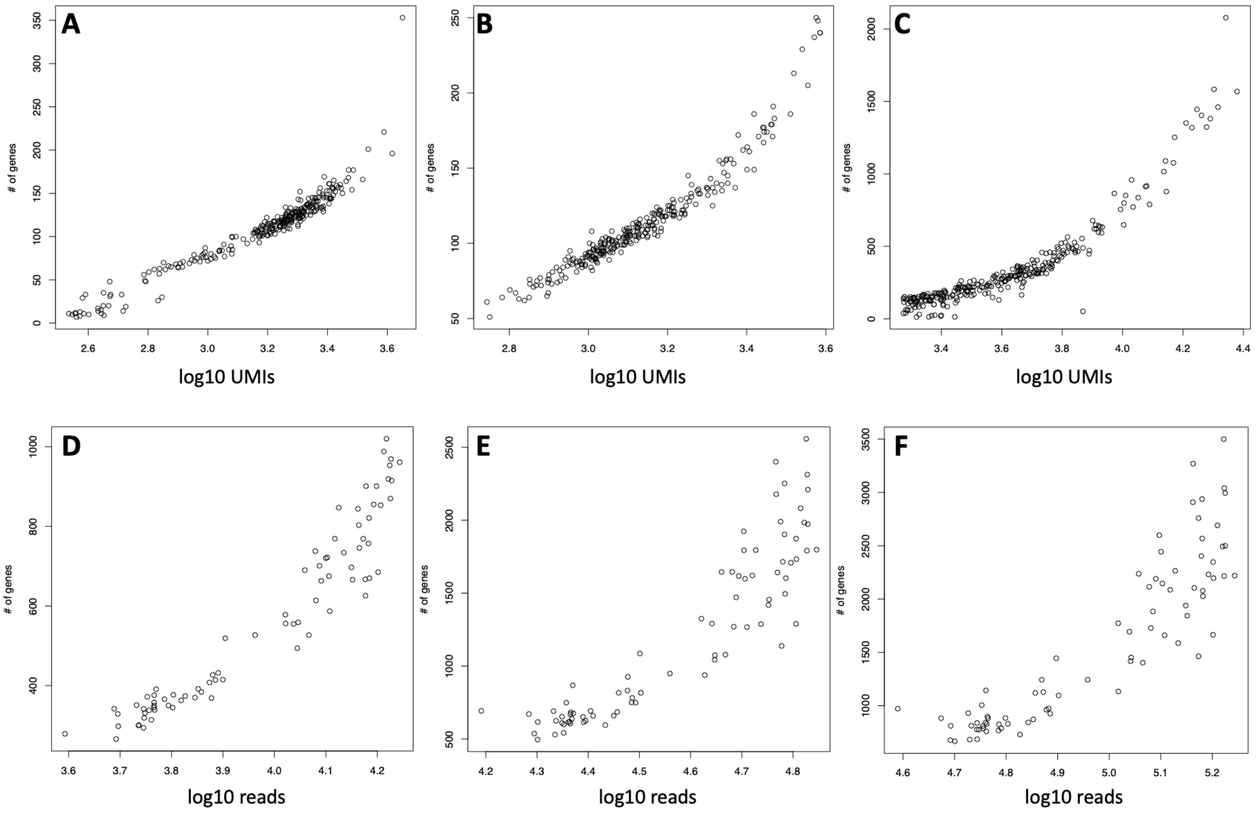

Figure 14: Number of detected genes with respect to mapped reads. A) 25K reads/cell 10XGenomics platform, 3' end sequencing v2 chemistry. B) 83K reads/cell 10XGenomics platform, 3' end sequencing v2 chemistry. C) 250K reads/cell 10XGenomics platform, 3' end sequencing v2 chemistry. D) 25K reads/cell C1 platform, whole transcript sequencing, E) 100K reads/cell C1 platform, whole transcript sequencing, F) 250K reads/cell C1 platform, whole transcript sequencing.

The above observations indicate that 3' end sequencing has a much lower genes called present with respect to whole transcript sequencing.

We have further investigated the following point:

- Is the number of total UMIs/cell affecting the separation between sub-populations?

To address the above point, we used two types of cells belonging to the T-cells [*Zheng* 2016]. The two sets of cells were sequenced with a coverage of approximately 21K reads/cell:

- T-cytotoxic (10209 cells, Figure 15A) cells,
- T-regulatory (10263, Figure 15B) cells.

We generated three datasets:

- **d3.4**, which is made of 100 cells randomly selected within cells having, in each of the two datasets, a total UMIs/cell value greater than 2511 in each cell.
- **d3**, which is made of 100 cells randomly selected within cells having, in each of the two datasets, a total UMIs/cell value comprised between 2511 and 1000 in each cell.
- **d3m**, which is made of 100 cells randomly selected within cells having, in each of the two datasets, a total UMIs/cell smaller than 1000 in each cell.

The separation between T-cytotoxic and T-regulatory achievable with PCA is shown in Figure 15C-E.

```
system("wget http://130.192.119.59/public/counts_effect.zip")
unzip("counts_effect.zip")
setwd("counts_effect")
#dots are only accepted in the file type separator!
system("mv df3.4.txt df3_4.txt")
topx(group="docker", file=paste(getwd(),"df3_4.txt", sep="/"), threshold=1000, logged=FALSE, type="expression", separator = "\t"
library(docker4seq)

#N.B. if the type parameter of pca function is set to "counts",
#raw counts are converted in log10 counts before executing PCA analysis.

pca(experiment.table="filitered_expression_df3_4.txt", type="counts",
    legend.position="topleft", covariatesInNames=TRUE, samplesName=FALSE,
    principal.components=c(1,2), pdf = TRUE,
    output.folder=getwd())

topx(group="docker", file=paste(getwd(),"df3.txt", sep="/"),threshold=1000, logged=FALSE, type="expression", separator = "\t")

pca(experiment.table="filitered_expression_df3.txt", type="counts",
    legend.position="topleft", covariatesInNames=TRUE, samplesName=FALSE,
    principal.components=c(1,2), pdf = TRUE,
    output.folder=getwd())

topx(group="docker", file=paste(getwd(),"df3m.txt", sep="/"),threshold=1000, logged=FALSE, type="expression", separator = "\t")

pca(experiment.table="filitered_expression_df3m.txt", type="counts",
    legend.position="topleft", covariatesInNames=TRUE, samplesName=FALSE,
    principal.components=c(1,2), pdf = TRUE,
    output.folder=getwd())
```

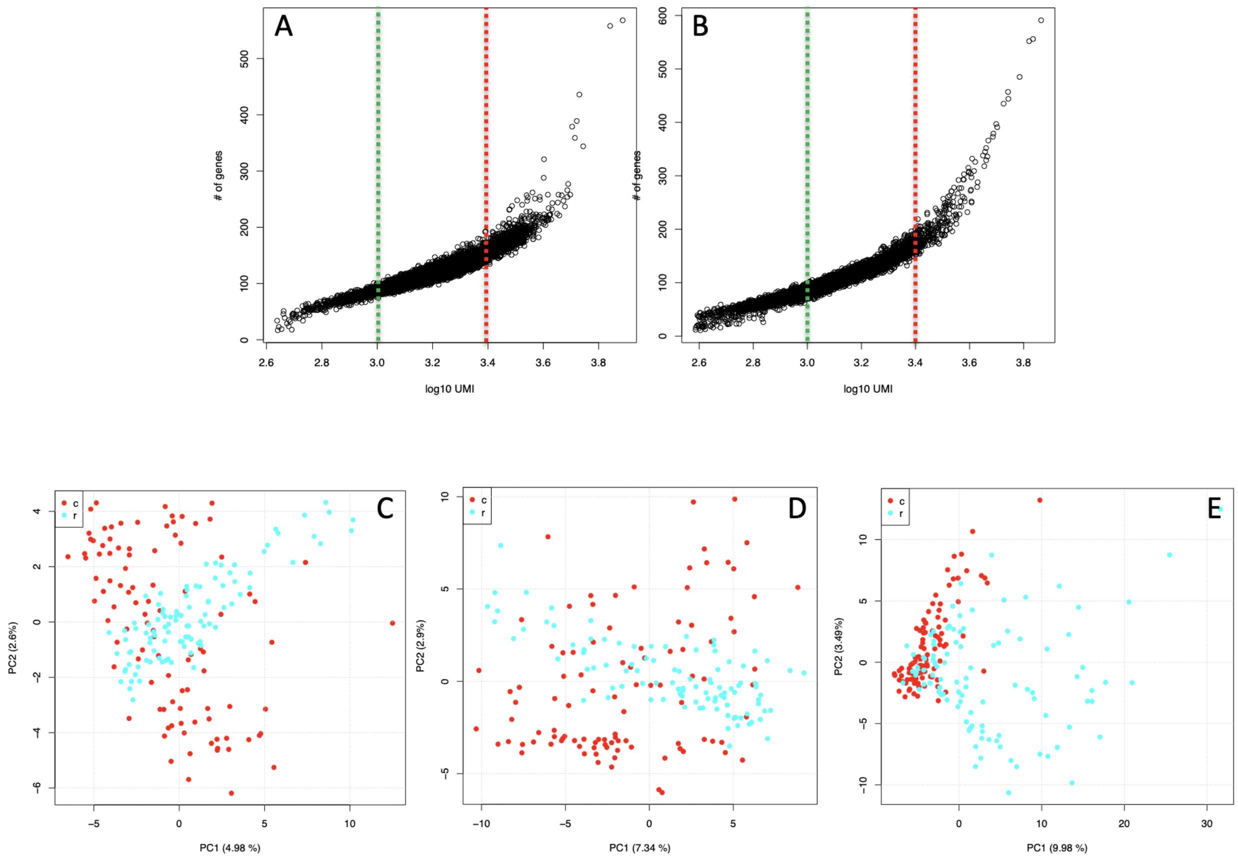

Figure 15: Detectable genes in a Zheng data subset. A) T-cytotoxic genes versus total cell reads plot. B) T-regulatory genes versus total cell reads plot. C) d3m set, made of cells with less than 1000 counts each, D) df3, made of cells with counts between 1000 and 2511. E) d3.4, made of cells with more than 2511 counts each. T-cytotoxic red dots, T-regulatory light blue dots

It is notable that only the dataset including cells with more than 2511 UMIs/cell (Figure 15E) is the one where the separation between the two T-cell types improves. In the d3 and d3m (Figure 15C,D) the amount of variance explained by the first component is much lower of that observable in d3.4 and the datasets are intersperse.

Thus, since 3' end sequencing platforms (10Xgenomics, inDrop) has the advantage to produce high number of sequenced cells, users might decide to select for clustering only the subset of cells with the highest number of genes called present.

Section 3.3 Identifying and removing cell low-quality outliers

Lorenz statistics was implemented in rCASC **lorenzFilter** function. This function derives from the work of Diaz and coworkers [*2016*] detecting low quality cells and this statistics correlates with live-dead staining [*Diaz et al. 2016*].

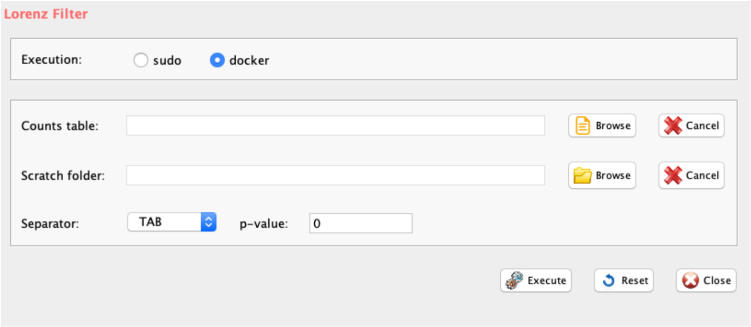

Figure 16: GUI: Lorenz Filter panel

- *Parameters* (only those without default; for the full list of parameters please refer to the function help) (Figure 16):
  - *scratch.folder (GUI Scratch folder)*, the path of the scratch folder
  - *file (GUI Counts table)*, full path to the count file MUST be provided
  - *p_value (GUI p-value)*, lorenz statistics threshold, suggested value 0.05 (i.e. 5% probability that a cell of low quality is selected)
  - *separator (GUI Separator)*, separator used in count file, e.g. '\t', ','

The output is a counts table without low quality cells and with the prefix **lorenz_filtered_**. Output will be in the same format and with the same separator of input.

```
#example data set provided as part of rCASC package
system("wget http://130.192.119.59/public/testSCumi_mm10.csv.zip")
unzip("testSCumi_mm10.csv.zip")
#IMPORTANT: full path to the file MUST be cell count file included!

#N.B. The input file for lorenzFilter are raw counts

library(rCASC)
# the p_value indicate the probability that a low quality cell is retained in the
# dataset filtered on the basis of Lorenz Statistics.
lorenzFilter(group="docker",scratch.folder="/data/scratch/",
             file=paste(getwd(),"testSCumi_mm10.csv", sep="/"),
             p_value=0.05, separator=',')

tmp0 <- read.table("testSCumi_mm10.csv", sep=",", header=T, row.names=1)
dim(tmp0)
#806 cells

tmp <- read.table("lorenz_testSCumi_mm10.csv", sep=",", header=T, row.names=1)
dim(tmp)
#782 cells
```

In the example above 24 cells were removed because of their low quality (Figure 17).

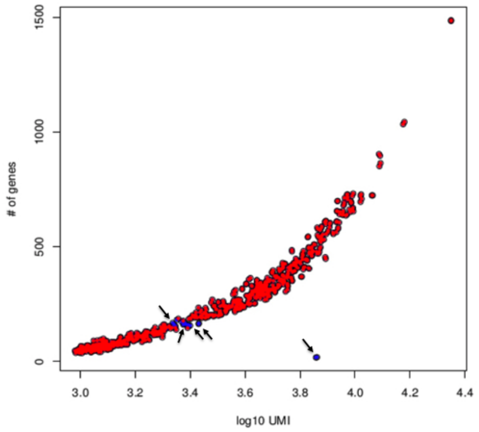

Figure 17: Lorenz filtering: cells retained after filtering are labelled in red, instead cells discarded because of their low quality are labelled in blue.

Section 3.4 Annotation and mitochondrial/ribosomal protein genes removal

The function **scannobyGtf** allows the annotation of single-cell matrix, if ENSEMBL gene ids are provided. The function requires the ENSEMBL GTF of the organism under analysis and allows the selection of specific annotation biotypes, e.g. protein_coding.

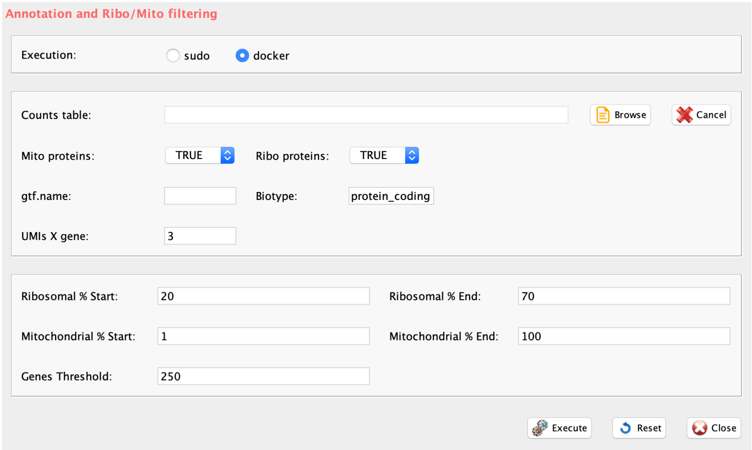

Figure 18: GUI: Annotation panel

- *Parameters* (only those without default; for the full list of parameters please refer to the function help) (Figure 18):
  - *file (GUI Counts table)*, full path to the count file MUST be provided
  - *gtf.name*, ENSEMBL gtf file name. GTF is located in the same folder where counts file is.
  - *biotype*, biotype of interest. See *www.ensembl.org/info/genome/genebuild/biotypes.html* for more information
  - *mt (GUI Mt)*, a boolean to define if mitochondrial genes have to be removed, FALSE mean that mt genes are removed

- *ribo.proteins (GUI Ribo Proteins)*, a boolean to define if ribosomal proteins have to be removed, FALSE mean that ribosomal proteins (gene names starting with rpl or rps) are removed
- *umiXgene (GUI UMIs X gene)*, a integer defining how many UMIs are required to call a gene as present. default: 3
- *riboStart.percentage*, start range for ribosomal percentage, cells within the range are kept
- *riboEnd.percentage*, end range for ribosomal percentage, cells within the range are kept
- *mitoStart.percentage*, start range for mitochondrial percentage, cells within the range are retained
- *mitoEnd.percentage*, end range for mitochondrial percentage, cells within the range are retained
- *thresholdGenes*, parameter to filter cells according to the number og significative genes expressed

```
#running annotation and removal of mito and ribo proteins genes
system("wget ftp://ftp.ensembl.org/pub/release-92/gtf/mus_musculus/Mus_musculus.GRCm38.94.gtf.gz")
system("gunzip Mus_musculus.GRCm38.94.gtf.gz")
scannobyGtf(group="docker", file=paste(getwd(),"testSCumi_mm10.csv",sep="/"),
                    gtf.name="Mus_musculus.GRCm38.94.gtf", biotype="protein_coding",
                    mt=TRUE, ribo.proteins=TRUE, umiXgene=3, riboStart.percentage=0,
                    riboEnd.percentage=100, mitoStart.percentage=0, mitoEnd.percentage=100, thresholdGenes=100)
```

The output are a file with prefix **annotated__**, containing all annotated genes and a file with prefix **filtered__annotated__** which contain the filtered subset of cells. Furthermore, the **filteredStatistics.txt** indicates how many cells and genes were removed.

Ribosomal RNA and ribosomal proteins represent a significant part of cell cargo. Large cells and actively proliferating cells will have respectively more ribosomes and more active ribosome synthesis [*Montanaro et al. 2008*]. Thus, ribosomal proteins expression might represent one of the major confounding factor in cluster formation between active and quiescent cells. Furthermore, the main function of mitochondria is to produce energy through aerobic respiration. The number of cell mitochondria depends on its metabolic demands [*Nasrallah and Horvath 2014*]. This might also affect clustering, favoring the separation between metabolic active and resting cells, with respect to functional differences between sub-populations. *scannobyGtf* allows also the removal of mitochondrial and ribosomal protein genes.

```
library(rCASC)
scannobyGtf(group="docker", file=paste(getwd(),"testSCumi_mm10.txt",sep="/"),
                    gtf.name="Mus_musculus.GRCm38.94.gtf", biotype="protein_coding",
                    mt=FALSE, ribo.proteins=FALSE,umiXgene=3,
                    riboStart.percentage=10, riboEnd.percentage=70, mitoStart.percentage=0,
                    mitoEnd.percentage=5, thresholdGenes=100)
```

In figure 19B is shown the effect of the removal of both mitochondrial and ribosomal protein genes and the removal of cells characterized by high percentage of mitochondrial protein genes (>5%) and too litte (<10%) or too much (>70%) ribosomal protein genes .

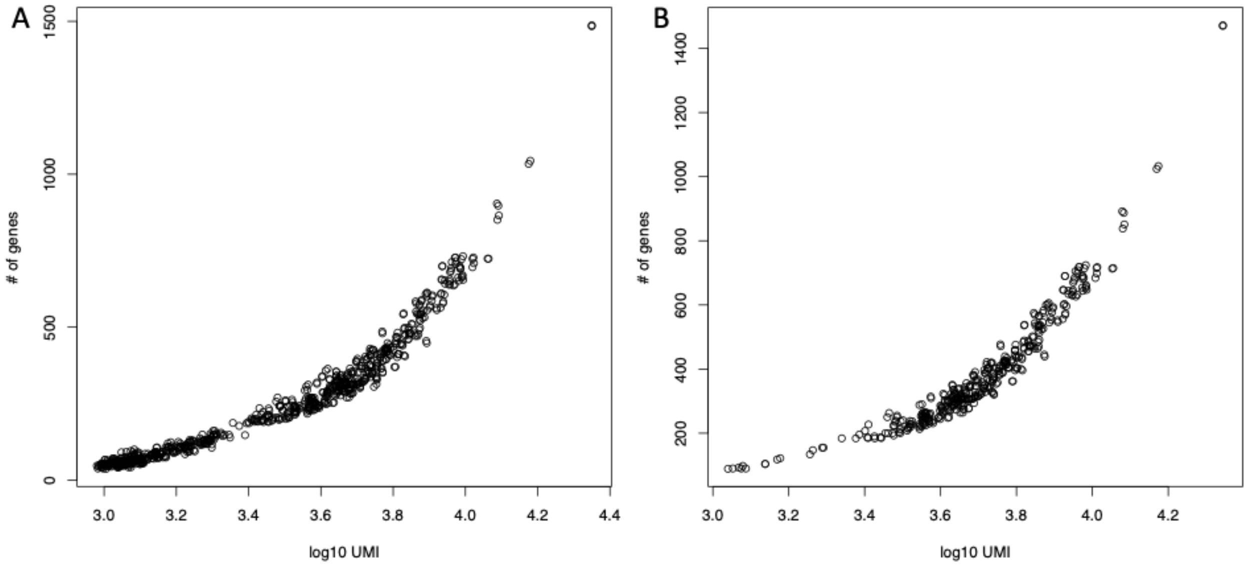

Figure 19: Removing mitochondrial and ribosomal proteins genes. A) All annotated cells. B) Only cells passing the filters

Section 3.5 Top expressed genes

For clustering purposes user might decide to use the top expressed/variable genes. The function **topx** provides two options:

- the selection of the X top expressed genes given a user defined threshold, parameter type="expression"
- the selection of the X top variable genes given a user defined threshold, parameter type="variance"
  - gene variance is calculated using edgeR Tag-wise dispersion. The method estimates the gene-wise dispersion implementing a conditional maximum likelihood procedure. For more information please refer to edgeR Bioconductor package manual.

The function also produces a pdf file gene_expression_distribution.pdf showing the changes in the UMIs/gene expression distribution upon **topx** filtering.

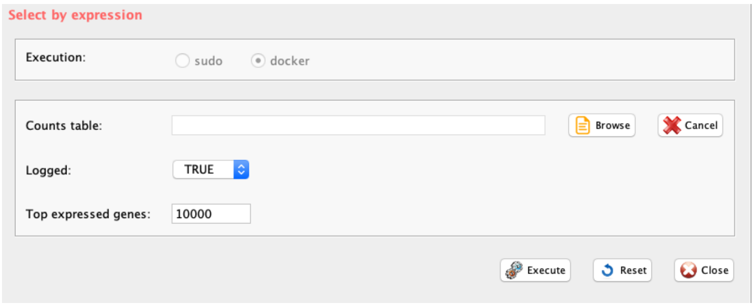

Figure 20: GUI: Top X expressed genes panel

- *Parameters* (only those without default; for the full list of parameters please refer to the function help)

(Figure 20):

- *file (GUI Counts table)*, full path to the count file MUST be provided
- *threshold (GUI Top expressed genes)*, number of top expressed genes to be selected. Default 0, i.e. only genes will all cell values equal to 0 will be removed
- *logged (GUI Logged)*, boolean, if FALSE gene expression data are log10 transformed before being plotted.
- *type*, expression refers to the selection of the top expressed genes, variance to the the selection of the top variable genes
- *separator*, separator used in count file, e.g. '\t', ','

```
library(rCASC)
genesUmi(file=paste(getwd(), counts.matrix="testSCumi_mm10.csv", sep="/"), umiXgene=3, sep="\t")
topx(group="docker", file=paste(getwd(),file.name="testSCumi_mm10.csv", sep="/"),threshold=10000, logged=FALSE, type="expression
genesUmi(file=paste(getwd(), counts.matrix="testSCumi_mm10_10000.txt", sep="/"), umiXgene=3,  sep="\t")
```

IMPORTANT: The core clustering tool in rCASC is SIMLR, **Section 5**. SIMLR requires that the number of genes must be larger than the number of analyzed cells.

Section 3.6 Data normalization

The best way to normalize single-cell RNA-seq data has not yet been resolved, especially in the case of UMI data. We inserted in our workflow two possible options:

- *SCnorm*, which works best with whole transcript data.
- *scone*, which provides different global scaling methods that can be applied to UMI single-cell data.

Section 3.6.1 SCnorm

SCnorm performs a quantile-regression based approach for robust normalization of single-cell RNA-seq data. SCnorm groups genes based on their count-depth relationship then applies a quantile regression to each group in order to estimate scaling factors which will remove the effect of sequencing depth from the counts.

IMPORTANT: SCnorm is not intended for datasets with more than ~80% zero counts, because of lack of algorithm convergence in these situations.

Section 3.6.1.1 Check counts-depth relationship

Before normalizing using **scnorm**, it is advised to check the data count-depth relationship. If all genes have a similar relationship then a global normalization strategy such as median-by-ratio in the DESeq package or TMM in edgeR will also be adequate. However, when the count-depth relationship varies among genes global scaling strategies leads to poor normalization. In these cases, the normalization provided by SCnorm is recommended.

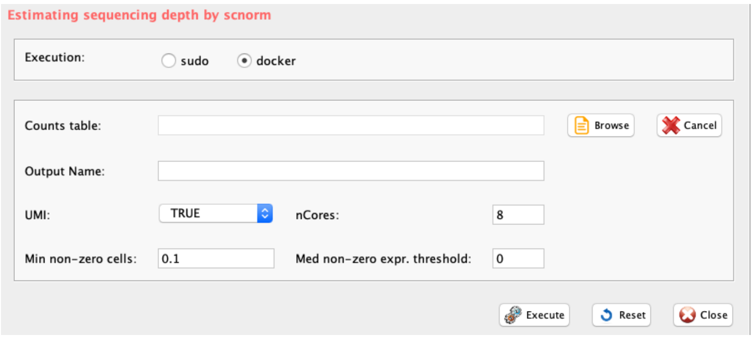

Figure 21: GUI: SCnorm: check counts-depth relationship panel

checkCountDepth provides a wrapper, in rCASC, for the checkCountDepth of the *SCnorm package*, which estimates the count-depth relationship for all genes.

- *Parameters* (only those without default; for the full list of parameters please refer to the function help) (Figure 21):
  - *file (GUI Counts table)*, full path to the file MUST be included. Only tab delimited files are supported
  - *conditions*, vector of condition labels, this should correspond to the columns of the un-normalized expression matrix. If not provided data is assumed to come from same condition/batch.
  - *ditherCounts (GUI UMI)*, boolean. Setting to TRUE might improve results with UMI data.
  - *FilterCellProportion (GUI Min non-zero cells)*, a value indicating the proportion of non-zero expression estimates required to include the genes into the evaluation. Default is .10, and will not go below a proportion which uses less than 10 total cells/samples
  - *FilterExpression (GUI Med non-zero expr. threshold)*, a value indicating exclude genes having median of non-zero expression below this threshold from count-depth plots #' @param ditherCounts, Setting to TRUE might improve results with UMI data, default is FALSE #' @param outputName, specify the path and/or name of output file.
  - *outputName*, name of output file.
  - *nCores*, number of cores to use.

```
#N.B. checkCountDepth function requires as input raw counts table

#this specific example is an UMI counts table made of 12 cells having at least 10K UMIs/cell.
system("wget http://130.192.119.59/public/example_UMI.txt.zip")
unzip("example_UMI.txt.zip")
conditions=rep(1,12)
checkCountDepth(group="docker", file=paste(getwd(), "example_UMI.txt", sep="/"),
    conditions=conditions, FilterCellProportion=0.1, FilterExpression=0,
    ditherCounts=TRUE, outputName="example_UMI", nCores=8)
```

The output is a PDF (Figure 22), providing a view of the counts distribution, and a file selected.genes.txt, which contains the genes selected to run the analysis.

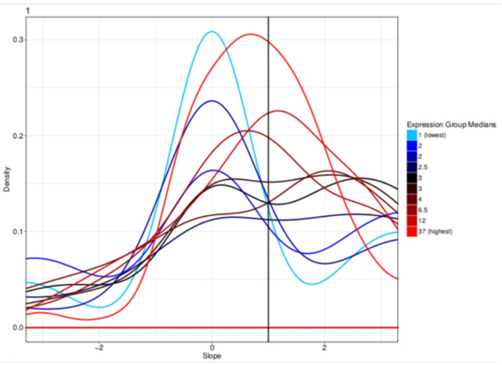

Figure 22: checkCountDepth output plot provides an evaluation of count-depth relationship in un-normalized data. The effects of the normalization procedure is shown in the following figure.

Section 3.6.1.2 scnorm

The **scnorm** function executes SCnorm from *SCnorm package*, which normalizes across cells to remove the effect of sequencing depth on the counts and returns the normalized expression count.

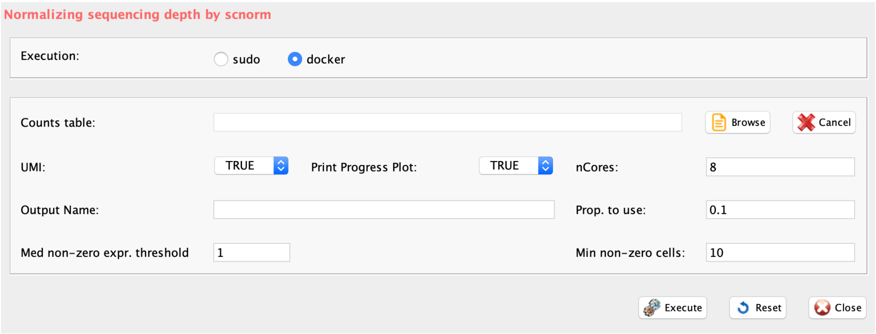

Figure 23: GUI: SCnorm: counts-depth normalization panel

- *Parameters* (only those without default; for the full list of parameters please refer to the function help) (Figure 23):
  - *file (GUI Counts table)*, full path to the file MUST be included. Only tab delimited files are supported
  - *conditions*, vector of condition labels, this should correspond to the columns of the un-normalized expression matrix.
  - *outputName*, specify the name of output file.
  - *nCores*, number of cores to use.
  - *filtercellNum (Min non-zero cells)*, the number of non-zero expression estimate required to include the genes into the SCnorm fitting (default = 10). The initial grouping fits a quantile regression to each gene, making this value too low gives unstable fits.
  - *ditherCount (GUI UMI)*, boolean. Setting to TRUE might improve results with UMI data.
  - *FilterExpression (GUI Med non-zero expr. threshold)*, a value indicating exclude genes having median of non-zero expression below this threshold from count-depth plots

  – *PropToUse (GUI Prop. to use)*, as default is set to 0.25, but to increase speed with large data set could be reduced, e.g. 0.1
  – *PrintProgressPlots*, boolean. If it is set to TRUE produces a plot as SCnorm determines the optimal number of groups

```
#N.B. scnorm function requires as input raw counts table

system("wget http://130.192.119.59/public/example_UMI.txt.zip")
unzip("example_UMI.txt.zip")
#this specific example is an UMI counts table made of 12 cells having at least 10K UMIs/cell.
conditions=rep(1,12)
scnorm(group="docker", file=paste(getwd(), "example_UMI.txt", sep="/"),
     conditions=conditions,outputName="example_UMI", nCores=8, filtercellNum=10,
     ditherCount=TRUE, PropToUse=0.1, PrintProgressPlots=TRUE, FilterExpression=1)
```

The output files are plots of the normalization effects (Figure 24), a tab delimited file containing the normalized data, with the prefix **normalized_**, and **discarded_genes.txt**, which contains the discarded genes.

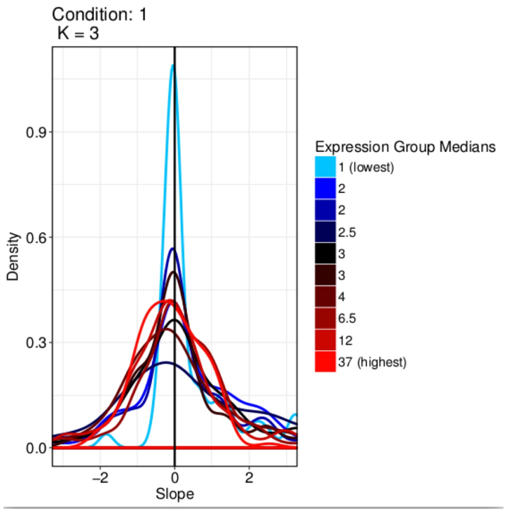

Figure 24: Effect of the SCnorm on the dataset in the previous figure

scnorm is compliant with *SIMLR*, the rCASC core clustering tool.

Section 3.6.2 scone

scone package embeds:

- Centered log-ratio (**CLR**) normalization
- Relative log-expression (**RLE**; DESeq) scaling normalization
  – the scaling factors are calculated for each lane as median of the ratio, for each gene, of its read count of its geometric mean across all lanes.
- Full-quantile normalization

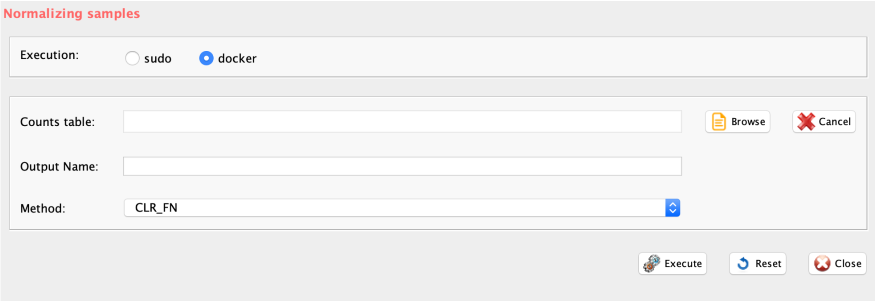

Figure 25: GUI: Normalization panel

  - quantile normalization is a technique for making two or more distributions identical in statistical properties. To quantile normalize two or more samples to each other, sort the samples, then set to the average (usually, arithmetic mean) of the samples. So the highest value in all cases becomes the mean of the highest values, the second highest value becomes the mean of the second highest values, and so on.

- Simple deconvolution normalization
- Sum scaling normalization
  - Gene counts are divided by the total number of mapped reads (or library size) associated with their lane and multiplied by the mean total count across all the samples of the dataset.
- Weighted trimmed mean of M-values (**TMM**, edgeR) scaling normalization (suitable for single-cell)
  - to compute the TMM factor, one lane is considered a reference sample and the others test samples, with TMM being the weighted mean of log ratios between test and reference, after excluding the most expressed genes and the genes with the largest log ratios.
- Upper-quartile (**UQ**) scaling normalization
  - the total counts are replaced by the upper quartile of counts different from 0 in the computation of the normalization factors.

```
#Weighted trimmed mean of M-values (TMM) scaling normalization
system("wget http://130.192.119.59/public/example_UMI.txt.zip")
unzip("example_UMI.txt.zip")
umiNorm(group="docker", file=paste(getwd(), "example_UMI.txt", sep="/"),
        outputName="example_UMI", normMethod="TMM_FN")
```

IMPORTANT: In case sub-population discovery is the analysis task, it is important to check if a specific normalization is compliant with the clustering approach in use. For example, in the case of *SIMLR*, the rCASC core clustering tool, the normalizations provided in **scone** are not compliant, because they remove some of the features required to run the SIMLR multi-kernel learning analysis. TMM is instead compliant with the rCASC implementation of **tSne**. In case *Seurat* clustering is used the dataset does not required any normalization since a normalization procedure is included in the algorithm.

Section 3.7 Log conversion of a count table

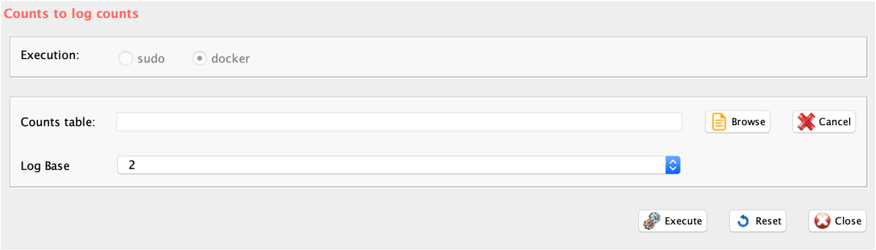

Figure 26: GUI: Log transformation for counts table panel

The function **counts2log** can convert a count table in a log10 values saved in a comma separated or tab delimited file.

- *Parameters* (only those without default; for the full list of parameters please refer to the function help) (Figure 26):
  - *file (GUI Counts table)*, full path to the file MUST be included.
  - *log.base*, the base of the log to be used for the transformation

```
counts2log(file=paste(getwd(), "example_UMI.txt", sep="/"), log.base=10)
```

Section 3.8 Detecting and removing cell cycle bias

Single-cell RNA-Sequencing measurement of expression often suffers from large systematic bias. A major source of this bias is cell cycle, which introduces large within-cell-type heterogeneity that can obscure the differences in expression between cell types. *Barron and Li* developed in 2016 a R package called *ccRemover* which removes cell cycle effects and preserves other biological signals of interest.

However, before applying *ccRemover*, it is essential to address if the removal of cell cycle effect is required. *reCAT* is a modeling framework for unsynchronized single-cell transcriptome data that can reconstruct cell cycle time-series. Thus, reCAT cell cycle prediction step can be used to check if cell cycle effect can be detected in a dataset and therefore ccRemover normalization approach will be needed.

Section 3.8.1 Evaluating the presence of cell cycle effect in a dataset: *reCAT*

reCAT prediction step is implemented in rCASC in the function **recatPrediction**, which requires a data set annotated using **scannobyGtf**.

- *Parameters* (only those without default; for the full list of parameters please refer to the function help) (Figure 27):
  - *scratch.folder*, the path of the scratch folder
  - *file (GUI Counts table)*, the path to the input file
  - *separator*, separator used in count file, e.g. '\t', ','

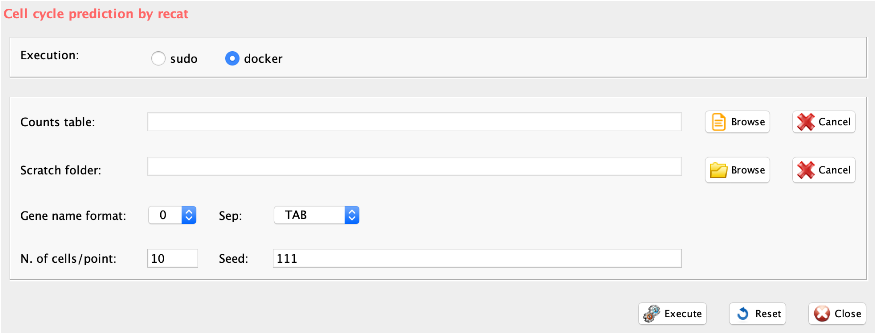

Figure 27: GUI: cell cycle estimation panel

- *geneNameControl (GUI gene name format)*, 0 if the matrix has gene symbol without ENSEMBL code. 1 if the gene names is formatted like this : ENSMUSG00000000001:Gnai3. If the gene names is only ENSEMBL name SCannoByGtf has to be executed before recatPrediction.
- *seed*, important parameter for reproduce the same result with the same input, default 111
- *window (GUI N. cells/point)*, number of cell plotted per point

To show the differences existing between a dataset characterized by cell cycle bias and one that is not, we used two datasets:

- the dataset published by [*Buettner et al. 2015*], containing naive-T-cells and T-helper2-cells mixed together and sorted on the basis of the cell cycle state.
- The quiescent naive T-cells dataset part of the publication of *Pace et al.*, expected to be in G0.

To execute the analysis on the same number of cells, 288 cells were randomly selected from quiescent naive T-cells dataset. In Figure 28A the presence of oscillatory behavior is evident in the predicted cells time series and the G1 and G2M trends are indicated respectively in blue and red dashed curves. On the other hand, the oscillatory behavior is totally absent (Figure 28B) in the naive T-cells, which are expected to be quiescent in G0.

```
#Raw from the Buettner publication
 system("wget http://130.192.119.59/public/buettner_G1G2MS_counts.txt.zip")
 unzip("buettner_G1G2MS_counts.txt.zip")

#annotating the data set to obtain the gene names in the format ensemblID:symbol
scannobyGtf(group="docker", file=paste(getwd(),"buettner_G1G2MS_counts.txt",sep="/"),
                    gtf.name="Mus_musculus.GRCm38.94.gtf", biotype="protein_coding",
                    mt=TRUE, ribo.proteins=TRUE,umiXgene=3, riboStart.percentage=0,
                    riboEnd.percentage=100, mitoStart.percentage=0, mitoEnd.percentage=100, thresholdGenes=100)

#running cell cycle prediction
recatPrediction(group="docker",scratch.folder="/data/scratch",
                file=paste(getwd(), "annotated_buettner_G1G2MS_counts.txt", sep="/"),
                separator="\t", geneNameControl=1, window=10, seed=111)
```

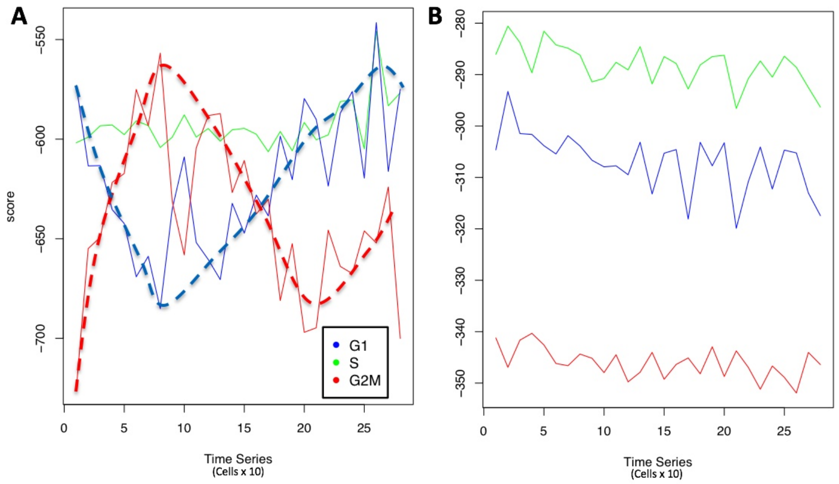

Figure 28: Cell cycle assignment to the cells. A) Buettner et al. (Nat. Biotechnol. 2015) raw dataset, cells are expected to be distributed in G1, S and G2M, B) Naive T-cells, expected to be mainly in G0 (Science 2018).

```
#same analysis as above on 10XGenomix data of quiescent naive-T cells.
#Raw counts table was generated with cellranger 2.0 starting from the h5 files at GEO.
system("wget http://130.192.119.59/public/GSM2833284_Naive_WT_Rep1_288cell.txt.zip")
unzip("GSM2833284_Naive_WT_Rep1_288cell.txt.zip")
scannobyGtf(group="docker", file=paste(getwd(),"GSM2833284_Naive_WT_Rep1_288cell.txt",sep="/"),
                    gtf.name="Mus_musculus.GRCm38.94.gtf", biotype="protein_coding",
                    mt=TRUE, ribo.proteins=TRUE,umiXgene=3, riboStart.percentage=0,
                    riboEnd.percentage=100, mitoStart.percentage=0, mitoEnd.percentage=100, thresholdGenes=100)


#N.B. recatPrediction function requires as input raw counts table previously annotated with scannobyGtf function

recatPrediction(group="docker",scratch.folder="/data/scratch",
                file=paste(getwd(), "annotated_GSM2833284_Naive_WT_Rep1_288cell.txt", sep="/"),
                separator="\t", geneNameControl=1, window=10, seed=111)
```

Section 3.8.2 Removing cell cycle effect in a dataset: *ccRemover*

ccRemover software is implemented in rCASC in the function **ccRemove**, which also requires a data set annotated using **scannobyGtf**.

- *Parameters* (only those without default; for the full list of parameters please refer to the function help) (Figure 29):
  - *scratch.folder*, the path of the scratch folder
  - *file (GUI Counts table)*, the path to the input file, including the counts table name
  - *separator*, separator used in count file, e.g. '\t', ','

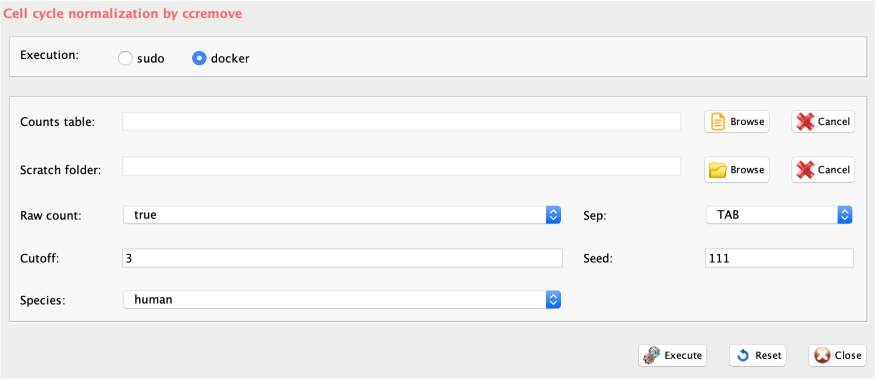

Figure 29: GUI: cell cycle bias removal panel

- *seed*, is important to reproduce the same results with the same input, default=111
- *cutoff*, p-value to use: 3 is almost equal to 0.05
- *species*, human or mouse
- *rawCount*, 1 for unlogged and not-normalized, 0 otherwise

IMPORTANT: The output of ccRemover does not require log transformation before clustering analysis.

```
#N.B. ccRemove function requires as input raw counts table previously anntoated with scannobyGtf function

#removing cell cycle effect
ccRemove(group="docker" , scratch.folder="/data/scratch",
        file=paste(getwd(),"annotated_buettner_G1G2MS_counts.txt", sep="/"), separator="\t",
        seed=111, cutoff=3, species="mouse", rawCount=1)
```

The analyses above were done using a SeqBox, equipped with an Intel i7-6770HQ (8 threads), 32 GB RAM and 500 GB SSD. They took in total 54 and 40 mins for recatPrediction respectively on Buettner and the naive T-cells datasets. 28 mins were needed by ccRemove on Buettner data set. ccRemover analysis produces a ready-for-clustering data normalized matrix. The matrix can be identified by the prefix **LS_cc_**. **ccRemove** output is compliant with *SIMLR*, the rCASC core clustering tool. **ccRemove** output does NOT require log transformation when applied to SIMLR.

```
#visualizing the dataset before and after cell cycle bias removal
#reformat the matrix header to be suitable with docker4seq PCA plotting function
tmp <- read.table("annotated_buettner_G1G2MS_counts.txt", sep="\t", header=T, row.names=1)
tmp.n <- strsplit(names(tmp), "_")
tmp.n1 <- sapply(tmp.n, function(x)x[1])
tmp.n2 <- sapply(tmp.n, function(x)x[2])
names(tmp) <- paste(tmp.n2, tmp.n1, sep="_")
write.table(tmp, "annotated_buettner_G1G2MS_countsbis.txt", sep="\t", col.names=NA)

library(devtools)
install_github("kendomaniac/docker4seq", ref="master")
library(docker4seq)
#N/B. setting type parameter to "counts" data will be log10 transformed before PCA analysis
```

```
pca(experiment.table="annotated_buettner_G1G2MS_countsbis.txt", type="counts",
      legend.position="topright", covariatesInNames=TRUE, samplesName=FALSE,
      principal.components=c(1,2), pdf = TRUE,
      output.folder=getwd())

#reformat the matrix header to be suitable with docker4seq PCA plotting function
tmp <- read.table("LS_cc_annotated_buettner_G1G2MS_counts.txt", sep="\t", header=T, row.names=1)
tmp.n1 <- sapply(tmp.n, function(x)x[1])
tmp.n2 <- sapply(tmp.n, function(x)x[2])
names(tmp) <- paste(tmp.n2, tmp.n1, sep="_")
write.table(tmp, "LS_cc_annotated_buettner_G1G2MS_countsbis.txt", sep="\t", col.names=NA)

pca(experiment.table="LS_cc_annotated_buettner_G1G2MS_countsbis.txt", type="TPM",
      legend.position="topright", covariatesInNames=TRUE, samplesName=FALSE,
      principal.components=c(1,2), pdf = TRUE,
      output.folder=getwd())
```

In Figure 30 are shown the results obtained using the ccRemove implementation in rCASC, using the Buettner dataset. The removal of the cell cycle effect (Figure 30B) is clearly shown by a reduction of the variance explained by PC1 and PC2 in the PCA plot.

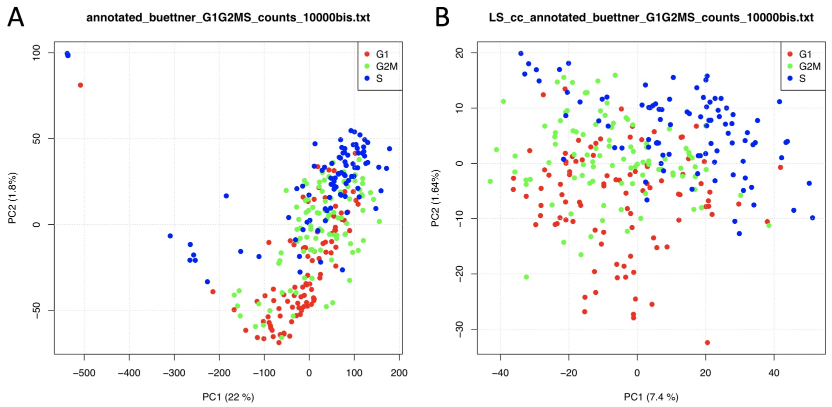

Figure 30: rCASC implementation of the ccRemove. A) PCA analysis of Buettner et al. (Nat. Biotechnol. 2015) log10 transformed raw data, B) PCA analysis of ccRemove cell-cycle normalized dataset.

Section 4 Estimating the number of clusters to be used for cell sub-population discovery.

The rCASC core clustering tool is *SIMLR*, which requires as input the number of clusters to be used to aggregate cell sub-populations. Unfortunately, there is no definitive answer to the definition of the most probable number of clusters, in which cells will aggregate. Some of the possible ways to identify the most probable number of clusters is summarised in: *"Determining the optimal number of clusters: 3 must known methods - Unsupervised Machine Learning"*.

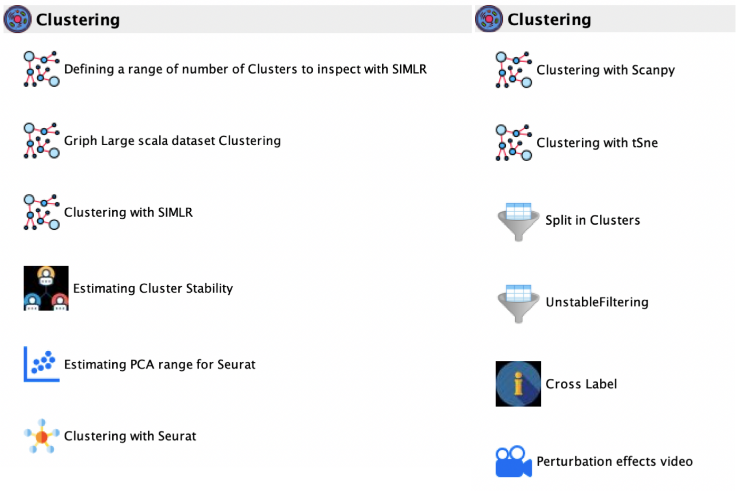

Figure 31: GUI: Clustering panel

Another important aspect is how the number of detectable clusters might change if the number of cells changes in the dataset, e.g. upon removal of a random subset of cells. Because single-cell experiment, at least today, are rarely characterized by biological replications and frequently they represent the initial step of an analysis aimed at the identification for new cell sub-populations, it is very important to assess the stability of cells aggregations detected by clustering methods.

Section 4.1 Estimating the number of cluster to be used for cell sub-population discovery by community detection method.

In rCASC, the identification of the optimal number of clusters is addressed, in presence of cells number perturbations, with *griph*. The clustering performed by griph is graph-based and uses the community detection method *Louvain modularity*. Griph algorithm is closer to agglomerative clustering methods, since every node is initially assigned to its own community and communities are subsequently built by iterative merging. Griph is embedded **clusterNgriph** function, which evaluates the number of clusters in which a set of cells will aggregate upon a user defined leave-N%-out cells bootstraps. In the example below the number of clusters are detected for the file ‘annotated_buettner_G1G2MS_counts_10000bis.txt’, used in **Section 3.8**.

- *Parameters* (only those without default; for the full list of parameters please refer to the function help) (Figure 32):
  - *scratch.folder*, the path of the scratch folder
  - *file (GUI Matrix counts)*, the path to the input file, including the counts table name
  - *nPerm*, number of permutations to perform
  - *permAtTime*, number of permutations that can be computed in parallel
  - *percent*, percentage of randomly selected cells removed in each permutation
  - *separator*, separator used in count file, e.g. ‘\t’, ‘,’

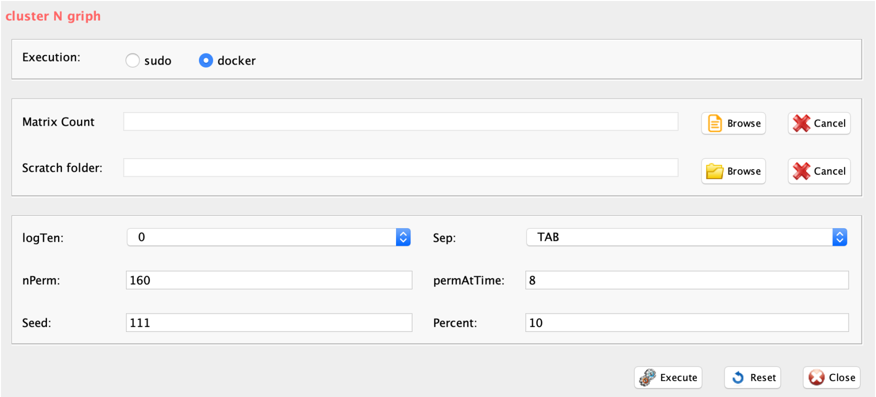

Figure 32: GUI: Range of numbers of clusters estimation panel

- *logTen*, integer, 1 if the count matrix is already in log10, 0 otherwise
- *seed*, important value to reproduce the same results with same input, default is 111

```
#N.B. If the input is a raw count table, before griph analysis data are log10 transformed

library(rCASC)
clusterNgriph(group="docker",scratch.folder="/data/scratch/",file=paste(getwd(),
              "annotated_buettner_G1G2MS_counts_10000bis.txt", sep="/"), nPerm=160,
              permAtTime=8, percent=10, separator="\t",logTen=0, seed=111)
```

In Figure 33 it is shown the output generated by **clusterNgriph**. The output folder is called **Results** and it is located in the folder from which the analysis started. Within Results is present a folder named as the dataset used for the analysis. In this case 'annotated_buettner_G1G2MS_counts_10000bis'. In the latter folder is present a folder, in this specific example '5', named with the number of clusters that were more represented as result of the bootstrap analysis. The file indicated with the blue arrow contains all the information to generate the griph output plot, used as reference to allocate cells to a specific cluster at each bootstrap step. The file indicated with the green arrow contains the cluster position for each cell over all bootstrap steps. The file indicated with the red arrow contains the cells removed at each bootstrap step. The file called 'hist.pdf', indicated with the black arrow, is the plot of the frequency of different number of clusters generated by griph as consequence of the bootstrap steps. In this specific case, over 160 permutations, 80 produced 5 clusters, 70 produced 4 clusters and 10 produced 6 clusters.

It has to be noted that in principle, since this dataset has a strong cell cycle effect, we would have expected ideally only three clusters: G1, S and G2M. This toy experiment clearly shows that perturbation of the dataset under analysis can affect the number of detectable clusters. Thus, to identify the clustering condition which guarantees the greatest cell stability in a cluster, in our opinion it is mandatory clustering cells taking in account perturbation effects. In Section 4.2 we further investigate this issue.

Section 4.2 K-mean clustering: Investigating how cell sub-populations aggregation is affected by dataset perturbations.

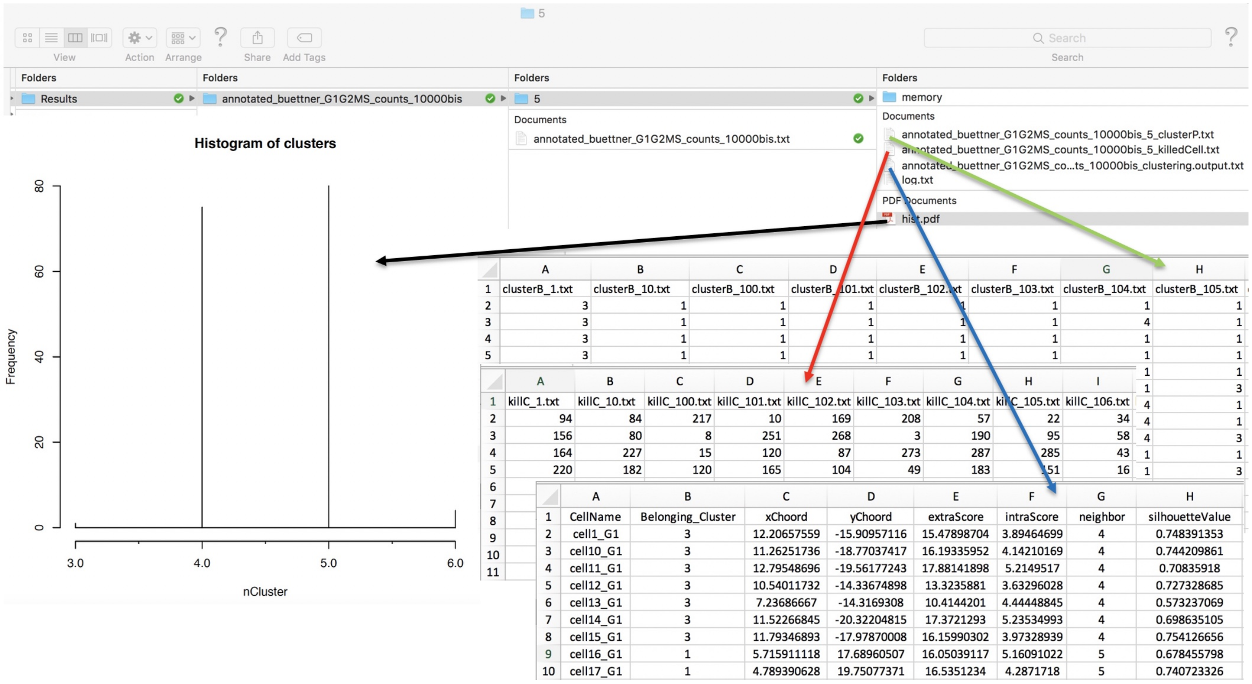

Figure 33: Output of clusterNgriph

As indicated above we are interested to identify not only the optimal cluster number but also if the cluster number is affected by removal of a random subset of cells. To observe the effect of datasets perturbation in clustering we built 4 datasets combining different cell types available in *Zheng* 2016 paper (Bold cell types are those that were progressively substituted in setA):

- setA 100 cells randomly selected for each cell type:
  - (B) B-cells (25K reads/cell), (M) Monocytes (100K reads/cell), (S) Stem cells (24.7K reads/cell), (NK) Natural Killer cells (29K reads/cell), (N) Naive T-cells (19K reads/cell)
- setB 100 cells randomly selected for each cell type:
  - (B) B-cells, (M) Monocytes, (H) **T-helper cells** (21K reads/cell), (NK) Natural Killer, (N) Naive T-cells
- setC 100 cells randomly selected for each cell type:
  - (C) **Cytotoxic T-cells** (28.6K reads/cell), (M) Monocytes, **T-helper cells**, (NK) Natural Killer, (N) Naive T-cells
- setD 100 cells randomly selected for each cell type:
  - (C) **Cytotoxic T-cells**, (NC) **Naive cytotoxic T-cells** (20K reads/cell), (H) **T-helper cells**, (NK) Natural Killer, (N) Naive T-cells

Moving from SetA to setD we added progressively cells coming from T-cell populations, making the cell-type partitioning more challenging because of the similarities between T-cell sub-populations.

We used PCA (Figure 34A-D) to visualize the dissimilarity between cells populations. PCA measures the variance between the elements of the dataset and the most important differences in variance are estimated by

the PC1.

```
system("wget http://130.192.119.59/public/section4.1_examples.zip")
unzip("section4.1_examples.zip")
system("cd section4.1_examples")

#visualizing the complexity of the datasets using PCA
library(docker4seq)
topx(group="docker",file=paste(getwd(),"bmsnkn_5x100cells.txt", sep="/"),threshold=1000, logged=FALSE, type="expression", separa
#converting filtered data in log10
counts2log(file=paste(getwd(), "filtered_expression_bmsnkn_5x100cells.txt", sep="/"), log.base=10)



#N.B. if the type parameter is set to FPKM or TPM, it is assumed that data are already log10 transformed
pca(experiment.table="filtered_expression_bmsnkn_5x100cells.txt", type="FPKM",
    legend.position="topleft", covariatesInNames=TRUE, samplesName=FALSE,
    principal.components=c(1,2), pdf = TRUE,
    output.folder=getwd())

topx(data.folder=getwd(),file.name="bmHnkn_5x100cells.txt",threshold=1000, logged=FALSE, type="expression", separator="\t")
counts2log(file=paste(getwd(), "filtered_expression_bmHnkn_5x100cells.txt", sep="/"), log.base=10)

pca(experiment.table="filtered_expression_bmHnkn_5x100cells.txt", type="FPKM",
    legend.position="topleft", covariatesInNames=TRUE, samplesName=FALSE,
    principal.components=c(1,2), pdf = TRUE,
    output.folder=getwd())

topx(data.folder=getwd(),file.name="CmHnkn_5x100cells.txt",threshold=1000, logged=FALSE, type="expression", separator="\t")
counts2log(file=paste(getwd(), "filtered_expression_CmHnkn_5x100cells.txt", sep="/"), log.base=10)

pca(experiment.table="filtered_expression_CmHnkn_5x100cells.txt", type="FPKM",
    legend.position="topleft", covariatesInNames=TRUE, samplesName=FALSE,
    principal.components=c(1,2), pdf = TRUE,
    output.folder=getwd())

topx(data.folder=getwd(),file.name="CNCHnkn_5x100cells.txt",threshold=1000, logged=FALSE, type="expression", separator="\t")
counts2log(file=paste(getwd(), "filtered_expression_CNCHnkn_5x100cells.txt", sep="/"), log.base=10)

pca(experiment.table="filtered_expression_CNCHnkn_5x100cells.txt", type="FPKM",
    legend.position="topleft", covariatesInNames=TRUE, samplesName=FALSE,
    principal.components=c(1,2), pdf = TRUE,
    output.folder=getwd())
```

PC1 shows that, as the differences between cell populations become smaller, moving from setA to setD, the aggregation in homogeneous groups of cells is compromised (Figure 34A-D).

To observe the effect of the reduced dissimilarity between populations on the stability of the number of clusters, we use the rCASC **clusterNgriph** function on the above mentioned 4 datasets using 160 permutations/each, and randomly removing in each permutation 10% of the cells. Each analysis took approximately 60 mins on

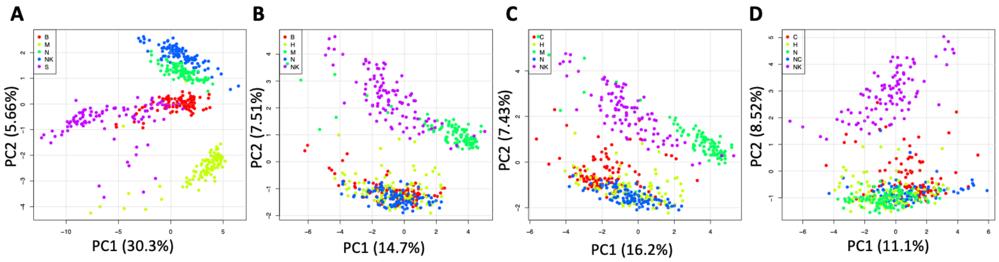

Figure 34: PCA is getting progressively unable to discriminate between the different cell subpopulations as the set of cells are getting functionally more similar to each other: A) PCA of setA, B) PCA of setB, C) PCA of setC, D) PCA of setD.

a SeqBox hardware.

```
#downloading datasets
system("wget http://130.192.119.59/public/section4.1_examples.zip")
unzip("section4.1_examples.zip")
setwd("section4.1_examples")

#setA
clusterNgriph(group="docker",scratch.folder="/data/scratch/",file=paste(getwd(),
              "bmsnkn_5x100cells.txt", sep="/"), nPerm=160, permAtTime=8,
              percent=10, separator="\t",logTen=0, seed=111)

#setB
clusterNgriph(group="docker",scratch.folder="/data/scratch/",file=paste(getwd(),
              "bmHnkn_5x100cells.txt", sep="/"), nPerm=160,
              permAtTime=8, percent=10, separator="\t",logTen=0, seed=111)

#setC
clusterNgriph(group="docker",scratch.folder="/data/scratch/",file=paste(getwd(),
              "CmHnkn_5x100cells.txt", sep="/"), nPerm=160,
              permAtTime=8, percent=10, separator="\t",logTen=0, seed=111)

#setD
clusterNgriph(group="docker",scratch.folder="/data/scratch/",file=paste(getwd(),
              "CNCHnkn_5x100cells.txt", sep="/"), nPerm=160,
              permAtTime=8, percent=10, separator="\t",logTen=0, seed=111)
```

It is notable that as the differences between the cells populations is narrowing (i.e in setA cells types are quite different in overall functional activity, as in setD four out of five cell types are T-cells sub-populations) the fluctuations in the detected number of clusters increase. In setA, where PCA is able to discriminate between the five cell populations (Figure 34A), out of 160 bootstraps only 1 gave a number of clusters different from the effective number of cell sub-populations (Figure 35A). In all the other three subsets perturbations result in a higher number of events in which number of clusters differs from 5 (Figure 35B-D).

As highlighted in **Section 3.2.1**, the difficulties in detecting a stable number of clusters, upon dataset

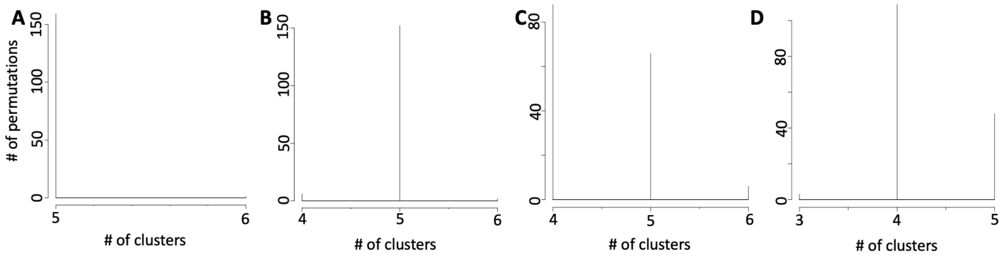

Figure 35: Clusters number is dependent by the cell type similarity: A) number of clusters detectable by griph in setA, B) number of clusters detectable by griph in setB, C) number of clusters detectable by griph in setC, D) number of clusters detectable by griph in setD

perturbations, is due to the limited number of detected genes. To further support this hypothesis, we run a comparison between the most expressed genes in the cell types used in the above example.

```
#downloading datasets
#section4.1_examples.zip contains raw counts from Zheng experiment available at 10XGenomics web site
#100 cells  were randomly extracted from from each sorted cell type dataset.
system("wget http://130.192.119.59/public/section4.1_examples.zip")
unzip("section4.1_examples.zip")
setwd("section4.1_examples")

#loading the datasets used to generate PCA
b <- read.table("cd19_b_100cell.txt", sep="\t", header=T, row.names=1)
mono <- read.table("cd14_mono_100cell.txt", sep="\t", header=T, row.names=1)
stem <- read.table("cd34_stem_100cell.txt", sep="\t", header=T, row.names=1)
nk <- read.table("cd56_nk_100cell.txt", sep="\t", header=T, row.names=1)
naiveT <- read.table("naiveT_100cell.txt", sep="\t", header=T, row.names=1)
cyto <- read.table("cytoT_100cell.txt", sep="\t", header=T, row.names=1)
naiveCyto <- read.table("naiveCytoT_100cell.txt", sep="\t", header=T, row.names=1)
helper <- read.table("cd4_h_100cell.txt", sep="\t", header=T, row.names=1)

#calculating the gene-level expression and ranking the genes from the most expressed to the least expressed
b.s <- sort(apply(b,1,sum), decreasing=T)
mono.s <- sort(apply(mono,1,sum), decreasing=T)
stem.s <- sort(apply(stem,1,sum), decreasing=T)
nk.s <- sort(apply(nk,1,sum), decreasing=T)
naiveT.s <- sort(apply(naiveT,1,sum), decreasing=T)
cyto.s <- sort(apply(cyto,1,sum), decreasing=T)
naiveCyto.s <- sort(apply(naiveCyto,1,sum), decreasing=T)
helper.s <- sort(apply(helper,1,sum), decreasing=T)

#function that measure the identity between lists of increasing lengths
overlap <- function(x,y){
  overlap.v <- NULL
  for(i in 1:length(x)){
     overlap.v[i] <- length(intersect(x[1:i], y[1:i]))
  }
```

```
  return(overlap.v)
}

#calculating the level of identity between lists of increasing lengths all comparisons are run with respect to naive T-cells.
naiveCyto.naiveT <- overlap(names(naiveT.s), names(naiveCyto.s))
b.naiveT <- overlap(names(naiveT.s), names(b.s))
mono.naiveT <- overlap(names(naiveT.s), names(mono.s))
stem.naiveT <- overlap(names(naiveT.s), names(stem.s))
nk.naiveT <- overlap(names(naiveT.s), names(nk.s))
naiveCyto.naiveT <- overlap(names(naiveT.s), names(naiveCyto.s))
helper.naiveT <- overlap(names(naiveT.s), names(helper.s))
cyto.naiveT <- overlap(names(naiveT.s), names(cyto.s))

#plotting the above data
plot(seq(1, 500), seq(1, 500), type="l", col="black", lty=2)
points(seq(1, 500), naiveCyto.naiveT[1:500], type="l", col="black")
points(seq(1, 500), cyto.naiveT[1:500], type="l", col="blue")
points(seq(1, 500), helper.naiveT[1:500], type="l", col="green")
points(seq(1, 500), mono.naiveT[1:500], type="l", col="red")
points(seq(1, 500), b.naiveT[1:500], type="l", col="brown")
points(seq(1, 500), stem.naiveT[1:500], type="l", col="orange")
points(seq(1, 500), nk.naiveT[1:500], type="l", col="violet")
legend("topleft", legend=c("Naive T-cytotoxic", "T-cytotoxic", "T-helper", "Monocytes", "B-cells", "Stem cells", "NK"),
       pch=15, col=c("black", "blue", "green", "red", "brown", "orange", "violet"))
```

Figure 36 shows the number of identical genes found in common between naive T-cells and the other sub-populations in setA and setD, using lists of increasing size and ordered by expression level. The plot shows that naive T-cytotoxic, T-cytoxic and T-helper, from setD, share with naive T-cells, within the top 500 most expressed genes, more genes with respect to the other cell types present in setA. Thus, the lack of cell-type specific genes between 4 out 5 cell types in SetD dataset negatively affect the stability dataset partitioning, upon bootstraps.

The results described in this section indicate that **clusterNgriph** is a valuable instrument to define a range of numbers of clusters to be further investigated with supervised clustering approaches.

Section 5 K-mean clustering: detecting cell sub-populations by mean of kernel based similarity learning (*SIMLR*).

The number of clustering and dimension reduction methods for single cell progressively increased over the last few years. Last year *Wang and coworkers* published SIMLR, a framework which learns a similarity measure from single-cell RNA-seq data in order to perform dimensions reduction. We decided to select this method as core clustering tool in rCASC, because outperformed eight methods published before 2017 [*Wang and coworkers*].

Specifically, we use SIMLR as clustering method recording the effects of data perturbation, i.e. removal of random subset of cells, on the clustering structure. Although, we think SIMLR provides important advantages with respect to other clustering methods, rCASC framework can embed also other data reduction tools. At the present time, tSne is also implemented within the rCASC data permutation framework.

One of the peculiarities of rCASC is the user tunable bootstrap procedure. rCASC represents bootstrap results via a cell stability score (Figure 37). In brief, a set of cells to be organized in clusters (Figure 37A) is analyzed with SIMLR, applying a user defined k number of clusters (Figure 37B). A user defined % of cells is removed from the original data set and these cells are clustered again (Figure 37C). The clusters obtained in each bootstrap step are compared with the clusters generated on the full dataset using Jaccard index (Figure 37D-E). If the Jaccard index is greater of a user defined threshold, e.g. 0.8, the cluster is called confirmed in the bootstrap step (Figure 37F). Then to each cell, belonging to the confirmed cluster, *cell stability score* value is increased of 1 unit (Figure 37G). At the end of the bootstrap procedure, cells are labeled with different symbols describing their *cell stability score* in a specific cluster (Figure 37H).

Section 5.1 Cell Stability Score: mathematical description.

Stability score Algorithm Let be C the count matrix with $N \times M$ dimension where N is the gene number and M is cell number.

$$C = \begin{bmatrix} c_{11} & c_{12} & \cdots \\ \vdots & \ddots & \\ c_{N1} & & c_{NM} \end{bmatrix}$$

Then, we define C^p the matrix generated by p^{th} permutation removing q random columns from the original matrix C. Moreover considering the pth permutation we denote L^p the set of all the removed cells in p^{th} permutation with $|L^p| = q$ and $\mathbf{cl}^p$ the vector with length $M - q$ encoding the relation between cells and clusters in p. Hence, $\mathbf{cl}_i^p$ identified the cluster in which the ith cell is inserted in permutation p. Finally, we use the notation $\mathbf{cl}^C$ for indicating the output of the clustering algorithm obtained by all the cells (i.e. matrix C).

The relation symmetric matrix R^p with dimension $M \times M$ is defined as follows:

$$R^p = \begin{bmatrix} r^p_{1,1} & r^p_{1,2} & \cdots \\ \vdots & \ddots & \\ r^p_{M,1} & & r^p_{M,M} \end{bmatrix}$$

where $r^p_{i,j}$ is:

$$r^p_{i,j} = \begin{cases} 1 \; if \; \mathbf{cl}_i^p = \mathbf{cl}_j^p \\ 0 \; otherwise \end{cases} \tag{1}$$

Similarly we defined R^C the relation symmetric matrix obtained considering all cells and R^{C-L^P} as the relation symmetric matrix R^C in which the columns and the rows associated with cells in L^p are removed. Observe that the sum $R^{C-L^P} + R^p$ will always gives as results a matrix with 3 possible values: 0,1 or 2.

$$R^{C-L^p}[j,i] + R^p[j,i] = \begin{cases} 1 & if\ cell\ i\ and\ cell\ j\ clustered\ together\ in\ R^{C-L^p} \\ & or\ in\ R^P\ only; \\ 2 & if\ cell\ i\ and\ cell\ j\ are\ always\ in\ the\ same\ cluster; \\ 0 & if\ cell\ i\ and\ cell\ j\ are\ never\ in\ the\ same\ cluster. \end{cases}$$

Let $\delta(i,k)$ be the kronecker delta, a function of two variables returning 1 if the variables are equal, and 0 otherwise:

$$\delta(i,k) = \begin{cases} 1 \; if \; i = k \\ 0 \; otherwise \end{cases} \tag{2}$$

then we define the function *length* that counts the occurrence of a value k fixing the row j in the matrix $R^{C-L^P} + R^p$.

$$length(j,p,k) = \sum_{i=1}^{M} \delta(R^{C-L^P}[j,i] + R^p[j,i], k) \tag{3}$$

We use the function *length* to count the occurrence of 1 or 2 in in the matrix $R^{C-L^P} + R^p$.

Finally, we define the permutation score $pscore_{j,p}$ as:

$$pscore_{j,p} = \frac{length(j,p,2)}{length(j,p,2) + length(j,p,1)} \tag{4}$$

where p is a permutation and j a cell. Then, this returns the percentage of cells initially clustered with cell j that remain clustered with cell j in the permutation p.

Then, we define $tscore_{j,s}$ as follow:

$$tscore_{j,s} = \frac{1}{P} \sum_{p \in P} 1_{pscore_{j,p} >= s} \tag{5}$$

where P is the total number of permutations and s is user-defined threshold. This metric compute the probability that a cell j is always clustered with the same set of cells given that $pscore_{j,p} >= s$.

1 Example

Be $\mathbf{cl} = \begin{Bmatrix} 1 & 2 & 2 & 1 & 2 & 1 \end{Bmatrix}$

Be $\mathbf{L} = \begin{Bmatrix} 6 & 2 & 2 & 4 \end{Bmatrix}$

Be $\mathbf{cl}^1 = \begin{Bmatrix} 1 & 2 & 1 & 1 & 2 \end{Bmatrix}$

Be $\mathbf{cl}^2 = \begin{Bmatrix} 1 & 2 & 1 & 2 & 2 \end{Bmatrix}$

Be $\mathbf{cl}^3 = \begin{Bmatrix} 1 & 2 & 1 & 1 & 2 \end{Bmatrix}$

Be $\mathbf{cl}^4 = \begin{Bmatrix} 1 & 2 & 2 & 1 & 2 \end{Bmatrix}$

$\forall\ p \in \{1,2,3,4\},\ R_p$ is calculated
for instance hereafter I reported R and R^1

$$R = \begin{bmatrix} 1 & 0 & 0 & 1 & 0 & 1 \\ 0 & 1 & 1 & 0 & 1 & 0 \\ 0 & 1 & 1 & 0 & 1 & 0 \\ 1 & 0 & 0 & 1 & 0 & 1 \\ 0 & 1 & 1 & 0 & 1 & 0 \\ 1 & 0 & 0 & 1 & 0 & 1 \end{bmatrix}$$

$$R^1 = \begin{bmatrix} 1 & 0 & 1 & 1 & 0 \\ 0 & 1 & 0 & 0 & 1 \\ 1 & 0 & 1 & 1 & 0 \\ 1 & 0 & 1 & 1 & 0 \\ 0 & 1 & 0 & 0 & 1 \end{bmatrix}$$

$\forall\ p\ R^{p'} + R^p\ is\ calculated$
for instance hereafter I reported $R^{p'} + R^1$

$$R'^1 + R^1 = \begin{bmatrix} 2 & 0 & 1 & 2 & 0 \\ 0 & 2 & 1 & 0 & 2 \\ 1 & 1 & 2 & 1 & 1 \\ 2 & 0 & 1 & 2 & 0 \\ 0 & 2 & 1 & 0 & 2 \end{bmatrix}$$

$\forall\ p,\ pscore\ is\ evaluated\ with\ S = 0.6$ for instance hereafter I reported $pscore_1$

$$pscore_1 = \begin{bmatrix} 1 \\ 1 \\ 0 \\ 1 \\ 1 \end{bmatrix}$$

This means that in permutation 1 the third cell is unstable ”jumping” from cluster number 1 to cluster number 2 in P. $\text{tscore}_{m,s}$ is evaluated then for

each cell $tscore_s = \begin{bmatrix} 0 \\ 0 \\ 0 \\ 0.25 \\ 0.25 \\ 0 \end{bmatrix}$

In this Example number of permutation is 4, for statistical relevance, an higher number of permutation is required.

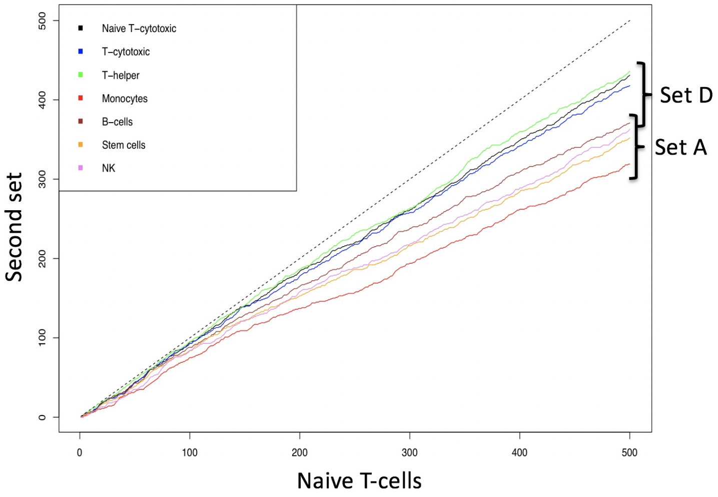

Figure 36: Identity between naive T-cells and the other cell types in set A and D in gene lists of increasing length. Identity between two data sets is shown by the dashed line.

SIMLR is embedded in **simlrBootstrap** function within the rCASC bootstrap framework.

- *Parameters* (only those without default; for the full list of parameters please refer to the function help) (Figure 38):
  - *scratch.folder*, the path of the scratch folder
  - *file (GUI Counts table)*, the path of the file, with file name and extension included
  - *nPerm*, number of permutations to be executed
  - *permAtTime*, number of permutations computed in parallel
  - *percent*, percentage of randomly selected cells removed in each permutation
  - *range1*, beginning of the range of clusters to be investigated
  - *range2*, end of the range of clusters to be investigated
  - *separator*, separator used in count file, e.g. '\t', ','
  - *logTen*, 1 if the count matrix is already in log10, 0 otherwise
  - *seed*, important value to reproduce the same results with same input, default is 111
  - *sp*, minimum number of percentage of cells that has to be in common in a cluster, between two permutations, default 0.8
  - *clusterPermErr*, probability error in depicting the number of clusters in each permutation, default = 0.05

```
system("wget http://130.192.119.59/public/section4.1_examples.zip")
unzip("section4.1_examples.zip")
setwd("section4.1_examples")
library(rCASC)
#annotating data setA
#annotating data setA
system("wget ftp://ftp.ensembl.org/pub/release-94/gtf/homo_sapiens/Homo_sapiens.GRCh38.94.gtf.gz")
```

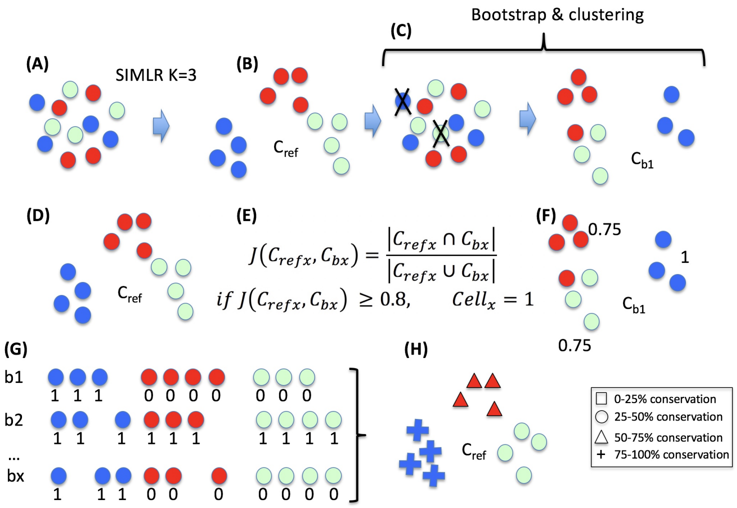

Figure 37: Cell stability score

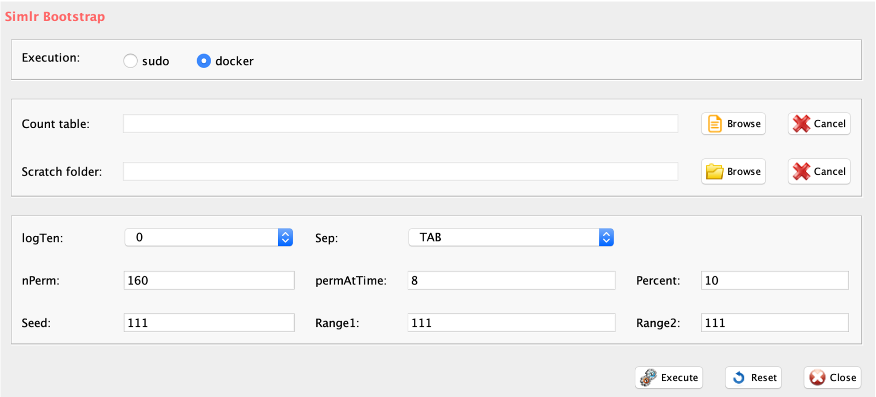

Figure 38: GUI: SIMLR clustering panel

```
system("gzip -d Homo_sapiens.GRCh38.94.gtf.gz")
system("mv Homo_sapiens.GRCh38.94.gtf genome.gtf")

scannobyGtf(group="docker", file=paste(getwd(),"bmsnkn_5x100cells.txt",sep="/"),
                  gtf.name="genome.gtf", biotype="protein_coding",
                  mt=TRUE, ribo.proteins=TRUE,umiXgene=3, riboStart.percentage=0,
                  riboEnd.percentage=100, mitoStart.percentage=0, mitoEnd.percentage=100, thresholdGenes=100)
#running SIMLR analysis using the range of clusters suggested by clusterNgriph in session 4.2
#N.B. if raw counts are provide as input data will be log10 transformed before SIMLR analysis
simlrBootstrap(group="docker",scratch.folder="/data/scratch/",
               file=paste(getwd(), "annotated_bmsnkn_5x100cells.txt", sep="/"),
               nPerm=160, permAtTime=8, percent=10, range1=4, range2=6,
               separator="\t",logTen=0, seed=111)

#annotating data setB
scannobyGtf(group="docker", file=paste(getwd(),"bmHnkn_5x100cells.txt",sep="/"),
                  gtf.name="genome.gtf", biotype="protein_coding",
```

```
                    mt=TRUE, ribo.proteins=TRUE,umiXgene=3, riboStart.percentage=0,
                    riboEnd.percentage=100, mitoStart.percentage=0, mitoEnd.percentage=100, thresholdGenes=100)
#running SIMLR analysis using the range of clusters suggested by clusterNgriph in session 4.2
simlrBootstrap(group="docker",scratch.folder="/data/scratch/",
               file=paste(getwd(), "annotated_bmHnkn_5x100cells.txt", sep="/"),
               nPerm=160, permAtTime=8, percent=10, range1=4, range2=6,
               separator="\t",logTen=0, seed=111)
```

The output of **simlrBootstrap** function is described in Figure 39. The output is localized in the *Results* folder (Figure 39 Folder 1), which is created in the folder where the analysis started. This folder contains the count matrices used in the analyses. In this example the count matrices are those belonging to setA and B (see Section 4.1). In the *Results* folder are also present folders labeled with the name of the count matrix used in the analysis (Figure 39 Folder 2, annotated_bmsnkn_5x100cells, annotated_bmHnkn_5x100cells). In each of these folders there are a set of folders indicated with a number that refers to the analyzed range of number of clusters (Figure 39 Folder 3). In this specific example the range of clusters goes from 4 to 6. In each *NameOfCountMatrix* folder there is a file called **_Stability_Violin_Plot.pdf** (Figure 39A) which represents the distribution of the cells stability scores over the bootstraps in the range of number of clusters investigated. Figure 39A clearly show that the analysis done with k=5 is the one providing the highest stability of cells within each cluster. The other plot that is also available in the folder (Figure 39 Folders 4) is **NameOfCountMatrix_vioplot.pdf**, Figure 39B, which contains the distribution of the *Silhouette* values for each cluster over the bootstraps. *Silhouette* value is a measure of computation cluster stability, and evaluates how similar an object is to its own cluster (cohesion) compared to other clusters (separation). Clearly the information provided by Silhouette plot is much less informative for the definition of the optimal clustering number with respect to the information provided by the cells stability score (Figure 39 blue arrow). In each clustering folder there is a pdf named *NameOfCountMatrix_Stability_Plot.pdf*, which contains two plots (Figure 39C-D) generated by the clustering program. These plots provide a 2D view of the clustering results from two different perspectives. In Figure 39C plot each cell is colored on the basis of the belonging cluster and it is labeled with a symbol indicating its cell stability score (CSS). Instead, in the plot in Figure 39D each cell is colored on the basis of its CSS: 0-25% black, 25-50% green, 50-75% gold and 75-100% red. Here, we show the plots generated with k=6 to better describe the information described in the above-mentioned clusters plots, as in k=5 clusters all cells have a CSS greater than 75%. In Figures 39C and D it is shown that 4 out of 6 clusters cells remain in a cluster between 75 to 100% of the bootstraps (+ symbol in Figure 39C and red dots in Figure 39D). The plot, Figure 39E, shows the genes detectable in each cell in function of the total number of reads/cell. In this plot cells are colored with the same color of their belonging cluster. This plot is useful to observe if the clustering is biased by the number of genes called in each cluster. In this specific example, only the green cluster is characterized by a number of detected genes, which is larger of those detectable in the other clusters (Figure 39E). This is expected, since blue cluster is made of Monocytes, which have been sequenced to 100K reads/cells, as all other cells in setA were sequenced between 19 to 29K reads/cell, see **Section 4.2**.

In Figure 40 are shown the effects, on cell stability, in SIMLR analysis done with 4 and 6 clusters for SetA

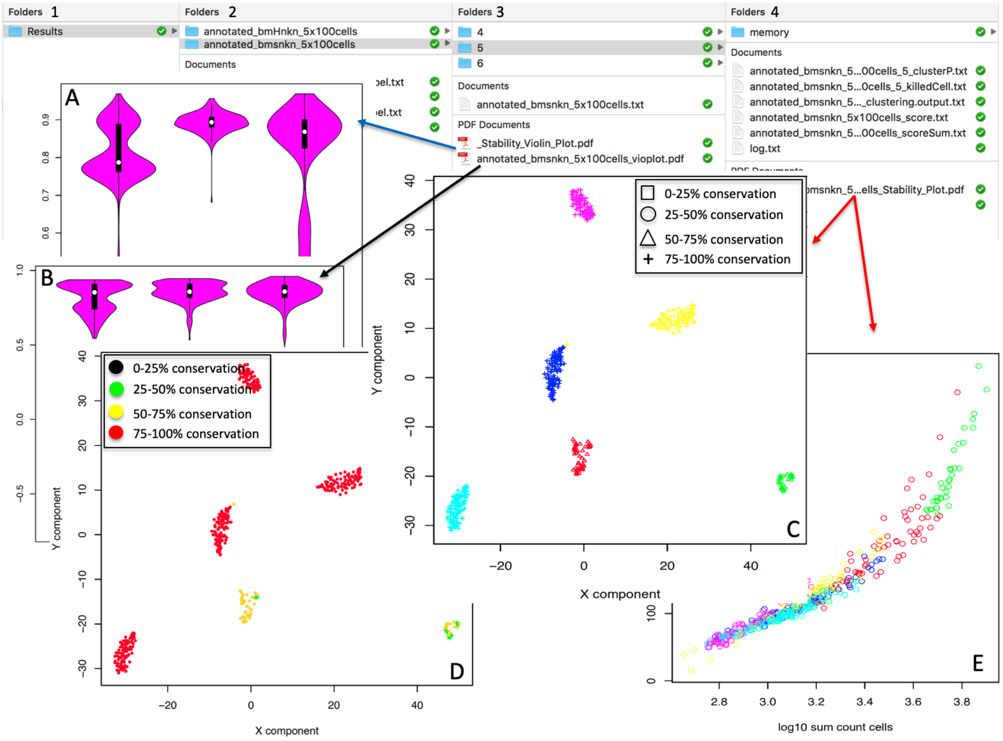

Figure 39: simlrBootstrap output. A) Cell Stability violin plot for the range of investigated clusters. B) Silhouette violin plot for the range of investigated clusters. C) SIMLR analysis: cells are partitioned in 6 cluster, defined by different colors. D) Cells colored on the basis of their Stability Score. E) Genes versus UMI counts, where cells are colored on the basis of their belonging cluster

(*annotated_bmsnkn_5x100cells.txt*) counts matrix. Using 4 as number of clusters, Figure 40A, some of the cells in each cluster show a reduced stability (50-75%, triangle) and arrows highlights cells characterized by a cell stability score between 25 to 50%. The circles in Figure 40B show the clusters where the full cluster has a cell stability between 50 to 75%. This observation indicates that the stability score is a useful measure to identify the optimal number of partitions to be used, i.e. the highest cell stability score was observed when five clusters were selected, corresponding to the number of cell types combined in SetA, see **Section 4.2**.

The effect of the perturbations induced in the clustering upon the removal of 10%, 20%, 30% and 50% of the data set was also investigated in SetA (*annotated_bmsnkn_5x100cells.txt*), Figure 41.

```
#running SIMLR analysis using the range of clusters suggested by clusterNgriph in session 4.2
simlrBootstrap(group="docker",scratch.folder="/data/scratch/",
               file=paste(getwd(), "annotated_bmHnkn_5x100cells.txt", sep="/"),
               nPerm=160, permAtTime=8, percent=10, range1=4, range2=6,
               separator="\t",logTen=0, seed=111)


simlrBootstrap(group="docker",scratch.folder="/data/scratch/",
               file=paste(getwd(), "annotated_bmHnkn_5x100cells.txt", sep="/"),
               nPerm=160, permAtTime=8, percent=20, range1=5, range2=5,
```

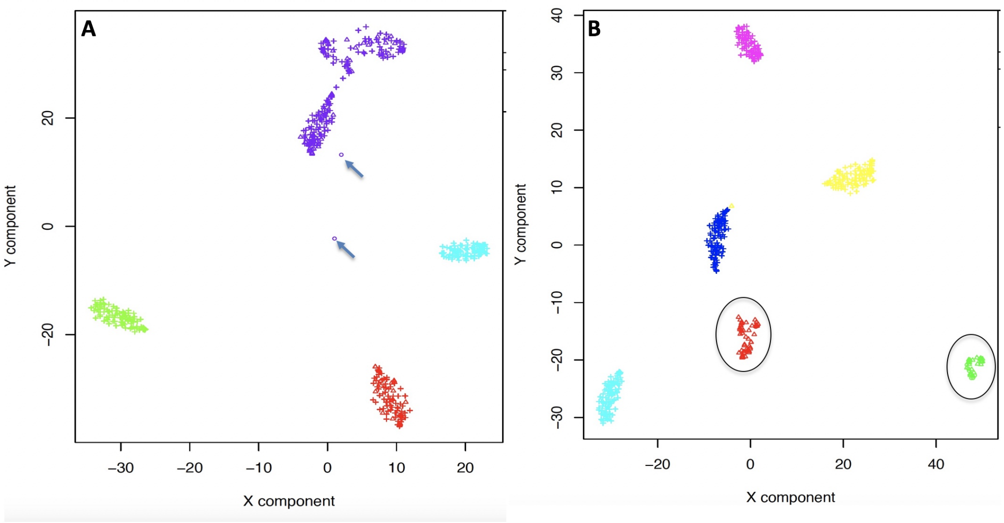

Figure 40: Clustering output including cell stability score. Different in stability observable between the partition of cells in 5 (A) or 6 clusters (B). Partition in 5 clusters is more stable upon perturbation than partition in 6 clusters. Circles indicates clusters in which CSS is between 50-75. All other clusters have CSS between 75 to 100. Arrows indicate the cells with CSS between 25-50.

```
               separator="\t",logTen=0, seed=111)

simlrBootstrap(group="docker",scratch.folder="/data/scratch/",
               file=paste(getwd(), "annotated_bmHnkn_5x100cells.txt", sep="/"),
               nPerm=160, permAtTime=8, percent=30, range1=5, range2=5,
               separator="\t",logTen=0, seed=111)

simlrBootstrap(group="docker",scratch.folder="/data/scratch/",
               file=paste(getwd(), "annotated_bmHnkn_5x100cells.txt", sep="/"),
               nPerm=160, permAtTime=8, percent=50, range1=5, range2=5,
               separator="\t",logTen=0, seed=111)
```

Increasing the fraction of cells removed at each permutation affects, in a negative way, the overall cell stability score of each cell in each cluster. However, the reduction in CSS is not identical for all clusters. In Figure 41, it is clear that cluster 2, completely made of NK cells, is the most stable cluster to the perturbations induced by increasing the number of removed cells. On the other side, cluster 4, mainly made by stem cells (92 cells), together with few B-cells (2 cells) and Monocytes (7 cells) is the least stable. Sorting by increasing CSS the cells in cluster 4, Figure 41D, all B-cells and Monocytes are found within the first 15 most unstable cells. This observation suggests that studying CSS, increasing the fraction of cells removed at each permutation, could highlight the presence of cells located at the boundaries of different clusters. In this specific example, clusters 1, which is mainly composed of B-cells and few stem cells (2 cells), and cluster 3, which is fully made of Monocytes, could represent the exchange clusters for the most unstable cells detected in cluster 4.

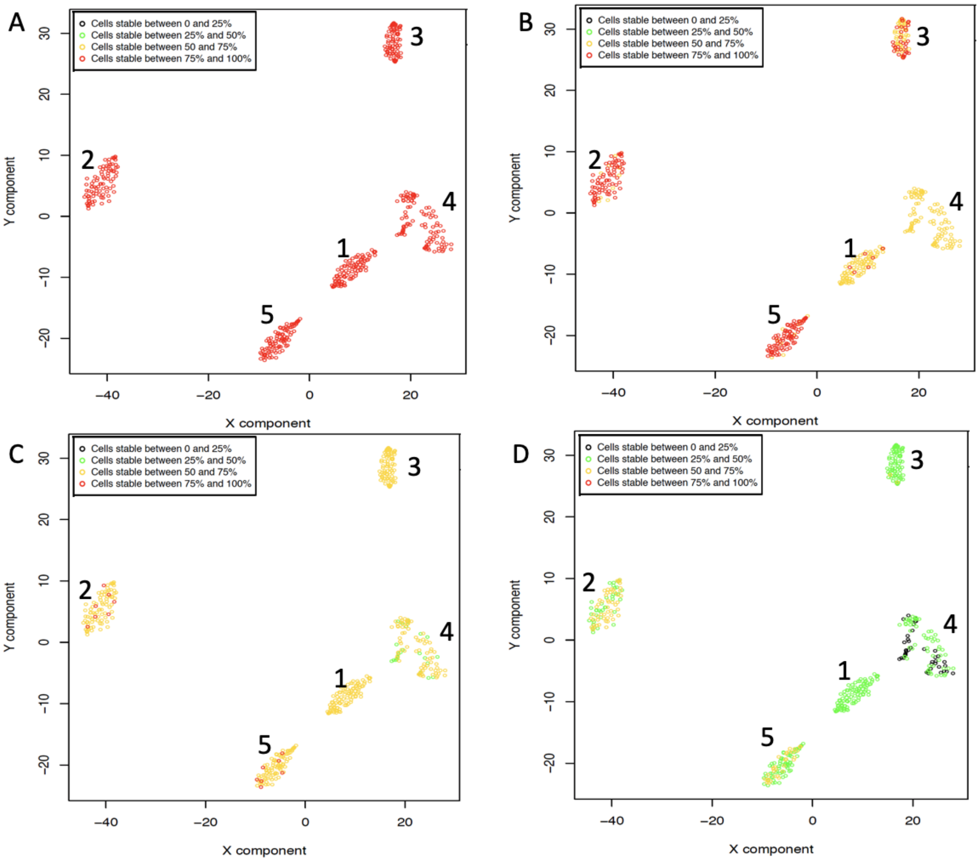

Figure 41: Cell stability score for SIMLR analysis with k=5 clusters removing progressively 10 (A), 20 (B), 30 (C) and 50 (D) percent of the cells at each permutation.

We investigated clusters composition of two sets of cells described in section 4.2: SetA and SetC. SetA is made of cell types with different biological characteristics, i.e. (B) B-cells, (M) Monocytes, (S) Stem cells, (NK) Natural Killer cells, (N) Naive T-cells. SetC contains Monocytes, Natural Killer cells and with T-cells subpopulations, i.e. (C) Cytotoxic T-cells, (H) T-helper cells and Naive T-cells, Figure 42. CSS provides indications on clusters cells heterogeneity. In Figure 42A and C, clusters characterized by high cell stability score are mainly constituted by one cell type. On the other hand, as CSS decrease, Figure 42B and D, clusters become characterized by an increasing heterogeneity in the cell types composition. E.g. In Figure 42B and D, cluster 5 (violet) has a CSS between 75% to 100% and it is made only by monocytes, cluster 2 (light green), which has a CSS between 50% and 75%, has a 11% contamination of T-helper cells. Cluster 3 (green), which has a CSS between 25 to 50%, is contaminated by 12% Cytotoxic T-cells and 4% T-helper cells. Finally, clusters 1 and 4, characterized by CSS between 0% to 25%, are very heterogeneous incorporating 4 out of 5 cell types present in this dataset.

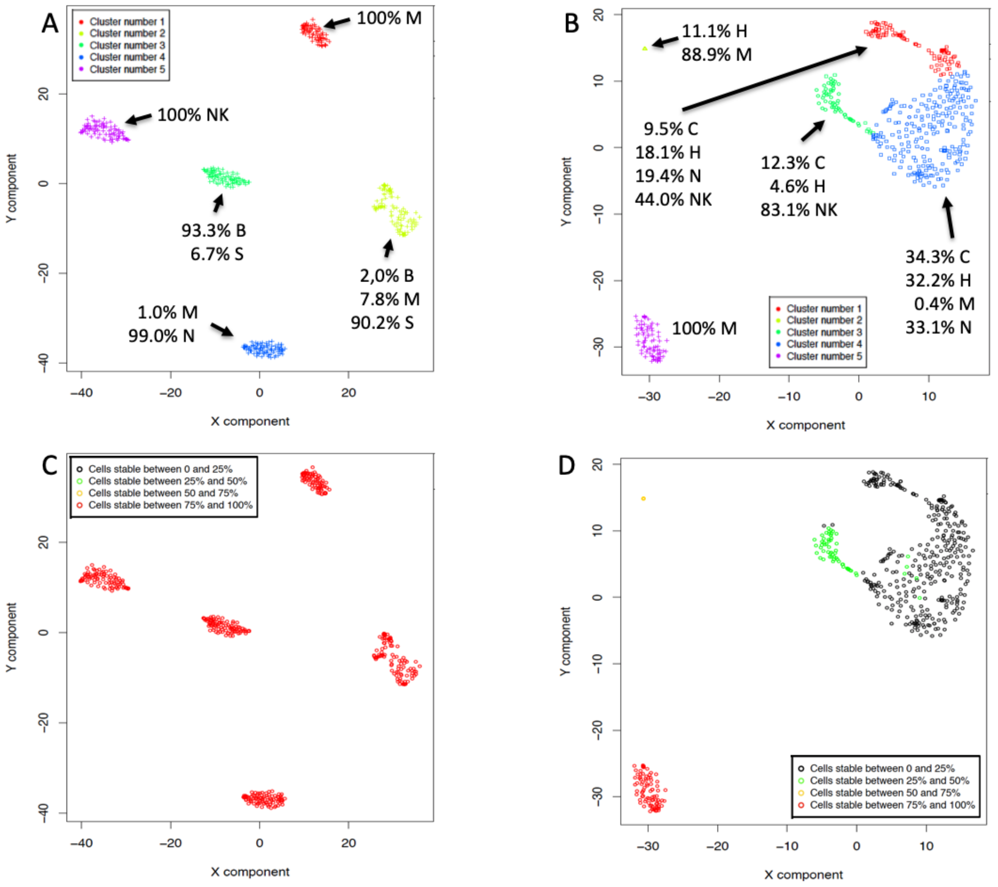

Figure 42: Investigating cell stability score relation with clusters cells heterogeneity. SIMLR analysis was performed on both datasets using 160 permutations and k=5. A) Set A, Section 4.2, SIMLR clusters structure, B) Set C, Section 4.2, SIMLR clusters structure. C) Set A CSS plot, D) Set C, CSS plot.

Section 5.2 Visualizing the cell clusters relocation during bootstraps.

The function **permutationMovie** allows the generation of a video showing the relocation of cells at each bootstrap. In this example we use the results shown in Figure 40B, where circles show the clusters where all cells have a cell stability between 50 to 75%.

- *Parameters* (only those without default; for the full list of parameters please refer to the function help) (Figure 43):
  - *scratch.folder*, path of the scratch folder
  - *file (GUI Matrix count)*, path of the file, with file name and extension included
  - *separator*, separator used in count file, e.g. '\t', ','
  - *framePP*, number of frames for each permutation
  - *permutationNumber,* number of random permutations, must be less or the same value of the total permutations dome in simlrBootstrap

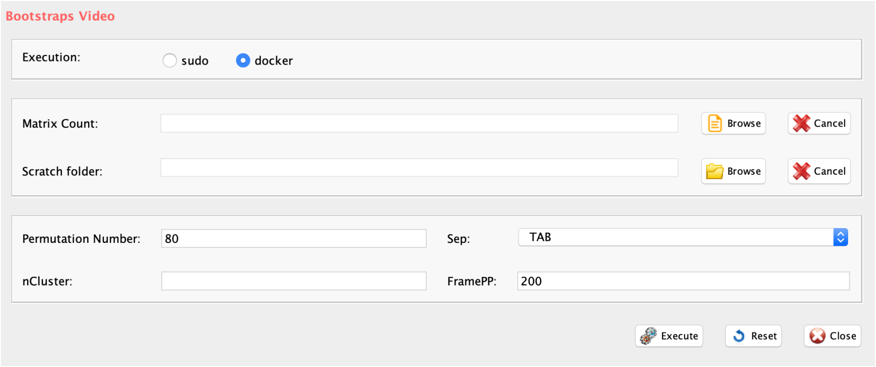

Figure 43: GUI: Permutation effect video panel. Clusters are identified by different colors

```
system("wget http://130.192.119.59/public/test_permutationvideo.zip")
unzip("test_permutationvideo.zip")
setwd("test_permutationvideo")
library("rCASC")
bootstrapsVideo(group="docker", scratch.folder="/data/scratch",
                file=paste(getwd(), "annotated_bmsnkn_5x100cells.txt", sep="/"),
                nCluster=6, separator="\t", framePP=200, permutationNumber=80)
```

The video was generated in 8 minutes on SeqBox hardware. The video is saved in the cluster folder used for the analysis and it has the name **outputname.mp4**. The video generated with the above script is accessible *here*.

In Figure 44A is shown a screenshot of the output of **bootstrapsVideo**. The output of this function provides extra information with respect to the standard output of the **simlrBootstrap** and **tsneBootstrap**, since the video provides an overview of the area (colored circles) in which the cells of a specific cluster are localized as consequence of the bootstraps. This visualization provides a better description of the maximum size of the clusters and simplify the identification of clusters that are more near to each other, because of clusters structure, Figure 44A. In this specific example, is notable that, even if the yellow cluster has a high cell stability score, Figure 44B, its size is much greater of that observable for all the other clusters, because few cells with lower cell stability score (50-75%, triangle) are located very near to the blue cluster, arrow in Figure 44B.

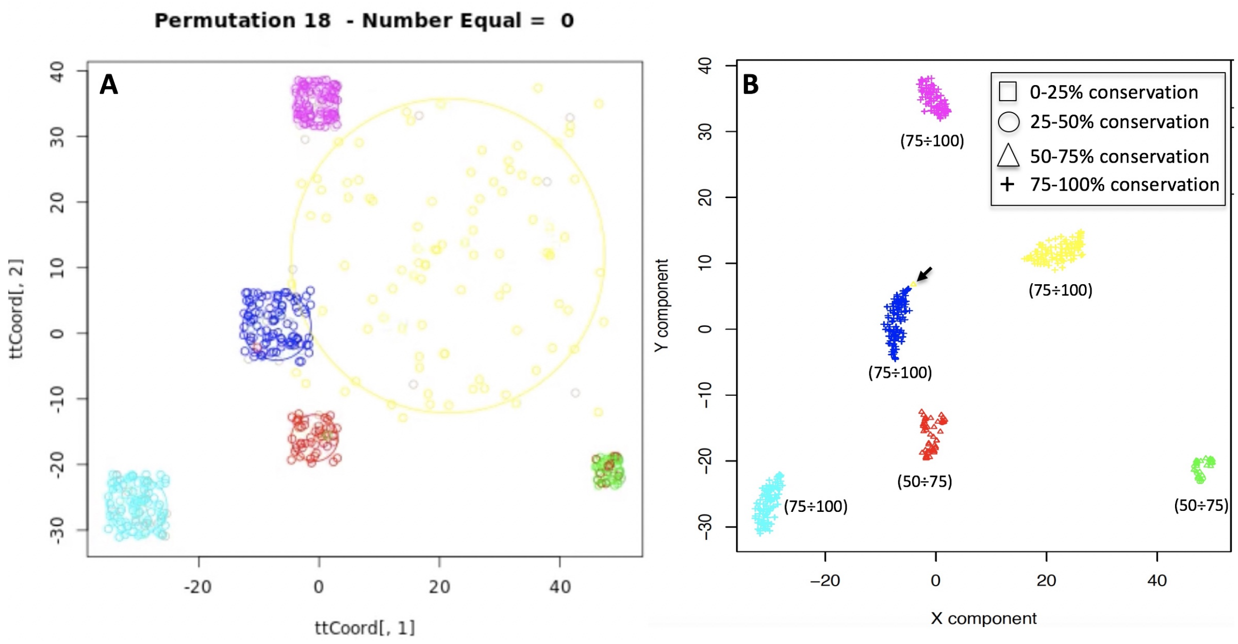

Figure 44: Comparing the output of bootstrapsVideo with respect to the one produced by simlrBootstrap. A) Output of bootstrapsVideo. B) simlrBootstrap output.

Section 5.3 Estimating cluster stability.

Hennig published an elegant paper entitled Cluster-wise assessment of cluster stability. The *R package fpc* is associated to the paper. In this package, Jaccard index is used to evaluate the similarity between clusters. The calculated score (cluster stability measurement, CSM) is intended to provide an overall quality score for each of the clusters in toto. In particular, the clusterboot function allows to evaluate the cluster stability using a personalized clustering function. We have implemented this function to estimate CSM of the clusters generated with SIMLR, Seurat and tSne.

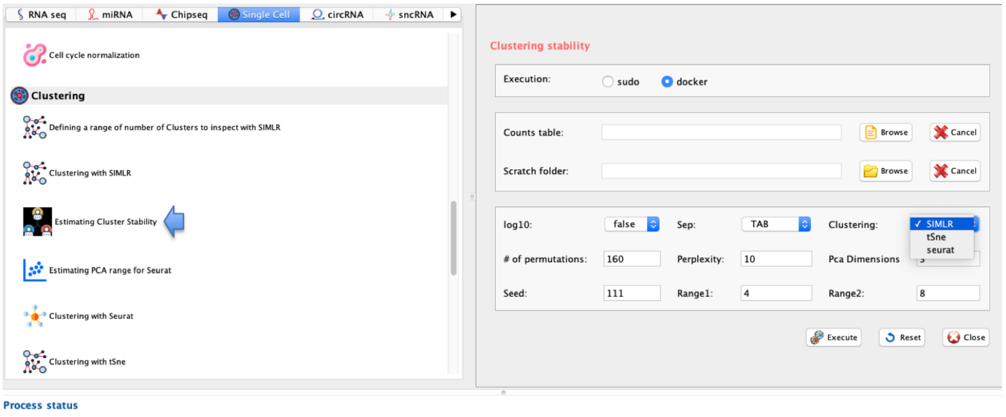

Figure 45: Estimating cluster stability.

- *Parameters* (only those without default; for the full list of parameters please refer to the function help):
  - *group*, a character string. Two options: sudo or docker, depending to which group the user belongs
  - *scratch.folder*, a character string indicating the path of the scratch folder
  - *file*, a character string indicating the path of the file, with file name and extension included
  - *nPerm*, number of permutations to perform the pValue to evaluate clustering
  - *range1*, first number of cluster for k means algorithm
  - *range2*, last number of cluster for k means algorithm
  - *separator*, separator used in count file, e.g. '\t', ','
  - *logTen*, 1 if the count matrix is already in log10, 0 otherwise
  - *clustering*, clustering method to use : "SIMLR" , "tSne", "griph"
  - *perplexity*, Number of close neighbors for each point. This parameter is specific for tSne. Default value is 10.Setting this parameter when use a clustering method different by tSne will be ignored.
  - *pcaDimensions*, dimensions to use for pca reduction for Seurat
  - *seed*, important value to reproduce the same results with same input

```
#SetA
clusterStability(group="docker",scratch.folder="/data/scratch/",
                 file=paste(getwd(), "bmsnkn_5x100cells.txt", sep="/"),
                 nPerm=160, range1=5, range2=5, separator="\t",
                 logTen=0, cluster="SIMLR", pcaDimensions=3)
#SetC
clusterStability(group="docker",scratch.folder="/data/scratch/",
                 file=paste(getwd(), "CNCHnkn_5x100cells.txt", sep="/"),
                 nPerm=160, range1=5, range2=5, separator="\t",
                 logTen=0, cluster="SIMLR", pcaDimensions=3)
```

The output of **clusterStability** are two plots, Figure 46.

Cluster stability measurement (CSM), Figure 46, which provides an overview of the stability of the cluster in toto, are in agreement with the CSS for SetA and SetC, Figure 42.

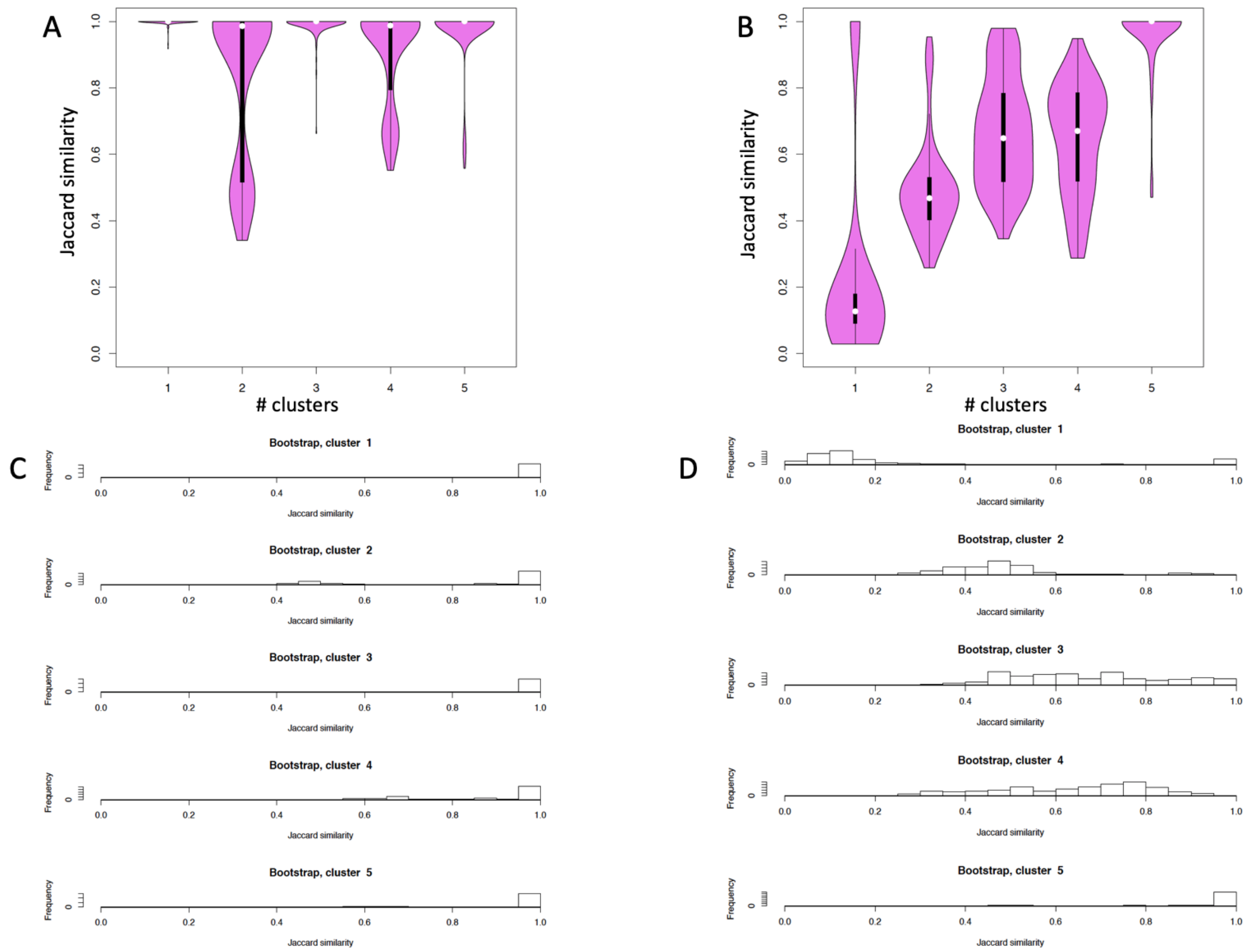

Figure 46: Estimating cluster stability output for SetA and SetC: A) Violin plot of Jaccard similarity value for SetA clusters. B) Violin plot of Jaccard similarity value for SetC clusters. C) Jaccard frequency distributions over each of SetA clusters. D) Jaccard frequency distributions over each of SetC clusters.

Section 6 Clustering (autonomously detecting the number of clusters required for data partitioning): *Seurat*, *griph* and *scanpy* graph-based clustering.

Seurat, *griph* and *scanpy* use similar clustering approaches. However, time required for clustering, in the rCASC framework, is quite different as the number of cell increases, see (Fig. 7 in main manuscript).

Section 6.1: *Seurat*

Recently *Freytag et al.* and *Do et al.* described, in their independent comparison papers, that *Seurat* delivered the overall best performance in cells clustering. Thus, we decided to include in our workflow also this clustering tool. Seurat clusters cells based on their PCA scores, with each PC essentially representing

a 'metagene' that combines information across a correlated gene set. The bootstrap approach described in **section 5.1** is also applied to Seurat clustering to assign cell stability score to the clustered cells. The first step of the analysis is the identification of the how many PCs have to be to included.

seuratPCAEval function allows the exploration of PCs to determine relevant sources of heterogeneity and to define the range of PCs to be used.

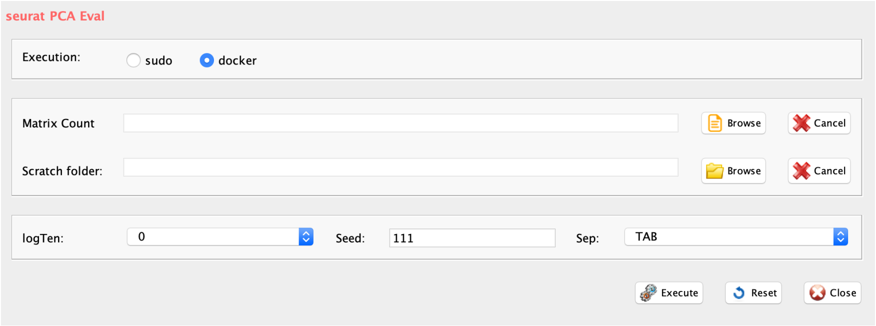

Figure 47: GUI: Estimating PCA range for Seurat panel

- *Parameters* (only those without default; for the full list of parameters please refer to the function help) (Figure 47):
  - *scratch.folder*, the path of the scratch folder
  - *file (GUI Matrix count)*, the path of the file, with file name and extension included
  - *separator*, separator used in count file, e.g. '\t', ','
  - *logTen*, 1 if the count matrix is already in log10, 0 otherwise
  - *seed*, important value to reproduce the same results with same input, default is 111

```
#N.B. seuratPCAEval requires in input raw counts or log10 transformed raw counts
#Before clustering data are normalized as suggested from the Seurat workflow
#using Seurat  NormalizeData function, with LogNormalize as normalization.method
#and 10000 as scale.factor parameters

system("wget http://130.192.119.59/public/section4.1_examples.zip")
unzip("section4.1_examples.zip")
setwd("section4.1_examples")
library(rCASC)
#defining the PC threshold for clustering.
seuratPCAEval(group="docker",scratch.folder="/data/scratch/",
              file=paste(getwd(), "bmsnkn_5x100cells.txt", sep="/"),
              separator="\t", logTen = 0, seed = 111)
```

The function generates a **Result** folder in which is present a folder with the same name of the dataset under analysis, in this specific case "bmsnkn_5x100cells". In the latter folder is present a PCA plot (PCE_bowPlot.pdf, Figure 48A), which allows the identification of the PCs to use, defining a cutoff where there is a clear elbow in the graph. In this example, it looks like the elbow would fall around PC 6.

seuratBootstrap function executes the seurat clustering (data reduction method PCA) and estimates cell

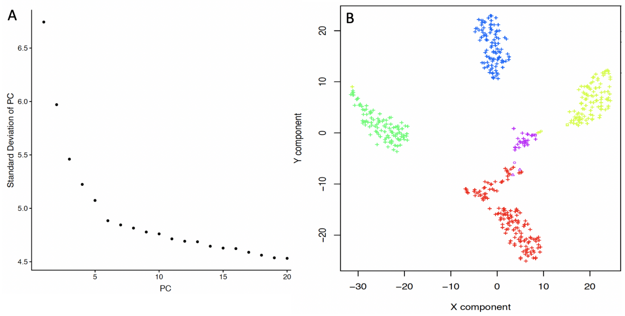

Figure 48: Seurat clustering: A) Seurat dataset PC component. B) Clustering results. Clusters are identified by different colors and CSS is given by different symbols: square 0-25, circle 25-50, triangle 50-75, cross 75-100

stability score.

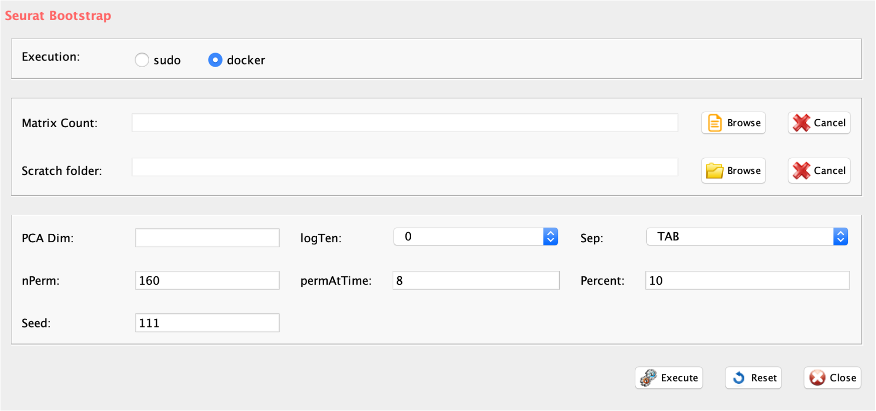

Figure 49: GUI: Seurat clustering panel

- *Parameters* (only those without default; for the full list of parameters please refer to the function help) (Figure 49):
  - *scratch.folder*, a character string indicating the path of the scratch folder
  - *file (GUI Matrix count)*, a character string indicating the path of the file, with file name and extension included
  - *nPerm*, number of permutations to be executed
  - *permAtTime*, number of permutations computed in parallel
  - *percent*, percentage of randomly selected cells removed in each permutation
  - *separator*, separator used in count file, e.g. '\t', ','
  - *logTen*, 1 if the count matrix is already in log10, 0 otherwise
  - *pcaDimensions*, 0 for automatic selection of PC elbow.
  - *seed*, important value to reproduce the same results with same input

```
#N.B. seuratBootstrap requires in input raw counts or log10 transformed raw counts

seuratBootstrap(group="docker",scratch.folder="/data/scratch/",
```

```
            file=paste(getwd(), "bmsnkn_5x100cells.txt", sep="/"),
            nPerm=160, permAtTime=8, percent=10, separator="\t",
            logTen=0, pcaDimensions=6, seed=111)
```

The function generates a **Result** folder in which is present a folder with the same name of the dataset under analysis, in this specific case "bmsnkn_5x100cells.txt". In the latter folder is present a folder with the number of clusters detected by Seurat, in this specific case **5**. In **5** folder there is the file with extension **_Stability_Plot.pdf**, which is the clustering picture with cells labeled with their stability score, Figure 48B). The file with the extension **_clusterP.txt**, containing for each permutation the location of each cell in the clusters. The file with the extension **_killedCell.txt**, where are saved the cells removed at each permutation. The file with the extension **_score.txt**, containing the scores assigned at each permutation to each cell. The file with extension **_scoreSum.txt** containing the Cell stability score for each cell. The file with extension **_clustering.output.txt**, summarizing all information required to generate the **_Stability_Plot.pdf** plot.

Section 6.2: *griph*

Griph was used as tool for clustering in Serra et al. 2019. It runs faster than Seurat as the number of cells increases.

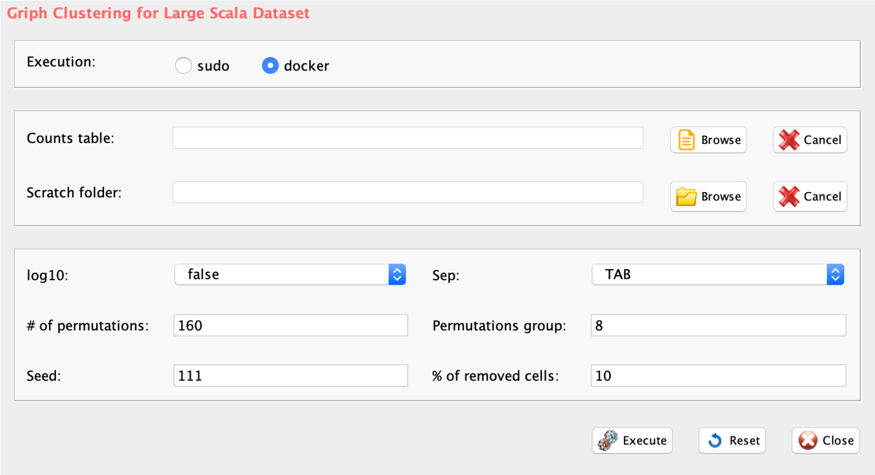

Figure 50: GUI: Griph clustering panel

- *Parameters* (only those without default; for the full list of parameters please refer to the function help) (Figure 50):
  - *group*, a character string. Two options: sudo or docker, depending to which group the user belongs
  - *scratch.folder*, a character string indicating the path of the scratch folder
  - *file*, a character string indicating the path of the file, with file name and extension included
  - *nPerm*, number of permutations to be executed
  - *permAtTime*, number of permutations computed in parallel

– *percent*, percentage of randomly selected cells removed in each permutation
– *separator*, separator used in count file, e.g. ‘\t’,‘,’
– *logTen*, 1 if the count matrix is already in log10, 0 otherwise
– *perplexity*, perplexity value for tsne projection
– *seed*, important value to reproduce the same results with same input

```
#N.B. griphBootstrap requires in input raw counts or log10 transformed raw counts

griphBootstrap(group="docker",scratch.folder="/data/scratch/",
               file=paste(getwd(), "bmsnkn_5x100cells.txt", sep="/"),
               nPerm=160, permAtTime=8, percent=10, separator="\t",logTen=0,
               seed=111)
```

Section 6.3: *scanpy*

Scanpy is a scalable toolkit for analyzing large single-cell gene expression data Wolf et al. 2019. It runs faster than Seurat and griph as the number of cells increases.

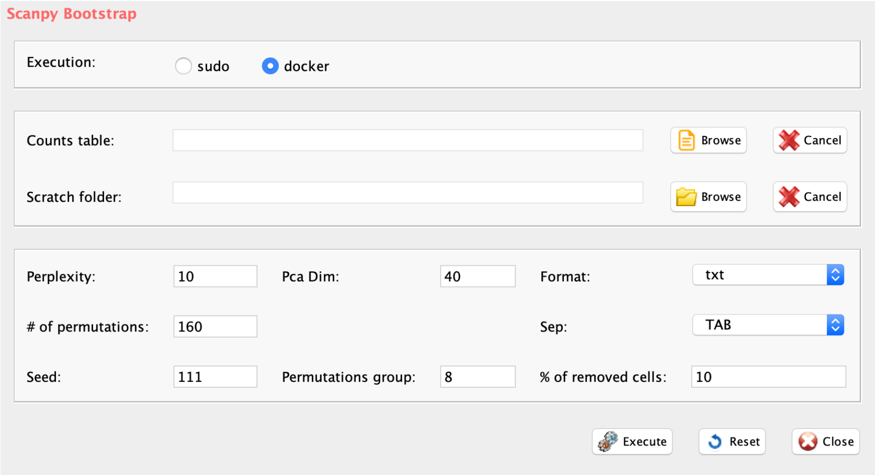

Figure 51: GUI: Griph clustering panel

- *Parameters* (only those without default; for the full list of parameters please refer to the function help) (Figure 51):
  – *group*, a character string. Two options: sudo or docker, depending to which group the user belongs
  – *scratch.folder*, a character string indicating the path of the scratch folder
  – *file*, a character string indicating the path of the file, with file name and extension included
  – *nPerm*, number of permutations to be executed
  – *permAtTime*, number of permutations computed in parallel
  – *percent*, percentage of randomly selected cells removed in each permutation
  – *separator*, separator used in count file, e.g. ‘\t’,‘,’
  – *perplexity*, perplexity value for tsne projection

– *pca_number*, PCA threshold selected using seuratPCAEval function.
– *seed*, important value to reproduce the same results with same input
– *format*, output file format csv or txt. Mandatory because scanpy only accepts sparse matrices

```
#N.B. scanpy requires in input raw counts in sparse format

scanpyBootstrap(group="docker",scratch.folder="/data/scratch/",
                file=paste(getwd(), "matrix.mtx", sep="/"),
                nPerm=160, permAtTime=8, percent=10, separator="\t",
                perplexity=10, pca_number=6, seed=111, format="txt")
```

Section 6.4 Comparing SIMLR, tSne, Seurat, griph and scanpy clustering.

In Wang [*2017*] article it is demonstrated that SIMLR outperforms other data reduction methods. *Freytag et al.* and *Do et al.* described that *Seurat* delivers the overall best performance in cells clustering. Here, we run a comparison between SIMLR, tSne, Seurat, griph and scanpy within the rCASC bootstrap framework to observe how cells stability score is affected by the three clustering methods. The bootstrap approach described in section 5.1 is also applied to tSne, Seurat, griph and scanpy clustering to assign cell stability score to the clustered cells. In rCASC tSne is implemented using the *Rtsne* package. Rtsne performs a data reduction on which k-mean clustering is applied. tSne is embedded in **tsneBootstrap** function within the rCASC bootstrap framework.

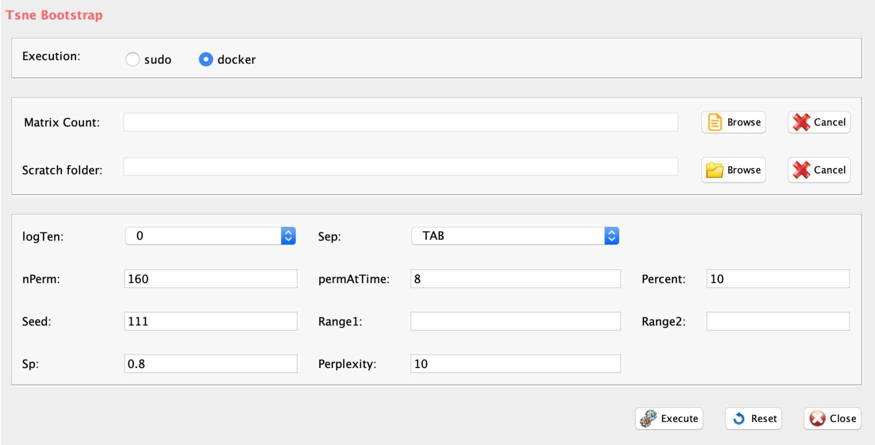

Figure 52: GUI: tSne clustering panel

- *Parameters* (only those without default; for the full list of parameters please refer to the function help) (Figure 52):
  – *scratch.folder*, the path of the scratch folder
  – *file (GUI Matrix count)*, the path of the file, with file name and extension included
  – *nPerm*, number of permutations to be executed
  – *permAtTime*, number of permutations computed in parallel
  – *percent*, percentage of randomly selected cells removed in each permutation

- *range1*, beginning of the range of clusters to be investigated
- *range2*, end of the range of clusters to be investigated
- *separator*, separator used in count file, e.g. '\t', ','
- *logTen*, 1 if the count matrix is already in log10, 0 otherwise
- *seed*, important value to reproduce the same results with same input, default is 111
- *sp*, minimum number of percentage of cells that has to be in common in a cluster, between two permutations, default 0.8
- *clusterPermErr*, probability error in depicting the number of clusters in each permutation, default = 0.05
- *perplexity*, number of close neighbors for each point. This parameter is specific for tSne. Default value is 10. the performance of t-SNE is fairly robust under different settings of the perplexity. The most appropriate value depends on the density of your data. A larger/denser dataset requires a larger perplexity. Typical values for the perplexity range between 5 and 50.

```
system("wget http://130.192.119.59/public/section4.1_examples.zip")
unzip("section4.1_examples.zip")
setwd("section4.1_examples")

system("wget ftp://ftp.ensembl.org/pub/release-94/gtf/homo_sapiens/Homo_sapiens.GRCh38.94.gtf.gz")
system("gzip -d Homo_sapiens.GRCh38.94.gtf.gz")
system("mv Homo_sapiens.GRCh38.94.gtf genome.gtf")

library(rCASC)
#annotating data setA

scannobyGtf(group="docker", file=paste(getwd(),"bmsnkn_5x100cells.txt",sep="/"),
                    gtf.name="genome.gtf", biotype="protein_coding",
                    mt=TRUE, ribo.proteins=TRUE,umiXgene=3, riboStart.percentage=0,
                    riboEnd.percentage=100, mitoStart.percentage=0, mitoEnd.percentage=100)
#N.B. tsneBootstrap requires in input raw counts or log10 transformed raw counts
#running tSne analysis using the range of clusters suggested by clusterNgriph in session 4.2
tsneBootstrap(group="docker",scratch.folder="/data/scratch/",
              file=paste(getwd(), "annotated_bmsnkn_5x100cells.txt", sep="/"),
              nPerm=160, permAtTime=8, percent=10, range1=4, range2=6, separator="\t",
              logTen=0, seed=111, sp=0.8, perplexity=10)
# required time 159 mins

#running SIMLR analysis using the range of clusters suggested by clusterNgriph in session 4.2
simlrBootstrap(group="docker",scratch.folder="/data/scratch/",
               file=paste(getwd(), "annotated_bmsnkn_5x100cells.txt", sep="/"),
               nPerm=160, permAtTime=8, percent=10, range1=4, range2=6,
               separator="\t",logTen=0, seed=111)
# required time 168 mins

#running Seurat clustering: defining the PC threshold for clustering.
seuratPCAEval(group="docker",scratch.folder="/data/scratch/",
```

```
            file=paste(getwd(), "annotated_bmsnkn_5x100cells.txt", sep="/"),
            separator="\t", logTen = 0, seed = 111)
#running Seurat clustering with bootstrap: threshold was detected at PC 6, see Section 6.
seuratBootstrap(group="docker",scratch.folder="/data/scratch/",file=paste(getwd(), "annotated_bmsnkn_5x100cells.txt", sep="/"),
                nPerm=160, permAtTime=8, percent=10, separator="\t",logTen=0, pcaDimensions=6, seed=111)
# required time 194 mins

#running griph clustering

griphBootstrap(group="docker",scratch.folder="/data/scratch/",
                file=paste(getwd(), "annotated_bmsnkn_5x100cells.txt", sep="/"),
                nPerm=160, permAtTime=8, percent=10, separator="\t",logTen=0,
                seed=111)

# required time 61 mins

#running scanpy clustering
scanpyBootstrap(group="docker",scratch.folder="/data/scratch/",
                file=paste(getwd(), "matrix.mtx", sep="/"),
                nPerm=160, permAtTime=8, percent=10, separator="\t",
                perplexity=10, pca_number=6, seed=111, format="txt")

#please note that an error like this might appear but is not affecting the overall results:
#Error response from daemon: can not get logs from container which is dead or marked for removal


# required time 9 mins
```

We run the analysis on the SetA described in Section 4.1. SIMLR Seurat and griph detect the 5 clusters, i.e. 5 cell types, and all clusters show an high CSC (Figure ??A-C). However, tSne detects the 5 clusters, but they are less stable in terms of CSS (Figure ??E). For scanpy we tested various combination of perplexity and PCs number but we failed to find a condition detecting the 5 clusters and clusters stability is very poor. From the point of view of the computation time, the above mentioned analysis took 159 mins for tSne, 168 mins for SIMLR, 194 mins for Seurat, 61 mins for griph and only 9 minus for scanpy.

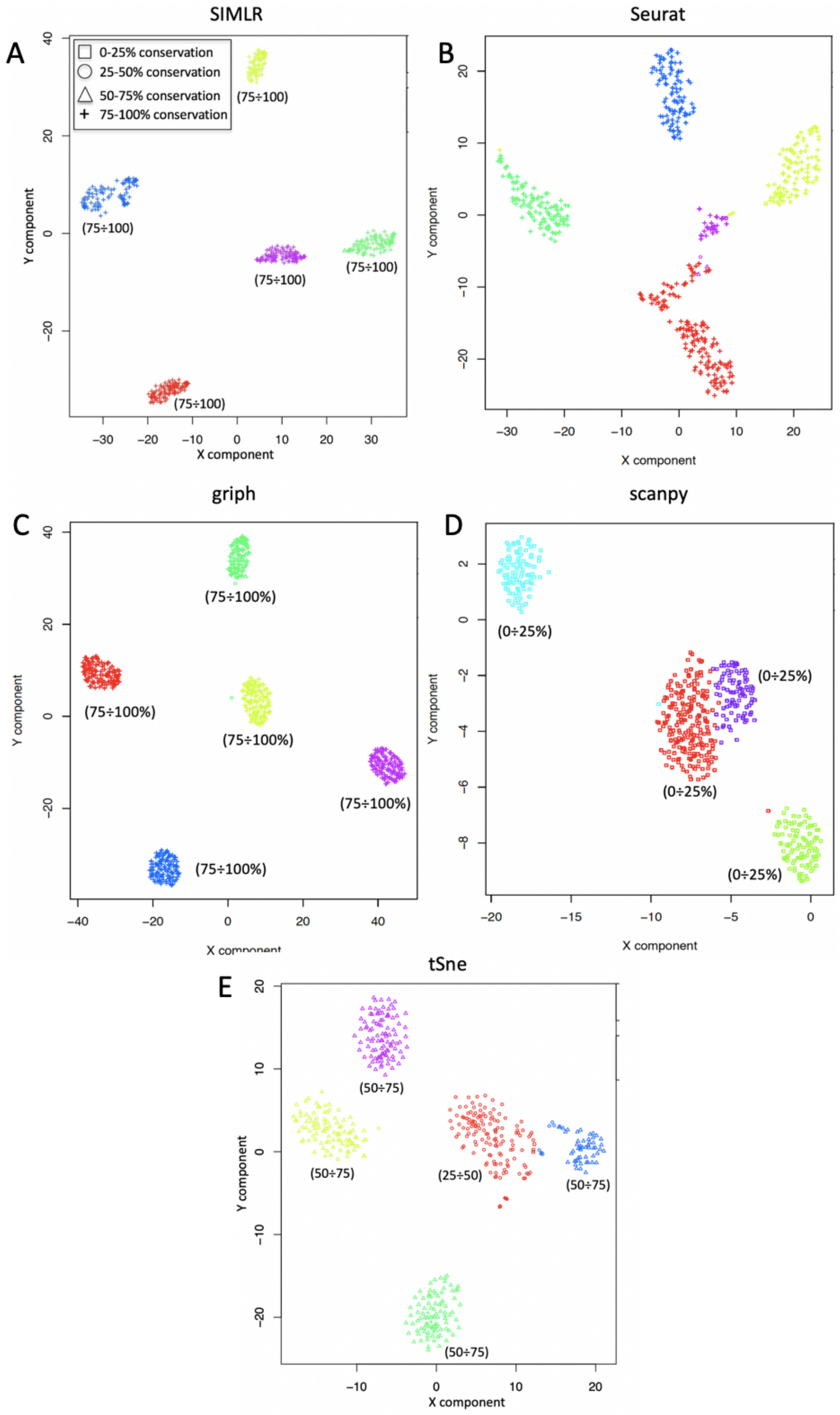

Figure 53: Comparing SIMLR and tSne within rCASC bootstrap framework. A) SetA clustered with SIMLR. B) SetA clustered with Seurat, C) SetA clustered with griph, D) SetA clustered with scanpy, E) SetA clustered with tSne. N.B. Colors do not define the same cluster in figure A and B.

Section 7 Detecting the genes playing the major role in clusters formation

Nowadays there are a lot of methods for the identification of differentially expressed genes between two populations of single-cell experiments [*Soneson et al.*]. However, the number of tools applicable to single-cells and allowing something similar to an ANOVA are very few. An ANOVA-like approach is described as applicable to single-cells in *edgeR Bioconductor package*. The edgeR ANOVA-like requires a comparison with respect to a reference set of cells. SIMLR and Seurat also provide ranking score for the genes, which are affecting mostly the clusters organization. SIMLR and Seurat gene ranking do not require a reference cluster for the analysis, thus providing wider applications with respect to ANOVA-like. The three methods are part of the rCASC framework.

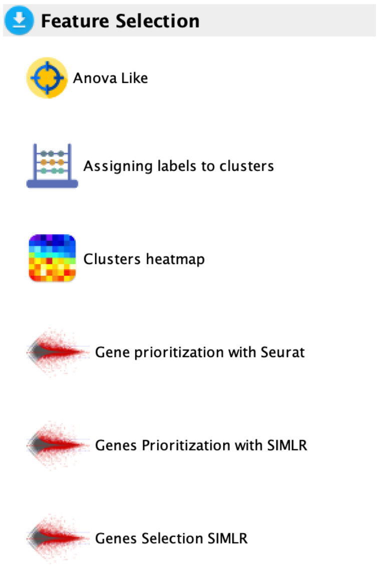

Figure 54: GUI: Feature selection panel

Section 7.1 A statistical approach to select genes affecting clusters formation: EdgeR ANOVA-like

The function **anovaLike** executes edgeR ANOVA-like with the settings required for single-cells analysis, for more information please refer to the edgeR vignette.

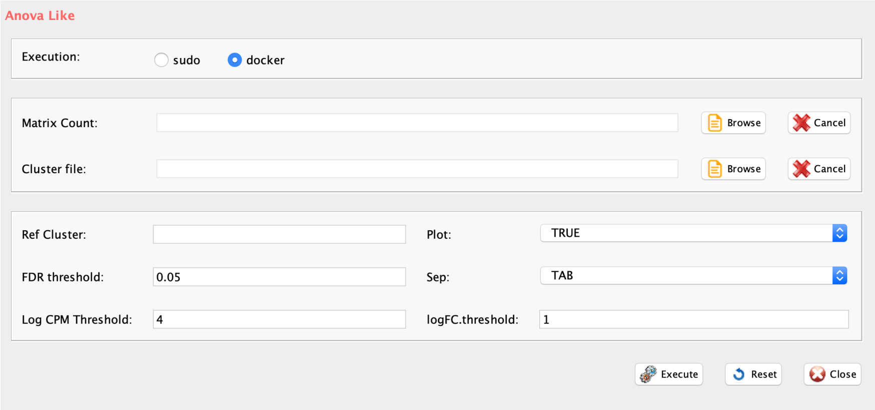

Figure 55: GUI: ANOVA-like feature selection panel

- *Parameters* (only those without default; for the full list of parameters please refer to the function help) (Figure 55):
  - *file (GUI Matrix count)*, a character string indicating the counts table file with the path of the file.
  - *sep*, separator used in count file, e.g. '\t', ','
  - *cluster.file*, a character string indicating the _clustering.output.txt file of interest, generated by bootstrapSimlar or bootStrapTsne. IMPORTANT this file must be located in the same folder where counts.table is placed
  - *ref.cluster*, a number indicating the cluster to be used a reference for anova-like comparison with the other clusters.
  - *logFC.threshold*, minimal logFC present in at least one of the comparisons with respect to reference covariate
  - *FDR.threshold*, minimal FDR present in at least one of the comparisons with respect to reference covariate
  - *logCPM.threshold*, minimal average abundance
  - *plot*, boolean, TRUE a plot of differentially expressed genes is generated

The input file is a counts table, which is modified by **anovaLike** adding the cluster number to cell name with an underscore. It is mandatory that no other underscores are located in the table. Then, counts table is reorganized to locate at the beginning of the table the set of cells belonging to the cluster used as reference group. The differentially expressed genes statistics for all genes are saved in the file with prefix **DE__**, followed by the counts table name. The filtered differentially expressed genes statistics, including only genes detected as differentially expressed in at least one of the clusters is saved with prefix **filtered__DE__** followed by the counts table name. **logFC__filtered__DE__** refers to the file containing only logFC extracted from **filtered__DE__** file. The count table is also saved reordered on the basis of cluster positions and has the extension **__reordered.txt**

```
#dataset derived from the analysis shown in section 8
system("wget http://130.192.119.59/public/annotated_setPace_10000_noC5_clustering.output.txt")
system("wget http://130.192.119.59/public/annotated_setPace_10000_noC5.txt.zip")
```

```r
unzip("annotated_setPace_10000_noC5.txt.zip")
#N.B. anovaLike requires in input raw counts
anovaLike(group="docker", data.folder=getwd(), counts.table="annotated_setPace_10000_noC5.txt",
        file.type="txt", cluster.file="annotated_setPace_10000_noC5_clustering.output.txt", ref.cluster=3,
        logFC.threshold=1, FDR.threshold=0.05, logCPM.threshold=4, plot=TRUE)

#the output of interest is logFC_filtered_DE_annotated_setPace_10000_noC5_reordered.txt
```

The function **clustersFeatures** selects the genes, which are more up-regulated in a specific cluster using **anovaLike** outputs.

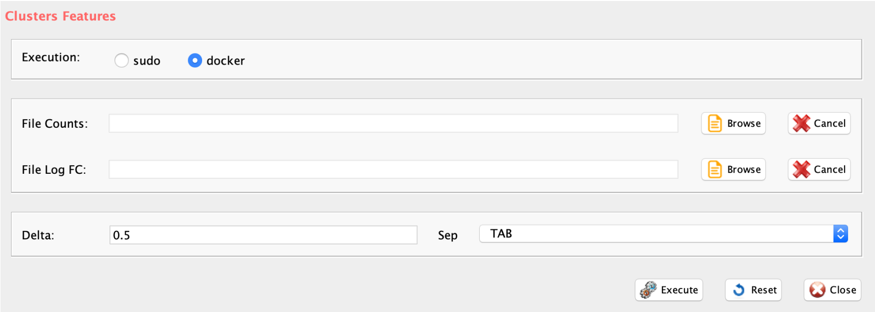

Figure 56: GUI: ANOVA like prioritization features panel

- *Parameters* (only those without default; for the full list of parameters please refer to the function help) (Figure 56):
  - *fileLogFC*, a character string indicating the absolute path to logFC_filtered_DE_ file generated by anovaLike function
  - *fileCounts*, a character string indicating the absolute path to reordered counts table file generated by anovaLike function
  - *delta*, the minimal distance between the max value of FC for a gene in a cluster of interest and the nearest other max FC in any of the other clusters. This value defines the minimal distance with respect to the same gene in another cluster to identify it as a cluster specific gene.
  - *sep*, separator used in count file, e.g. '\t', ','

This function focus only on upregulated genes because the ANOVA-like procedure compares all clusters with respect to a reference cluster. Thus, up-regulated genes are genes specific of the cluster under analysis as those down-regulated are characteristics of the reference cluster. The delta parameter describes the minimal distance existing, for a specific gene average logFC in a specific cluster, with respect to all the other clusters under analysis. The outputs of the function are four tab delimited files:

- the file with prefix **onlyUP__**, followed by the counts table name, i.e. the count table only containing the selected genes,
- the file with prefix**onlyUP__**, followed by **logFC__filtered__DE__**, the table containing logFC only for the selected genes,

- the file **onlyUP__clusters__specific__genes.txt**, which contains the list of specific genes associated with the corresponding cluster.
- the file **onlyUP__clusters__specific__genesSYMBOLs.txt**, which contains the list of specific genes SYMBOLS from onlyUP_clusters_specific_genes.txt. This file is used by function **hfc** to generate features specific heatmap.

```
#dataset derived from the exemplary analysis shown in section 8
system("wget http://130.192.119.59/public/clusters.features.zip")
unzip("clusters.features.zip")
setwd("clusters.features")

clustersFeatures(group="docker", data.folder=getwd(),
         logFC.table="logFC_filtered_DE_annotated_setPace_10000_noC5_reordered.txt",
         counts.table="annotated_setPace_10000_noC5_reordered.txt", delta=0.5)
```

Section 7.1.1 Visualizing cluster specific genes by heatmap.

The gene-features extracted by **clustersFeatures** cannot be used again in **simlrBootstrap**, if the overall number of genes is smaller of the cell number, for more information see SIMLR publication. This limitation does not apply to tsneBootstrap.

The function **hfc** allows to create a heatmap for the results generated from **simrBootstrap/tsneBootstrap** and the output of **clustersFeatures**.

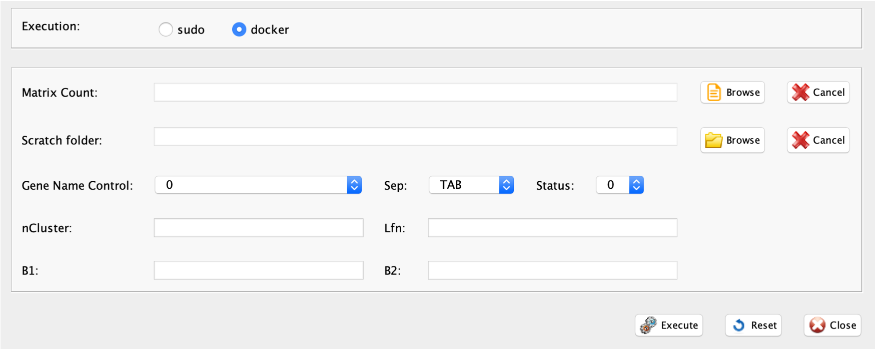

Figure 57: GUI: Heatmap for prioritized genes panel

- *Parameters* (only those without default; for the full list of parameters please refer to the function help) (Figure 57):
  - *scratch.folder*, the path of the scratch folder
  - *file (GUI Matrix count)*, the path of the file, with file name and extension included
  - *nCluster*, n of the clusters on which to run the analysis. The function expect the presence of Result folder generated by **simrBootstrap/tsneBootstrap**
  - *separator*, separator used in count file, e.g. '\t', ','

- *lfn*, name of the list of genes WITHOUT extension, if **clustersFeatures** function was used on anovaLike output the name of the file is "onlyUP__clusters__specific__geneSYMBOLs"
- *geneNameControl*, 0 if the matrix has gene name without ENSEMBL geneID. 1 if the gene names is formatted like this : ENSMUSG00000000001:Gnai3.
- *status*, 0 if is raw count, 1 otherwise
- *b1*, the lower range of signal in the heatmap,for negative value write "/-5". To ask the function to do automatic value assignment set 0
- *b2*, the upper range of signal in the heatmap,for negative value write "/-2". To ask the function to do automatic value assignment set 0

```
system("wget http://130.192.119.59/public/hfc.zip")
unzip("clusters.features.zip")
setwd("clusters.features")

hfc(group="docker",scratch.folder="/data/scratch",
       file=paste(getwd(),"annotated_setPace_10000_noC5.txt", sep="/"),
       nCluster=5, separator="\t", lfn="onlyUP_clusters_specific_geneSYMBOLs",
       geneNameControl=1, status=0, b1="/-1", b2="1")
```

The output is made of four pdf (Figure 58):

- *lfn-parameter*__hfc__log10.pdf counts in log10 format, e.g. onlyUP__clusters__specific__geneSYMBOLs__hfc__log10.pdf
- *lfn-parameter*__hfc__zscore.pdf Z score
- *lfn-parameter*__hfc__CSS.pdf cell cluster stability score
- *lfn-parameter*__hfc__all__genes.pdf

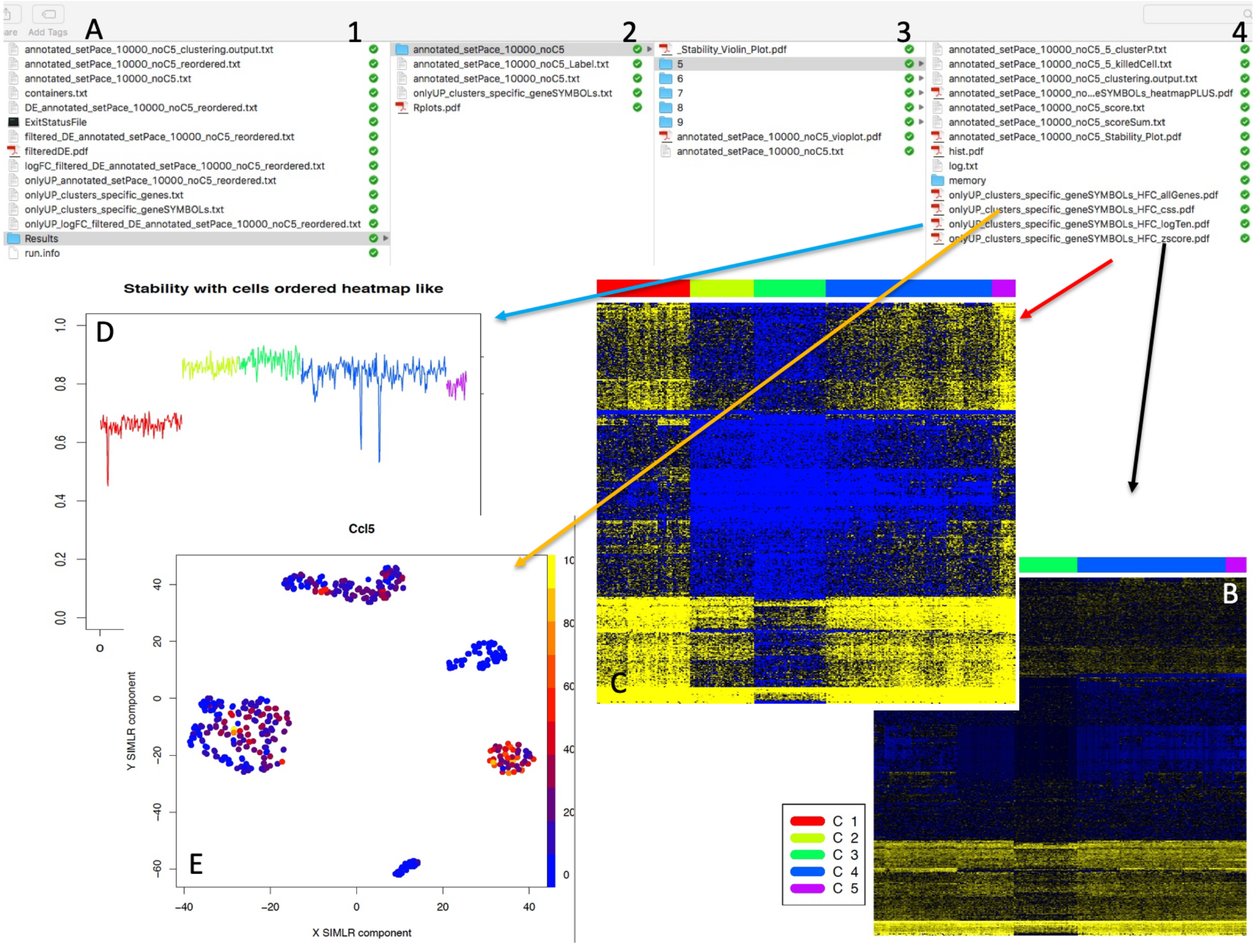

Figure 58: Output of hfc. A) Path of location of hfc function results. The output is located in cluster folder used for the analysis, B) Log10 counts heatmap, high number of counts is indicated by bright yellow color as instead low number of counts is indicated by bright blue color. On the top of the figure clusters are represented as bars of different colors, C) Z-score heatmap, high positive Z-score is indicated by bright yellow color as instead high negative Z-core is indicated by bright blue color, D) Cell Stability Score for each cell in each cluster, for more information see Section 5, CSS is represented with different colors lines on the basis of the belonging cluster, E) SIMLR clustering output for each gene within those used to generate the heatmap. Cells are colored on the basis of the amount of CPMs for the gene under analysis, blue color refers to low CPM as instead bright yellow indicates high CPM.

Section 7.2 A machine learning approach: SIMLR genes prioritization

SIMLR gene prioritization approach relies on the output similarity and does not depend on the downstream dimension reduction or clustering. The advantage of this step is that one can identify highly fluctuating genes that are consistent with the learned similarity in the multiple-kernel space.

The function **genesPrioritization** executes SIMLR gene prioritization within the rCASC bootstrap framework.

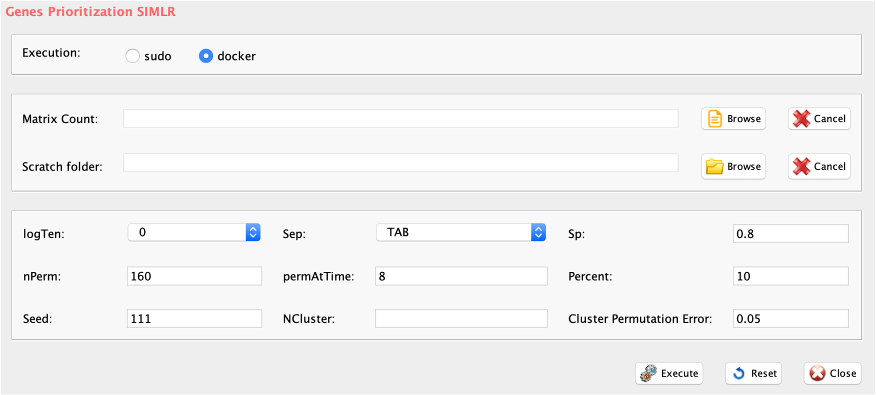

Figure 59: GUI: SIMLR genes prioritization panel

- *Parameters* (only those without default; for the full list of parameters please refer to the function help) (Figure 59):
  - *scratch.folder*, the path of the scratch folder
  - *file (GUI Matrix count)*, the path of the file, with file name and extension included
  - *nPerm*, number of permutations to be executed
  - *permAtTime*, number of permutations that can be computes in parallel
  - *percent*, percentage of randomly selected cells removed in each permutation
  - *nCluster*, the number of clusters, where to run prioritization
  - *separator*, separator used in count file, e.g. '\t', ','
  - *logTen*, 1 if the count matrix is already in log10, 0 otherwise
  - *seed*, important value to reproduce the same results with same input
  - *sp*, minimum number of percentage of cells that has to be in common between two permutation to be the same cluster, default 0.8.
  - *clusterPermErr*, error that can be done by each permutation in cluster number depicting, default=0.05

```
#dataset derived from the exemplary analysis shown in section 8
system("wget http://130.192.119.59/public/annotated_setPace_10000_noC5.txt.zip")
unzip("annotated_setPace_10000_noC5.txt.zip")
#N.B. genesPrioritization requires in input raw counts or log10 transformed raw counts
genesPrioritization(group="docker",scratch.folder="/data/scratch/",
                    file=paste(getwd(),"annotated_setPace_10000_noC5.txt", sep="/"),
                    nPerm=160, permAtTime=8, percent=10, nCluster=5, separator="\t",
                    logTen=0, seed=111, sp=0.8, clusterPermErr=0.05)
```

The output of the function are a pdf named **geneRank.pdf** (Figure 61), containing the overall distribution of -log10 expression mean with respect to the Delta confidence of a gene being important for clustering, and a tab delimited file with the extension **_pvalList.txt** which contains, for each bootstrap, the gene importance

score for clustering.

The function **genesSelection** allows to run a selection of the most interesting genes for clustering on the basis of two parameters:

- maxDeltaConfidence:
  - max value for Delta confidence for genes features selection. Default is 0.01
- minLogMean:
  - min value for Log mean for genes feature selection. Default is 0.05

The above values can be set in the **genesSelection** function on the basis of the output of geneRank.pdf (Figure 61).

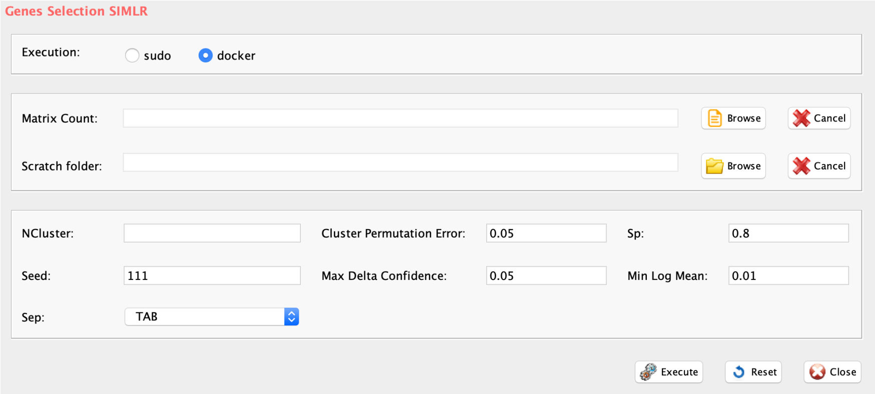

Figure 60: GUI: SIMLR genes selection panel

- *Parameters* (only those without default; for the full list of parameters please refer to the function help) (Figure 60):
  - *scratch.folder*, path of the scratch folder
  - *file (GUI Matrix count)*, the path of the counts file, with file name and extension included
  - *nCluster*, the number of clusters, where prioritization was run. In the working folder should be present the Result folder generated by **genesPrioritization** function.
  - *separator*, separator used in count file, e.g. '\t', ','
  - *seed*, important value to reproduce the same results with same input, default 111
  - *sp*, minimum number of percentage of cells that has to be in common in a cluster, between two permutations, default 0.8
  - *clusterPermErr*, probability error in depicting the number of clusters in each permutation, default = 0.05
  - *maxDeltaConfidence*, max value for Delta confidence for genes feature selection
  - *minLogMean*, min value for Log mean for genes feature selection

```
#dataset derived from the exemplary analysis shown in section 8
genesSelection(group="docker",scratch.folder="/data/scratch/",
               file=paste(getwd(), "annotated_setPace_10000_noC5.txt", sep="/"),
```

```
                nCluster=5, separator="\t", seed=111, sp=0.8, clusterPermErr=0.05,
                maxDeltaConfidence=0.01, minLogMean=20)

#mv annotated_setPace_10000_noC5_clusters_specific_geneSYMBOLs.txt in the main folder

hfc(group="docker",scratch.folder="/data/scratch",
       file=paste(getwd(),"annotated_setPace_10000_noC5.txt", sep="/"),
       nCluster=5, separator="\t",
       lfn="annotated_setPace_10000_noC5_clusters_specific_geneSYMBOLs",
       geneNameControl=1, status=0, b1="/-1", b2="1")
```

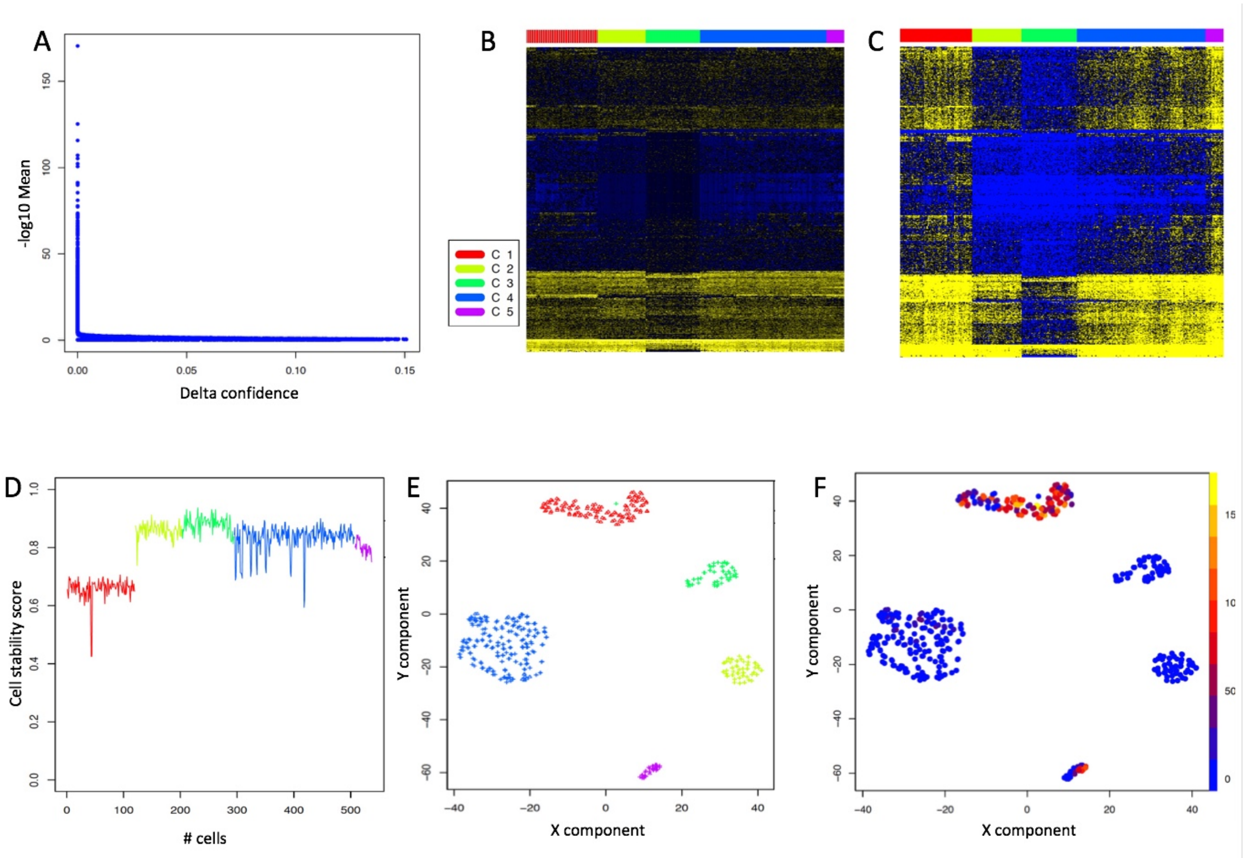

Figure 61: genePrioritization and genesSelection outputs. A) Prioritization plot, from this plot the maxDelta-Confidence (x axis) and the minLogMean (y axis), required by genesSelection can be extrapolated, i.e. in this case respectively 0.01 and 20. B) log10 counts heatmap from the output of genesSelection: 577 genes, high number of counts is indicated by bright yellow color as instead low number of counts is indicated by bright blue color. On the top of the figure clusters are represented as bars of different colors. C) Z-score heatmap, high positive Z-score is indicated by bright yellow color as instead high negative Z-core is indicated by bright blue color. D) Cell stability score. E) simlrBootstrap output. F) Single gene CPM expression in SIMLR clusters, Cells are colored on the basis of the amount of CPMs for the gene under analysis, blue color refers to low CPM as instead bright yellow indicates high CPM. The expression is available for each of the genes from genesSelection output

The output of **genesSelection** is a file with extension **_clusters_specific_geneSYMBOLs.txt**, which can be used as input for** hfc** function, see **Section 6.1.1**. The list of prioritized genes cannot not be associated to a specific cluster as in the case of the anovaLike, see **Section 6.1**. Thus, more information will be provided on the cluster specificity from the heatmap visualization (Figure **??**), see **hfc** function.

Section 7.3 Seurat genes prioritization

Seurat gene prioritization is performed by **seuratPrior** function.

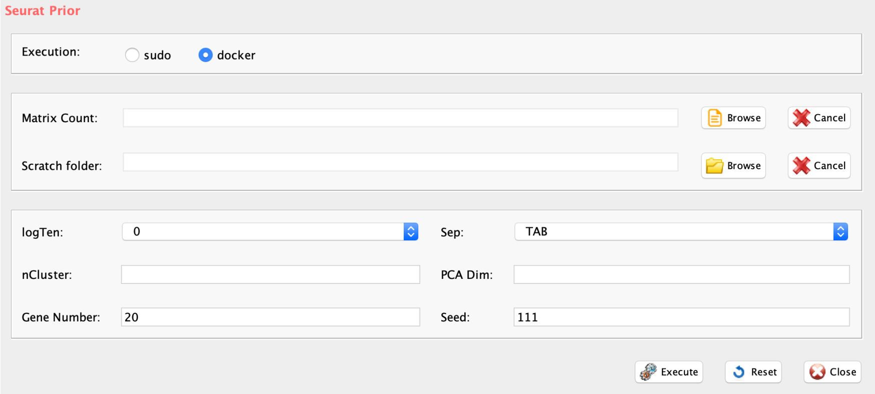

Figure 62: GUI: Seurat genes prioritization panel

- *Parameters* (only those without default; for the full list of parameters please refer to the function help) (Figure 62):
  - *scratch.folder*, a character string indicating the path of the scratch folder
  - *file (GUI Matrix count)*, a character string indicating the path of the file, with file name and extension included
  - *separator*, separator used in count file, e.g. '\t', ','
  - *logTen*, 1 if the count matrix is already in log10, 0 otherwise
  - *seed*, important value to reproduce the same results with same input
  - *PCADim*, dimensions of PCA for Seurat clustering
  - *geneNumber*, numbers of specific genes for each cluster
  - *nCluster*, number of cluster analysis.

```
system("wget http://130.192.119.59/public/section4.1_examples.zip")
unzip("section4.1_examples.zip")
setwd("section4.1_examples")

library(rCASC)

#running Seurat PC distribution
#N.B. Seurat functions requires in input raw counts or log10 transformed raw counts
seuratPCAEval(group="docker",scratch.folder="/data/scratch/",
              file=paste(getwd(), "bmsnkn_5x100cells.txt", sep="/"),
              separator="\t", logTen = 0, seed = 111)

#running Seurat clustering with bootstrap
seuratBootstrap(group="docker",scratch.folder="/data/scratch/",file=paste(getwd(), "bmsnkn_5x100cells.txt", sep="/"),
                nPerm=160, permAtTime=8, percent=10, separator="\t",logTen=0, pcaDimensions=6, seed=111)
```

```
#running Seurat clustering with bootstrap: elbow was detected at PC 6, see Section 6.
seuratPrior(group="docker", scratch.folder="/data/scratch/",
            file=paste(getwd(), "bmsnkn_5x100cells.txt", sep="/"), separator="\t",
            logTen=0, seed=111, PCADim=6, geneNumber=20, nCluster=5)
```

The output is a file with the prefix **Markers_**

	p_val	avg_logFC	pct.1	pct.2	p_val_adj	cluster	gene
1	3.22E-52	1.5362096	0.921	0.344	1.05E-47	1	ENSG00000111716
2	3.74E-46	0.844168	0.993	0.834	1.22E-41	1	ENSG00000122406
3	1.36E-45	2.2045356	0.57	0.034	4.46E-41	1	ENSG00000198851
4	2.52E-43	2.0700739	0.503	0.011	8.24E-39	1	ENSG00000167286
5	9.59E-26	1.3982863	0.589	0.149	3.14E-21	1	ENSG00000142546
6	1.17E-25	0.9933811	0.391	0.034	3.83E-21	1	ENSG00000166681
7	1.77E-25	0.845277	0.901	0.544	5.81E-21	1	ENSG00000181163
8	1.17E-23	1.0833788	0.821	0.367	3.83E-19	1	ENSG00000133872
9	2.47E-23	1.4599244	0.377	0.046	8.07E-19	1	ENSG00000139193
10	3.27E-19	0.8650031	0.338	0.04	1.07E-14	1	ENSG00000078596
11	1.83E-17	0.798671	0.709	0.301	5.99E-13	1	ENSG00000136732
12	5.46E-17	0.8894942	0.285	0.032	1.79E-12	1	ENSG00000237943
13	2.65E-16	0.7977282	0.649	0.255	8.66E-12	1	ENSG00000100380
14	1.61E-14	0.9703806	0.318	0.063	5.27E-10	1	ENSG00000100100
15	3.35E-14	0.9499176	0.311	0.063	1.10E-09	1	ENSG00000185198
16	1.42E-13	1.0419252	0.305	0.063	4.64E-09	1	ENSG00000257698
17	5.14E-12	0.8403336	0.483	0.189	1.68E-07	1	ENSG00000179144
18	7.95E-12	0.8991283	0.358	0.112	2.60E-07	1	ENSG00000182866
19	1.44E-11	0.8572148	0.444	0.158	4.72E-07	1	ENSG00000188404
20	6.99E-09	0.9712438	0.695	0.593	2.29E-04	1	ENSG00000171223
21	6.93E-88	3.0576497	0.928	0.041	2.27E-83	2	ENSG00000105369
22	1.15E-80	2.7387083	0.91	0.062	3.76E-76	2	ENSG00000007312
23	1.89E-60	2.2059988	0.613	0.003	6.20E-56	2	ENSG00000156738

Figure 63: Seurat gene prioritization

Section 8 Supporting tools

The Conversion of a dense matrix, with 0s in sparse format, not including 0s, is performed by **csvToSparse** function and the conversion from a sparse to a dense matrix is done by **h5tocsv**

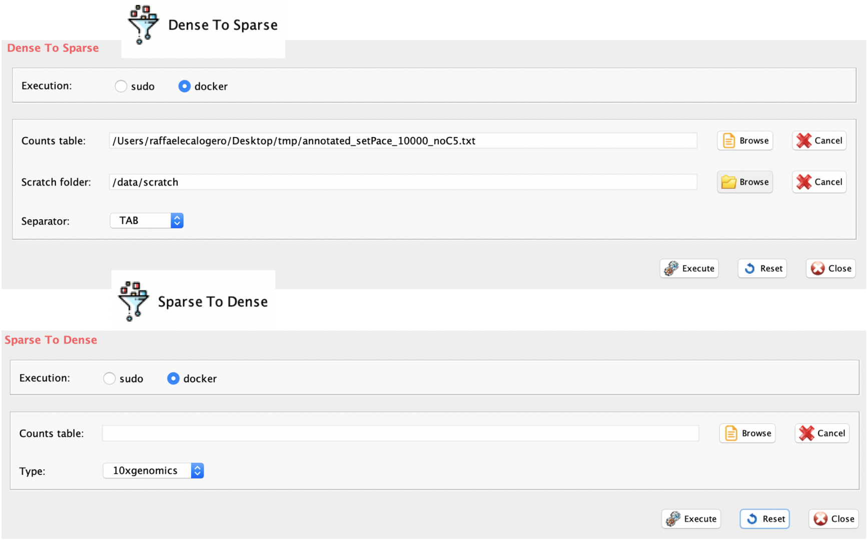

Figure 64: GUI: dense to sparse and sparse to dense matrix conversion panels

- *csvToSparse parameters* (only those without default; for the full list of parameters please refer to the function help) (Figure 64):
  - *group*, a character string. Two options: sudo or docker, depending to which group the user belongs
  - *scratch.folder*, a character string indicating the path of the scratch folder
  - *file*, a character string indicating the path of the file, with file name and extension included
  - *separator*, separator used in count file, e.g. '\t', ','

```
system("wget http://130.192.119.59/public/annotated_setPace_10000_noC5.txt.zip")
unzip("annotated_setPace_10000_noC5.txt.zip")
csvToSparse(group="docker", scratch="/data/scratch", file=paste(getwd(),
            "annotated_setPace_10000_noC5.txt", sep="/"), separator="\t")
```

- *h5tocsv parameters* (only those without default; for the full list of parameters please refer to the function help) (Figure 64):
  - *group*, a character string. Two options: sudo or docker, depending to which group the user belongs

+*file*, path of the sparse matrix file. For h5 file the full path MUST be included. For mtx matrix the folder MUST contain tsv and mtx files and the FULL path to mtx matrix MUST be provided

  - *type*, h5 refers to h5 files and 10xgenomics to the folder containing barcodes.tsv, genes.tsv and matrix.mtx

```r
system("wget http://130.192.119.59/public/annotated_setPace_10000_noC5.txt.zip")
unzip("annotated_setPace_10000_noC5.txt.zip")
csvToSparse(group="docker", scratch="/data/scratch", file=paste(getwd(),
            "annotated_setPace_10000_noC5.txt", sep="/"), separator="\t")
h5tocsv(group="docker", file=paste(getwd(),"matrix.mtx",sep="/"), type="10xgenomics")
```

A random subset from a matrix can be extracted using **subSetCell** function. Two matrices can be merged using **mergeMatrix** function.

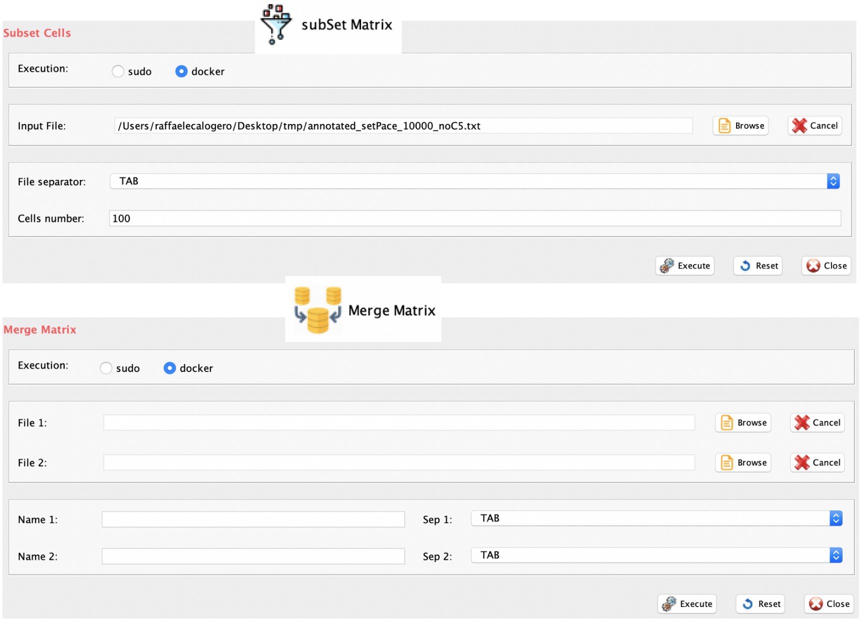

Figure 65: GUI: Random selection a subset of cells from a matrix file panel and merge matrices panel

- *subSetCell parameters* (only those without default; for the full list of parameters please refer to the function help) (Figure 65):
  - *group*, a character string. Two options: sudo or docker, depending to which group the user belongs
  - *scratch.folder*, a character string indicating the path of the scratch folder
  - *file*, a character string indicating the path of the file, with file name and extension included
  - *separator*, separator used in count file, e.g. ‘‘,’,’
  - *cells.number*, number of cells to be extracted
- *mergeMatrix parameters* (only those without default; for the full list of parameters please refer to the function help) (Figure 65):
  - *group*, a character string. Two options: sudo or docker, depending to which group the user belongs
  - *scratch.folder*, a character string indicating the path of the scratch folder
  - *file*, a character string indicating the path of the file, with file name and extension included
  - *separator*, separator used in count file, e.g. ‘‘,’,’
  - *cells.number*, number of cells to be extracted

```
system("wget http://130.192.119.59/public/annotated_setPace_10000_noC5.txt.zip")
unzip("annotated_setPace_10000_noC5.txt.zip")

subSetCell(group="docker", scratch.folder="/data/scratch",
            file=paste(getwd(), "annotated_setPace_10000_noC5.txt",sep="/"),
            separator="\t", cells.number=200)

mergeMatrix(group="docker", scratch.folder="/data/scratch",file1=paste(getwd(),"annotated_setPace_10000_
```

The function **dimensions** generates a file containing the size of the matrix under analysis.

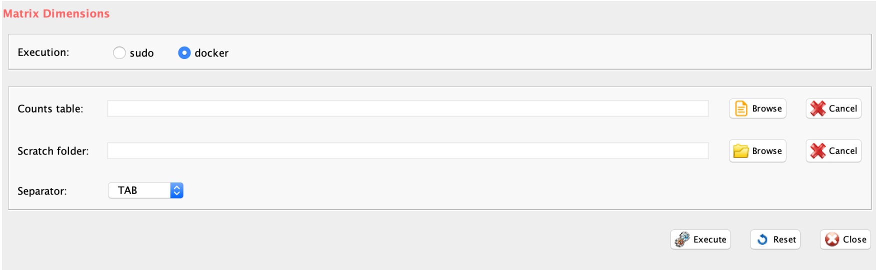

Figure 66: GUI: Calculating the number of rows and columns panel

- *dimensions parameters* (only those without default; for the full list of parameters please refer to the function help) (Figure 66):
  - *group*, a character string. Two options: sudo or docker, depending to which group the user belongs

+*scratch.folder*, a character string indicating the path of the scratch folder

+*file*, a character string indicating the path of the file, with file name and extension included

+*separator*, separator used in count file, e.g. ‘,’

```
system("wget http://130.192.119.59/public/annotated_setPace_10000_noC5.txt.zip")
unzip("annotated_setPace_10000_noC5.txt.zip")

dimensions(group="docker", scratch.folder="/data/scratch",
           file=paste(getwd(),"annotated_setPace_10000_noC5.txt", sep="/"),
           separator="\t")
```

Section 9 An example of rCASC analysis: *GEO:GSE106264*.

Pace et al. explored the role of histone methyltransferase Suv39h1 in murine CD8+ T cells activated after Listeria monocytogenes infection. They showed that Suv39h1 controls the expression of a set of stem cell-related memory genes and its silencing increases long-term memory reprogramming capacity. The single-cell sequencing data from the *Pace's Science paper*:

- naive CD8+ T lymphocytes, here named **N**
- CD8+ T cells activated after Listeria monocytogenes infection, here named **NA**
- naive Suv39h1-defective CD8+ T lymphocytes, here named **Nd**
- Suv39h1-defective CD8+ T cells activated after Listeria monocytogenes infection, here named **NdA**

Section 9.1 Selecting subsets of cells with the highest number of called genes.

On the basis of our observation in **Section 3.2.1**, we selected, for each of the above mentioned groups, the cells with higher number of total reads/cell. Specifically, we analyzed 600 cells, combining respectively 50 cells from **N** and **Nd** and 250 cells for **NA** and **NdA**. The rational of the different number of cells for the naive and the activated is due to the expectation that activated cells will be organize in multiple clusters and naive are expected to be homogeneous. Cell labels in the counts table were modified adding the groups *N*, *Nd*, *NA* and *NdA*.

Using this dataset, we tried to address the following questions:

1. Does Suv39h1 silence gene expression program in naive CD8+ T cells?
2. Does Suv39h1 silence gene expression program in activated CD8+ T cells?
3. Does Suv39h1 silencing affect the differentiation of new subsets of activated CD8+ T lymphocytes?

With the script below we have performed the following steps:

- annotating the counts table with **scannobyGtf** function,
- removing the ribosomal and mitochondrial genes with **scannobyGtf** function,
- selecting the most expressed 10K genes for the analysis with **topx** function,
- detecting the range of interesting number of clusters to be used for data partitioning (6-9) with **clusterNgriph**
- identifying 6 as the optimal number of clusters and evaluated the cell stability in each cluster with **simlrBootstrap**
- generating the video of the bootstrap analysis with **bootstrapsVideo**

```r
#Dataset constructed for demonstration purposes using raw counts from GSE106264.
#Raw counts were generated with cellranger 2.0 from h5 files.
system("wget http://130.192.119.59/public/setPace.txt.zip")
unzip("setPace.txt.zip")

#annotating genes and removing ribosomal and mitochondrial proteins
scannobyGtf(group="docker", file=paste(getwd(),"setPace.txt",sep="/"),
                    gtf.name="genome_mm10.gtf", biotype="protein_coding",
                    mt=FALSE, ribo.proteins=FALSE,umiXgene=3, riboStart.percentage=0,
                    riboEnd.percentage=100, mitoStart.percentage=0, mitoEnd.percentage=100)

#selecting 10K top expressed genes
topx(data.folder=getwd(),file.name="annotated_setPace.txt",threshold=10000, logged=FALSE)

#defining the range of number of clusters to be investigated
clusterNgriph(group="docker",scratch.folder="/data/scratch/",file=paste(getwd(),
              "annotated_setPace_10000.txt", sep="/"), nPerm=160,
              permAtTime=8, percent=10, separator="\t",logTen=0, seed=111)
#38 mins on SeqBox hardware

#running the SIMLR clustering in the range 6-9 detected by clusterNgriph
simlrBootstrap(group="docker",scratch.folder="/data/scratch/",
               file=paste(getwd(), "annotated_setPace_10000.txt", sep="/"),
               nPerm=160, permAtTime=8, percent=10, range1=6, range2=9,
               separator="\t",logTen=0, seed=111)

# visualizing cells instability
bootstrapsVideo(group="docker", scratch.folder="/data/scratch",
                file=paste(getwd(), "annotated_setPace_10000.txt", sep="/"),
                nCluster=6, separator="\t", framePP=200, permutationNumber=80)
```

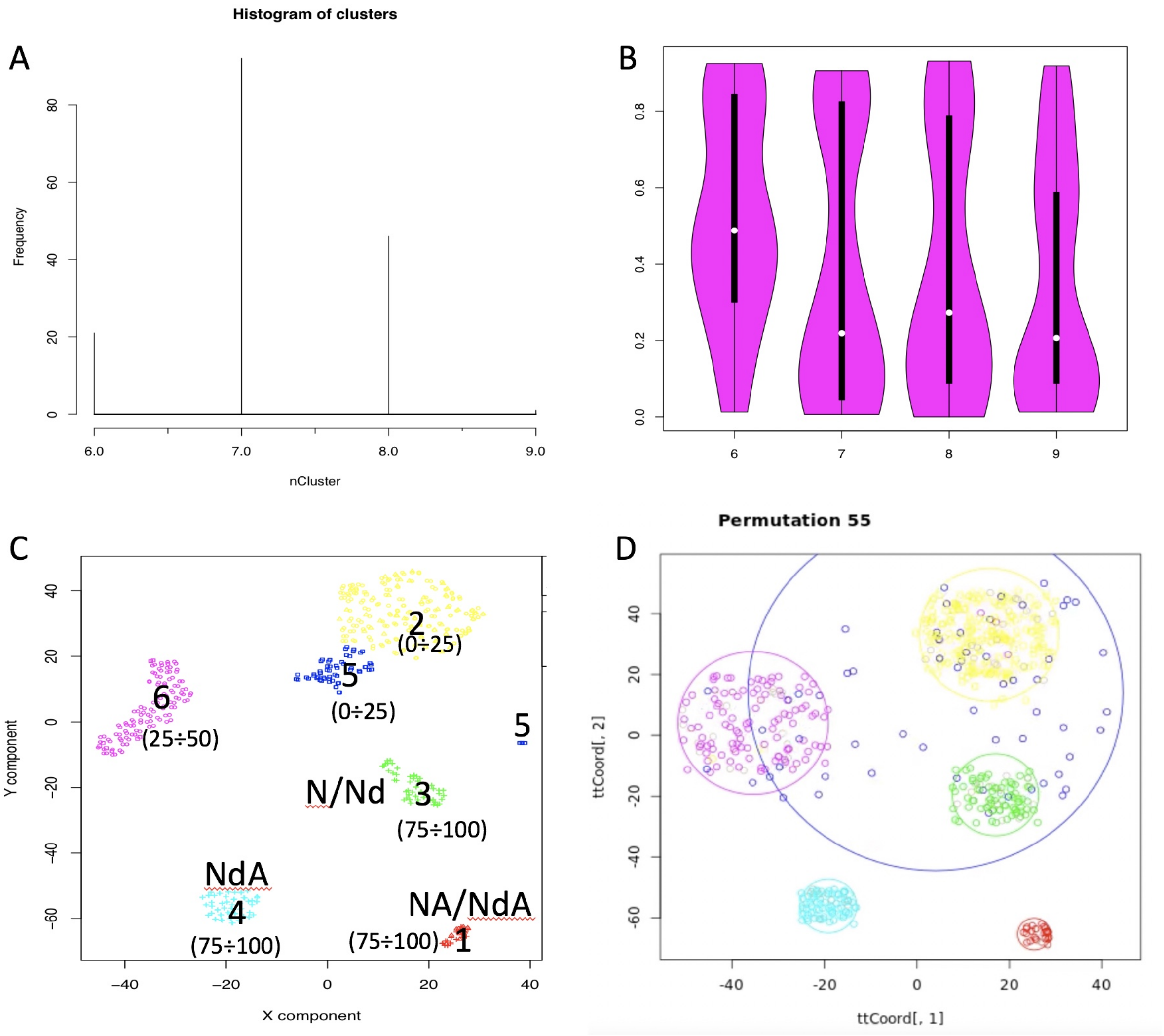

Figure 67: Analysis of a subset of cells from GSE106264 dataset. A) Clusters detected by clusterNgriph. B) Cell stability score detected by simlrBootstrap. C) Clusterization with 6 clusters with simlrBootstrap, in parenthesis is given the overall stability score of each cluster. The components of each cluster, in terms of cells experiment groups, are also indicated for the most stable clusters. D) Permutation 55 extracted from the video generate with bootstrapsVideo.

In Figure 67 are summarized the results of the analysis executed on the Pace's dataset. A limitation of the clustering based on SIMLR is due to the need of providing as input the number of clusters (k) in which the data should be organized. Instead of asking to user to define arbitrarily the k number of clusters, we used *griph* as tool to identify a range of k clusters to be inspected by SIMLR. Figure 67A shows the frequency of k number of clusters, in which the Pace's dataset can be organized using griph software, upon 160 bootstraps in which 10% of the cells is randomly removed from the initial data set. Griph analysis identify a range of clusters going from k=6 to k=9. K=7 is the most represented data organization detected by griph, followed

by 8, 6 and 9 clusters. The range of k clusters detected using griph is then investigated with SIMLR. SIMLR is run for each k of the k-range defined with griph tool. CSS violin plot (Figure 67B) shows that the mean stability for k=6 ($\overline{CSS} \sim 0.5$) is higher than the others ks ($\overline{CSS} < 0.3$). Clusters k=7 and k=8 do not represent the most stable organizations in terms of CSS (Figure 67B), although they are the most frequent organizations observed in griph analysis (Figure 67A). Since the best $\overline{CSS}$ is observed in k=6, we explored these clusters (Figure 67C). In Figure 67C clusters 1, 3 and 4 show a quite good stability, since cells stay in these clusters between 75 to 100% of the bootstraps. The inspection of Pace's experiment groups organization (i.e. **N**= naïve WT, **Nd**= naïve Suv39h1 KO, **NA**=activated WT, **NdA**=activated Suv39h1 KO) in k=6 clusters, Figure 67C, show that cluster number 4 is the only one containing only NdA (33% of the total NdA) cells. Thus, suggesting that a subpopulation of activated Suv39h1-silenced cells has a specific transcription profile, which differentiates them from all wild type activated cells. Another interesting cluster is number 6, where the amount of NA and NdA is unbalance, 35% NA and 13.6% of NdA, suggesting that Suv39h1 silencing does not guarantee at the same efficiency the differentiation of this cell subpopulation as in the case of wild type cells. Cluster 5 is the most heterogeneous cluster. It is composed by 6 N cells, 2 Nd cells, 34 NA cells, 21 NdA cells. Despite the presence of a limited number of naive cells, which might be explained as partially activated, cluster 5 is composed by an unbalance number of activated cells, i.e. 13.6% NA and 8.4% NdA of total cells. However, since cluster 5 is characterized by a very low CSS (0-25%) it is possible that this cluster contains cells localized at the boundaries of clusters 2, 3 and 6. On the other side clusters 1, 2, 3 have nearly the same number of wild type and Suv39h1 silenced cells, suggesting that these subsets of cells are not influenced by Suv39h1 silencing:

- cluster 1 contains nearly the same amount of activated wild type, 16 NA (6.4%), and Suv39h1 KO cells, 14 NdA (5.6%);
- cluster 2 is made of 44.8% of NA and 39.6% of NdA cells;
- cluster 3 is made of 44 N (88%) and 48 Nd (96%).

NB % always refers to the total number of cells used in this example (50 N, 50 Nd, 250 NA, 250 NdA); *annotated_setPace_10000_clustering.output.txt*, contains all information required to reproduce clusters shown in Figure 67C. In Figure 67D it is shown permutation 55 of the output of **bootstrapsVideo**, which can be seen *here*. This permutation is representative of the majority of the observed permutations. From **bootstrapsVideo** analysis is clear that cluster 5 is strongly affecting the cell score stability of clusters 6 and 2, and for less extent that of cluster 3.

This analysis can provide some preliminary answers to the questions raised above:

1. Does Suv39h1 silence gene expression programs in naive CD8+ T cells?
    a. No, naive cells cluster together independently from Suv39h1 silencing.
2. Does Suv39h1 silence gene expression programs in activated CD8+ T cells?
    a. Yes, cluster 4 is only made of an activated Suv39h1 KO sub-population. Furthermore, although quite unstable, cluster 6 sub-population is less represented in activated Suv39h1 silenced cells,

suggesting a reduction in efficiency in the differentiation of this sub-population upon Suv39h1 silencing.

3. Does Suv39h1 silencing affect the differentiation of new subsets of activated CD8+ T lymphocytes?

    a. Yes, cluster 4 represents a unique Suv39h1 subset of activated CD8+ T cells.

Section 9.2 Improving clustering results

On the basis of Figure 67D and the output of *bootstrapsVideo* clearly cells in cluster 5 represent a strong interference for an efficient clustering. Thus, we investigated if, removing cluster 5 cells (Figure 67C), cell stability score for cluster 6 and 2 could be improved.

```
system("wget http://130.192.119.59/public/annotated_setPace_10000.txt.zip")
unzip("annotated_setPace_10000.txt.zip")
system("wget http://130.192.119.59/public/annotated_setPace_10000_clustering.output.txt")

#removing cluster 5 cells from annotated_setPace_10000.txt

pace <- read.table("annotated_setPace_10000.txt", sep="\t", header=T, row.names=1)
clusters.info <- read.table("annotated_setPace_10000_clustering.output.txt", sep="\t", header=T, row.names=1)

discard <- rownames(clusters.info)[which(clusters.info$Belonging_Cluster==5)]

pace <- pace[,which(!names(pace)%in%discard)]
write.table(pace, "annotated_setPace_10000_noC5.txt", sep="\t", col.names=NA)

#defining the range of number of clusters to be investigated
clusterNgriph(group="docker",scratch.folder="/data/scratch/",file=paste(getwd(),
              "annotated_setPace_10000_noC5.txt", sep="/"), nPerm=160,
              permAtTime=8, percent=10, separator="\t",logTen=0, seed=111)


#running the SIMLR clustering in the range 5-9 detected by clusterNgriph
simlrBootstrap(group="docker",scratch.folder="/data/scratch/",
               file=paste(getwd(), "annotated_setPace_10000.txt", sep="/"),
               nPerm=160, permAtTime=8, percent=10, range1=5, range2=9,
               separator="\t",logTen=0, seed=111)
#execution time 276 mins in SeqBox
```

The results of the analysis done removing cells associated to cluster 5 are shown in Figure 68. The range of clusters suggested by **clusterNgriph** are between 5 and 9, Figure 68A. The best cells stability score is observable for 5 clusters, Figure 68B. **simlrBootstrap** results show a large improvement of the overall stability of the clusters Figure 68C with respect to the previous analysis, Figure 67C. It is also notable that the samples organization with in clusters agrees with preliminary answers in Section 5.1.1 .

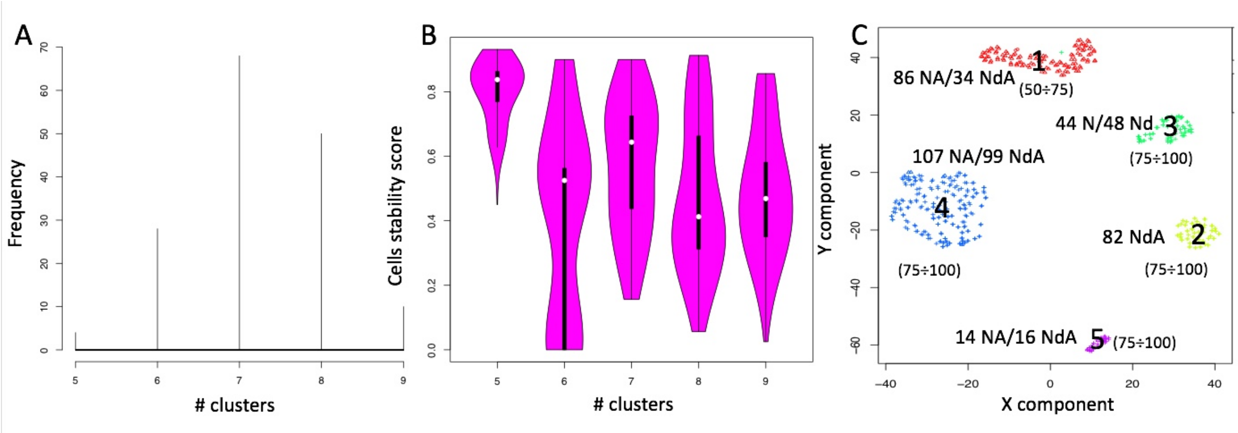

Figure 68: Analysis of Pace dataset. A) Clusters detected by clusterNgriph. B) Cell stability score detected by simlrBootstrap. C) Clusterization with 5 clusters with simlrBootstrap, in parenthesis is given the overall stability score of each cluster. The components of each cluster, in terms of cells experiment groups, are also indicated for the most stable clusters.

Section 9.3 Detecting the genes playing the major role in clusters generation: EdgeR ANOVA-like analysis

We detected a total of 2742 differentially expressed genes using the naive cluster 3 as reference in an ANOVA-like analysis, see **Section 6.1**.

```
system("wget http://130.192.119.59/public/annotated_setPace_10000_noC5_clustering.output.txt")
system("wget http://130.192.119.59/public/annotated_setPace_10000_noC5.txt.zip")
unzip("annotated_setPace_10000_noC5.txt.zip")

anovaLike(group="docker", data.folder=getwd(), counts.table="annotated_setPace_10000_noC5.txt",
        file.type="txt", cluster.file="annotated_setPace_10000_noC5_clustering.output.txt", ref.cluster=3,
        logFC.threshold=1, FDR.threshold=0.05, logCPM.threshold=4, plot=TRUE)

#the output of interest is logFC_filtered_DE_annotated_setPace_10000_noC5_reordered.txt
```

The following analysis focus only on up-regulated genes, i.e. those gene specifically more expressed in a cluster with respect to the naive cluster used as reference in the ANOVA-like.

```
system("wget http://130.192.119.59/public/clusters.features.zip")
unzip("clusters.features.zip")
setwd("clusters.features")

clustersFeatures(group="docker", data.folder=getwd(),
         logFC.table="logFC_filtered_DE_annotated_setPace_10000_noC5_reordered.txt",
         counts.table="annotated_setPace_10000_noC5_reordered.txt", delta=0.5)
```

We analyzed the results coming out of the **clustersFeatures** selection focusing on the two clusters that are

different in cell ratio between wt and Suv39h1-defective T-cells: cluster 1, showing a 2.4:1 ratio between wt and Suv39h1-defective T-cells, and 2, only detected in Suv39h1-defective T-cells, Figure 68C. 31 genes were detected as specific for cluster 1. *EnrichR* analysis indicated the over-representation of STAT3 in ENCODE and ChEA Consensus TFs from ChIP-X datasets and TNFRSF13B, CCL5, CCL4 and CXCR6 in Cytokine-cytokine receptor interaction dataset. On the basis of the above observations and on a PUBMED search (*Ccl5*, *STAT3*, *Ccl9* and *Cxcr6*), we suggest that cluster 1 is made of early activated precursors having some traits of memory cells. This cluster is also characterized by an unbalance ratio between activated wt T-cells and Suv39h1-defective T-cells.

The cluster 2 has some traits of memory cells, on the basis of EnrichR analysis of 100 genes. Enrichment analysis shows enrichment for STAT3 transcription specific paths and GO Biological process enrichment for **response to cytokine**. Furthermore, a PUBMED search showed the presence in this cluster of memory specific genes such as *TCF7*, *FOXO1*, *Bach2*, *Ccr7*, *Id3*, *Il7r*, *Myc*, *Pdk1*, *Socs3*, *Tnfsf8*,

The analysis of cluster 2 was extended using Ingenuity Pathways Analysis (IPA). This sub-population of cells, only detectable within Suv39h1-defective T-cells, is enriched for immuno-related functions: Development of hematopoietic progenitor cells, Adhesion of lymphocytes, Quantity of T-lymphocytes, systemic autoimmune syndrome. A subset of cluster 2 genes can be also aggregated in a network characterized by various transcription factors (Figure 69). Interestingly, in this network are present Id3, Bach2 IL7R and Myc. Combining the information that Bach2 is associated to the generation (*Roychoudhuri et al. 2016*) of long-lived memory cells and Id3 to their maintenance (*Yang et al. 2011*) together with the knowledge that IL7R/Myc expression is found to be associated to a longer persistence of mouse memory T-cell (*Pace et al. 2018*) it could be suggested that cells belonging to this cluster are precursors of long-lived memory cells.

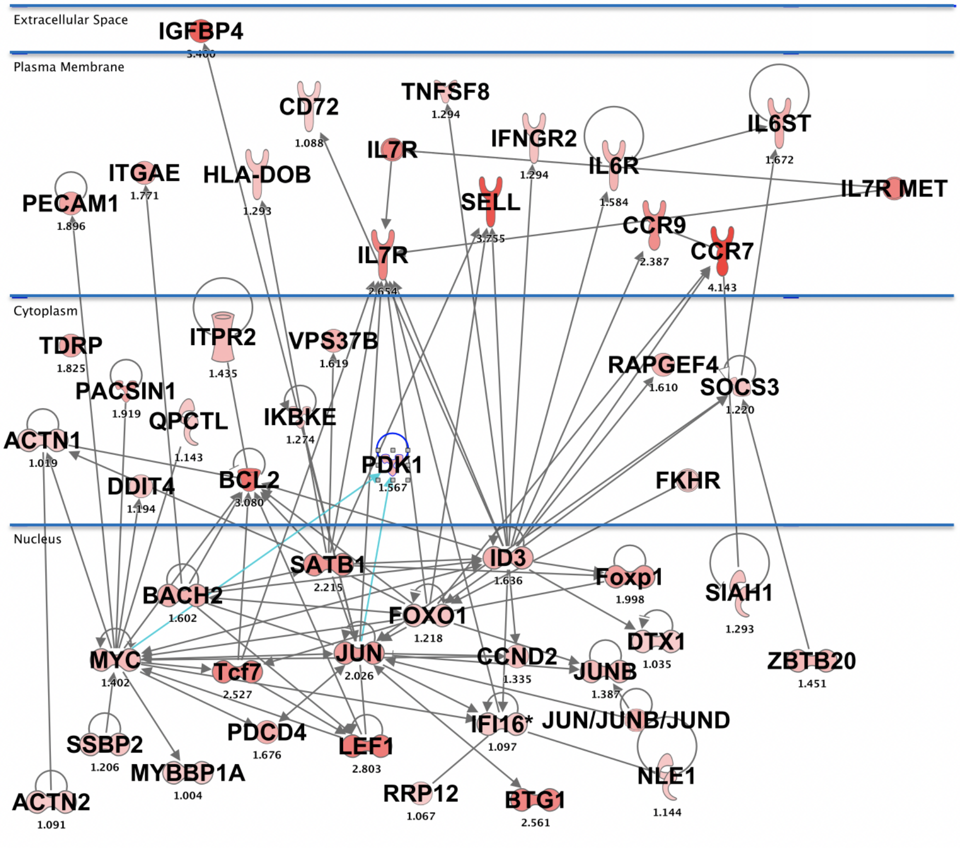

Figure 69: IPA C2 connected genes.

The paper *Pace* showed that Suv39h1-defective CD8+ T cells show sustained survival and increased long-term memory reprogramming capacity. Our reanalysis with rCASC extends the information provided from the single cell analysis in Pace paper, suggesting the presence of an enriched Suv39h1-defective memory subset, cluster 2 in Figure 68C. Noteworthy, this example suggests that even few hundreds cells are sufficient to discriminate between sub-populations.

Section 10 Tips and tricks

In this section we highlight some points that might help data analysis.

Section 10.1 Data preprocessing

Lorenz statistics (*lorenzeFilter* function) was designed using C1 - Fluidigm and smart-seq libraries. There are no evidences that it performes optimally on UMI based sequencing. In case of UMI, we suggest the use of *topx* and *filterZeros* functions, which respectively allow the selection of a user defined set of genes with high

dispersion/expression and the removal of genes with a user defined fraction of 0s.

Unless there is a specific interest on mitochondrial and ribosomal protein genes, we suggest to remove them before performing clustering (*scannobyGtf*, function). Specifically ribosomal genes represent a high fraction of the UMI counts associated to a cell and might result in driving the cells partioning.

The normalization for sequencing depth (*checkCountDepth* and *scnorm* functions) was designed for smart-seq single cell sequencing data. This normalization can be applied to UMI sequencing data but it requires at least 10K UMIs/cell.

Cell cycle estimation (*recatPrediction* function) and removal (*ccRemove** function) were designed for smart-seq single cell sequencing data. In our hands they are also working on UMI seqencing data althought, if the cell number exceed few thousands, cell cycle estimation became very slow. We suggest to perform cell cycle estimation on a random subset of few hundreds cells (*subSetCell* function) of the data set under analysis.

Section 10.2 Clustering

As part of the output of the clustering procedure there is a plot of the number of detected genes as function of the number of cell UMIs. In this plot, each cell is colored on the basis of the cluster it belongs. This plot helps in understanding if cell clusters simply correlate with number of genes called in each cell. Ideally, there should not be any correlation between clusters and number of genes detected in a cell, since clustering should be driven by cells' biological content and not by the number of detected genes.

On the basis of our tests SIMLR and Seurat generated results comparable in terms of CSS and clusters homogeneity. Since griph and scanpy results correlate less with SIMLR and Seurat, we suggest to use them only if a very large set of cells has to be analysed, e.g. >20K cells.
