# Supplementary material for: rCASC: reproducible classification analysis of single-cell sequencing data
Source: Gigascience. 2019 Sep 8;8(9):giz105. doi: 10.1093/gigascience/giz105 (PMC6732171; doi:10.1093/gigascience/giz105)
Supplement: giz105_Response_to_Reviewer_Comments_Original_Submission [file giz105_response_to_reviewer_comments_original_submission.pdf]

Author's Response To Reviewer Comments

Close

GIGA-D-18-00522
Dear Editor,
First of all, we wish to thank the reviewers for their valuable comments and useful suggestions which helped us to improve the paper and the tool.
Editor request: Please register any new software application in the SciCrunch.org database to receive a RRID (Research Resource Identification Initiative ID) number, and include this in your manuscript. This will facilitate tracking, reproducibility and re-use of your tool.
Answer: we registered rCASC to SciCrunch and it is now associated with the Research Resource Identification Initiative ID : SCR_017005
Reviewer #1:
Comments to the Author:
This paper presents a pipeline to infer single-cell clusters using scRNA-Seq data (rCASC), infer significant features linked to each cluster, and can analyse various metrics during the processing. Notably, the results of the pipeline can be divided into 3 major outputs, A) cells-features matrix generation, B) Clustering, and C) inference of significant features per clusters. Also, the pipeline is able to perform various additional substeps such as Matrix preprocessing (normalization), outliers removal, features removal, cell cycle specific features removal. The pipeline is implemented in R using Docker containers and has a GUI interface coded in Java. Finally; the authors claimed have invented a metric: the CSS, to evaluate cluster stability in their single-cell analyses. First, It is a pleasant surprise to be able to install everything needed to perform scRNA-Seq analysis with few simple commands (with exception of Docker which can be tricky for non IT people). Also, developing scRNA-Seq analytical toolbox easy to use and efficient are an innovative direction due to the importance and the multidisciplinary aspect of the field. However, I have major concerns which I think should be addressed before publication.
Major Comments:
Comment 1: First, the abstract and the text contain different confusing aspects that must be rewritten. The authors describe a "supervised approach": SIMLR which is seen as the alternative of the "Seurat clustering". From my knowledge, SIMLR is a clustering workflow and thus is also an unsupervised approach, by contrast with any other supervised approaches using training datasets as input (classification/regression...). I don't know what is a "supervised clustering" if not a classification procedure. Clustering are always unsupervised with the exception of "semi-supervised" clustering (use of seed samples).
Answer 1: As pointed out by the reviewer both Seurat and SIMLR use an unsupervised approach, and they mainly differ in the metrics driving the clustering analysis. SIMLR is capable of learning an appropriate cell-to-cell similarity metric from the input single-cell data and to exploit it for the clustering task. In details, the learning phase identifies a distance metric that best fits the structure of the data by combining multiple Gaussian kernels. This allows the tool to deal with the large noise and drop-out effect of single-cell data that could not be easily fitted with specific statistical assumptions made by standard dimension reduction algorithms. Differently, Seurat clustering algorithm is based on the Euclidean distance in PCA space, and refines the edge weights between any two cells based on the shared overlap in their local neighbourhoods (Jaccard similarity). Since these two clustering approaches have their specific criticality and strengths we decided to integrate both of them in our framework. Finally, according to the reviewer' comments we modified the following sentences:
In Abstract-Findings: “Subpopulations discovery can be instead achieved using unsupervised and supervised clustering technique”
was modified in
“Subpopulations discovery can be instead achieved using different clustering techniques based on different distance metrics”.
In keywords: “supervised clustering, unsupervised clustering”
was modified in
“clustering”
In Findings: “Therefore, rCASC provides raw data preprocessing, subpopulation discovery via

supervised/unsupervised clustering and cluster-specific genes-signatures detection”
was modified in
“Therefore, rCASC provides raw data preprocessing, subpopulation discovery via different clustering approaches and cluster-specific genes-signatures detection”
“rCASC implements as unsupervised clustering tool and as supervised clustering tools”.
was modified in
“rCASC integrates two clustering tools, namely Seurat [13] and SIMLR [15], which mainly differ in the metrics driving the clustering analysis”.
Comment 2: Also, I don't understand why this package is superior in term of "Computational and Functional" reproducibility compared with any other packages for which a similar reasoning can be also applied.
Answer 2: We would like to thank the reviewer to give us the possibility to explain better this aspect. rCASC is one of the tools developed under the umbrella of the Reproducible Bioinformatics project (http://www.reproducible-bioinformatics.org/), an open-source community aimed to provide to biologists and medical scientists, without scripting skills, a easy to use framework, which also guarantees the ability to reproduce results independently by the underlying hardware, using docker containerization (computational reproducibility), and providing tools that fulfil the best practice rules for reproducible computational research, proposed in 2013 by Sandve [PLoS computational biology 2013, 9(10)] (functional reproducibility). The reproducible bioinformatics project was founded and it is maintained by the research team of Elixir node at University of Turin. The reproducible bioinformatics project was published on BMC Bioinformatics (Kulkarni et al. 2018). An example of stand-alone hardware/software infrastructure for bulk RNAseq, developed within the Reproducible Bioinformatics project, was described in Beccuti et al. (Bioinformatics 2017). Thus, to the best of our knowledge, rCASC is the only computational framework, which provides a complete computational reproducibility for integrated analysis of single cell data (from counts generation to cell subpopulation identification). Last but not least rCASC is listed within the tools developed by the Italian Elixir node (https://bio.tools/rCASC).
Finally, the following phrase in the Findings section: “In this context rCASC provides a modular workflow to address at the same time the problem of functional and computational reproducibility. rCASC provides single cell analysis functionalities within the reproducible rules described by Sandve [5]. rCASC is part of the Reproducible Bioinformatics Project [6], which is a project designed to provide to the biological community a reproducible and user-friendly bioinformatics ecosystem [8].”
was modified in:
“rCASC is one of the tools developed under the umbrella of the Reproducible Bioinformatics project (http://www.reproducible-bioinformatics.org/), an open-source community aimed to provide to biologists and medical scientists, without advance scripting skills, an easy to use framework, which also guarantees the ability to reproduce results independently by the underlying hardware, using docker containerization (computational reproducibility). Indeed, it was developed following the best practice rules for reproducible computational research, proposed in 2013 by Sandve [PLoS computational biology 2013, 9(10)] (functional reproducibility). The reproducible bioinformatics project was founded and it is maintained by the research team of Elixir node at University of Turin. The reproducible bioinformatics project was published on BMC Bioinformatics (Kulkarni et al. 2018). An example of stand-alone hardware/software infrastructure for bulk RNAseq, developed within the Reproducible Bioinformatics project, was described in Beccuti et al. (Bioinformatics 2017). Thus, to the best of our knowledge, rCASC is the only computational framework, which provides both computational and functional reproducibility for an integrated analysis of single cell data, from counts generation to cell subpopulation identification. rCASC is also listed within the tools developed by the Italian Elixir node (https://bio.tools/rCASC).”
Comment 3: Then, the authors claimed to have invented a new metric: the "Cell-stability Score", which is based on the computation of a stability score by clustering multiple bootstrap sampling and computing the jaccard index. Clustering stability measurement is not new and previous works already described more formally the use of bootstrapping together with clustering and Jaccard index to estimate cluster stability (http://www.homepages.ucl.ac.uk/~ucakche/papers/clusta.pdf (2006), https://arxiv.org/abs/1503.0205). These example algorithms are not based on single-cell datasets (other stability approaches exist for single-cells), but since the approach described in the first paper is very similar, a more comprehensive bibliography of clustering stability should be present in the manuscript as well a rewriting of the CSS description/notion, highlighting the similarity with previous works.
Answer 3: As mentioned by the reviewer the cell stability score method implemented by us uses
Jaccard
index,
which

is
also
used
in
Hennig
paper
(http://www.homepages.ucl.ac.uk/~ucakche/papers/clusta.pdf 2006). However, between our approach and the one of Hennig's paper there is a substantial difference: in Hennig's paper the Jaccard index is used to evaluate the similarity between clusters and the calculated score provides an overall quality score for each of the clusters in toto. In our implementation the stability score is not related to the clusters, but it is specific for each cell. We believe that cell stability score allows us to have a more precise view of which are the cells affected by perturbations of the dataset structure.
To better explain this issue, we modify the paper section "rCASC: a single cell analysis workflow designed to provide data reproducibility.", adding the following phrases before the sentence: "To the best of our knowledge, rCASC is the only workflow performing clustering in presence of data perturbation ...":
"Cluster stability is an important topic in Clustering (for a review see von Luxburg 2010). Stability measurement, taking advantage of bootstrapping, was also addressed by Hennig (2007). Specifically, Hennig uses Jaccard index to evaluate the overall stability of each cluster. In rCASC, we have implemented a cell stability score (CSS), which uses the Jaccard index to estimate the stability of each cell in each cluster. CSS provides an enhanced description of each cluster, since it allows the identification of subset of cells, in any cluster, which are particularly sensitive to perturbation of the overall dataset structure, i.e. cell bootstrapping. Moreover, the cluster stability measurement proposed by Henning was included in rCASC. Specifically, we have implemented the "clusterboot "function from the fpc R package (https://cran.r-project.org/web/packages/fpc/index.html), which allows the evaluation of the cluster stability using a personalized clustering function). So far in our knowledge, rCASC is the only single-cell analysis workflow performing clustering in presence of data perturbation ...". We added the Section 5.3 in Supplementary file to describe this functionality.
Comment 4: In term of additional experiments, I think it would be interesting to have an idea of the ratio: number of CPUs/ RAM/ computational time according to: the number of cells / number of features (i.e.: matrix dimension), and read depth (linked to fastq size). More specifically, what are the limiting steps in term of computation? What are the steps the less expensive ? A new figure might be necessary to represent the contribution of each step in term of computation.
Answer 4: We thanks the reviewer for this comment. We added a new figure describing the effect of cells and number of genes on the computation efficacy of SIMLR, Seurat, tSne and griph (Figure 7). In the Methods section we also added the paragraph "Scalability", in which we discussed the computational performance of the used methods.
See also A9 major comments reviewer 2 and A6a to your Comment 6.
Comment 5: Ideally, a comparison with the other cited pipeline would be also interesting, but this amount of work might be out of scope of this study.
Answer 5: We agree with reviewer that this point is out of the scope of our manuscript.
Comment 6:
Q6a) I have some concerns with the choice of clustering algorithms used. Despite Seurat is well established in the community and SIMLR is also a well recognized algorithm, I am not sure if these algorithms can handle very large sparse datasets (i.e. more than 10K cells), that are becoming the new standard in the field. Notably, are these algorithms able to handle sparse data? SIMLR needs a specified K, thus inferring the best K requires to screen amongst an array of Ks and thus might be very time consuming. Would it exist better and simpler alternatives to handle very large and sparse datasets that might be included in rCASC?
A6a) To answer to the above comment we run a performance experiment increasing the cell size and varying the number of genes used in clustering experiment. Please see Answer A9 major comments reviewer 2. A new paragraph, scalability, was added in method section in the main manuscript. In Figure 7A of paragraph scalability, it is shown the computing time (Intel i7 3.5 GHx, 4 cores, 32 GB RAM, 500GB SSD) required to execute 160 permutations on a dataset varying from 200 to 5000 cells using SIMLR, tSne, griph and Seurat. Specifically, Seurat analysis with 5000 cells can be completed in 50 hours. Computing times can be significantly reduced if a high-end multi-cores server is used. We observe that when the number of considered cells overcomes 5000 units then all the clustering tool integrated in rCASC requires more than 32GB RAM.
SIMLR, tSne, griph and Seurat were all designed to work with sparse data.
We agree with reviewer that SIMLR is more computational demanding with respect to the other implemented tools, but it provides very good clustering performance as reported in SIMLR paper

(Wang et al. 2017). SIMLR was also released in a version to handle large scale dataset, but we did not integrate it because, when we tested it, we observed that the sensitivity and specificity of the method were not better of those of tSne (not shown in this manuscript). Of course, we are constantly looking for new clustering methods, proposed in the literature, able to overcame the limitations of the current tools. The latest comparison between clustering methods (Duò et al. 2018 on F1000: https://www.ncbi.nlm.nih.gov/pmc/articles/PMC6134335/) indicates that the clustering algorithm implemented in Seurat toolkit is the most computationally time efficient.
Q6b) Using a clustering stability metric is I think a very good idea. Is it possible to get an average stability score per cluster to have an idea if a cluster is noisy or robust?
A6b) We thank the reviewer with this useful comment. We answer to this point at the above comment 3.
Q6c) Also, even a stable cluster according to a bootstrap experiment is not a guarantee of a "biologically" stable cluster, and can reflect a biased in the method used (for example, a dummy algorithm clustering cells according to their name will produce very stable but useless clusters).
A6c) According to one of the requests of reviewer 2, see A2b major comments reviewer 2, we collected extra data, which are indicating that cell stability score (CSS) inversely correlate with the cells' heterogeneity of clusters, see Section 5 Figure 41 of the supplementary information. Since, we observed that the clusters characterized by high CSS are mainly constituted of cells of the same type, this suggests a correlation with the biological characteristics of the cells.
Q6d) What is the use of griph (Graph Inference of Population Heterogeneity). Why not using the stability measure to estimate the best K?
A6d) Indeed, we use the clusters stability measure to estimate with K is the best choice. However, to provide a suggestion on the range of numbers of clusters to be investigated, we decided to use griph tool, first because it is based on Louvain modularity optimization algorithm, as the Seurat clustering method we have integrated in rCASC. Then, because its execution time is the best between the clustering tools we have implemented in rCASC, see Figure 7 in the main manuscript. We did not implement griph as complete clustering tool in rCASC, because it is not published, yet.
Q6e) The package requires a very large amount of memory to be able to install all the docker dependencies and I was not able to install it on my own computer (out of memory). Is there any way to propose "lighter" versions in order to be able to use it on a standard computer? Overall, I am not sure if all these different steps are always mandatory to obtain biologically meaningful single-cell clusters (Of course, they might be required in some specific cases), compared to more straightforward approaches (matrix creation -> embedding -> clustering).
A6e) Concerning the docker images storage in local computer, it is not necessary to execute the command downloadContainers(), which download all rCASC docker images. In this case, only the required images will be downloaded on the basis of the rCASC function in use. This option will require extra time to execute each new function, since the corresponding docker image needs to be downloaded. We have also added the option "mini" for downloadContainers(), which downloads only 17 dockers out 26 docker images and provides all basic functionalities to handle a single-cell counts table.
Concerning RAM requirements, we can execute all rCASC functions with 16 GB RAM on datasets up 1000 cells. The main limitation is given by the possibility of performing no more than one permutation at a time.
Minor Comments:
Q1) The supplementary files document very rigorously the software which is really pleasant. Some figures are not very informative and might be combined together (For example figures 1, 3 and 4 And figures 2 and 6?).
A1) We thank reviewer for the suggestion, but we prefer to leave these figures separated to keep clear the analysis workflow structure.
Q2) Can you describe briefly what is the Seurat specific normalization?
A2) Seurat normalization step is the one suggested from the Seurat workflow. This information was added in supplementary file: "Before clustering data are normalized as suggested from the Seurat workflow using Seurat NormalizeData function, with LogNormalize as normalization.method and 10000 as scale.factor parameters."
Reviewer #2: The authors present rCASC, an integrated analysis framework for single-cell RNA sequencing data that combines a range of existing and novel computational tools. While some workflows for reproducible scRNA-Seq data analysis exist, the authors provide an analysis strategy using docker containers and a graphical user interface for reproducibility. rCASC is implemented as an R package and as graphical user interface, which allows bioinformaticians as well as biologists with little experience to perform statistical data analysis. However, there are some concerns about the chosen analysis tools and the presentation of results that need to be addressed:
Major comments

Q1) The description of the algorithms used is often not clear. In the main text (p.4 l.16-21), the authors mention normalization and clustering strategies taken from other tools. When other tools are used, it would be good to briefly describe the underlying algorithms (either in the main text or the methods section). For example, Seurat is a toolbox for data analysis and not a clustering tool and it has to be specified which clustering strategy is used. The authors should also make sure to properly cite the original publication of functions implemented in the rCASC toolbox (for example the reCAT function and the griph package).
A1) We added for each function described in section Methods a brief description of the implemented algorithm. We apologize for the error in citing reCAT, we fixed it. Concerning griph, we only provided the github repository, because developers told us that it will be published as part of a larger paper entitled "Self-organization and symmetry breaking in intestinal organoid development" accepted for publication in Nature. However, at the time of this rebuttal the paper is not out, yet. We will update its reference as soon as it will be available.
Q2a) The authors developed the Cell Stability Score based on iteratively clustering a sub-sampled dataset. This is a good approach and informative for cluster stability. It would however be good to visualize the stability scores for datasets subsampled to 20%, 30%, and even 50%.
A2a) We extended the analysis described in Figure 38 of the vignette removing 20%, 30% and 50% of the cells. The results were added as Figure 40 and commented in vignette text: "The effect of the perturbations induced in the clustering upon the removal of 10%, 20%, 30% and 50% of the data set was also investigated in SetA (annotated_bmsnkn_5x100cells.txt), Figure 40. We can observe that the overall cell stability score of each cell in each cluster is reduced increasing of the fraction of cells removed in each permutation. However, the reduction in CSS is not identical for all clusters. In Figure 40, it is clear that cluster 2, completely composed of NK cells, is the most stable cluster to the perturbations induced by increasing the number of removed cells. On the other side, cluster 4, mainly made by stem cells (92 cells), together with few B-cells (2 cells) and Monocytes (7 cells) is the least stable. Sorting by increasing CSS the cells in cluster 4, Figure 40D, all B-cells and Monocytes are found within the first 15 most unstable cells."
Q2b) Furthermore, it is crucial to link the CSS to the clustering ground truth with the underlying assumption that the true discovery rate for "stable" cells is larger than the one for cells with lower CSS.
A2b) We have investigated the clusters composition of two sets of cells described in section 4.2: SetA and SetC. SetA is composed by cells with different biological characteristics, i.e. (B) B-cells, (M) Monocytes, (S) Stem cells, (NK) Natural Killer cells, (N) Naive T-cells. SetC contains Monocytes, Natural Killer cells and with T-cells subpopulations, i.e. (C) Cytotoxic T-cells, (H) T-helper cells and Naive T-cell. The results are summarized in Figure 41 and commented in the vignette text: "We investigated clusters composition of two sets of cells described in section 4.2: SetA and SetC.
SetA composed by cells with different biological characteristics, i.e. (B) B-cells, (M) Monocytes, (S) Stem cells, (NK) Natural Killer cells, (N) Naive T-cells.
SetC contains Monocytes, Natural Killer cells and with T-cells subpopulations, i.e. (C) Cytotoxic T-cells, (H) T-helper cells and Naive T-cell, Figure 41. CSS provides indications on clusters cells heterogeneity. In Figure 41A and C, clusters characterized by high cell stability score are mainly constituted by one cell type. On the other hand, as CSS decrease, Figure 41B and D, the clusters become characterized by an increasing heterogeneity in the cell types composition. E.g. In Figure 41B and D, cluster 5 (violet) has a CSS between 75% to 100% and it is composed only by monocytes, cluster 2 (light green), which has a CSS between 50% and 75%, has a 11% contamination of T-helper cells. Cluster 3 (green), which has a CSS between 25 to 50%, is contaminated by 12% Cytotoxic T-cells and 4% T-helper cells. Finally, clusters 1 and 4, characterized by CSS between 0% to 25%, are very heterogeneous incorporating 4 out of 5 cell types present in this dataset.".
Q3) The authors should discuss why they chose to store e.g. normalized data and analysis results in .csv or .txt files rather than using slots of a S4 class (in sparse matrix format) commonly used in R data analysis.
A3) All tools embedded in rCASC use as input source a tab or a comma delimited file and only Seurat imports counts table file in an object of class Seurat. 10XGenomics distributes counts table file also in H5 (Hierarchical Data Format 5 File) format, Bioconductor rhdf5 package offers support for this type of files, but none of the tools implemented in rCASC is able handle the R data structure returned by the rhdf5 package. Since, as far as we know there is not a general agreement on the format of single cell count tables we prefer to wait till an agreement on standardized data format will become available.
Q4) In the vignette is it often not clear what the scale bars or colour coding means. The authors should expand figure legends, plots and axes with the necessary information to understand what is displayed.
A4) We carefully revised the figure legend to clarify colours and legend bars.

Q5) When performing dimensionality reduction it is important to i) correctly normalize the data and ii) indicate if the counts were log-transformed. These information are missing in some parts of the vignette. For example the authors use a wrapper function to perform PCA on page 17. It is not clear if the data was normalized and log-transformed which could have an impact on the interpretability of the results.
A5) We carefully revised the various part of the vignette and we added information referring to the data characteristics.
Q6) The scannobyGtf function performs gene annotation and removes mitochondrial genes and genes encoding ribosomal proteins. For some analyses, these genes can be informative regarding the metabolic and proliferative state of the cell and should not be removed. The authors should therefore consider splitting the scannobyGtf function into two functions; one for annotation and one for filtering. If users detect unwanted variation in the expression of genes encoding for ribosomal proteins, this effect should be removed using regression approaches such as scLVM rather than excluding the genes from the dataset.
A6) The scannobyGtf function was updated to make optional the removal of mitochondrial and ribosomal protein genes.
Q7) Section 3.5 Top expressed genes: By selecting the set of top expressed genes the authors might be biased by the variation detected in highly expressed housekeeping genes. Instead, and in line with commonly performed analysis for scRNA-Seq data, the authors should include an approach to detect highly variable genes as the set of informative genes. This also facilitates the inclusion of cell-type specific genes in the clustering approach (see paragraph on page 39 in the vignette).
A7) We thank reviewer for this useful comment. We have added the option to filter the dataset on the basis of gene dispersion in the topx function. Section 3.5: “For clustering purposes user might decide to use the top expressed genes. The function topx selects the X top expressed genes given a user defined threshold.”
was modified in:
“For clustering purposes user might decide to use the top expressed/variable genes. The function topx provides two options:
- the selection of the X top expressed genes given a user defined threshold, parameter type="expression"
-the selection of the X top variable genes given a user defined threshold, parameter type="variance"
The function also produces a pdf file gene_expression_distribution.pdf showing the changes in the UMIs/gene expression distribution upon topx filtering.”
Q8) When toy datasets are used it is important to state if these are the data from the original publication or if the data were pre-processed (see bottom of page 29 in the vignette).
A8) We updated the description of the toy experiments
Q9) The authors implemented different clustering strategies in the rCASC toolbox. While the cell stability score is explained in detail, it is not clear what exactly is used for SIMLR and tSNE clustering. Both are methods to perform non-linear dimensionality reduction and only SIMLR is designed to also perform clustering. tSNE is not a clustering tool and it is well known that it introduces artefacts when visualizing complex data. It would therefore be good to explain if the SIMLR internal clustering approach is used or if the authors perform k-means clustering on the dimensionality reduced data-points. For reasons of scalability and the number of input genes, I wonder whether the SIMLR approach is suitable for large dataset (e.g. 50,000 cells).
A9) In vignette section 5 and 6 we described SIMLR and Seurat as the two clustering tools used in rCASC. The choice of these two tools is given by the comparisons performed by Wang and coworkers in their paper on SIMLR (Nat Methods. 2017) and by the independent observations of Freytag and coworkers
(https://f1000research.com/articles/7-1297/v2)
and
Duo’
and
Robinson
(https://f1000research.com/articles/7-1141/v2) indicating that, in their tests, Seurat delivered the overall best performance in cells clustering. The above-mentioned references are indicated in section 5 and 6.
In section 5 we described that bootstraps are used to estimate the cell stability score using SIMLR. To clarify that also in Seurat clustering the bootstraps are implemented we added the following phrase in section 6: “The bootstrap approach described in section 5.1 is also applied to Seurat clustering to assign cell stability score to the clustered cells.”
In
rCASC

tSne
is
implemented
using
the
Rtsne
package
(https://cran.r-
project.org/web/packages/Rtsne/index.html). The tSne implementation is only used for comparison with respect to SIMLR and Seurat in section 6.1. Rtsne package provides an internal k-mean clustering, which is performed on the dimensionality reduced data-points. To clarify this point we added the following phrases in section 6.1: "The bootstrap approach described in section 5.1 is also applied on tSne clustering to assign cell stability score to the clustered cells. In rCASC tSne is implemented using the Rtsne (https://cran.r-project.org/web/packages/Rtsne/index.html) package. Rtsne performs a data reduction on which k-mean clustering is applied."
Concerning scalability and the number of input genes a similar request was also asked by reviewer 1. Therefore, we run a scalability test using the GSE106264 dataset described in Section 8 of the vignette. The scalability analysis is described in the main manuscript in materials section subsection scalability: "To estimate the scalability of rCASC clustering we used the GSE106264 dataset made of 10035 cells and published by Pace and coworkers in 2018 [18]. We randomly sampled the 10035 cells (27998 ENSEMBL GENE IDs) to obtain the following subsets of cells: 400, 600, 800, 1000, 2000, 5000. Starting from the 800 cells set we randomly sampled the genes: 10000, 8000, 6000, 4000, 2000, 1000, 800. We run SIMLR, tSne, griph and Seurat using 160 permutation within SeqBox hardware [26]: Intel i7 3.5GHz (4 cores), 32 GB RAM and 500 GB SSD disk. SIMLR resulted to be the slowest and, given the used workstation, it cannot allocate for the analysis more than 2000 cells (Figure 7A). All the other tools were able to handle up to 5000 cells, within the limit of 32 GB of RAM available in the hardware setting used in this analysis. Computation time was nearly linear for all tools till 1000 cells. Only griph clustering resulted to be nearly insensitive to the increasing number of cells (Figure 7A). The computing time as function of increasing number of genes has a quite limited effect on the overall computing time (Figure 7B)."
Q10) There are typos and phrasing issues in the figure legends and the vignette. For example, the legend of figure 2 needs editing to make it understandable.
A10) We thank reviewer for the careful and precise reading of our manuscript. We edited the vignette to eliminate typos and phrasing issues.
Minor comments
Q1) The processing of raw sequencing data in the form of fastq files is computationally expensive and usually performed on high performance computing systems. The authors decided to implement wrapper functions to process 10X and inDrop data. While these technologies are used by the majority of the field, other technologies generate individual fastq files per cell and a function could be implemented to process this data. Furthermore, the authors use the pre-build genomes supplied by 10X for the cellranger pipeline but on the other hand build the reference for the inDrop pipeline from scratch. These approaches are not comparable since the 10X genomes are filtered to only include protein-coding genes. The authors should therefore implement a wrapper function for the cellranger mkref call to allow a more flexible use of genomic references.
A1) We thank reviewer for the useful comments. Concerning the processing of fastq data we implemented inDrop and 10XGenomics fastq processing software because they are compliant with the minimal hardware requirements indicated in Section 1.1.
For smart-seq we have added into the vignette the following paragraph: "Section 2.3 Smart-seq full transcript sequencing.
Smart-seq protocol generates a full transcript library for each cell, i.e. a fastq file for each cell. To convert fastq in counts we suggest to use rnaseqCounts or wrapperSalmon counts from docker4seq package [Kulkarni et al.]. Both above-mentioned functions are compliant with minimal hardware requirements indicated for rCASC and are part, as rCASC, of the Reproducible Bioinformatics Project.
The function rnaseqCounts is a wrapper executing on each fastq:
• quality evaluation of fastq with FastQC software,
• trimming of adapters with skewer,
• mapping reads on genome using STAR and counting isoforms and genes with RSEM.
The function wrapperSalmon instead implements FastQC and skewer and calculates isoforms and genes counts using Salmon software."
We have also implemented a new function called cellrangeIndexing, which allows the generation of 10Xgenomics compliant reference genome. The description of cellrangeIndexing was added to the vignette in Section 2.2.

Q2) The framework is developed to run on a linux machine and it would be useful to provide an implementation for Mac and Windows.
A2) The rCASC framework was developed to be compliant with SeqBox (Beccuti et al. Bioinformatics 2017), i7 3.5GHz, 32GB RAM, 500 GB SSD running linux. We have tested rCASC with the latest version of Docker Desktop (v 2.0.0.3) on a Mac machine with 16 GB RAM i7 GHz 3.5, 4 cores. Configuring the virtual machine to use 12 GB RAM and 2 cores we can execute all examples provided in the rCASC vignette. However, RAM requirements become quite demanding, exceeding 12 GB, when more than 1000 cells are used for clustering. We decided to not extend the rCASC framework to window platform. Instead, within the Elixir framework, we are in the early phase of porting rCASC (https://github.com/pmandreoli/rCASC_wrappers) in LANIAKEA galaxy (https://elixir-italy-science-gateway.cloud.ba.infn.it/) in collaboration with LANIAKEA's developers.
Q3) On page 16 in the vignette, the authors discuss the relationship between the number of reads per cell and the number of genes detected. While this dependency is known, the authors should acknowledge that different cell-types show differences in their transcriptional rate and that a technical assessment of the reads per cell vs. genes detected is difficult to perform when comparing different cell-types.
A3) We thank reviewer for this important indication. To incorporate reviewer's suggestion, we added in the page 16 the following phrase: "However, it has to be underlined that each cell type is characterized by a peculiar transcriptional rate and therefore the technical assessment of the reads per cell vs. genes detected between different cell types, i.e. Figure 13A-C, might be bias by differences in the transcriptional rate of the different cells used in this specific example. Instead, the above-mentioned bias does not affect Figure 13D-F because they are generated by a down sampling of a set of cells sequenced with the smart-seq protocol at a coverage of 1 million reads/cell."
Q4) Figure 16, page 20: It is not possible to identify the cells that were removed after filtering.
A4) In Figure 16 the removed cells are those labelled in blue. Figure 16 was modified adding arrows to better highlight cells that were removed and Figure 16 legend: "Effect of Lorenz filtering, cells shown in blue have been discarded because of their low quality"
was modified in the following way:
"Lorenz filtering: cells retained after filtering are labelled in red as instead cells discarded because of their low quality are labelled in blue."
Q5) Figure 21 needs more explanation in the figure legend
A5) the phrase: "checkCountDepth output plot" was modified in: "checkCountDepth output plot provides an evaluation of count-depth relationship in un-normalized data. The effects of the normalization procedure is shown in the following figure."
Q6) It is not possible to see the CSS for individual cells as displayed by the authors. I would recommend displaying a side-by-side plot where cells in one plot are coloured by cluster ID and cells in the other plot are coloured based on their CSS.
A6) A new plot was added to the NameOfCountMatrix_Stability_Plot.pdf file, which provides the results of the clustering. The new plot provides the cluster picture with cell coloured on the basis of their CSS. Figure 38 was also modified to include this new plot. CSS is described with the following colours: 0-25% black, 25-50% green, 50-75% gold and 75-100% red. The phrase: "The plot in Figure 38C provides a 2D view of the clustering results. In this plot each cell is labeled with a symbol indicating its cell stability score."
was modified in the following way:
"In each clustering folder there is a pdf named NameOfCountMatrix_Stability_Plot.pdf, which contains two plots (Figure 38C-D) generated by the clustering program. These plots provide a 2D view of the clustering results from two different perspectives. In Figure 38C plot each cell is coloured on the basis of the belonging cluster and it is labeled with a symbol indicating its cell stability score (CSS). Instead, in the plot in Figure 38D each cell is coloured on the basis of its CSS: 0-25% black, 25-50% green, 50-75% gold and 75-100% red."
Q7) Page 51 in the vignette: there is a broken link to one figure
A7) We fixed it
Q8) There is a colouring discrepancy between Figure 51 (Z score transformed counts) and Figure 54 (log10-tranformed counts) where the rCASC uses the same colour scale.
A8) Now the colours of Fig. 51 B and C correspond to those in Fig. 54 B and C.
Q9) Page 66 in the vignette: the authors should better explain why they chose k=6 and not k=7 and where the difference between Figure 57 A and B is coming from.
A9) To clarify the description of fig 57, the phrase:
"In Figure 57 are summarized the results of the analysis executed on the Pace's dataset. Data perturbations, Figure 57A, allows data organization between 6 to 9 clusters, where 7 clusters is the most represented group. Cell stability score, from the SIMLR analysis executed on the above range of clusters, is shown in Figure 57B. Six clusters show a slightly higher stability with respect to the

others. The overall stability of 6 clusters is however sub-optimal, since it is spread between 0 and 0.9 cell stability score. In Figure 57C it is shown the clusters structure generated with SIMLR on 6 clusters. Clusters 1, 3 and 4 show a quite good stability, Figure 57C. Cluster 3 is made of 44 N (88%) and 48 Nd (96%), suggesting that naive CD8+ T lymphocytes are not affected by the silencing of Suv39h1 gene. Cluster 1 contains 16 NA (6.4%) and 14 NdA (5.6%). Cluster 2 is made of 44.8% of NA and 39.6% of NdA cells. Clusters 1 and 2 group together, interdependently from Suv39h1 gene silencing. Cluster 6 is made of 35% NA and 13.6% of NdA. In cluster 6 the amount of NA and NdA is unbalance, suggesting that the Suv39h1 silencing does not guarantee the efficient differentiation of the cell subpopulation in cluster 6. Cluster 4 only contains NdA (33%), indicating that at least a subpopulation of activated Suv39h1-silenced cells has a specific transcription profile that differentiate them from the wild type activated cells. Cluster 5 is made of 6 N cells, 2 Nd cells, 34 NA cells, 21 NdA cells. Despite the presence of a limited amount of naive cells, which might be explained as partially activated, cluster 5 is made mainly of activated cells, i.e. 13.6% NA and 8.4% NdA of total cells. The cluster structure (Figure 57D) and cell cluster stability scores (Figure 57C) might suggest that cluster 5 is made of a precursor subset."
was modified in the following way:
"In Figure 57 are summarized the results of the analysis executed on the Pace's dataset. A limitation of the clustering based on SIMLR is due to the need of providing as input the number of clusters (k) in which the data should be organized. Instead of asking to user to define arbitrarily the k number of clusters, we used griph (https://github.com/ppapasaikas/griph) as tool to identify a range of k clusters to be inspected by SIMLR. Figure 57A shows the frequency of the k number of clusters, in which the Pace's dataset can be organized using griph software, upon 160 bootstraps in which 10% of the cells is randomly removed from the initial data set. Griph analysis identify a range of clusters going from k=6 to k=9. K=7 is the most represented data organization detected by griph, followed by 8, 6 and 9 clusters.
The range of k clusters detected using griph is then investigated with SIMLR. SIMLR is run for each k of the k-range defined with griph tool. CSS violin plot (Figure 57B) shows that the mean stability for k=6 (CSSm ~ 0.5) is higher than to the others ks (CSSm < 0.3).
Clusters k=7 and k=8 do not represent the most stable organizations in terms of CSS (Figure 57B), although they are the most frequent organizations observed in griph analysis (Figure 57A).
Since the best CSSm is observed in k=6, we explored these clusters (Figure 57C). In Figure 57C, clusters 1, 3 and 4 show a quite good stability, since cells stay in these clusters between 75% to 100% of the bootstraps.
The inspection of Pace's experiment groups organization (i.e. N= naïve WT, Nd= naïve Suv39h1 KO, NA=activated WT, NdA=activated Suv39h1 KO) in k=6 clusters, Figure 57C, show that cluster number 4 is the only one containing only NdA (33% of the total NdA) cells. Thus, suggesting that a subpopulation of activated Suv39h1-silenced cells has a specific transcription profile, which differentiates them from all wild type activated cells. Another interesting cluster is number 6, where the amount of NA and NdA is unbalance, 35% NA and 13.6% of NdA, suggesting that Suv39h1 silencing does not guarantee at the same efficiency the differentiation of this cell subpopulation as in the case of wild type cells. Cluster 5 is the most heterogeneous cluster. It is composed by 6 N cells, 2 Nd cells, 34 NA cells, 21 NdA cells. Despite the presence of a limited number of naive cells, which might be explained as partially activated, cluster 5 is composed by an unbalance number of activated cells, i.e. 13.6% NA and 8.4% NdA of total cells. However, since cluster 5 is characterized by a very low CSS (0-25%) it is possible that this cluster contains cells localized at the boundaries of clusters 2, 3 and 6. On the other side clusters 1, 2, 3 have nearly the same number of wild type and Suv39h1 silenced cells, suggesting that these subsets of cells are not influenced by Suv39h1 silencing:

•
•
•

cluster 1 contains nearly the same amount of activated wild type, 16 NA (6.4%), and Suv39h1 KO cells, 14 NdA (5.6%); cluster 2 is made of 44.8% of NA and 39.6% of NdA cells;
cluster 2 is made of 44.8% of NA and 39.6% of NdA cells;
cluster 3 is made of 44 N (88%) and 48 Nd (96%)"

Close
