# Supplementary material for: rCASC: reproducible classification analysis of single-cell sequencing data
Source: Gigascience. 2019 Sep 8;8(9):giz105. doi: 10.1093/gigascience/giz105 (PMC6732171; doi:10.1093/gigascience/giz105)
Supplement: giz105_Response_to_Reviewer_Comments_Revision_1 [file giz105_response_to_reviewer_comments_revision_1.pdf]

Author's Response To Reviewer Comments

Close

Rebuttal letter for the paper:

rCASC: reproducible Classification Analysis of Single Cell sequencing data.

Luca Alessandrì, Francesca Cordero, Marco Beccuti, Maddalena Arigoni, Martina Olivero, Greta Romano, Sergio Rabellino,Nicola Licheri, Gennaro De Libero, Luigia Pace and Raffaele A Calogero

Dear Editor,
First of all, we wish to thank the reviewers for their valuable comments and useful suggestions which helped us to substantially improve the paper and its associated tool.

Hereafter we report our answers to the reviews' comments.

Reviewer #1

First, I would like to thank the authors for the hard and great work they did to address the different major aspect previously mentioned. Notably, they evaluated the time complexity of random, artificially created datasets, using 160 permutations, using 6 different sizes for both features (800 to 10000) and number of cells (from 400 to 5000). They also rewritten several sections of the manuscript such as the CSS part, and corrected different concerning aspects (such as the "supervised clustering" designation). They also simplified their pipeline and added a "mini" option to be able to download only a few Docker containers on all the available containers of rCASC which are enough to provide basic functionalities to handle single-cells. Finally, they investigated the relationship between stability and cluster significant with additional experiments. In the other hand, I am a little bit disappointed by the performance of rCASC in term of scaling-up. Using rCASC, it seems difficult to process and cluster more than 5K cells with a reasonable machine, such as a personal computer of a cluster node (with RAM up to 64GiG), and in a reasonable time (less than 24-48h). Unfortunately, I was expecting this result because rCASC uses a lot of tools that at some point require the computation of a step having a polynomial complexity. For example, SIMLR require the computation of a cell-cell distance matrix. Increasing the number of cells gives rapidly a matrix that either takes a very long time to compute /and/or cannot be loaded in the RAM of the computer. Being able to process a larger number of cells is I think a very important aspect of any new single-cell bioinformatic pipeline because A) the technology is growing rapidly and we can expect a larger amount of cells to be processed in the near future, and B) more and more single cell datasets are available leading to meta-analyses combining multiple single-cell datasets.
My first comment is: is there any settings of rCASC that can be used to process a dataset larger than 5K cells? Ideally 100K (See for example Scanpy pipeline that can process up to 1M) would be a good number. However, I think that at least 10K cells is a minimum. For example, it seems that Griph is a method the less greedy in term of resources used. Then, maybe a specific set of settings designated for "very_large_dataset" processing can be used with Griph to be able to compute larger dataset? In that case, not all the options of rCASC need to be used (especially the ones using a lot of resources). Is it possible to adapt the stability metric to such datasets? However, if the authors think that clustering datasets larger than 5K cells is out of scope of this study what would be the reason to justify the non-scalability of the method? I agree that rCASC allows a very rigorous and complete analysis of a smaller dataset but is the processing of 1 to 5K cells enough considering the single-cell RNA-Seq field evolution (it is an open question)?

Answer #1:
We incorporated in rCASC the clustering tools scanpy and griph as required by the reviewer. Moreover, we tested the performance of rCASC using Seurat, griph, and scanpy tools considering 10K, 35K, 68K and 101K cells. The analysis was executed on an SGI server (10 x CPU E5-4650 2.4GHz (16 cores), 1TB RAM, 30 TB SATA raid disk) allocating 40 threads for each analysis.
The results of this comparison highlight that griph works slightly faster than Seurat tool. However, both

these tools do not scale well when more than 68K cells are analyzed. Differently scanpy shows the best performance up to 101K cells.
Hence, figure 7 was redraw to report the new results, and its caption “Scalability analysis of clustering tools implemented in rCASC. A) Time required to perform 160 permutations as function of increasing number of cells on a set of 27998 genes. B) Time required to perform 160 permutations as function of increasing number of genes on a set of 800 cells.” was updated in the following way:
“Scalability analysis of the clustering tools implemented in rCASC. A) Time required to perform 160 permutations as function of increasing number of cells on approximately 20,000 genes. Left panel: SIMLR, tSne, Seurat and griph clustering up to 5,000 cells was executed on a SeqBox [7] (1 x CPU i7-6770HQ 3.5 GHz (8 cores), 32 GB RAM, 1TB SSD). Right panel: Seurat, griph and scanpy analyses were extended until 101,000 cells using an SGI server (10 x CPU E5-4650 2.4GHz (16 cores), 1TB RAM, 30 TB SATA raid disk). B) Time required to perform 160 permutations as function of increasing number of genes on a set of 800 cells, analysis performed on a SeqBox.”

Moreover, Scalability section in the main manuscript “To estimate the scalability of rCASC clustering we used the GSE106264 dataset made of 10035 cells and published by Pace and coworkers in 2018 [21]. We randomly sampled the 10035 cells (27998 ENSEMBL GENE IDs) to obtain the following subsets of cells: 400, 600, 800, 1000, 2000, 5000. Starting from the 800 cells set we randomly sampled the genes: 10000, 8000, 6000, 4000, 2000, 1000, 800. We run SIMLR, tSne, griph and Seurat using 160 permutation within SeqBox hardware [7]: Intel i7 3.5GHz (4 cores), 32 GB RAM and 500 GB SSD disk. SIMLR resulted to be the slowest and, given the above hardware implementation, it cannot allocate for the analysis more than 2000 cells (Figure 7A). All the other tools were able to handle up to 5000 cells within the limit of 32 GB of RAM available in the hardware setting used in this analysis. Computation time was nearly linear for all tools till 1000 cells. Only griph clustering resulted to be nearly insensitive to the increasing number of cells (Figure 7A). The computing time as function of increasing number of genes has a quite limited effect on the overall computing time (Figure 7B).” was updated as follows:
“To estimate the scalability of rCASC clustering we used the GSE106264 dataset made of 10,035 cells and published by Pace and coworkers in 2018 [23] and the 10,000/33,000/68,000 cells PBMC human datasets, available at 10xGenomics repository (www.10xgenomics.com).
We randomly generated from the 10035 cells (27998 ENSEMBL GENE IDs) the following subsets of cells: 400, 600, 800, 1000, 2000, 5000. Moreover, for the subsets with more than 600 cells we randomly sampled the genes: 10000, 8000, 6000, 4000, 2000, 1000, 800. We run SIMLR, tSne, griph and Seurat using 160 permutations within SeqBox hardware [7]: Intel i7 3.5GHz (4 cores), 32 GB RAM and 500 GB SSD disk. SIMLR resulted to be the slowest and, given the above hardware implementation, it cannot allocate for the analysis more than 2000 cells (Figure 7A left panel). All the other tools were able to handle up to 5000 cells within the limit of 32 GB of RAM available in the hardware setting used in this analysis. Computation time was nearly linear for all tools till 1000 cells. Only griph clustering resulted to be nearly insensitive to the increasing number of cells (Figure 7A). We extended, for Seurat, griph and scanpy, the scalability analysis to 10K, 33K, 68K and 101K cells, using 10,000/33,000/68,000 cells from PBMC human datasets, available at 10xGenomics repository (www.10xgenomics.com), and 101,000 cells dataset, made assembling the above mentioned 33,000 and 68,000 PBMC datasets. The analysis was executed on a SGI server (10 x CPU E5-4650 2.4GHz (16 cores), 1TB RAM, 30 TB SATA raid disk) allocating 40 threads for each analysis. Scanpy outperforms the other two methods and griph behaves slightly better than Seurat (Figure 7A right panel).
The computing time as function of increasing number of genes has a quite limited effect on the overall computing time (Figure 7B).”

Finally, the supplementary file was updated adding all the new functionalities implemented as consequence of the reviewer’s requests.

Reviewer #2

The authors have now extensively revised the manuscript and added a number of useful functions to the existing rCASC package. By doing so, the authors addressed the majority of my previous comments. However, there remain a few minor comments that should be addressed prior to publication.

1) Relating to the previous major comment 1: The publication associated to the griph package can now be cited as: Serra et al., Self-organization and symmetry breaking in intestinal organoid development. Nature (2019).

Answer 1:
we updated the reference.

2) Relating to the previous major comment 7: It would be useful to provide more details on which variability measure (variance, squared coefficient of variation or a regression residual) is used to rank genes based on their variability in the topx function while setting type="variance".

Answer 2:
we updated the description of topx function in the supplementary material specifying that user can decide to use the top expressed/variable genes for clustering. The function **topx** provides the selection of the X top expressed genes given a user defined threshold.

Hence, in the supplementary material the following sentences were revised:

“For clustering purposes user might decide to use the top expressed/variable genes. The function **topx** provides two options:
the selection of the X top expressed genes given a user defined threshold, parameter type="expression"
the selection of the X top variable genes given a user defined threshold, parameter type="variance"

+ gene variance is calculated using edgeR Tag-wise dispersion. The method estimates the gene-wise dispersion implementing a conditional maximum likelihood procedure. For more information please refer to edgeR Bioconductor package manual.”

3) Relating to the previous major comment 10: The legend of Figure 2 in the main manuscript has not been corrected for spelling and phrasing mistakes yet.

Answer 3: The legend of Figure 2 was revised as suggested by the reviewer.

Close
