# Supplementary material for: rCASC: reproducible classification analysis of single-cell sequencing data
Source: Gigascience. 2019 Sep 8;8(9):giz105. doi: 10.1093/gigascience/giz105 (PMC6732171; doi:10.1093/gigascience/giz105)
Supplement: giz105_Response_to_Reviewer_Comments_Revision_2 [file giz105_response_to_reviewer_comments_revision_2.pdf]

# Author's Response To Reviewer Comments

Close

rCASC: reproducible Classification Analysis of Single Cell sequencing data.

Luca Alessandrì, Francesca Cordero, Marco Beccuti, Maddalena Arigoni, Martina Olivero, Greta Romano, Sergio Rabellino,Nicola Licheri, Gennaro De Libero, Luigia Pace and Raffaele A Calogero

Dear Editor,
First of all, we wish to thank the reviewers for their valuable comments and useful suggestions which helped us to substantially improve the paper and its associated tool.

Hereafter we report our answers to the reviews' comments.

Reviewer reports:

Reviewer #1: The authors incorporated additional clustering methods (Scanpy and Griph) that prove to be scalable for datasets having larger sizes which corresponds to the field needs.
In particular, Scanpy seems to reveal no issue to scale up to 100K cells in the benchmark executed opposite to the other methods.
I recommend accepting this manuscript since I think it is well suited for current and future analytical needs for single cells.

Minor comments:

Question 1: Is there any limitation or trick to use for the preprocessing procedures (low cell quality filter, normalization, annotation, cell cycle removal, matrix creation) executed before the clustering when increasing the sample / feature size?
I presume no because the authors have used them with large dataset. Then, It will be worth metioning that in the manuscript with a brief estimate of the computational time / memory needed.

Answer 1: All samples were preprocessed removing ribosomal/mitochondrial protein genes and cells with a total count of UMIs lower than 100. This information was added in the scalability paragraph: "All the above samples were preprocessed removing ribosomal/mitochondrial protein genes and cells with a total count of UMIs lower than 100."
Concerning the computational time/memory required for the analysis we added the following phrase at the end of Scalability paragraph:
"The definition of the computing time for an analysis depends on multiple parameters: i) the number of permutations performed in parallel, ii) the number of cells under analysis, iii) the clustering tool in use and iv) the hardware used for the analysis. Concerning the amount of RAM required for each permutation run in parallel, up to 5000 cells the maximum amount of RAM required is approximately 4 GB, from 10000 to 100000 cells, the maximum RAM required is approximately 20 GB. Independently by the clustering approach and the size of the dataset, we suggest to run at least 100 permutations to correctly estimate CSS."

Question 2: The figure 3 is not updated with Scanpy and griph.

Answer2: We updated Fig. 3 as suggested by the reviewer. Moreover, we updated Fig. 4C which now includes griph and scanpy functions
Question 3: I don't understand the use of the term hierarchical clustering in the manuscript and in the suppl. material.

Answer 3: We removed the term "hierarchical" from Fig. 1 and in supplementary data.

------------------------------------------------------------------------------------------------------------------------
-------
Concerning software repository:
------------------------------------------------------------------------------------------------------------------------
-------

Dear Editor,
hereafter we reported general information about the current software repositories:

1. The rCASC package is available at this github repository: https://github.com/kendomaniac/rCASC
2. All the docker images are stored in the docker hub:
docker.io/repbioinfo/
3.GUI for rCASC is available at this github repository:
https://github.com/mbeccuti/4SeqGUI
4. All the sample data are retrievable at 130.192.119.59 and the paths are indicated in the supplementary material.

Moreover we registered rCASC in bio.tools and in SciCrunch.org (id: SCR_017005)

Close
