# Supplementary material for: rCASC: reproducible classification analysis of single-cell sequencing data
Source: Gigascience. 2019 Sep 8;8(9):giz105. doi: 10.1093/gigascience/giz105 (PMC6732171; doi:10.1093/gigascience/giz105)
Supplement: giz105_Reviewer_1_Report_Original_Submission — Olivier Poirion, Ph.D. -- 1/22/2019 Reviewed [file giz105_reviewer_1_report_original_submission.pdf]

Reviewer Report

Title: rCASC: reproducible Classification Analysis of Single Cell sequencing data

Version: Original Submission **Date:** 1/22/2019

Reviewer name: Olivier Poirion, Ph.D.

Reviewer Comments to Author:

This paper presents a pipeline to to infer single-cell clusters using scRNA-Seq data (rCASC), infer significant features linked to each cluster, and can analyse various metrics during the processing. Notably, the results of the pipeline can be divided into 3 major outputs, A) cells-features matrix generation, B) Clustering, and C) inference of significant features per clusters. Also, the pipeline is able to perform various additional substeps such as Matrix preprocessing (normalization), outliers removal, features removal, cell cycle specific features removal. The pipeline is implemented in R using Docker containers and has a GUI interface coded in Java. Finally; the authors claimed have invented a metric: the CSS, to evaluate cluster stability in their single-cell analyses. First, It is a pleasant surprise to be able to install everything needed to perform scRNA-Seq analysis with few simple commands (with exception of Docker which can be tricky for non IT people). Also, developing scRNA-Seq analytical toolbox easy to use and efficient are an innovative direction due to the importance and the multidisciplinary aspect of the field. However, I have major concerns which I think should be addressed before publication.
Major Comments:
First, the abstract and the text contain different confusing aspects that must be rewritten. The authors describe a "supervised approach": SIMLR which is seen as the alternative of the "Seurat clustering". From my knowledge, SIMLR is a clustering workflow and thus is also an unsupervised approach, by contrast with any other supervised approaches using training datasets as input (classification/regression…). I don't know what is a "supervised clustering" if not a classification procedure. Clustering are always unsupervised with the exception of "semi-supervised" clustering (use of seed samples). Also, I don't understand why this package is superior in term of "Computational and Functional" reproducibility compared with any other packages for which a similar reasoning can be also applied.
Then, the authors claimed to have invented a new metric: the "Cell-stability Score", which is based on the computation of a stability score by clustering multiple bootstrap sampling and computing the jaccard index. Clustering stability measurement is not new and previous works already described more formally the use of bootstrapping together with clustering and Jaccard index to estimate cluster stability (http://www.homepages.ucl.ac.uk/~ucakche/papers/clusta.pdf (2006), https://arxiv.org/abs/1503.0205). These example algorithms are not based on single-cell datasets (other stability approaches exist for single-cells), but since the approach described in the first paper is very similar, a more comprehensive bibliography of clustering stability should be present in the manuscript as well a rewriting of the CSS description/notion, highlighting the similarity with previous works.
In term of additional experiments, I think it would be interesting to have an idea of the ratio: number of

CPUs/ RAM/ computational time according to: the number of cells / number of features (i.e.: matrix dimension), and read depth (linked to fastq size). More specifically, what are the limiting steps in term of computation? What are the steps the less expensive ? A new figure might be necessary to represent the contribution of each step in term of computation. Ideally, a comparison with the other cited pipeline would be also interesting, but this amount of work might be out of scope of this study.
I have some concerns with the choice of clustering algorithms used. Despite Seurat is well established in the community and SIMLR is also a well recognized algorithm, I am not sure if these algorithms can handle very large sparse datasets (i.e. more than 10K cells) , that are becoming the new standard in the field. Notably, are these algorithms able to handle sparse data? SIMLR needs a specified K, thus inferring the best K requires to screen amongst an array of Ks and thus might be very time consuming. Would it exists better and simpler alternatives to handle very large and sparse datasets that might be included in rCASC?
Using a clustering stability metric is I think a very good idea. Is it possible to get an average stability score per cluster to have an idea if a cluster is noisy or robust? Also, even a stable cluster according to a bootstrap experiment is not a guarantee of a "biologically" stable cluster, and can reflect a biased in the method used (for example, a dummy algorithm clustering cells according to their name will produce very stable but useless clusters).
What is the use of griph (Graph Inference of Population Heterogeneity). Why not using the stability measure to estimate the best K?
The package requires a very large amount of memory to be able to install all the docker dependencies and I was not able to install it on my own computer (out of memory). Is there any way to propose "lighter" versions in order to be able to use it on a standard computer? Overall, I am not sure if all these different steps are always mandatory to obtain biologically meaningful single-cell clusters (Of course, they might be required in some specific cases), compared to more straightforward approaches (matrix creation -> embedding -> clustering).
Minor Comments:
The supplementary files document very rigorously the software which is really pleasant.
Some figures are not very informative and might be combined together (For example figures 1, 3 and 4 And figures 2 and 6?).
Can you describe briefly what is the Seurat specific normalization?

Level of Interest

Please indicate how interesting you found the manuscript: Choose an item.

Quality of Written English

Please indicate the quality of language in the manuscript: Choose an item.

Declaration of Competing Interests

Please complete a declaration of competing interests, considering the following questions:

- Have you in the past five years received reimbursements, fees, funding, or salary from an organisation that may in any way gain or lose financially from the publication of this manuscript, either now or in the future?
- Do you hold any stocks or shares in an organisation that may in any way gain or lose financially from the publication of this manuscript, either now or in the future?
- Do you hold or are you currently applying for any patents relating to the content of the manuscript?
- Have you received reimbursements, fees, funding, or salary from an organization that holds or has applied for patents relating to the content of the manuscript?
- Do you have any other financial competing interests?
- Do you have any non-financial competing interests in relation to this paper?

If you can answer no to all of the above, write 'I declare that I have no competing interests' below. If your reply is yes to any, please give details below.

I declare that I have no competing interests

I agree to the open peer review policy of the journal. I understand that my name will be included on my report to the authors and, if the manuscript is accepted for publication, my named report including any attachments I upload will be posted on the website along with the authors' responses. I agree for my report to be made available under an Open Access Creative Commons CC-BY license (http://creativecommons.org/licenses/by/4.0/). I understand that any comments which I do not wish to be included in my named report can be included as confidential comments to the editors, which will not be published.

Choose an item.

To further support our reviewers, we have joined with Publons, where you can gain additional credit to further highlight your hard work (see: https://publons.com/journal/530/gigascience). On publication of this paper, your review will be automatically added to Publons, you can then choose whether or not to claim your Publons credit. I understand this statement.

Yes Choose an item.
