# Supplementary material for: rCASC: reproducible classification analysis of single-cell sequencing data
Source: Gigascience. 2019 Sep 8;8(9):giz105. doi: 10.1093/gigascience/giz105 (PMC6732171; doi:10.1093/gigascience/giz105)
Supplement: giz105_Reviewer_1_Report_Revision_1 — Olivier Poirion, Ph.D. -- 4/30/2019 Reviewed [file giz105_reviewer_1_report_revision_1.pdf]

Reviewer Report

Title: rCASC: reproducible Classification Analysis of Single Cell sequencing data

Version: Revision 1 **Date:** 4/30/2019

Reviewer name: Olivier Poirion, Ph.D.

Reviewer Comments to Author:

First, I would like to thank the authors for the hard and great work they did to address the different major aspect previously mentioned.
Notably, they evaluated the time complexity of random, artificially created datasets, using 160 permutations, using 6 different sizes for both features (800 to 10000) and number of cells (from 400 to 5000). They also rewritten several sections of the manuscript such as the CSS part, and corrected different concerning aspects (such as the "supervised clustering" designation). They also simplified their pipeline and added a "mini" option to be able to download only a few Docker containers on all the available containers of rCASC which are enough to provide basic functionalities to handle single-cells. Finally, they investigated the relationship between stability and cluster significant with additional experiments. In the other hand, I am a little bit disappointed by the performance of rCASC in term of scaling-up. Using rCASC, it seems difficult to process and cluster more than 5K cells with a reasonable machine, such as a personal computer of a cluster node (with RAM up to 64GiG), and in a reasonable time (less than 24-48h). Unfortunately, I was expecting this result because rCASC uses a lot of tools that at some point require the computation of a step having a polynomial complexity. For example, SIMLR require the computation of a cell-cell distance matrix. Increasing the number of cells gives rapidly a matrix that either takes a very long time to compute /and/or cannot be loaded in the RAM of the computer. Being able to process a larger number of cells is I think a very important aspect of any new single-cell bioinformatic pipeline because A) the technology is growing rapidly and we can expect a larger amount of cells to be processed in the near future, and B) more and more single cell datasets are available leading to meta-analyses combining multiple single-cell datasets.
My first comment is: is there any settings of rCASC that can be used to process a dataset larger than 5K cells? Ideally 100K (See for example Scanpy pipeline that can process up to 1M) would be a good number. However, I think that at least 10K cells is a minimum. For example, it seems that Griph is a method the less greedy in term of resources used. Then, maybe a specific set of settings designated for "very_large_dataset" processing can be used with Griph to be able to compute larger dataset? In that case, not all the options of rCASC need to be used (especially the ones using a lot of resources). Is it possible to adapt the stability metric to such datasets? However, if the authors think that clustering datasets larger than 5K cells is out of scope of this study what would be the reason to justify the non-scalability of the method? I agree that rCASC allows a very rigorous and complete analysis of a smaller dataset but is the processing of 1 to 5K cells enough considering the single-cell RNA-Seq field evolution (it is an open question)?

Level of Interest

Please indicate how interesting you found the manuscript: Choose an item.

Quality of Written English

Please indicate the quality of language in the manuscript: Choose an item.

Declaration of Competing Interests

Please complete a declaration of competing interests, considering the following questions:

- Have you in the past five years received reimbursements, fees, funding, or salary from an organisation that may in any way gain or lose financially from the publication of this manuscript, either now or in the future?
- Do you hold any stocks or shares in an organisation that may in any way gain or lose financially from the publication of this manuscript, either now or in the future?
- Do you hold or are you currently applying for any patents relating to the content of the manuscript?
- Have you received reimbursements, fees, funding, or salary from an organization that holds or has applied for patents relating to the content of the manuscript?
- Do you have any other financial competing interests?
- Do you have any non-financial competing interests in relation to this paper?

If you can answer no to all of the above, write 'I declare that I have no competing interests' below. If your reply is yes to any, please give details below.

I declare that I have no competing interests

I agree to the open peer review policy of the journal. I understand that my name will be included on my report to the authors and, if the manuscript is accepted for publication, my named report including any attachments I upload will be posted on the website along with the authors' responses. I agree for my report to be made available under an Open Access Creative Commons CC-BY license (http://creativecommons.org/licenses/by/4.0/). I understand that any comments which I do not wish to be included in my named report can be included as confidential comments to the editors, which will not be published.

Choose an item.

To further support our reviewers, we have joined with Publons, where you can gain additional credit to further highlight your hard work (see: https://publons.com/journal/530/gigascience). On publication of this paper, your review will be automatically added to Publons, you can then choose whether or not to claim your Publons credit. I understand this statement.

Yes Choose an item.
