# Supplementary material for: rCASC: reproducible classification analysis of single-cell sequencing data
Source: Gigascience. 2019 Sep 8;8(9):giz105. doi: 10.1093/gigascience/giz105 (PMC6732171; doi:10.1093/gigascience/giz105)
Supplement: giz105_Reviewer_2_Report_Original_Submission — Nils Eling -- 1/31/2019 Reviewed [file giz105_reviewer_2_report_original_submission.pdf]

Reviewer Report

Title: rCASC: reproducible Classification Analysis of Single Cell sequencing data

Version: Original Submission **Date:** 1/31/2019

Reviewer name: Nils Eling

Reviewer Comments to Author:

The authors present rCASC, an integrated analysis framework for single-cell RNA sequencing data that combines a range of existing and novel computational tools. While some workflows for reproducible scRNA-Seq data analysis exist, the authors provide an analysis strategy using docker containers and a graphical user interface for reproducibility. rCASC is implemented as an R package and as graphical user interface, which allows bioinformaticians as well as biologists with little experience to perform statistical data analysis.

However, there are some concerns about the chosen analysis tools and the presentation of results that need to be addressed:

Major comments

1) The description of the algorithms used is often not clear. In the main text (p.4 l.16-21), the authors mention normalization and clustering strategies taken from other tools. When other tools are used, it would be good to briefly describe the underlying algorithms (either in the main text or the methods section). For example, Seurat is a toolbox for data analysis and not a clustering tool and it has to be specified which clustering strategy is used. The authors should also make sure to properly cite the original publication of functions implemented in the rCASC toolbox (for example the reCAT function and the griph package).

2) The authors developed the Cell Stability Score based on iteratively clustering a sub-sampled dataset. This is a good approach and informative for cluster stability. It would however be good to visualize the stability scores for datasets subsampled to 20%, 30%, and even 50%. Furthermore, it is crucial to link the CSS to the clustering ground truth with the underlying assumption that the true discovery rate for "stable" cells is larger than the one for cells with lower CSS.

3) The authors should discuss why they chose to store e.g. normalized data and analysis results in .csv or .txt files rather than using slots of a S4 class (in sparse matrix format) commonly used in R data analysis.

4) In the vignette is it often not clear what the scale bars or colour coding means. The authors should expand figure legends, plots and axes with the necessary information to understand what is displayed.

5) When performing dimensionality reduction it is important to i) correctly normalize the data and ii) indicate if the counts were log-transformed. These information are missing in some parts of the vignette. For example the authors use a wrapper function to perform PCA on page 17. It is not clear if the data was normalized and log-transformed which could have an impact on the interpretability of the results.

6) The scannobyGtf function performs gene annotation and removes mitochondrial genes and genes encoding ribosomal proteins. For some analyses, these genes can be informative regarding the metabolic and proliferative state of the cell and should not be removed. The authors should therefore

consider splitting the scannobyGtf function into two functions; one for annotation and one for filtering. If users detect unwanted variation in the expression of genes encoding for ribosomal proteins, this effect should be removed using regression approaches such as scLVM rather than excluding the genes from the dataset.

7) Section 3.5 Top expressed genes: By selecting the set of top expressed genes the authors might be biased by the variation detected in highly expressed housekeeping genes. Instead, and in line with commonly performed analysis for scRNA-Seq data, the authors should include an approach to detect highly variable genes as the set of informative genes. This also facilitates the inclusion of cell-type specific genes in the clustering approach (see paragraph on page 39 in the vignette).

8) When toy datasets are used it is important to state if these are the data from the original publication or if the data were pre-processed (see bottom of page 29 in the vignette).

9) The authors implemented different clustering strategies in the rCASC toolbox. While the cell stability score is explained in detail, it is not clear what exactly is used for SIMLR and tSNE clustering. Both are methods to perform non-linear dimensionality reduction and only SIMLR is designed to also perform clustering. tSNE is not a clustering tool and it is well known that it introduces artefacts when visualizing complex data. It would therefore be good to explain if the SIMLR internal clustering approach is used or if the authors perform k-means clustering on the dimensionality reduced data-points. For reasons of scalability and the number of input genes, I wonder whether the SIMLR approach is suitable for large dataset (e.g. 50,000 cells).

10) There are typos and phrasing issues in the figure legends and the vignette. For example, the legend of figure 2 needs editing to make it understandable.

Minor comments

1) The processing of raw sequencing data in the form of fastq files is computationally expensive and usually performed on high performance computing systems. The authors decided to implement wrapper functions to process 10X and inDrop data. While these technologies are used by the majority of the field, other technologies generate individual fastq files per cell and a function could be implemented to process this data. Furthermore, the authors use the pre-build genomes supplied by 10X for the cellranger pipeline but on the other hand build the reference for the inDrop pipeline from scratch. These approaches are not comparable since the 10X genomes are filtered to only include protein-coding genes. The authors should therefore implement a wrapper function for the cellranger mkref call to allow a more flexible use of genomic references.

3) The framework is developed to run on a linux machine and it would be useful to provide an implementation for Mac and Windows.

4) On page 16 in the vignette, the authors discuss the relationship between the number of reads per cell and the number of genes detected. While this dependency is known, the authors should acknowledge that different cell-types show differences in their transcriptional rate and that a technical assessment of the reads per cell vs. genes detected is difficult to perform when comparing different cell-types.

5) Figure 16, page 20: It is not possible to identify the cells that were removed after filtering.

6) Figure 21 needs more explanation in the figure legend

7) It is not possible to see the CSS for individual cells as displayed by the authors. I would recommend displaying a side-by-side plot where cells in one plot are coloured by cluster ID and cells in the other plot are coloured based on their CSS.

8) Page 51 in the vignette: there is a broken link to one figure
9) There is a colouring discrepancy between Figure 51 (Z score transformed counts) and Figure 54 (log10-tranformed counts) where the rCASC uses the same colour scale.
10) Page 66 in the vignette: the authors should better explain why they chose k=6 and not k=7 and where the difference between Figure 57 A and B is coming from.

Level of Interest

Please indicate how interesting you found the manuscript: Choose an item.

Quality of Written English

Please indicate the quality of language in the manuscript: Choose an item.

Declaration of Competing Interests

Please complete a declaration of competing interests, considering the following questions:

- Have you in the past five years received reimbursements, fees, funding, or salary from an organisation that may in any way gain or lose financially from the publication of this manuscript, either now or in the future?
- Do you hold any stocks or shares in an organisation that may in any way gain or lose financially from the publication of this manuscript, either now or in the future?
- Do you hold or are you currently applying for any patents relating to the content of the manuscript?
- Have you received reimbursements, fees, funding, or salary from an organization that holds or has applied for patents relating to the content of the manuscript?
- Do you have any other financial competing interests?
- Do you have any non-financial competing interests in relation to this paper?

If you can answer no to all of the above, write 'I declare that I have no competing interests' below. If your reply is yes to any, please give details below.

I declare that I have no competing interests

I agree to the open peer review policy of the journal. I understand that my name will be included on my report to the authors and, if the manuscript is accepted for publication, my named report including any attachments I upload will be posted on the website along with the authors' responses. I agree for my report to be made available under an Open Access Creative Commons CC-BY license (http://creativecommons.org/licenses/by/4.0/). I understand that any comments which I do not wish to

be included in my named report can be included as confidential comments to the editors, which will not be published.

Choose an item.

To further support our reviewers, we have joined with Publons, where you can gain additional credit to further highlight your hard work (see: https://publons.com/journal/530/gigascience). On publication of this paper, your review will be automatically added to Publons, you can then choose whether or not to claim your Publons credit. I understand this statement.

Yes Choose an item.
