# Supplementary material for: rCASC: reproducible classification analysis of single-cell sequencing data
Source: Gigascience. 2019 Sep 8;8(9):giz105. doi: 10.1093/gigascience/giz105 (PMC6732171; doi:10.1093/gigascience/giz105)
Supplement: giz105_Reviewer_2_Report_Revision_1 — Nils Eling -- 4/29/2019 Reviewed [file giz105_reviewer_2_report_revision_1.pdf]

**Reviewer Report**

**Title: rCASC: reproducible Classification Analysis of Single Cell sequencing data**

**Version: Revision 1** **Date:** 4/29/2019

**Reviewer name: Nils Eling**

**Reviewer Comments to Author:**

The authors have now extensively revised the manuscript and added a number of useful functions to the existing rCASC package. By doing so, the authors addressed the majority of my previous comments. However, there remain a few minor comments that should be addressed prior to publication.
1) Relating to the previous major comment 1: The publication associated to the griph package can now be cited as: Serra et al., Self-organization and symmetry breaking in intestinal organoid development. Nature (2019).
2) Relating to the previous major comment 7: It would be useful to provide more details on which variability measure (variance, squared coefficient of variation or a regression residual) is used to rank genes based on their variability in the topx function while setting type="variance".
3) Relating to the previous major comment 10: The legend of Figure 2 in the main manuscript has not been corrected for spelling and phrasing mistakes yet.

**Level of Interest**

Please indicate how interesting you found the manuscript: Choose an item.

**Quality of Written English**

Please indicate the quality of language in the manuscript: Choose an item.

**Declaration of Competing Interests**

Please complete a declaration of competing interests, considering the following questions:

- Have you in the past five years received reimbursements, fees, funding, or salary from an organisation that may in any way gain or lose financially from the publication of this manuscript, either now or in the future?
- Do you hold any stocks or shares in an organisation that may in any way gain or lose financially from the publication of this manuscript, either now or in the future?
- Do you hold or are you currently applying for any patents relating to the content of the manuscript?
- Have you received reimbursements, fees, funding, or salary from an organization that holds or has applied for patents relating to the content of the manuscript?

- Do you have any other financial competing interests?
- Do you have any non-financial competing interests in relation to this paper?

If you can answer no to all of the above, write 'I declare that I have no competing interests' below. If your reply is yes to any, please give details below.

I declare that I have no competing interests

I agree to the open peer review policy of the journal. I understand that my name will be included on my report to the authors and, if the manuscript is accepted for publication, my named report including any attachments I upload will be posted on the website along with the authors' responses. I agree for my report to be made available under an Open Access Creative Commons CC-BY license (http://creativecommons.org/licenses/by/4.0/). I understand that any comments which I do not wish to be included in my named report can be included as confidential comments to the editors, which will not be published.

Choose an item.

To further support our reviewers, we have joined with Publons, where you can gain additional credit to further highlight your hard work (see: https://publons.com/journal/530/gigascience). On publication of this paper, your review will be automatically added to Publons, you can then choose whether or not to claim your Publons credit. I understand this statement.

Yes Choose an item.
